# Pan-transcriptome identifying master genes and regulation network in response to drought and salt stresses in Alfalfa (*Medicago sativa* L.)

**DOI:** 10.1038/s41598-021-96712-x

**Published:** 2021-08-26

**Authors:** Cesar Augusto Medina, Deborah A. Samac, Long-Xi Yu

**Affiliations:** 1 United States Department of Agriculture-Agricultural Research Service, Plant Germplasm Introduction and Testing Research, Prosser, WA 99350 USA; 2 United States Department of Agriculture-Agricultural Research Service, Plant Science Research Unit, 1991 Upper Buford Circle, 495 Borlaug Hall St, Paul, MN 55108 USA

**Keywords:** Plant sciences, Plant stress responses, Drought, Salt, Computational biology and bioinformatics, Gene regulatory networks

## Abstract

Alfalfa is an important legume forage grown worldwide and its productivity is affected by environmental stresses such as drought and high salinity. In this work, three alfalfa germplasms with contrasting tolerances to drought and high salinity were used for unraveling the transcriptomic responses to drought and salt stresses. Twenty-one different RNA samples from different germplasm, stress conditions or tissue sources (leaf, stem and root) were extracted and sequenced using the PacBio (Iso-Seq) and the Illumina platforms to obtain full-length transcriptomic profiles. A total of 1,124,275 and 91,378 unique isoforms and genes were obtained, respectively. Comparative analysis of transcriptomes identified differentially expressed genes and isoforms as well as transcriptional and post-transcriptional modifications such as alternative splicing events, fusion genes and nonsense-mediated mRNA decay events and non-coding RNA such as circRNA and lncRNA. This is the first time to identify the diversity of circRNA and lncRNA in response to drought and high salinity in alfalfa. The analysis of weighted gene co-expression network allowed to identify master genes and isoforms that may play important roles on drought and salt stress tolerance in alfalfa. This work provides insight for understanding the mechanisms by which drought and salt stresses affect alfalfa growth at the whole genome level.

## Introduction

Alfalfa (*Medicago sativa* L.) is the most widely cultivated legume forage in the world and the third largest crop in United States, due to its high biomass production and nutritional value for livestock^1^. However, environmental factors such as drought and high salinity affect alfalfa growth, nutritive value and biomass production^2,3^. Plants respond to drought and high salinity in several ways: osmotic and ion homeostasis, which affect maintenance of turgor pressure; detoxification signaling through redox signaling; cell growth control by increasing levels in ethylene responsive transcription factors (ERF); or activating dehydration tolerance response factors. Understanding physiological and genetic mechanisms involved in drought and salt stress tolerance is highly imperative for sustainable alfalfa production.

RNA sequencing (RNA-seq) has been used to obtain transcriptional profiles and to generate de novo gene expression information. However, short read lengths is a major limitation for identifying full-length transcripts. To overcome this problem, during the past few years, the use of Pacific BioSciences (PacBio) or Oxford Nanopore Technologies platforms have become popular because they dramatically increase read length^4,5^.

The PacBio Isoform Sequencing (Iso-Seq) generates long-reading sequences that allows obtaining full-length transcripts in one single read. Iso-Seq has been used to deepen the analysis of transcriptomes in different crops^6–8^. Additionally, Iso-Seq generates information about transcriptome complexity including alternative spicing events (ASE), non-coding RNAs (ncRNAs) or nonsense-mediated mRNA decay (NMD)^9^. Long non-coding RNAs (lncRNAs) are non-coding RNAs greater than 200 bp in length with low expression and high instability^10^. lncRNAs are involved in *cis*- and *trans*-acting gene regulation processes like plant adaptation to abiotic stress^11^. Additionally, coupling Iso-Seq reads with RNA-seq data can be used to identify circular RNAs (circRNAs) and to generate gene regulation networks of highly correlated genes^12^. The circRNA is a form of non-coding RNA generated by 3′–5′ head-to-tail back-splicing and is highly stable in comparison with linear RNAs. It has been reported that circRNA is involved in many biological processes including abiotic stress response^13^.

In alfalfa, transcriptomic analysis were used to identify differentially expressed genes under different conditions including salt stress^14,15^, freezing stress^16^, and bacterial pathogen infection^17^. Most recently, Duan et al.^18^ identified hub genes and modules closely related to floral pigmentation in two alfalfa cultivars. Luo et al.^19^ generated a full-length transcriptome of alfalfa root tips of ‘Zhongmu No. 1’ under osmotic and ionic stresses. However, only parts of genes and isoforms were captured in these studies as only a single tissue source and single variety was used. Furthermore, no study has yet identified circRNAs in autotetraploid alfalfa. A robust transcriptome in alfalfa using more tissue sources and varieties with extreme responses to specific stresses is required to generate a more complete transcriptomic atlas that can be used in alfalfa genomics-assisted breeding programs.

In this research we used three germplasms, Wilson (drought resistant), Saranac (nontolerant to drought and salt), PI467895 (salt resistant) and generated 21 full-length alfalfa transcriptomes from three tissue sources (leaf, stem and root) of plants subjected to salt (SS), drought (DS) and control (CK) treatments. Our goal was to generate more complete, rigorous, and comprehensive isoform expression atlas in alfalfa response to drought and high salinity. Here we report the discovery of ASE, NMD, transcription factors and regulators, ncRNAs in alfalfa and the construction of gene regulation networks to better understand molecular mechanisms by which drought and high salinity affect plant growth and biomass production in alfalfa.

## Results

### Generation of full-length non-concatemers reads

RNA libraries were generated from 21 samples for Iso-Seq and RNA-seq. More than 5.5 terabytes of raw reads were obtained from Iso-Seq and 108.5 gigabytes of circular consensus sequences (CCS) files were generated from reads that passed at least two times through the insert. The CCS files produced a total of 19,405,930 zero-mode waveguides (ZMWs) and 17,007,966 (87.64%) ZMWs passed all filters (Supplementary Table 1). The demultiplexing process was done by the LIMA barcode demultiplexing^20^ to produce 21 BAM files with a total of 16,594,967 QC-passed reads. The demultiplexed reads were refined with Isoseq3 to remove concatemers and polyA tails, resulting in a total of 16,419,731 (98.94%) full length non-concatemers (FLNC) reads (Supplementary Table 2).

Redundant isoforms from SAM files were collapsed with TAMA collapse^9^ using the option exon cascade collapse generating 21 collapsed transcriptomes in BED format. Comparing the number of corrected-trimmed reads with collapsed isoforms showed a clear positive Pearson’s correlation of R = 0.81. Saranac-SS-Stem had a relatively low number of corrected-trimmed reads (456,574) but produced a high number of collapsed isoforms (49,646). Saranac-CK-Leaf had a high number of corrected-trimmed reads (1,061,656) but produced a similar number of collapsed isoforms (49,687) to Saranac-SS-Stem (Supplementary Figure 1).

### Characterization of alfalfa isoforms

**Figure 1:**
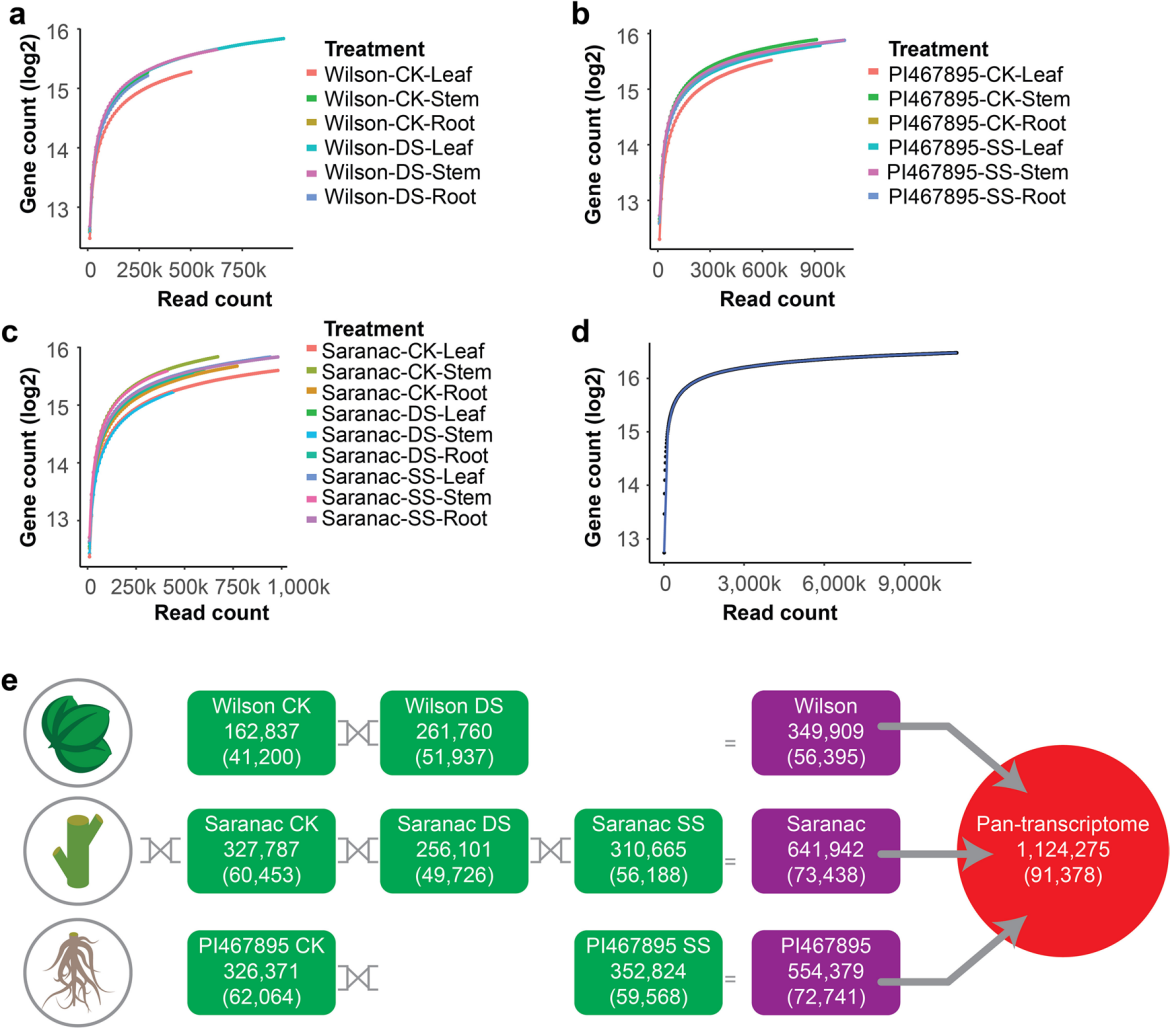
Analysis of the *Medicago sativa* pan-transcriptome. Gene saturation curves in (**a**) Wilson, (**b**) PI467895, (**c**) Saranac transcriptomes and (**d**) pan-transcriptome. **e** Non-redundant isoforms and genes in Wilson, Saranac and PI467895 by control (CK), drought stress (DS) and salt stress (SS) (green boxes). Different treatments and genotypes were used to obtain non-redundant isoforms and genes (purple boxes). Finally, they were merged to obtain the pan-transcriptome (red circle).

To evaluate the quality of the libraries and to confirm the number of detected genes in the pan-transcriptome, TAMA-GO was used to produce a saturation curve for gene discovery by Iso-Seq using read support levels for each gene/transcript collected during the clustering and merging process. Saturation curves in 21 transcriptomes intend to capture all gene repertoires and the curves became a plateau when the number of reads reached > 300,000 for most of libraries. (Fig. 1a–c). Some libraries such as Wilson-DS-Root, Wilson-CK-Stem or Wilson-CK-Root showed lower read counts, associated with low number of FLNC (Supplementary Table 2) which caused the count curve stopped before reaching plateau. When all libraries were merged, the saturation curve occurred after 3 million reads (Fig. 1d).

To unify the gene and isoform IDs among transcriptomes, all 21 BED files were merged using the TAMA merge tool^9^. A pan-transcriptome with 1,124,275 non-redundant isoforms and 91,378 non-redundant genes was obtained. To discover the individual contribution of each treatment to the pan-transcriptome, non-redundant isoforms and genes were identified in Wilson-DS, Wilson-CK, PI467895-SS, PI467895-CK, Saranac-CK, Saranac-DS and Saranac-SS. Wilson-CK has the lowest number of isoforms and genes (162,837 and 41,200, respectively) and PI467895-SS has the highest number of isoforms (352,824) and PI467895-CK has the highest number of genes (62,064) (Fig. 1e). Non-redundant isoforms and genes were identified by germplasm where Saranac had a higher number of isoforms and genes (641,942 and 73,438 respectively), while Wilson had lowest number of isoforms and genes (349,909 and 56,395 respectively).

Core and tissue-specific transcriptomes were obtained from PI467895, Saranac and Wilson identifying shared isoforms or genes by the intersection (∩) of leaf, stem and root by germplasm and conditions (Supplementary Table 3). PI467895 was the germplasm with more core isoforms and genes (14,860 and 17,018, respectively) while Wilson was the germplasm with lowest number of isoforms and genes (9,140 and 11,597, respectively). Isoforms and genes from same germplasm and tissue were compared by treatments (Supplementary Table 4). Stress-specific isoforms were higher compared with control-specific isoforms. For example, in Wilson > 50% of isoforms were unique in drought stress in all tissue sources. Conversely, core genes were higher than stress or control genes. In Wilson > 48% of genes were core between control and drought stress (DS ∩ CK).

### Transcriptome annotation and nonsense-mediated mRNA decay

**Figure 2:**
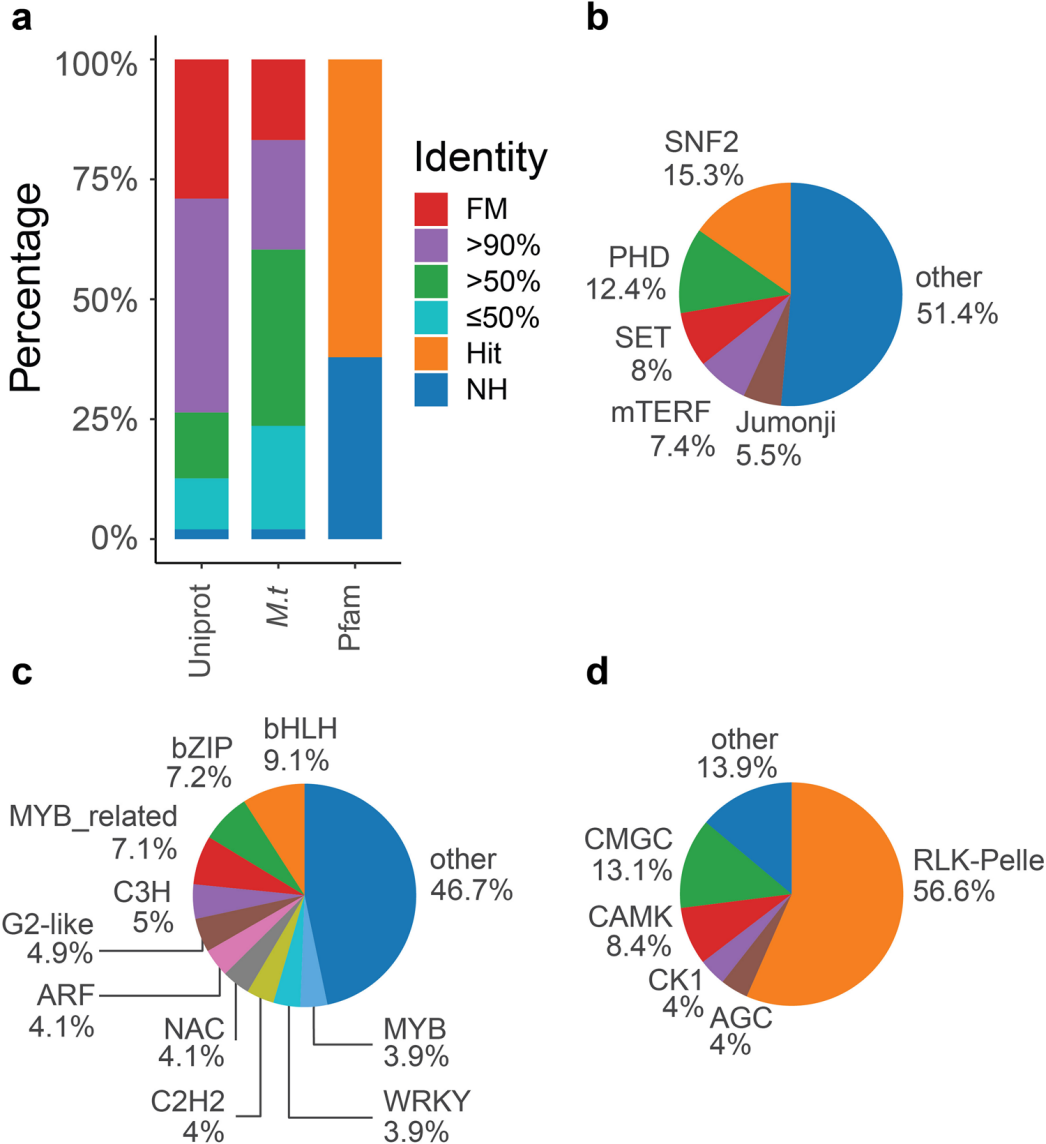
Transcriptome annotation in different databases. (**a**) Percentage of isoforms annotated by Uniprot100, *Medicago truncatula* proteins and PfamA databases in full match (FM), > 90%, > 50% or < 50% BLASTP was used to identity hit or non-hit (NH). (**b–d**) Pie chart with the most relevant families of (**b**) transcriptional regulators, (**c**) transcription factors and (**d**) protein kinases in alfalfa pan-transcriptome.

Isoforms were annotated against Uniprot100^21^ *Medicago truncatula*^22^, iTAK^23^, PlantRegMap^24^, and pfamA^25^ databases (Fig. 2). Uniprot100 and *M. truncatula* protein databases retrieve 827,313 and 445,462 isoforms annotated with > 90% of amino acid identity, respectively. 697,742 isoforms were annotated by pfamA and domain information was used to retrieve GO terms. 17,084 isoforms were annotated as transcription regulators (TR) and most common families were SNF2 (2,613), PHD (2,123), SET (1,368) and mTERF (1,258) (Fig. 2b, Supplementary Table 5). 47,955 isoforms were annotated as transcription factors (TF) and most common families were bHLH (4,375), bZIP (3,467) and MYB-related (3,424) (Fig. 2c, Supplementary Table 5). 43,006 isoforms were annotated as protein kinases (PK) and most common families were RLK-Pelle (24,352), CMGC (5,614) and CAMK (3,610) (Fig. 2d, Supplementary Table 5).

All 21 transcriptomes showed similar numbers of TF, TR, or PK (Supplementary Table 6). The most frequent PK and TF were RLK-Pelle and bHLH, respectively and the most frequent TR was SNF2 in all treatments, except in Saranac-DS-Stem (PHD). Isoforms annotated as TF, TR and PK were compared in control vs stressed conditions from same germplasm and same tissue source. The isoforms only present in stressed conditions were considered upregulated, and isoforms only present in control conditions were considered downregulated (Supplementary Table 7). For example, isoforms annotated as RLK-Pelle shows differences in up and downregulated isoforms among treatments. PI467895-SS-Leaf and PI467895-SS-Root had more RLK-Pelle isoforms upregulated while PI467895-SS-Stem had more RLK-Pelle isoforms downregulated.

TAMA GO: ORF and NMD predictions tool^9^ was used to predict NMD and their exon distribution. NMD was predicted in 989,464 (64.2%) isoforms and it was most frequent in the first exons. The percentage of transcripts without predicted NMD were similar among treatments, with a total of 550,985 (35.8%) transcripts. The highest percentage of NMD was in Saranac-CK-Leaf (69.59%) and Wilson-CK-Stem (68.86%) while the lowest percentage was in Wilson-DS-Stem (66.44%) (Supplementary Table 8). Since NMD affects gene expression levels, we correlated NMD with expression levels in ${log}_{2}TPM$ obtained from RNA-seq data. We were able to identify transcripts without NMD having higher expression mean values compared with transcripts with NMD (Supplementary Figure 2). The ${log}_{2}TPM$ values were lower with NMD in isoforms with more than 10 exons. Among them, Wilson-CK-Root had the highest ${log}_{2}TPM$ values with a maximum value of 1.00 in isoforms without NMD.

### SQANTI3 isoforms classification

**Figure 3:**
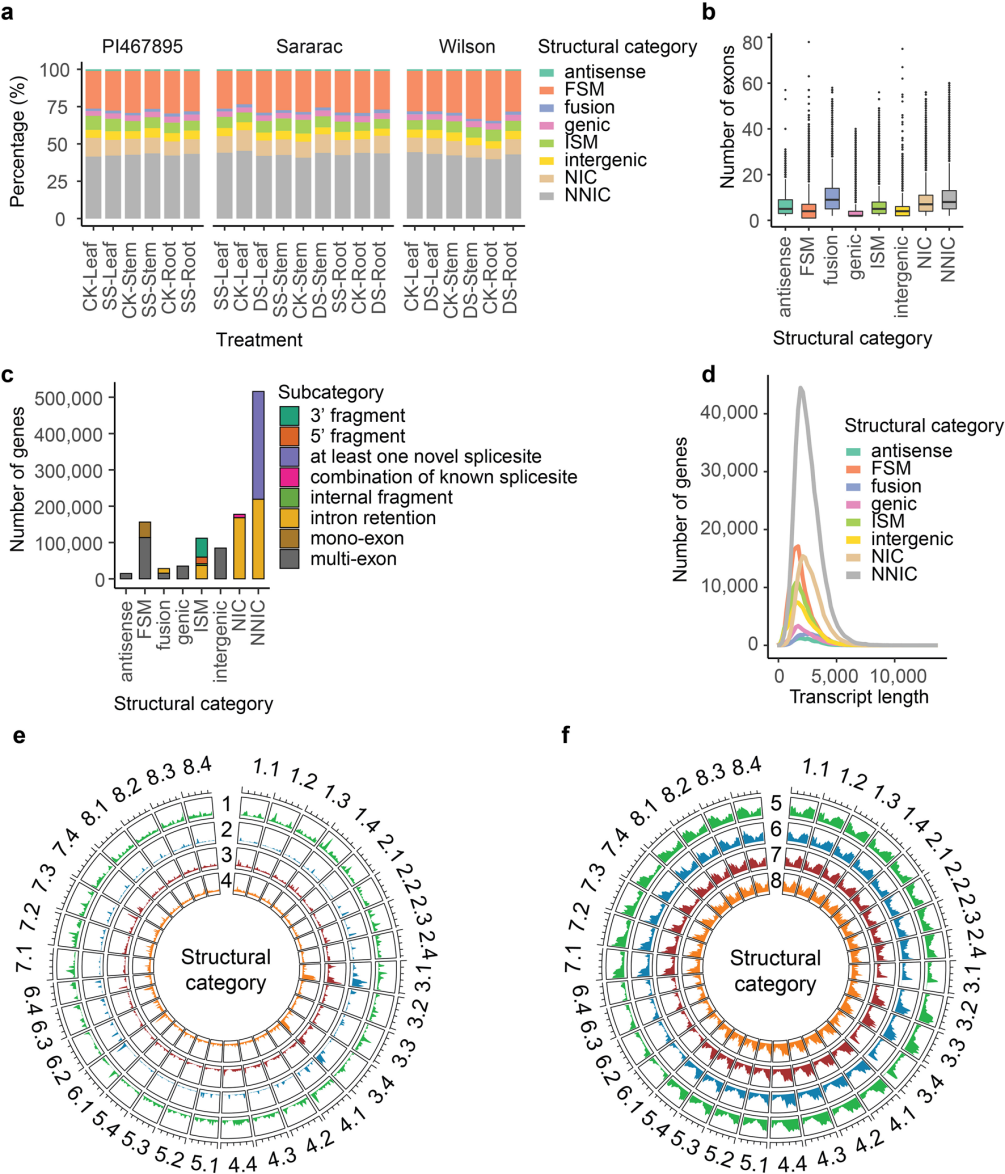
Structural categories of transcriptome classified by SQANTI3. (**a**) Percentage of structural category classification in 21 transcriptomes: germplasms PI467895, Saranac and Wilson in three tissue sources; leaf, stem and root; under salt stress (SS), drought stress (DS) or control non-stressed treatments (CK). (**b**) Boxplot of structural category classification by exon number. (**c**) Structural category frequency by transcript length in base pairs. (**d**) Gene count classified by structural category and subcategory classification. (**e**) Structural category distribution along *Medicago sativa* chromosomes for (1) fusion, (2) antisense, (3) genic, and (4) intergenic transcripts. (**f**) Structural category distribution along *M. sativa* chromosomes for (5) FSM, (6) ISM, (7) NIC and (8) NNIC transcripts. Structural category in SQANTI3 classified transcripts in antisense, full-splice match (FSM), fusion, genic, incomplete-splice match (ISM), intergenic, novel in catalog (NIC) or novel not in catalog (NNIC) transcripts.

SQANTI3 structural classification allowed us to classify isoforms into eight groups: full splice match (FSM), incomplete splice match (ISM), novel in catalog (NIC), novel not in catalog (NNIC), antisense, fusion, genic and intergenic. The most common categories in all treatments were NNIC (mean 42.87%) followed by FSM (mean 26.96%) and the most uncommon categories were Fusion (mean 1.94%) and Antisense (mean 1.12%) (Fig. 3a). Structural classifications were compared by exon distribution and found that genic and antisense groups had the lowest exon number compared with other groups (Fig. 3b). The transcripts were subclassified into subcategories of 5’ fragment (18,433), 3’ fragment (52,388), internal fragment (4,735) and intron retention (IR) (36,684). IR was present in fusion, ISM NIC and NNIC structural classifications. All antisense, genic, and intergenic transcripts were classified into the multi-exon subcategory (Fig. 3c). We did not identify differences in length distribution according to structural classification (Fig. 3d). Structural classification distribution was plotted against the alfalfa allele aware genome finding high number of antisense, genic, and intergenic transcripts at the end of chromosomes 3.1 and 3.4 and lowest isoform density on chromosomes 6.1, 6.2, 6.3 and 6.4 (Fig. 3e,f).

### Noncoding RNAs in alfalfa

**Figure 4:**
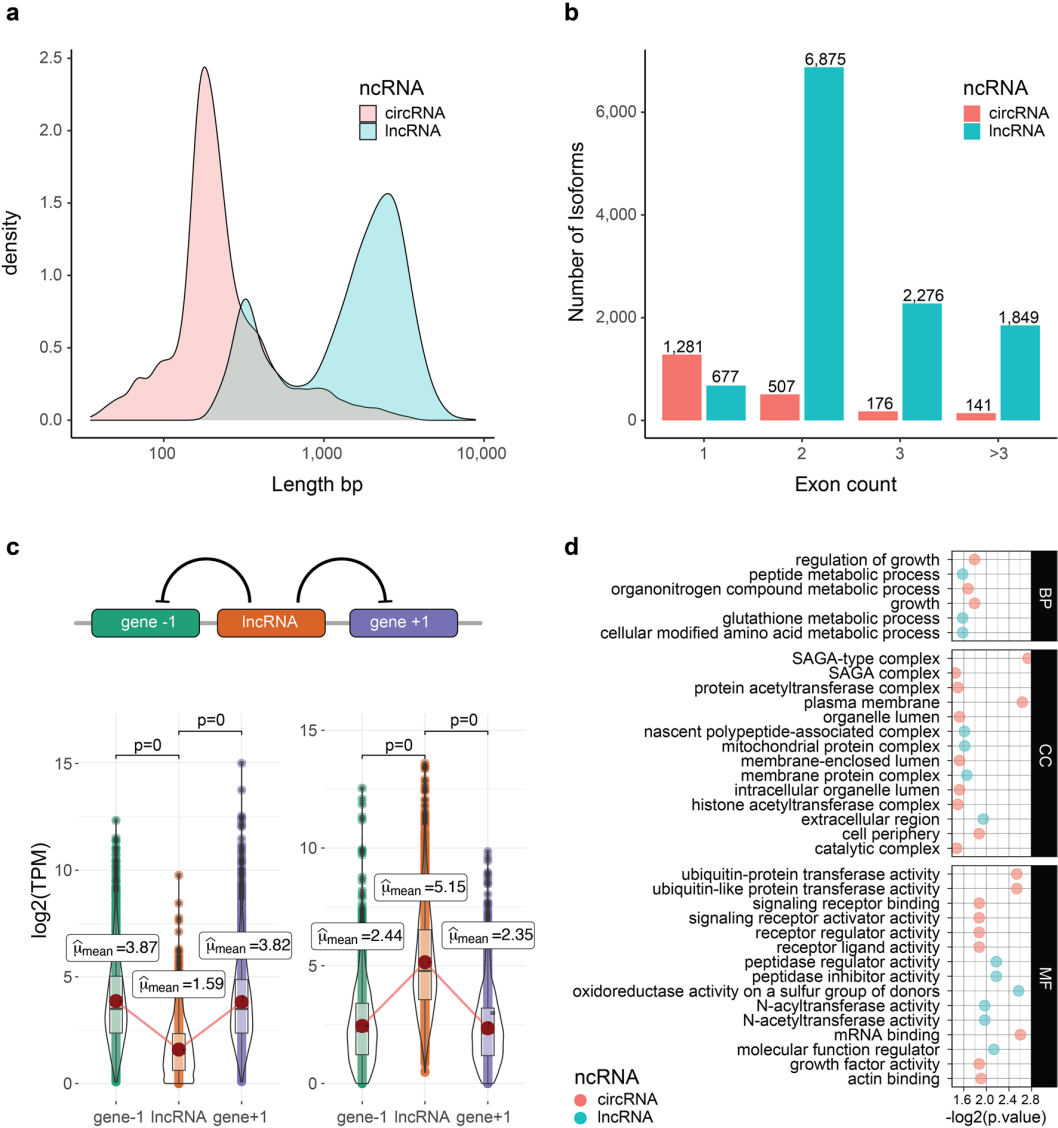
Analysis of noncoding RNAs in *Medicago sativa*. (**a**) Length distribution in bp for lncRNAs and circRNAs. (**b**) Exon count for lncRNAs and circRNAs. (**c**) Up: model for lncRNA regulation in adjacent genes. Down: histogram and violin plot for expression levels in log_2_TPM of gene -1 lncRNA and gene + 1. Only significative comparisons are shown based on p-Bonferroni − corrected. (**d**) Gene ontology enrichment for unique terms in lncRNAs and circRNAs in of biological process (BP), cellular component (CC) and molecular function (MF).

Noncoding RNAs, are group of transcripts involved in post-transcriptional regulation. Long noncoding RNAs (lncRNA) were predicted using the plant long noncoding RNA prediction (PlncPRO)^26^. A total of 11,677 non-redundant lncRNAs were obtained in all 21 Iso-Seq libraries. PI467895-SS-Stem had the highest lncRNAs (1016), while lowest lncRNAs (164) were predicted in Wilson-CK-Root (Supplementary Table 9). The mean length of lncRNAs was 2,081 bp and maximum length was 8,817 bp. Additionally, there was a high frequency of lncRNAs between 200 and 300 bp, possibly due to that 200 bp was the minimum threshold to detect lncRNAs (Fig. 4a). LncRNAs with two and three exons were the most frequent with 6,875 and 2276, respectively (Fig. 4b). LncRNAs were classified according to structural classification using SQANTI3, where the most important categories were identified as follows: intergenic (55.1%), NNIC (16.8%) and ISM (11.3%) (Supplementary Table 10). LncRNAs can regulate the expression of up or downstream genes (Fig. 4c upper part). We compared expression levels of lncRNAs vs up and downstream genes and identified 2,105 lncRNAs differentially upregulated and 1,894 lncRNAs differentially downregulated compared to adjacent genes (Fig. 4c lower part). Gene ontology enrichment analysis of adjacent genes to lncRNAs identified several terms related with drought or salt stress like hydrotropism, oxidoreductase activity, response to auxin, response to stimulus (Fig. 4d, Supplementary Table 11).

CircRNAs were predicted from the RNA-seq data using circexplorer2^27^. Only high confidence circRNAs were analyzed and merged to generate the first circRNA genome-wide profiling in alfalfa. We identified 2,108 non-redundant circRNAs highly specific by treatment, tissue source or germplasm. The highest number of circRNA were found in Saranac-DS-Root with 260 circRNAs, while Wilson-DS-Root had 34 circRNAs (Supplementary Table 9). The mean length of circRNAs was 317 bp and maximum length was 3,613 bp (Fig. 4a). CircRNAs were mainly mono-exonic and the maximum number of exons detected were 18 (Fig. 4b). Gene ontology enrichment analysis of host genes of circRNAs shows how these ncRNAs are targeting to key genes related with drought or salt stress (Fig. 4d). Several parental genes associated with circRNAs were annotated as important regulators: 62 circRNAs were associated with genes annotated as TF, 60 circRNAs were associated with genes annotated as PK. We identified 13 and 22 specific GO terms related to lncRNAs and circRNAs, respectively. We identified common GO terms between lncRNAs and circRNAs involved in auxin response, signal transduction, UDP-glycosyltransferase activity, or catalytic activity (Supplementary Table 11).

### Differential expressed genes and Weighted correlation network analysis

RNA-seq data from leaf, stem and root of the same germplasm and stress treatment were grouped to identify differential expressed genes (DEG). 90 DEG were identified in Saranac (Supplementary Figure 3), 107 DEG were identified in PI467895 (Supplementary Figure 4), and 135 DEG were identified in Wilson (Supplementary Figure 5). Isoform count and annotation are summarized in Supplementary Tables 12 and 13, including six lncRNAs, one circRNA, eight PKs in seven families, 20 TFs in 12 families and 67 uncharacterized isoforms.

**Figure 5:**
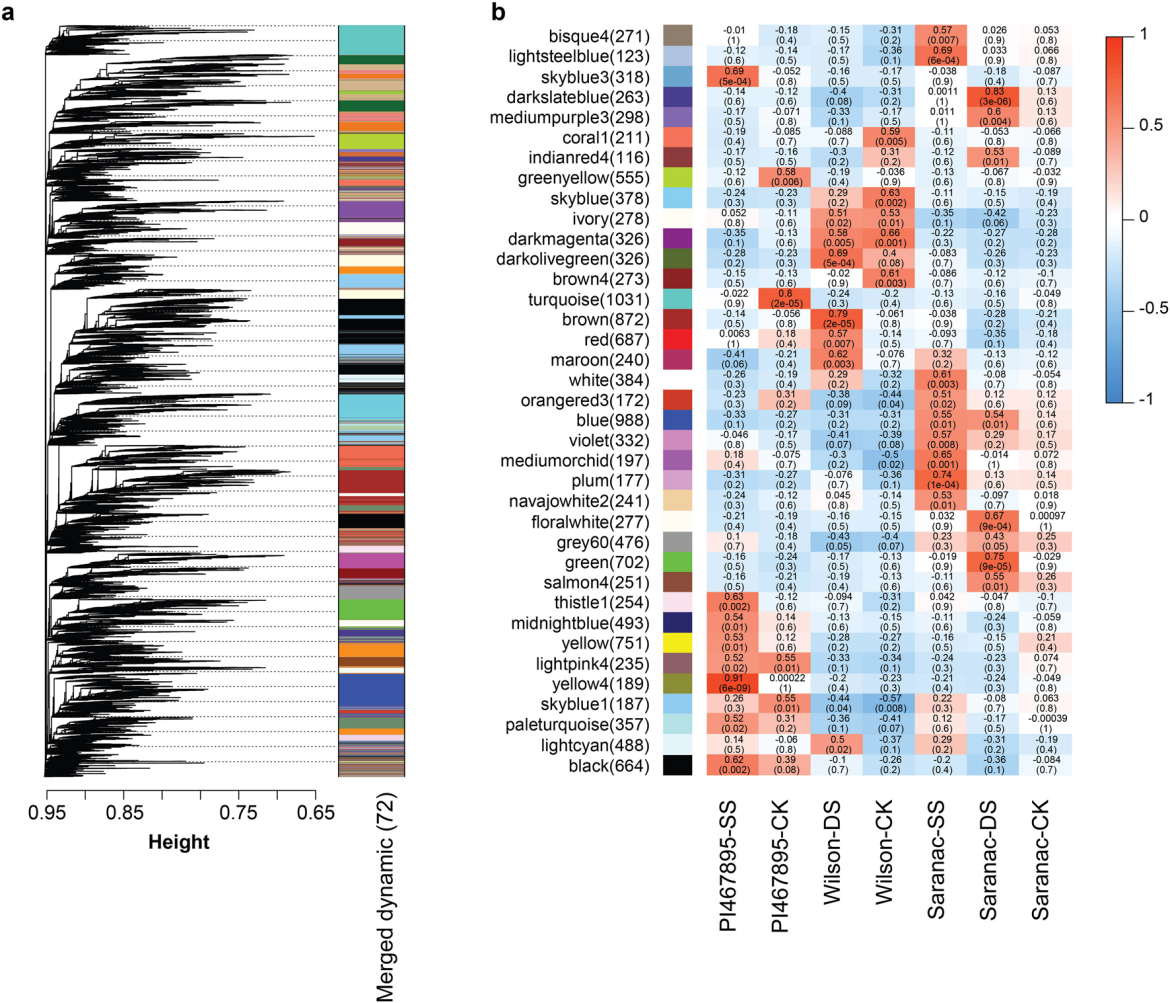
Gene co-expression modules detected by weighted correlation network analysis. (**a**) Clustering dendrogram of isoform expressed in all treatments based on a topological overlap matrix with merged module colors. The color rows present visual comparisons of module assignments based on the merge dynamic method. (**b**) Module-trait association among modules of isoforms by treatments: PI467895-SS, PI467895-CK, Wilson-DS, Wilson-CK, Saranac-SS, Saranac-DS and Saranac-CK. Drought stress (DS), salt stress (SS) and control non-stressed (CK). In each cell the upper values are Pearson correlations from -1 (blue) to 1 (red) and lower values are *p* values of association.

To carry out a weighted correlation of gene network analysis (WGCNA), the WGCNA R package was used^12^. Adjacency matrix was calculated using the soft-thresholding power 8. Hierarchical clustering was used to identify 72 modules using the dynamicTreeCut function (Fig. 5a). Isoforms for each module were analyzed according to alfalfa germplasm and stress conditions. The analysis identified several significant modules–trait associations based on the threshold of Pearson correlation > 0.5 and *p *value < 0.05. PI467895-SS, Wilson-DS, Saranac-SS and Saranac-DS had seven, five, seven, and six significant modules, respectively. Additionally, blue module with 988 isoforms was significantly correlated with both Saranac-SS and Saranac-DS. All significant modules identified by treatment were exported to Cytoscape to generate transcript networks. The PI467895-SS network contained 3,026 isoforms including 28 lncRNA and 86 TF. The Wilson-DS network had 2,613 isoforms including 18 lncRNA and 48 TF. The Saranac-SS network had 2614 isoforms including 29 lncRNA and 78 TF. The Saranac-DS network had 2,895 isoforms including 40 lncRNA and 121 TF. In total 105 and 307 isoforms were identified as lncRNA and TF respectively. A list of TFs and lncRNAs identified by modules and treatment are in Supplementary Table 14.

**Figure 6:**
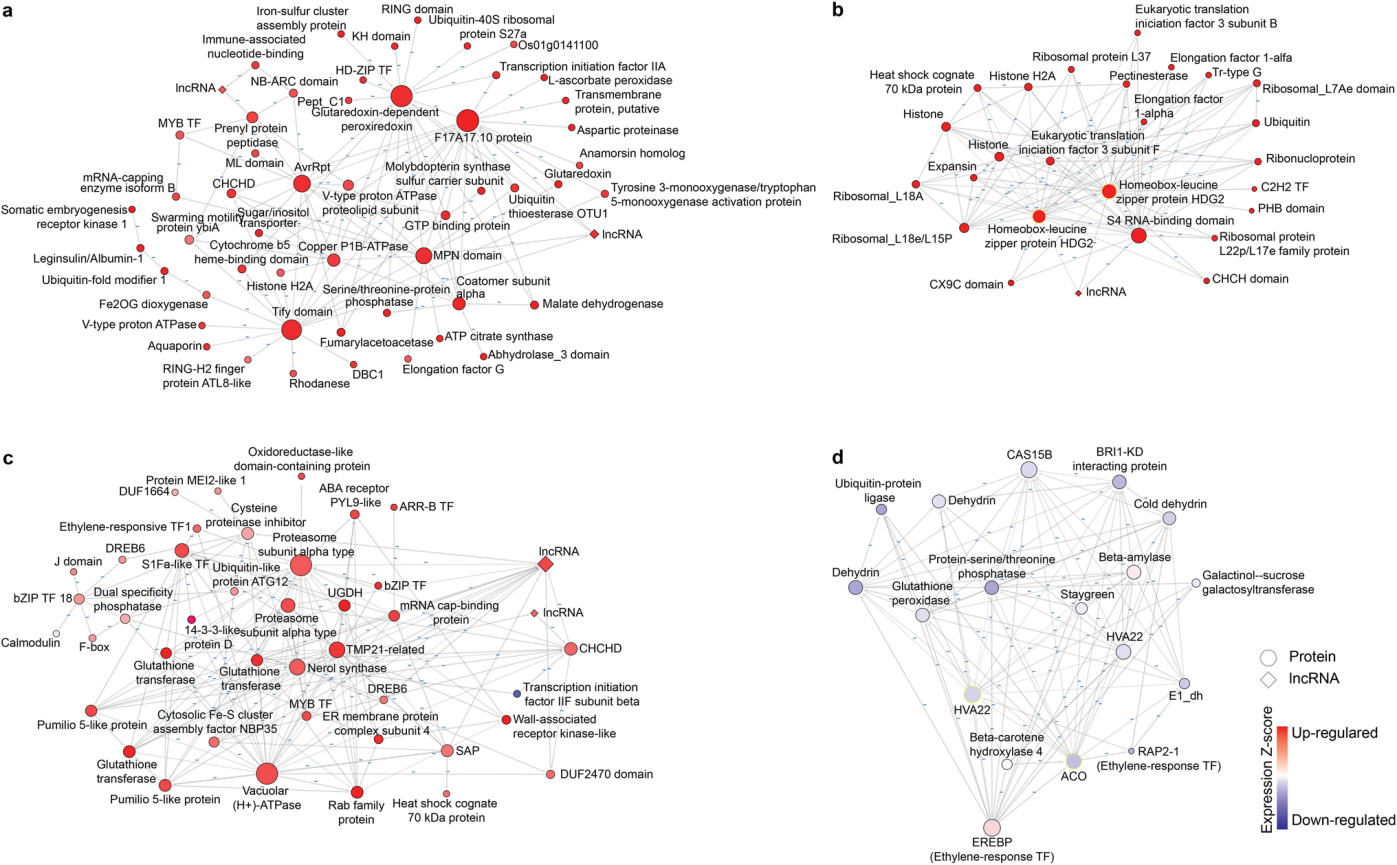
Gene networks in response to salt and drought stresses in tolerant germplasms. (**a**) Sub-network of PI467895-SS. (**b**) Subnetwork of Wilson-DS. (**c**) Sub-network of Saranac-SS. (**d**) Subnetwork of Saranac-DS. Modules of isoforms were exported for weighted correlation network analysis in R package (WGCNA) to obtain Cytoscape keeping hub isoforms, lncRNAs and transcription factors. Red and blue isoforms correspond to upregulated and downregulated isoforms, respectively. Node size is proportional to number of edges (degree), circular shapes correspond isoforms coding proteins and rhombus shapes correspond to lncRNAs, under drought stress (DS), salt stress (SS) and control non-stressed (CK).

A total of 11,148 isoforms in 26 modules were analyzed in the Uniprot database to retrieve Uniprot ID, protein names and metabolic pathways. In total, 279 out of 11,148 isoforms were annotated in some metabolic pathways (Supplementary Table 15). Among metabolic pathways, protein ubiquitination was the most common pathway with 41 genes in 18 modules followed by glycolysis with 36 genes in 11 modules and protein glycosylation with 16 genes in nine modules. To improve characterization of gene regulation networks, PI467895-SS, Wilson-DS, Saranac-SS and Saranac-DS networks were visualized in Cytoscape. All non-connected components, and non-annotated proteins were filtered from networks. The PI467895-SS network was composed of 3,026 isoforms and 2,537 connected components including 50 lncRNA and 94 TF (Supplementary Figure 6). The Wilson network was composed of 2613 isoforms and 2286 connected components including 40 lncRNA and 55 TF (Supplementary Figure 7). The Saranac-SS network was composed of 2,616 isoforms and 2,134 connected components including 25 lncRNA and 82 TF (Supplementary Figure 8). The Saranac-DS network was composed of 2,895 isoforms and 2,553 connected components including 55 lncRNA and 152 TF (Supplementary Figure 9). Finally, to reduce complexity of the networks, subnetworks were constructed independently selecting hub isoforms, lncRNAs and TF (Fig. 6).

## Discussion

### Comparative transcriptomic analysis using PacBio and RNA-seq

In this study, we used both Iso-Seq and RNA-seq platforms and generated genome-scale transcriptomes from 21 samples of three tissue sources (leaf, stem and root) in three germplasms (Saranac, PI467895 and Wilson) under three stress conditions (SS, DS and CK). Iso-Seq allows to obtain full-length transcripts without assembly steps, helping in obtaining completed alternative splicing events (ASE). Isoforms were aligned to allele-aware chromosomes of the autotetraploid alfalfa genome (*M. sativa* cv XinJiangDaYe) which was assembled in all 32 chromosomes (2n = 2n = 4× = 32) and 9,789 scaffolds with a total of 164,632 protein-coding genes^28^. It has been reported that the use of single genotype for transcriptome analysis in response to abiotic stress may not represent the genome-scale diversity of transcripts in a particular species^29^. Therefore, in the present study, we used three germplasms of alfalfa and analyzed 21 transcriptomes to generate a merged pan-transcriptome with 1,124,275 isoforms in 91,378 genes. This approach made a significant improvement in isoforms and gene discovery detecting almost seven times more transcripts than those reported in the reference genome of alfalfa cv XinJiangDaYe^28^.

We identified differences in numbers of isoforms and genes among germplasms, which might be caused by structural variation^30^ or incomplete capture of genes in an individual^19^. For example, PI467895 has more than 5,000 core isoforms in comparison with Wilson and Saranac (Supplementary Table 3). All transcriptomes had a similar average gene length (~ 2,400 bp) with a maximum length of 13,424 bp, which is similar to a previous report^19^. However, in this work we discovery a vast number of new isoforms. Only 27.2% isoforms were classified as FSM; isoforms with the same splicing pattern as predicted in the reference genome. Isoforms belong to NNIC, isoforms with novel splice donors or acceptors represent 42.7%, demonstrating the usefulness of Iso-Seq to discover new isoforms in alfalfa.

It has been reported that NMD is an important process to identify premature termination codons in controlling gene expression^31^. Previous work with NMD in alfalfa focused on heat shock transcription factor 1 (*MsHSF1*) which is related with nodule formation by inoculation with *Sinorhizobium meliloti*^32^. In this work, we were able to predict NMD in 62.4% of isoforms and revealed that this process was a common post-transcriptional modification in alfalfa, which helped in understanding the complexity of gene expression. Additionally, the frequency of NMD and expression mean values decreased as exon predicted NMD increased, thus, higher expression levels occurred in isoforms without NMD than those with NMD. These results agree with the previous Iso-Seq results in maize and sorghum^33^.

### Transcription factors, protein kinases and transcription regulators in alfalfa

Previous studies identified 265 MYB TFs in alfalfa^34^. In the present study, we identified 46,260 transcription factors and nine new TF families (ARR-B, G2-like, HRT-like, LFY, MIKC-MADS, STAT, VOZ, WOX) in alfalfa and added to the alfalfa transcription factor database^35^. Interestingly, VOZ (Vascular Plant One-Zinc-Finger) TFs have been reported as positive regulators of salt tolerance in Arabidopsis and rice^36,37^. In this work we predicted 159 isoforms annotated as VOZ TFs with different expression patterns according to tissue or treatments. Additionally, in this work, we identified 46 isoforms of signal transducer and activator of transcription (STAT) TFs in alfalfa. STATs are very common in non‐plant species, and involved in the activation of the Janus tyrosine kinase (JAK)/STAT pathway^38^. It has been suggested that the GRAS TFs fulfil the function of STAT TFs in plants^39^. However, in this work, we identified both GRAS and STAT TFs which opens the question of whether STAT and GRAS TFs have redundant functions in alfalfa.

RLK-Pelle, CAMK, CK1 and CMGC were the most frequent PK families among all transcriptomes analyzed in this study and this is the first report of genome-wide level of PK in alfalfa. In *M. sativa*, calcium/calmodulin-dependent protein kinase (CAMK) is involved in the rearrangement of intramolecular hydrophobic interactions and in calcium oscillations^40^. In rice, the PK CAMK OsDMI3, confers saline-alkaline tolerance by modulating the Na^+^ and H^+^ influx in root^41^. Here we reported 243 genes and 3,610 isoforms annotated as CAMK, with the higher number of isoforms up and downregulated in Saranac-SS-root.

Transcription regulators (TR) identified in the present study were classified in 23 families and the most abundant families were SNF2 and mitochondrial transcription termination factor (mTERF). SNF2 is involved in positive and negative regulation of gene expression of a large number of genes. mTERF is involved in organellar gene expression. In *Chlamydomonas*, mTERF protein MOC1 is involved in controlling the stromal redox and played roles in the communication between chloroplast and mitochondrion, organelles for redox homeostasis^42^. Additionally, 855 isoforms in 79 genes were annotated as Auxin/Indole-3-Acetic Acid (Aux/IAA) transcriptional regulator family and 1,986 isoforms in 71 genes were annotated as auxin response factor (ARF) transcription factor family which are involved in auxin early response. In *Medicago truncatula* Aux/IAA family is involved in nodule formation during infection with *Sinorhizobium meliloti*^43^. Additionally, we were able to differentiate the low frequent families of TF in different germplasm and tissue sources. For example, 6 genes and 22 isoforms were annotated as HRT-like transcription factor in PI467895-stem. Among those, however, the isoform G77245.10 was only present in PI467895-SS-Stem, while isoform G77245.6 was only present in PI467895-CK-Stem (Supplementary Figure 10).

### The importance of non-coding RNA a in response to drought and salt stresses

NcRNAs play a crucial role in abiotic stress tolerance through five different pathways, including target mimicry, sRNA precursors, DNA methylation, antisense transcription, and histone modification. The mechanism of ncRNAs in response to drought or salt stress may be through abscisic acid (ABA) signaling pathways and modulation of ion channels (Supplementary Table 16). For example, in *M. truncatula*, the expression of 12 lncRNAs involved in osmotic and salt stress have been validated and lncRNA TCONS_00020253 is upregulating Medtr1g081900 a Na+/H+ex-changer (NHX) gene which improves salt tolerance^44^.

In this work, we identified a diversity of ncRNAs which is in agreement with the previous reports^45^. This is the first report of lncRNAs in regulation of plant response to drought and salt stress in *M. sativa*, although previous reports highlighted the importance of lncRNAs in response to osmotic and salt stress and cold stress in *M. truncatula*^11,44^. Additionally, we were able to identify 565 lncRNA with a length between 200 and 300 bp with abnormal distribution. The high frequency of transcripts between 200 and 300 bp could be affected by ncRNAs classification. ncRNAs can be classified as small nucleolar RNAs (snoRNAs) if the size is between 60 and 300 bp or as lncRNA if the size is > 200 bp. The overlap of 200–300 bp may cause a redundancy between snoRNAs and lncRNA. We predicted a lower number of lncRNAs compared with previous studies of *M. truncatula*. Wang et al.^44^ predicted 23,324 lncRNAs under osmotic and salt stress, and Zhao et al.^11^ predicted 24,368 lncRNAs under cold stress. This inconsistency could be due to the genomic differences between *M. truncatula* and *M. sativa*, or different methodologies used for lncRNA prediction. In the present study, we used plncPRO^26^, a tool designed specifically for predicting lncRNAs in plants based on random forest algorithm, which could be more stringent but more accurate than the method used by Wang et al., where lncRNAs were predicted based on lack of coding potential^44^. Most of lncRNA were intergenic, and many of them showed an inverse expression compared with their adjacent genes. Our result agreed with the role of ncRNAs in transcriptional silencing of adjacent genes.

CircRNAs are important class of ncRNAs with multiple roles including tolerance to drought and salt stress previously reported in cucumber, maize and Arabidopsis^13,46^. Here we predicted 2,108 non-redundant circRNAs in resistant and non-resistant alfalfa genotypes under drought and salt stress. This is the first report of circRNA in response to drought and high salinity in alfalfa. Several circRNAs involve in regulation of growth process, ubiquitin activity and signaling pathways, and participating SAGA (Spt–Ada–Gcn5 Acetyltransferase) transcriptional complex.

### Gene regulation networks

WGCNA has been used to identify correlated genes under low temperature in *M. falcata*^38^ and to identify potential regulators of flower color pigmentation in *M. sativa*^47^. In this work we obtained a dynamic tree with 26 significatively associated modules related to salt or drought stress in PI467895, Wilson or Saranac (Fig. 5). Blue module with 988 isoforms in 922 genes was significatively associated in the non-tolerant genotype Saranac under drought and salt stress.

This result could be relevant in plant breeding, because blue module isoforms co-regulated in drought and salt stress can be used as molecular markers. Yellow4 module, significatively associated with PI467895-SS, had 189 isoforms in 127 genes present at a high correlation (0.91). We identified 341, 210 and 98 isoforms predicted as TF, PK and TR respectively. Blue, green and yellow were the modules with high number of TFs (42, 36, 27). Additionally, we found 170 isoforms predicted as lncRNAs. This finding helped in generating the regulatory network in response to drought and salt stress in alfalfa. The analysis of gene regulatory networks allowed identifying highly connected isoforms (hubs) and the interaction of TF and lncRNA with other genes.

Hierarchical layout algorithm from Cytoscape was used for illustrating the main direction or “flow” within a network arranged by the layer and the order of the isoforms to identify master regulators in each network. Characterization of master genes identified in gene networks in response to drought and salt stresses are showed in Table 1. The PI467895-SS subnetwork was composed by 55 isoforms identifying a glutaredoxin-dependent peroxiredoxin (G63340.1) as an important hub (Fig. 6a). Glutaredoxin-dependent peroxiredoxin has been reported to be involved in salt stress adaptation by redox signaling^48^. Additional reports showed downregulation of glutaredoxin-dependent peroxiredoxin by salt stress in Arabidopsis^49^. In this work we discovered glutaredoxin-dependent peroxiredoxin that was regulated by multiple genes such as ubiquitin and HD-ZIP transcription factors. Also, the TIFY domain protein was identified as another important hub. TIFY domain proteins are TFs involved in growth and plant development. In watermelon, TIFY genes (ClTIFY1 and ClTIFY2) were highly induced by salt stress^50^.

**Table 1:** Master genes identified in alfalfa pan-transcriptome by weighed gene co-expression network analysis.

Isoform	Protein name	Uniprot ID	Reported function	References
G1130.10	Nuclear fusion defective 6 (NFD6), chloroplastic	A0A1S2XCH5	Required for karyogamy during female gametophyte development	^87^
G47352.5	C2H2-type domain-containing protein	C6TJ33	Involved in drought stress response	^51^
G12294.8	ERF TF (RAP2-1-like)	A0A1S3U158	Transcription factor with role in adaptation to drought and salt stress	^88^
G1447.4	Beta-carotene hydroxylase 4	G4Y9K0	Required for biosynthesis of zeaxanthin, a carotenoid precursor of abscisic acid	^89^
G28222.9	HVA22-like protein	A0A498JQ43	Induced by drought and salt stress	^90^
G9711.2	1-aminocyclopropane-1-carboxylate oxidase (ACO)	A0A445LLE1	Involved in ethylene biosynthesis	^61^
G30158.7	MYB TF	A0A4D6MN31	Involved in tolerance to drought and salt stress via an ABA-dependent pathway	^91^
G1134.5	Ethylene-responsive element binding protein (EREBP protein)	D5LMH1	Transcription factor with role in adaptation to drought and salt stress	^88^
G3736.3	Zinc finger A20 and AN1 stress-associated protein 8 (SAP)	A0A371IE91	Involved in abiotic stress tolerance	^59^
G10416.2	Vacuolar (H+)-ATPase G subunit	A0A1R3HQE5	Role in sodium sequestration into the central vacuole in response to salt stress	^92^
G7530.16	Glycolipid transfer protein (GLTP)	C6SYQ5	Role in transfer of sphingoid- and glycerol-based glycolipids	^93^
G5353.7	Homeobox protein HAZ1	A0A371FMI3	Transcriptional repressor involved in the regulation of gibberellin	^94^
G16835.10	Tify TF	A0A445DW05	TF induced by salt stress	^50^
G3495.11	Malate dehydrogenase	F5B9G0	Increase salt and cold tolerance modifying the redox state and salicylic acid content	^95^
G63340.1	Glutaredoxin-dependent peroxiredoxin	A0A2G9GS61	Involved in salt stress adaptation by redox signaling	^48^
G27169.8	MPN domain-containing protein (JAMM domain)	A0A5B6ZYU7	Domain present metalloenzymes involved in deubiquitination	^96^
G19941.23	Fumarylacetoacetase	A0A444ZCX7	Involved in synthesizes of acetoacetate and fumarate from L-phenylalanine degradation	^97^
G8059.13	Coatomer subunit alpha	Q70I39	Involved in protein transport from the ER, via the Golgi up to the trans Golgi network	^98^

Wilson-DS subnetwork was composted by 26 isoforms (Fig. 6b). Among them, Isoform G47352.5, a C2H2-type domain-containing protein, was annotated as a master regulator of abiotic stress tolerance. In Arabidopsis the C2H2 family involved in drought stress response^51^. Proteins of this family, Csa1G085390 and Csa7G071440 were upregulated in response to salt and drought stresses in *Cucumis sativus*^52^. Isoform G31078.4 1 was annotated as an expansin, which was involved in cell wall extension and cell enlargement and is induced in drought in tobacco^53^. Whereas in the Wilson-DS network, the high number of ribosomal, histone and uncharacterized proteins caused difficulty in the assumptions about the network. Nevertheless, we identified two isoforms as central hubs. One was annotated as homeobox-leucine zipper protein (HD-ZIP) HDG2 and another was annotated as S4-RNA binding domain protein. They were interacted with other genes in the Wilson-DS network. Previous reports suggested that HD-ZIP ATHB-12, increased salt tolerance by regulating Na^+^ ion homeostasis in yeast^54^ while the *MtBH2*, a HD-ZIP II of *M. truncatula* was negatively regulated in response to abiotic stress in Arabidopsis^55^.

In Saranac-SS network (Fig. 6c) isoform G10416.2 (vacuolar (H^+^)-ATPase) was a central hub. Previous report suggested its importance in response to salt stress^56^. Isoform G33123.7 (nerol synthase) is a terpene synthase involved in nerol production, an acyclic monoterpene alcohol. The role of terpene synthases in *M. truncatula* is related with response to insect herbivory^57^; however, it has been reported that 20 terpene synthases were suppressed by salt stress in *Camellia sinensis*^58^. In this study, we identified isoform G17260.6, annotated as a MYB transcription factor, regulating 15 isoforms including nerol synthase and lncRNA-G55925.1. Isoforms G16125.2 and G3736.3 were annotated as zinc finger A20 and AN1 domain-containing stress-associated protein 8 which are involved in salt stress tolerance^59^. Two isoforms, G30392.2 and G30392.4, were annotated as dehydration-responsive element-binding protein (DREB6), which was involved in drought and salt tolerances in soybean^60^.

The Saranac-DS subnetwork (Fig. 6d) had a high number of isoforms that were downregulated by drought. Isoform G9711.2, annotated as 1-aminocyclopropane-1-carboxylate oxidase-like 4 (ACO) was a hub node. ACO is a key enzyme involved in ethylene production, which plays essential roles in mediating plant responses to biotic and abiotic stresses^61^. Isoform G28222.9 annotated as HVA22-like protein that was induced by abscisic acid with responses to ABA, drought, cold, and salt stresses^62^. Additionally, isoform G1134.5 annotated as EREBP protein (Ethylene-responsive transcription factor) regulated 33 isoforms including isoform ACO and HVA22-like genes. Two isoforms (G63271.4 and G64916.4) were annotated as dehydrin proteins. Dehydrin proteins have roles as protectors of membrane integrity in plant cells under different abiotic stresses including drought stress. In alfalfa, dehydrins has been related with freezing tolerance^63^.

### Similarities and differences between drought and high salinity responses

Although drought and high salinity are different abiotic factors affecting alfalfa production, it has been reported that they share some physiological responses during early stages^64^. Drought and high salinity trigger the production and accumulation of ABA, which in turn induce many stress response genes. Several transcription factors such as MYC/MYB or nuclear factor-Y (NF-Y) are known to regulate the ABA-responsive gene expression. Both drought and salt stresses can damage cellular components and cause metabolic dysfunction, nutrient imbalance, and oxidative stress.

In alfalfa it has been reported that *MsMYB2L* was induced by multiple abiotic stress factors including drought and salt^65^ and *MsMYB4* increase the salinity tolerance ability in an ABA dependent manner 15. In the current work we identified 1,850 isoforms in 274 genes annotated as MYB TF. There is an increase in isoforms annotated as MYB TF in PI467895 and Wilson in stressed treatments (Supplementary Table 6). We found that isoforms G64834.42 and G69600.7 were upregulated by drought and salt stress in Wilson and PI467895, respectively (Supplementary Table 13) and isoform G80387.8 was identified as a master gene in PI467895-SS subnetwork (Fig. 6a).

Dehydrins play important roles in drought tolerance, as protective proteins. For example, dehydrin *MtCAS31* from *M. truncatula* is a positive regulator of drought response^64^. In our work we identified 30 isoforms annotated as dehydrins. Dehydrin isoforms distribution show that most of isoforms were detected in Saranac-DS-Leaf with 17 isoforms and isoforms were not detected in any tissue of Saranac-SS or Saranac-CK in stem and leaf (Supplementary Figure 11). Additionally, isoforms G63271.4 and G64916.4 were present as key regulators in Saranac-DS subnetwork.

Plants use a calcium-dependent protein kinase pathway known as the SOS pathway for salt stress signaling and Na^+^ tolerance. SOS1 is a Na^+^/H^+^ antiporter that can extrude Na^+^ into the soil solution and load Na^+^ into the xylem for long-distance transport to leaves^66^. We identified 97 isoforms annotated as SOS1. The highest number of SOS1 isoforms were identified in salt tolerant germplasm PI467895-SS-Root and PI467895-CK-Root with 22 and 23 isoforms, respectively, while in salt susceptible variety Saranac-SS-Root and Saranac-CK-Root there were just 12 and 8 isoforms, respectively (Supplementary Figure 12). A high isoform diversity of SOS1 in roots of salt tolerant alfalfa suggest an active of role of Na + detoxification mechanism and they agree with previous reports^19^.

### Conclusion and remark

Our study described transcriptomes at the whole genome-scale in three alfalfa germplasms with contrasting tolerance to drought and high salinity. The use of the autotetraploid alfalfa genome as a reference allowed us to identify extensive isoform heterogeneity and differences in allele distributions. The complexity of alfalfa transcriptome has been underestimated and RNA-seq do not show all isoform complexity, therefore the results presented in this work illuminates the isoform diversity in polyploids crop. We used Iso-Seq to capture rare/unstable transcripts in each condition without need of transcripts reconstruction based on short read sequences and predictive algorithms. The use of combination of Iso-Seq to generate the full-length transcripts and RNA-seq to correct the transcript sequences allowed us to identify full length DEGs and isoforms in alfalfa with high accuracy.

The pan-transcriptome atlas revealed specific expressed isoforms in leaves, stems and roots by salt and drought stress treatments in tolerant and non-tolerant alfalfa germplasm. Additionally, this is the first report of circRNAs identified in alfalfa with different profiles depending on tissue sources, genotypes and stress treatments. Finally, gene regulation networks allowed us to identify key candidate genes involved in regulation of plant response to salt and drought stresses, including 170 predicted lncRNAs, 341 transcription factors and several non-annotated genes. This work provides new insights into the mechanisms by which drought and salt stress affect transcriptomes at the whole genome scale. The master regulatory genes can be used for developing molecular markers to improve tolerance in alfalfa to drought and salt stress.

## Materials and methods

### Plant material and growth conditions

Two alfalfa varieties, Wilson (drought tolerant) and Saranac (drought susceptible)^67^, were used for the drought stress experiment. Plants were clonally propagated by stem cuttings which were transplanted into 5 cm diameter pots filled with sand and grown for eight weeks in a greenhouse with a 14 h light period and a temperature of 25 °C. They were irrigated daily with Hoagland’s solution^68^. Six clones of each germplasm were divided into two treatments, three control plants and three receiving drought stress. For the drought stress group, water was withheld for three days until visible signs of drought stress (leaf wilting) appeared in the susceptible variety Saranac, but not in the resistant variety Wilson. Control plants were watered daily. After 3 days of drought stress, roots, stems, and leaves of drought stressed and control plants were collected and frozen in liquid nitrogen until used for RNA extraction. For salinity stress, a salt tolerant accession (PI467895) and the same susceptible variety (Saranac) were used. Plants were grouped in the same way as for drought stress. The control group was irrigated daily with Hoagland’s solution and the salinity stress group was irrigated with Hoagland’s solution containing 100 mM NaCl. After seven days of salinity stress, roots, stems and leaves were collected and frozen in liquid nitrogen until RNA was extracted.

### RNA extraction, Iso-Seq and RNA-seq library preparation

Total RNA was extracted from leaves, stems and roots using RapidPure RNA plant kit (MP Biomedicals, Irvine, CA, USA) according to the manufacturer’s protocol. RNA samples were quantified using Qubit 2.0 fluorometer (Life Technologies, Carlsbad, CA, USA) to ensure a concentration ≥ 50 ng/µl. In addition, the quality of RNA was measured with Nanodrop 1000 (Thermo Sientific, Hanover Park, IL, USA) spectrophotometer to ensure the ratio of A260/A280 was between 1.8 and 2. Iso-Seq libraries were prepared and sequenced at Woodbury Genome Center, Cold Spring Harbor Laboratory (Cold Spring Harbor, NY, USA) following the isoform sequencing protocol PN 101-763-800 Version 02 described by Pacific Biosciences^69^. An input of ≥ 300 ng of RNA with RNA integrity number (RIN) ≥ 7.0 by sample was used to synthesize the first strand cDNA using NEBNext Single Cell/Low Input cDNA Synthesis kit. cDNA was amplified using the amplification module and Iso-Seq Express Oligo Kit including 12 µM of 16-mer barcoded primers. Amplified cDNA was purified using a standard workflow and three samples in equal molar quantities were pooled for each SMRTbell. 21 libraries were sequenced in a total of seven SMRTbells using a PacBio Sequel II system and a P6-C4 chemistry.

Same samples used for Iso-Seq were used for RNA-seq. Sample quality control was checked to ensure RIN ≥ 7.0 and a concentration of ≥ 50 ng/µl of RNA. Libraries were prepared using TruSeq RNA Library Prep Kit v2 and they were sequenced using the Illumina HiSeq sequencing platform at GENEWIZ (South Plainfield, NJ, USA). Sequencing configuration was 2 × 150 bp, single index per lane. Twenty-one flow cells were used, and each cell contained a single type of tissue source from a germplasm with a specific treatment.

### Generation of FLNC

The PacBio subreads obtained from a single movie in PacBio format were classified into circular consensus sequences (CCS) with ccs command from IsoSeq Version 3.4.0 pipeline^70^ to produce seven CCS.BAM files. The seven CCS.BAM files were demultiplexed using lima command from IsoSeq Version 3.4.0^70^ with default parameters (lima-isoseq-dump-clips-peek-guess), resulting in 21 BAM files corresponding to tissue source-germplasm-treatment specific transcriptomes. Demultiplexed transcriptomes were refined with the function isoseq3 refine (− -require-polya) of IsoSeq Version 3.4.0 to remove polyA tails and to detect full length non-concatemer (FLNC) reads.

### Hybrid error correction and mapping

The FLNC files were converted into FASTA format with SAMtools Version 1.10^71^. The Iso-Seq data were corrected with an hybrid error correction approach using RNA-Seq short sequence reads with the program LoRDEC Version 0.6^72^ with the following parameters: k-mer size of 21 and abundance threshold of 2. The corrected and polished reads were aligned to the *M. sativa* allele aware reference genome^28^ using the alignment program Minimap2 Version 2.17^73^ with the following parameters: -ax splice which assumes that the read orientation of the transcript strand is unknown, outputs without secondary alignments and present options configured to splice HQ (-C5 -O6,24 -B4). Finally, the aligned BAM files were sorted to subsequent isoform characterization.

### Isoform characterization

TAMA collapse was used to collapse isoforms using following parameters: collapse common exons ends flags, coverage: 99, identity: 85, 5′ threshold: 10 bp, exon/Splice junction threshold: 10 bp, 3′ threshold: 10 bp, and specifying no capped flag option^9^. After TAMA collapse, 21 BED files were obtained. All BED files were merged to identify unique genes based on tissue source, treatment and germplasm using TAMA merge^9^ with the option of merge duplicate groups to obtain a pan-transcriptome in BED format with unified gene and isoforms IDs.

Pan-transcriptome was annotated and NMD was predicted with the tool TAMA GO: ORF and NMD predictions^9^. The amino acid sequences were aligned against the Uniprot100 protein database^21^ and *M. truncatula*^22^ using diamond BASTP^74^. The results of BLASTP annotation were classified into four groups: (1) open reading frames (ORFs) with complete BLASTP match (FM); (2) ORFs with incomplete BLASTP match categorized in > 90%, > 50% or ≤ 50% of amino acid sequence identity by the BLASTP; (3) ORFs without hits in the protein database (NH); and (4) amino acid sequences without ORF prediction by tama_orf_seeker.py (NO). The BLASTP results were parsed by tama_orf_blastp_parser.py and a new BED file with coding sequences (CDS) regions generated with tama_cds_regions_bed_add.py to exclude stop codon in the CDS region. Transcription factors were predicted using plant transcription factor database PlantRegMap^24^, transcription regulators and protein kinases were predicted using iTAK database^23^ and domain proteins were identified using PfamA database^25^. Finally, all annotations information was combined in the BED file to compare and summarize the results.

The isoforms of collapsed BED files were classified in eight structural categories according to SQANTI3^75^ using the *M. sativa* allele aware reference genome and annotation^28^. Junction files (SJ.out.tab) were obtained from RNA-seq short read data using STAR version 2.7^76^ with minimum intron length set to 20 bp, maximum intron length set to 50 kb and default settings. The SQANTI3 outputs were filtered with sqanti3_RulesFilter.py for intra priming 0.6 and short read junction support to obtain the sqanti_filter.gtf. Isoform densities were plotted by structural categories using circlize Version 0.4.13 R package^77^.

### NcRNAs predictions

Long non-coding RNAs (lncRNAs) were predicted with plncPRO^26^. First, a specific model was built using the mRNA and lncRNA data of *M. truncatula* Mt4.0v1 from Phytozome^78^ and GREENC^79^ databases, respectively using the function of plncpro build. Second, high confidence lncRNAs of the 21 transcriptomes of *M. sativa* were predicted using predict function following parameters: minimum length to define a lncRNA > 200 bp and BLAST database = Uniprot100 DB.

CircRNAs were predicted with Circexplorer2^27^ which identify back-splice junctions and mapped this information of multiple split alignments. The RNA-seq pair-end data was aligned with STAR version 2.7^76^ to obtain the file Chimeric.out.junction. The file Chimeric.out.junction was parsed and circRNAs were annotated with CIRCexplorer2 parse and CIRCexplorer2 annotate respectively.

### Gene ontology

GO enrichment analysis was done with topGO R package Version^80^ 2.44.0. An enrichment test was done with the algorithm “weight01” and Kolmogorov–Smirnov test to obtain a list of the top ten GO enriched terms in BP, MF and CC.

### RNA-seq quantification

Illumina datasets of RNA-seq were evaluated for quality control with FastQC Version 0.11.9^81^ and then quantified with Salmon Version 0.11.3^82^ using as reference pan-transcriptome FASTA sequences. Transcript per million (TPM) mapped reads of each isoform was obtained to generate an expression matrix. Absolute TPM values were imported using with tximport Version 3.13 R package^83^ using the option lengthScaledTPM and TPM were normalized following the script in Bioconductor to create a DEGList for the use in edgeR^84^. DEG were detected among germplasm under different conditions and were plotted using pheatmap Version 1.0. 8 R package^85^. It is important to emphasize that it was necessary to group different tissue sources from the same germplasm and treatment to perform statistical analysis with limma using p-value = 0.05, adjust method of false discovery rate and log2 fold change = 1.5.

### Weighed gene co-expression network analysis (WGCNA)

The WGCNA R package^12^ was used to identify modules of highly differentially expressed genes based on normalized expression data using leaf, stem and root tissue sources as replicates for the same germplasm ~ condition. Function goodSamplesgenes of WGCNA R package was used to remove unqualified genes and pickSoftThreshold function was used to choose an appropriate soft-thresholding power (β) based on a scale-free topology criterion. Adjacency matrix was generated based on the criterion of approximate scale-free topology using the soft thresholding power 8. The adjacency matrix was transformed into Topological Overlap Matrix (TOM) and dissimilarity matrix (1-TOM). Co-expression network of significative modules were exported as .graphml and analyzed using Cytoscape Version 3.8.2^86^. Only interconnected genes were exported in cys and in jpeg formats.

## Supplementary Information

Supplementary Information 1.

Supplementary Information 2.

## Data Availability

Our study complies with relevant institutional, national, and international guidelines and legislation. Permissions were obtained from USDA-ARS Western Regional Plant Introduction Station for collecting and using alfalfa plants for this study. All plant materials used in this study were provided by the USDA-ARS Western Regional Plant Introduction Station and they are public available upon request. The PacBio demultiplexed reads and RNA-seq fastq files were deposited in NCBI Sequence Read Archive (SRA) under accession number PRJNA667169. All 21 individual transcriptomes and pan-transcriptome in BED format, isoform annotation file, metadata information and alfalfa network files in CYS format are available in https://figshare.com/articles/dataset/Sup_transcriptome/13692103.

## References

[1] USDA. Census of Agriculture 2017. USDA-National Agricultural Statistics Service 820. https://www.nass.usda.gov/Publications/AgCensus/2017/index.php#full_report (2017).

[2] Liu Y, Wu Q, Ge G, Han G, Jia Y (2018). Influence of drought stress on afalfa yields and nutritional composition. BMC Plant Biol..

[3] Pessarakli M, Huber JT (1991). Biomass production and protein synthesis by alfalfa under salt stress. J. Plant Nutr..

[4] Rhoads A, Au KF (2015). PacBio sequencing and its applications. Genomics Proteomics Bioinform..

[5] Jain M, Olsen HE, Paten B, Akeson M (2016). The Oxford nanopore MinION: delivery of nanopore sequencing to the genomics community. Genome Biol..

[6] Abdel-Ghany SE (2016). A survey of the sorghum transcriptome using single-molecule long reads. Nat. Commun..

[7] Feng S, Xu M, Liu F, Cui C, Zhou B (2019). Reconstruction of the full-length transcriptome atlas using PacBio Iso-Seq provides insight into the alternative splicing in *Gossypium australe*. BMC Plant Biol..

[8] Minio, A. *et al.* Iso-Seq allows genome-independent transcriptome profiling of grape berry development. *G3 Genes Genomes Genet*. 10.1534/g3.118.201008 (2019). 10.1534/g3.118.201008 PMC6404599 30642874

[9] Kuo RI (2020). Illuminating the dark side of the human transcriptome with long read transcript sequencing. BMC Genomics.

[10] Kapranov P (2007). RNA maps reveal new RNA classes and a possible function for pervasive transcription. Science.

[11] Zhao M (2020). Identification of tissue-specific and cold-responsive lncRNAs in *Medicago truncatula* by high-throughput RNA sequencing. BMC Plant Biol..

[12] Langfelder P, Horvath S (2008). WGCNA: an R package for weighted correlation network analysis. BMC Bioinform..

[13] Zhu YX (2019). Identification of cucumber circular RNAs responsive to salt stress. BMC Plant Biol..

[14] Postnikova OA, Shao J, Nemchinov LG (2013). Analysis of the alfalfa root transcriptome in response to salinity stress. Plant Cell Physiol..

[15] Dong W, Liu X, Li D, Gao T, Song Y (2018). Transcriptional profiling reveals that a MYB transcription factor *MsMYB4* contributes to the salinity stress response of alfalfa. PLoS ONE.

[16] Shu Y (2017). Transcriptome sequencing analysis of alfalfa reveals cbf genes potentially playing important roles in response to freezing stress. Genet. Mol. Biol..

[17] Nemchinov LG, Shao J, Lee MN, Postnikova OA, Samac DA (2017). Resistant and susceptible responses in alfalfa (*Medicago sativa*) to bacterial stem blight caused by *Pseudomonas syringae* pv *syringae*. PLoS ONE.

[18] Duan H-R (2020). Identification of the regulatory networks and hub genes controlling alfalfa floral pigmentation variation using RNA-sequencing analysis. BMC Plant Biol..

[19] Luo D (2019). Full-length transcript sequencing and comparative transcriptomic analysis to evaluate the contribution of osmotic and ionic stress components towards salinity tolerance in the roots of cultivated alfalfa (*Medicago sativa* L.). BMC Plant Biol..

[20] PacificBiosciences. Lima. 1. https://github.com/PacificBiosciences/barcoding (2020).

[21] Bateman A (2019). UniProt: a worldwide hub of protein knowledge. Nucleic Acids Res..

[22] Pecrix Y (2018). Whole-genome landscape of *Medicago truncatula* symbiotic genes. Nat. Plants.

[23] Zheng Y (2016). iTAK: a program for genome-wide prediction and classification of plant transcription factors, transcriptional regulators, and protein kinases. Mol. Plant.

[24] Tian F, Yang D-CC, Meng Y-QQ, Jin J, Gao G (2020). PlantRegMap: charting functional regulatory maps in plants. Nucleic Acids Res..

[25] Finn RD (2014). Pfam: the protein families database. Nucleic Acids Res..

[26] Singh U, Khemka N, Rajkumar MS, Garg R, Jain M (2017). PLncPRO for prediction of long non-coding RNAs (lncRNAs) in plants and its application for discovery of abiotic stress-responsive lncRNAs in rice and chickpea. Nucleic Acids Res..

[27] Zhang X-O (2016). Diverse alternative back-splicing and alternative splicing landscape of circular RNAs. Genome Res..

[28] Chen H (2020). Allele-aware chromosome-level genome assembly and efficient transgene-free genome editing for the autotetraploid cultivated alfalfa. Nat. Commun..

[29] Hirsch CN (2014). Insights into the Maize Pan-Genome and Pan-Transcriptome. Plant Cell.

[30] Zhou P (2017). Exploring structural variation and gene family architecture with De Novo assemblies of 15 *Medicago* genomes. BMC Genomics.

[31] Chang Y-F, Imam JS, Wilkinson MF (2007). The Nonsense-Mediated Decay RNA Surveillance Pathway. Annu. Rev. Biochem..

[32] He G-H (2016). Drought-responsive WRKY transcription factor genes TaWRKY1 and TaWRKY33 from wheat confer drought and/or heat resistance in arabidopsis. BMC Plant Biol..

[33] Wang B (2018). A comparative transcriptional landscape of maize and sorghum obtained by single-molecule sequencing. Genome Res..

[34] Zhou Q (2019). MYB transcription factors in alfalfa (*Medicago sativa*): genome-wide identification and expression analysis under abiotic stresses. PeerJ.

[35] Postnikova OA, Shao J, Nemchinov LG (2014). In silico identification of transcription factors in *Medicago sativa* using available transcriptomic resources. Mol. Genet. Genomics.

[36] Prasad K, Xing D, Reddy A (2018). Vascular plant one-zinc-finger (VOZ) transcription factors are positive regulators of salt tolerance in arabidopsis. Int. J. Mol. Sci..

[37] Ganie SA, Ahammed GJ, Wani SH (2020). Vascular plant one zinc-finger (VOZ) transcription factors: novel regulators of abiotic stress tolerance in rice (*Oryza sativa* L.). Genet. Resour. Crop Evol..

[38] Cui G (2019). Full-length transcriptome sequencing reveals the low-temperature-tolerance mechanism of *Medicago falcata* roots. BMC Plant Biol..

[39] Richards DE, Peng J, Harberd NP (2000). Plant GRAS and metazoan STATs: one family?. BioEssays.

[40] Swainsbury DJK, Zhou L, Oldroyd GED, Bornemann S (2012). Calcium ion binding properties of *Medicago truncatula* calcium/calmodulin-dependent protein kinase. Biochemistry.

[41] Ni L (2020). Calcium/calmodulin-dependent protein kinase *OsDMI3* positively regulates saline-alkaline tolerance in rice roots. Plant Signal. Behav..

[42] Uhmeyer A, Cecchin M, Ballottari M, Wobbe L (2017). Impaired mitochondrial transcription termination disrupts the stromal redox poise in chlamydomonas. Plant Physiol..

[43] Shen C (2014). Genome-wide identification and expression profiling analysis of the Aux/IAA gene family in *Medicago truncatula* during the early phase of *Sinorhizobium meliloti* Infection. PLoS ONE.

[44] Wang T-Z, Liu M, Zhao M-G, Chen R, Zhang W-H (2015). Identification and characterization of long non-coding RNAs involved in osmotic and salt stress in *Medicago truncatula* using genome-wide high-throughput sequencing. BMC Plant Biol..

[45] Li S (2017). Genome-wide identification and functional prediction of cold and/or drought-responsive lncRNAs in cassava. Sci. Rep..

[46] Zhang P (2019). A large-scale circular RNA profiling reveals universal molecular mechanisms responsive to drought stress in maize and Arabidopsis. Plant J..

[47] Du H (2017). Screening and identification of key genes regulating fall dormancy in alfalfa leaves. PLoS ONE.

[48] Finkemeier I (2005). The mitochondrial type II peroxiredoxin F is essential for redox homeostasis and root growth of *Arabidopsis thaliana* under Stress. J. Biol. Chem..

[49] Horling F, König J, Dietz K-J (2002). Type II peroxiredoxin C, a member of the peroxiredoxin family of *Arabidopsis thaliana*: its expression and activity in comparison with other peroxiredoxins. Plant Physiol. Biochem..

[50] Yang Y (2019). Comprehensive analysis of TIFY transcription factors and their expression profiles under jasmonic acid and abiotic stresses in watermelon. Int. J. Genomics.

[51] Sakamoto H, Araki T, Meshi T, Iwabuchi M (2000). Expression of a subset of the arabidopsis Cys2/His2-type zinc-finger protein gene family under water stress. Gene.

[52] Yin J (2020). Genome-wide characterization of the C2H2 zinc-finger genes in *Cucumis sativus* and functional analyses of four CsZFPs in response to stresses. BMC Plant Biol..

[53] Li F (2011). Drought tolerance through over-expression of the expansin gene TaEXPB23 in transgenic tobacco. J. Plant Physiol..

[54] Shin D (2004). Athb-12, a homeobox-leucine zipper domain protein from *Arabidopsis thaliana*, increases salt tolerance in yeast by regulating sodium exclusion. Biochem. Biophys. Res. Commun..

[55] Song S, Chen Y, Zhao M, Zhang W-H (2012). A novel *Medicago truncatula* HD-Zip gene, *MtHB2*, is involved in abiotic stress responses. Environ. Exp. Bot..

[56] Ratajczak R, Richter J, Luttge U (1994). Adaptation of the tonoplast V-type H+-ATPase of *Mesembryanthemum crystallinum* to salt stress, C3-CAM transition and plant age. Plant Cell Environ..

[57] Erb M (2009). Belowground ABA boosts aboveground production of DIMBOA and primes induction of chlorogenic acid in maize. Plant Signal. Behav..

[58] Zhou H-C, Shamala LF, Yi X-K, Yan Z, Wei S (2020). Analysis of terpene synthase family genes in *Camellia sinensis* with an emphasis on abiotic stress conditions. Sci. Rep..

[59] Giri J, Vij S, Dansana PK, Tyagi AK (2011). Rice A20/AN1 zinc-finger containing stress-associated proteins (SAP1/11) and a receptor-like cytoplasmic kinase (OsRLCK253) interact via A20 zinc-finger and confer abiotic stress tolerance in transgenic Arabidopsis plants. New Phytol..

[60] Nguyen QH (2019). Overexpression of the *GmDREB6* gene enhances proline accumulation and salt tolerance in genetically modified soybean plants. Sci. Rep..

[61] Houben M, Van de Poel B (2019). 1-Aminocyclopropane-1-carboxylic acid oxidase (ACO): the enzyme that makes the plant hormone ethylene. Front. Plant Sci..

[62] Shen Q, Uknes SJ, Ho TH (1993). Hormone response complex in a novel abscisic acid and cycloheximide-inducible barley gene. J. Biol. Chem..

[63] Rémus-Borel W (2010). Dehydrin variants associated with superior freezing tolerance in alfalfa (*Medicago sativa* L.). Theor. Appl. Genet..

[64] Munns R (2002). Comparative physiology of salt and water stress. Plant. Cell Environ..

[65] Song Y (2019). The constitutive expression of alfalfa *MsMYB2L* enhances salinity and drought tolerance of *Arabidopsis thaliana*. Plant Physiol. Biochem..

[66] Zhu M (2016). *Nax* loci affect SOS1-like Na+/H+ exchanger expression and activity in wheat. J. Exp. Bot..

[67] Lin S (2020). Identification of genetic loci associated with forage quality in response to water deficit in autotetraploid alfalfa (*Medicago sativa* L.). BMC Plant Biol..

[68] *Hoagland, D. R. & Arnon, D. I. The water-culture method for growing plants without soil. Circular. California Agricultural Experiment Station* vol. 347 (The College of Agriculture University of California Berkeley, 1950).

[69] PacificBiosciences. PN 101-763-800 Version 02. Pacific Bioscence 13. https://www.pacb.com/wp-content/uploads/Procedure-Checklist-Iso-Seq-Express-Template-Preparation-for-Sequel-and-Sequel-II-Systems.pdf (2019).

[70] Gordon SP (2015). Widespread polycistronic transcripts in fungi revealed by single-molecule mRNA sequencing. PLoS ONE.

[71] Li H (2009). The sequence alignment/map format and SAMtools. Bioinformatics.

[72] Salmela L, Rivals E (2014). LoRDEC: accurate and efficient long read error correction. Bioinformatics.

[73] Li H (2018). Minimap2: pairwise alignment for nucleotide sequences. Bioinformatics.

[74] Buchfink B, Xie C, Huson DH (2015). Fast and sensitive protein alignment using DIAMOND. Nat. Methods.

[75] Tardaguila M (2018). SQANTI: extensive characterization of long-read transcript sequences for quality control in full-length transcriptome identification and quantification. Genome Res..

[76] Dobin A (2013). STAR: ultrafast universal RNA-seq aligner. Bioinformatics.

[77] Gu Z, Gu L, Eils R, Schlesner M, Brors B (2014). circlize implements and enhances circular visualization in R. Bioinformatics.

[78] Goodstein DM (2012). Phytozome: a comparative platform for green plant genomics. Nucleic Acids Res..

[79] Paytuví Gallart, A., Hermoso Pulido, A., Anzar Martínezde Lagrán, I., Sanseverino, W. & Aiese Cigliano, R. GREENC: a wiki-based database of plant lncRNAs. *Nucleic Acids Res.* **44**, 1161–1166 (2016). 10.1093/nar/gkv1215 PMC4702861 26578586

[80] Alexa, A. & Rahnenfuhrer, J. *topGO: Enrichment Analysis for Gene Ontology*. R package version 2.42.0. https://bioconductor.org/packages/release/bioc/html/topGO.html (2020).

[81] Andrews, S., Krueger, F., Seconds-Pichon, A., Biggins, F. & Wingett, S. FastQC. A quality control tool for high throughput sequence data. Babraham Bioinformatics. *Babraham Institute*. https://www.bioinformatics.babraham.ac.uk/projects/fastqc/ (2015).

[82] Patro R, Duggal G, Love MI, Irizarry RA, Kingsford C (2017). Salmon provides fast and bias-aware quantification of transcript expression. Nat. Methods.

[83] Soneson C, Love MI, Robinson MD (2016). Differential analyses for RNA-seq: transcript-level estimates improve gene-level inferences. F1000Research.

[84] Robinson MD, McCarthy DJ, Smyth GK (2010). edgeR: a Bioconductor package for differential expression analysis of digital gene expression data. Bioinformatics.

[85] Kolde, R. pheatmap: Pretty heatmaps. https://cran.r-project.org/web/packages/pheatmap/index.html (2015).

[86] Shannon P (2003). Cytoscape: a software environment for integrated models of biomolecular interaction networks. Genome Res..

[87] Portereiko MF (2006). Nuclear fusion defective1 encodes the arabidopsis RPL21M protein and is required for Karyogamy during Female gametophyte development and fertilization. Plant Physiol..

[88] Xie Z, Nolan TM, Jiang H, Yin Y (2019). AP2/ERF transcription factor regulatory networks in hormone and abiotic stress responses in arabidopsis. Front. Plant Sci..

[89] Du H (2010). Characterization of the β-carotene hydroxylase gene *DSM2* conferring drought and oxidative stress resistance by increasing xanthophylls and abscisic acid synthesis in rice. Plant Physiol..

[90] Brands A, Ho THD (2002). Function of a plant stress-induced gene, HVA22 synthetic enhancement screen with its yeast homolog reveals its role in vesicular traffic. Plant Physiol..

[91] Wu J (2019). Expression of the maize MYB transcription factor ZmMYB3R enhances drought and salt stress tolerance in transgenic plants. Plant Physiol. Biochem..

[92] Apse MP (1999). Salt tolerance conferred by overexpression of a vacuolar Na+/H+ Antiport in arabidopsis. Science.

[93] Brown RE, Mattjus P (2007). Glycolipid transfer proteins. Biochim. Biophys. Acta Mol. Cell Biol. Lipids.

[94] Wen BQ, Xing MQ, Zhang H, Dai C, Xue HW (2011). Rice homeobox transcription factor HOX1a positively regulates gibberellin responses by directly suppressing EL1. J. Integr. Plant Biol..

[95] Wang QJ (2016). The enhancement of tolerance to salt and cold stresses by modifying the redox state and salicylic acid content via the cytosolic malate dehydrogenase gene in transgenic apple plants. Plant Biotechnol. J..

[96] Isono E, Nagel M-K (2014). Deubiquitylating enzymes and their emerging role in plant biology. Front. Plant Sci..

[97] Dixon DP, Edwards R (2006). Enzymes of tyrosine catabolism in *Arabidopsis thaliana*. Plant Sci..

[98] Gaudet P, Livstone MS, Lewis SE, Thomas PD (2011). Phylogenetic-based propagation of functional annotations within the gene ontology consortium. Brief. Bioinform..

