# Supplementary material for: Pan-transcriptome identifying master genes and regulation network in response to drought and salt stresses in Alfalfa (Medicago sativa L.)
Source: Sci Rep. 2021 Aug 26;11:17203. doi: 10.1038/s41598-021-96712-x (PMC8390513; doi:10.1038/s41598-021-96712-x)

Pan-Transcriptome Identifying Master Genes and Regulation Network in Response to Drought and Salt Stresses in Alfalfa (*Medicago sativa* L.)

Cesar Augusto Medina[1], Deborah A. Samac[2] and Long-Xi Yu[1*]

[1]United States Department of Agriculture-Agricultural Research Service, Plant Germplasm Introduction and Testing Research, Prosser, WA 99350, USA; cesar.medinaculma@wsu.edu

[2]United States Department of Agriculture-Agricultural Research Service, Plant Science Research Unit, 1991 Upper Buford Circle, 495 Borlaug Hall St. Paul, MN 55108; debby.samac@usda.gov

* Corresponding author: Long-Xi Yu (longxi.yu@usda.gov)

Supporting information

Supplementary Figure 1. Pearson's correlation between corrected-trimmed reads and collapsed gene count by TAMA collapse 21 alfalfa transcriptomes from three germplasms (Saranac, Wilson, PI467895), three tissue sources (Leaf, Stem, Root) and under drought stress (DS), salt stress (SS) or control non-stressed (CK) conditions.

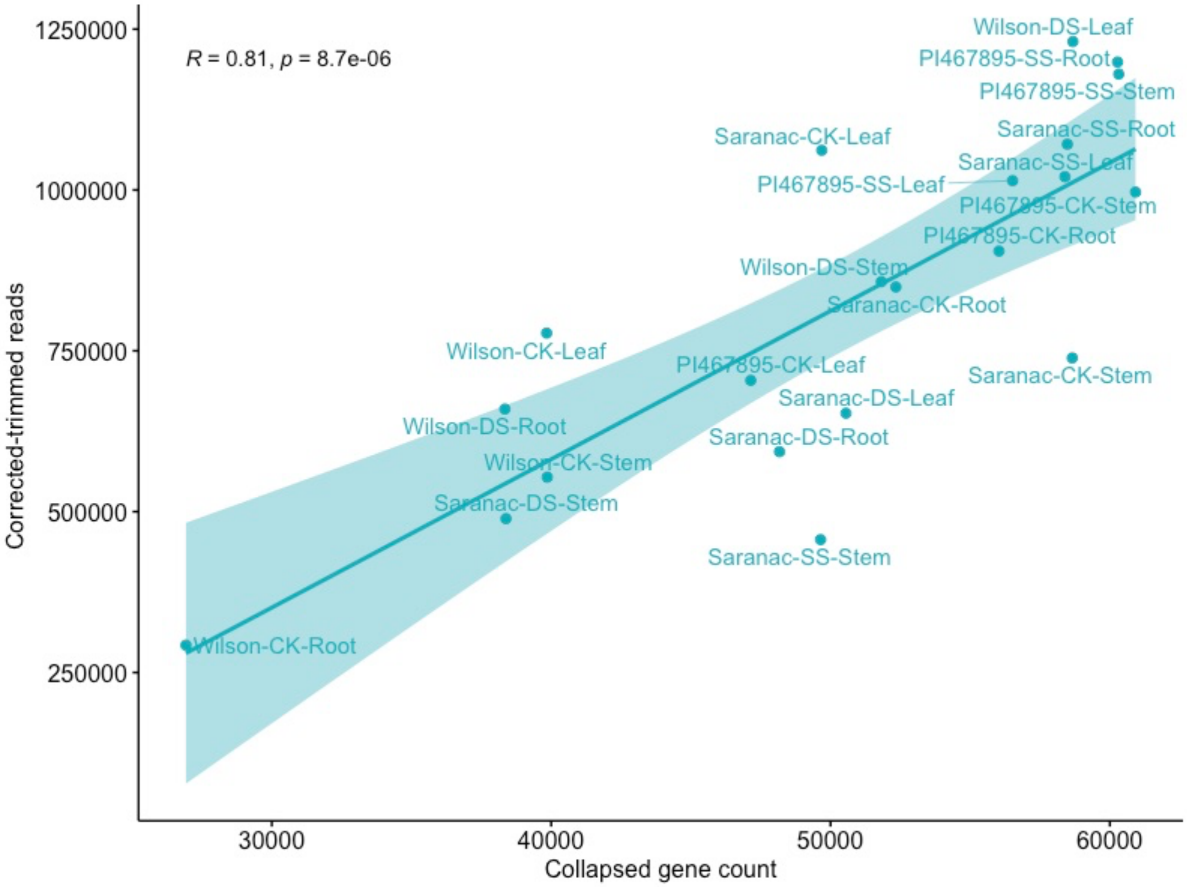

Supplementary Figure 2. Expression levels in log_2 of transcript per million (log_2TPM) by nonsense mediated mRNA decay (NMD) in germplasms Saranac, PI467895 and Wilson under drought stress (DS), salt stress (SS) or control non-stressed (CK) conditions. **A.** Expression levels in accession PI467895. **B.** Expression levels in variety Wilson. **C.** Expression levels in variety Saranac. NMD was classified according to the exon where it was predicted (NMD1−NMD≥10) and transcripts without NMD are reported as prot_ok.

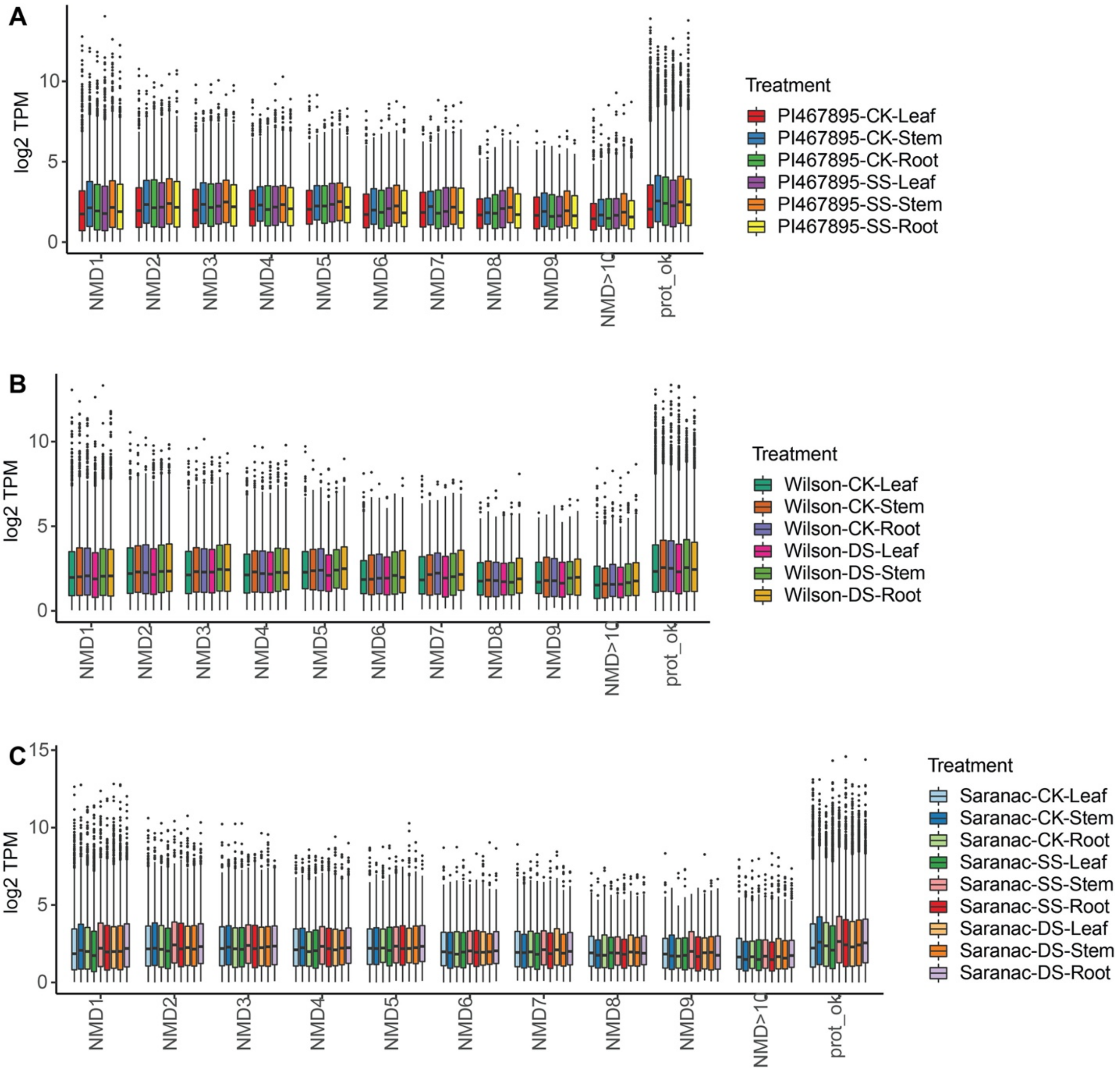

Supplementary Figure 3. Differential expressed genes (DEG) between control (CK), drought stress (DS) and salt stress (SS) in Saranac. DEG were normalized using z-score to compare expression of common genes in leaf stem and root tissues under CK DS and SS. DEG were plotted using pheatmap Version 1.0. 8 R package [84].

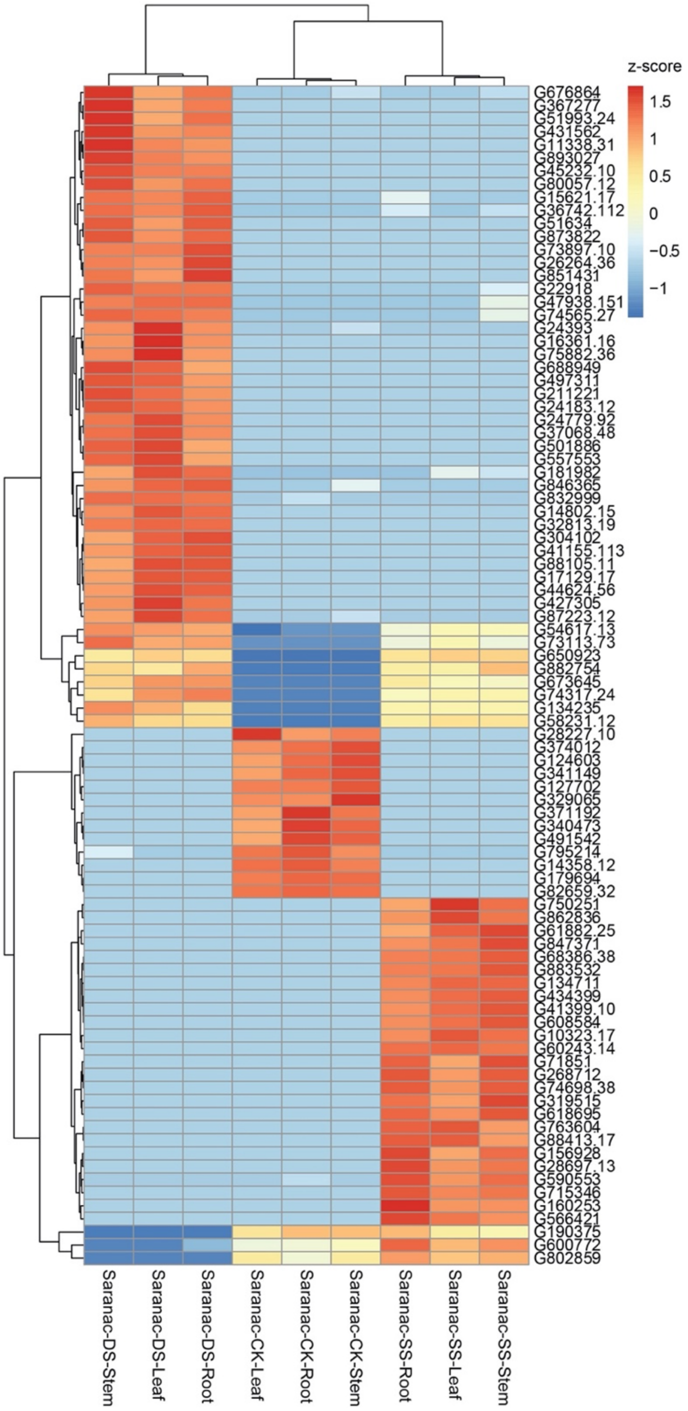

Supplementary Figure 4. Differential expressed genes (DEG) between control (CK) and salt stress (SS) in leaf, stem, and root tissues of PI467895. DEG were normalized using z-score to compare expression of common genes in leaf stem and root tissues under CK and SS. DEG were plotted using pheatmap Version 1.0. 8 R package [84].

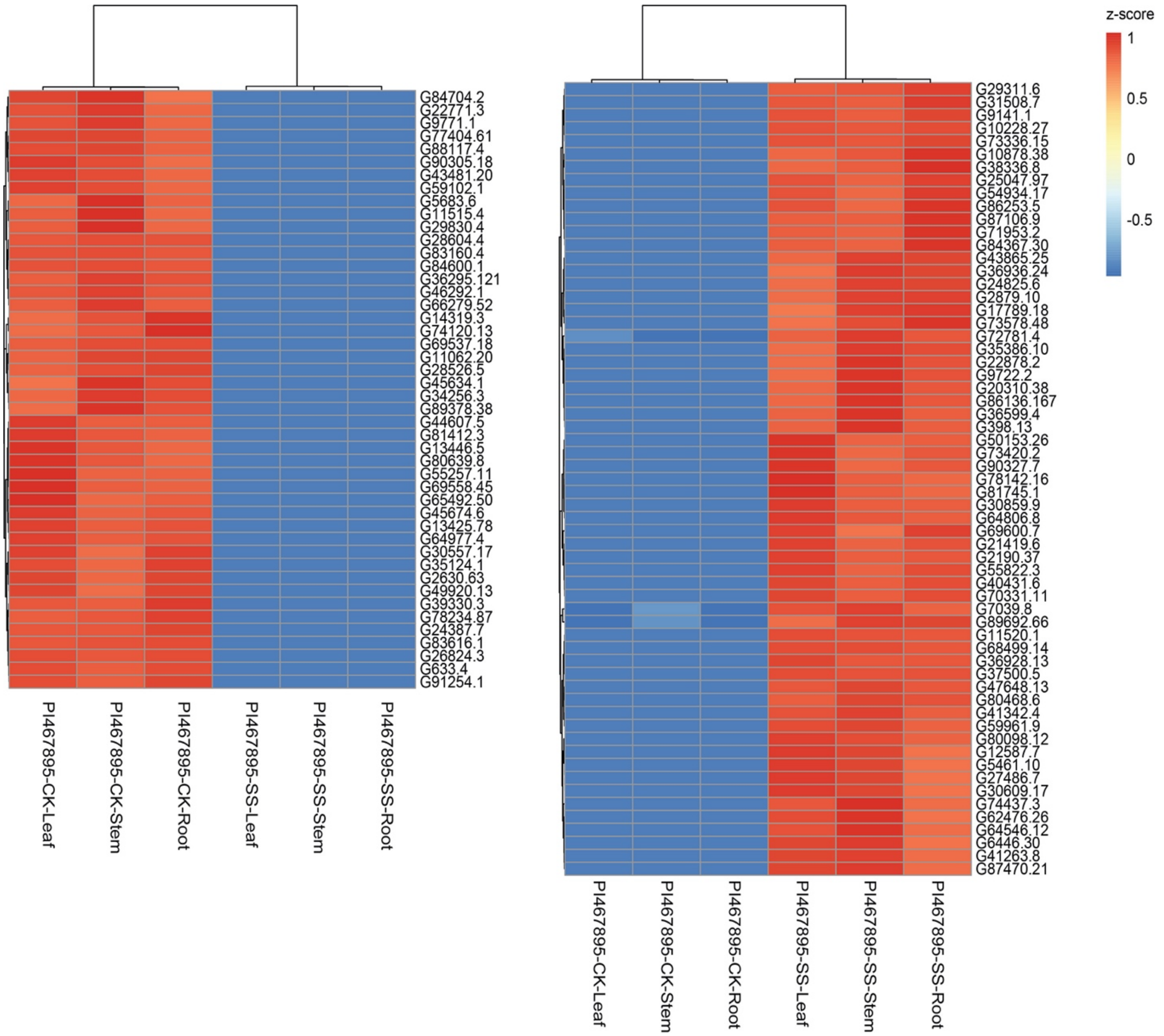

Supplementary Figure 5. Differential expressed genes (DEG) isoforms between control (CK) and drought stress (DS) in Wilson. DEG were normalized using z-score to compare expression of common genes in leaf stem and root tissues under CK and DS. DEG were plotted using pheatmap Version 1.0. 8 R package [84].

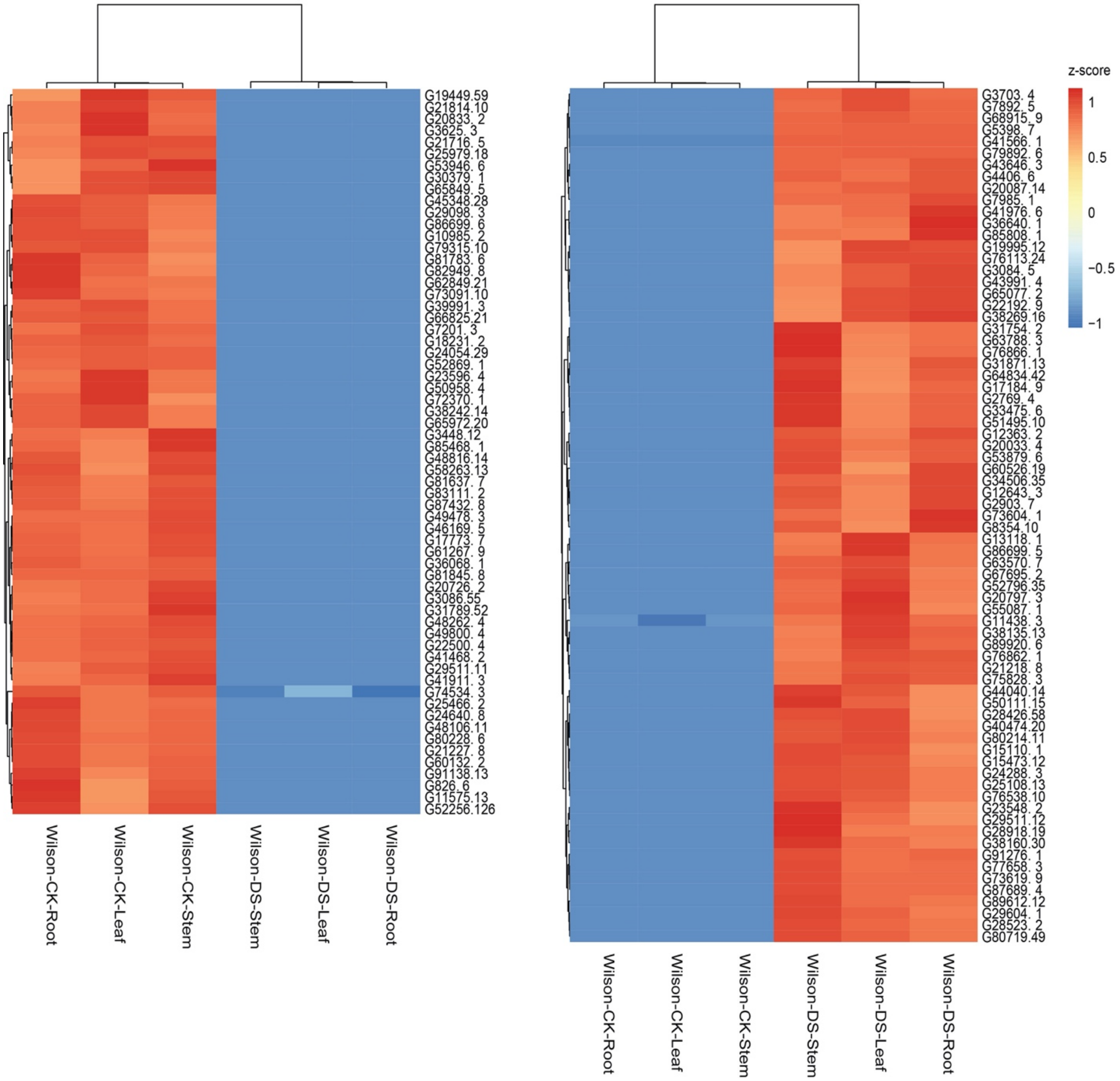

Supplementary Figure 6. Main subnetworks generated from eigengenes connected in PI467895 under salt stress. Red and blue nodes correspond to upregulated and downregulated isoforms, respectively. Node size is proportional to number of edges (degree), circular shapes correspond to isoforms encoding proteins and rhombus shapes correspond to lncRNAs.

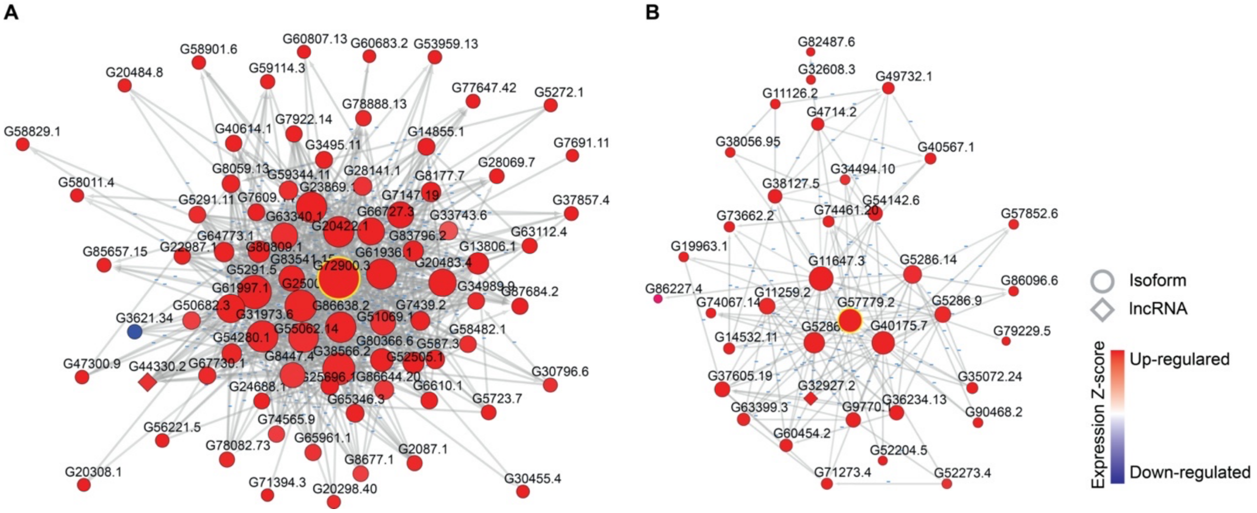

Supplementary Figure 7. Main subnetworks generated from eigengenes connected in Wilson under drought stress. Red and blue nodes correspond to upregulated and downregulated isoforms, respectively. Node size is proportional to number of edges (degree), circular shapes correspond isoforms encoding proteins and rhombus shapes correspond to lncRNAs.

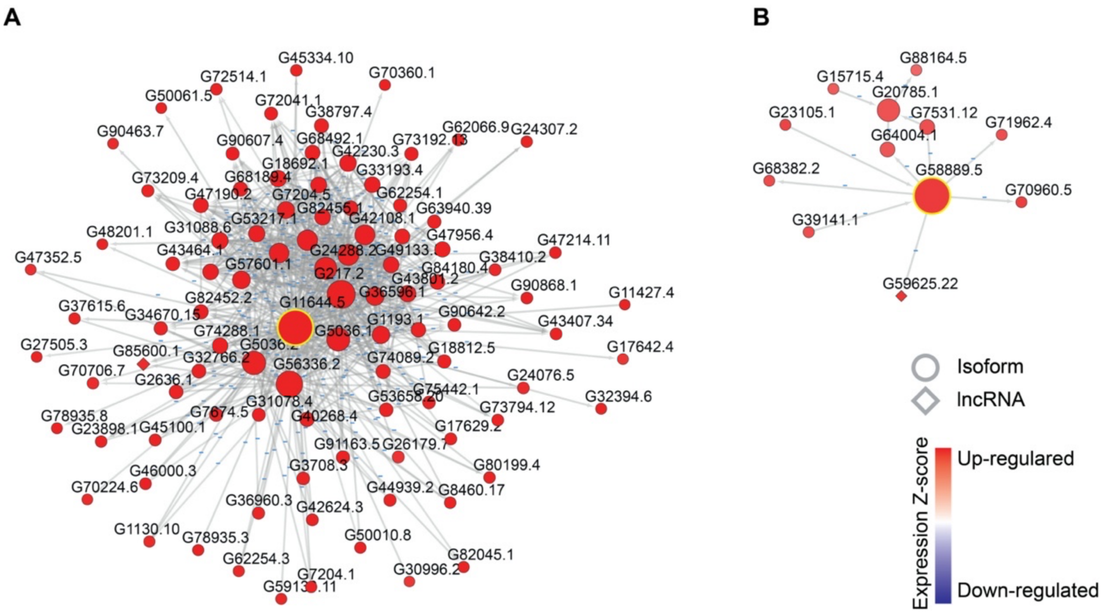

Supplementary Figure 8. Main subnetwork generated from eigengenes connected in Saranac under salt stress. Red and blue nodes correspond to upregulated and downregulated isoforms, respectively. Node size is proportional to number of edges (degree), circular shapes correspond isoforms encoding proteins and rhombus shapes correspond to lncRNAs.

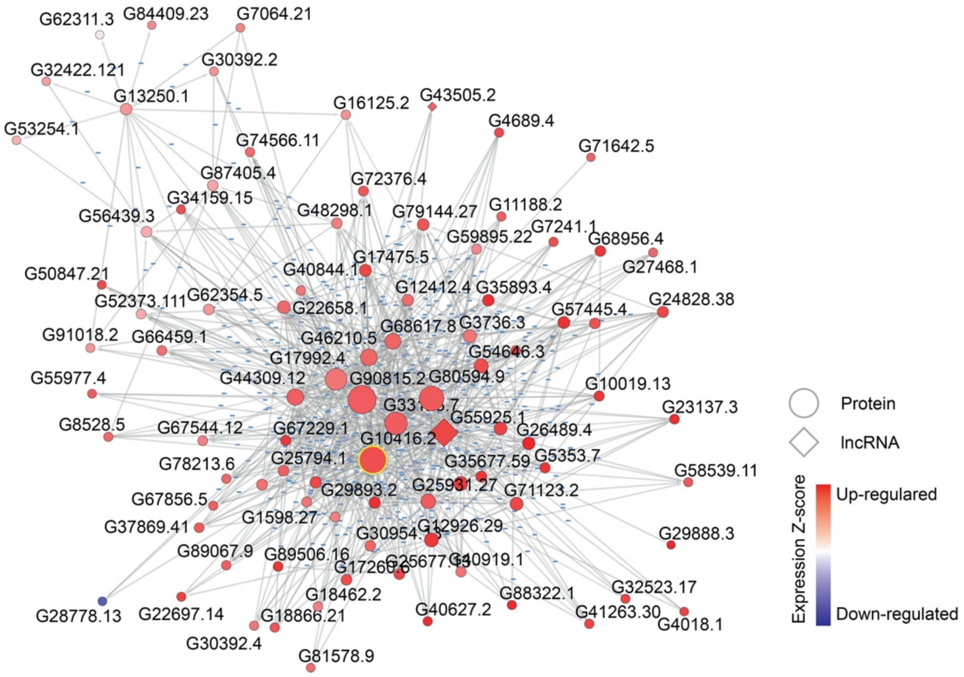

Supplementary Figure 9. Main subnetworks generated from eigengenes connected in Saranac under drought stress. Red and blue nodes correspond to upregulated and downregulated isoforms, respectively. Node size is proportional to number of edges (degree), circular shapes correspond isoforms encoding proteins and rhombus shapes correspond to lncRNAs.

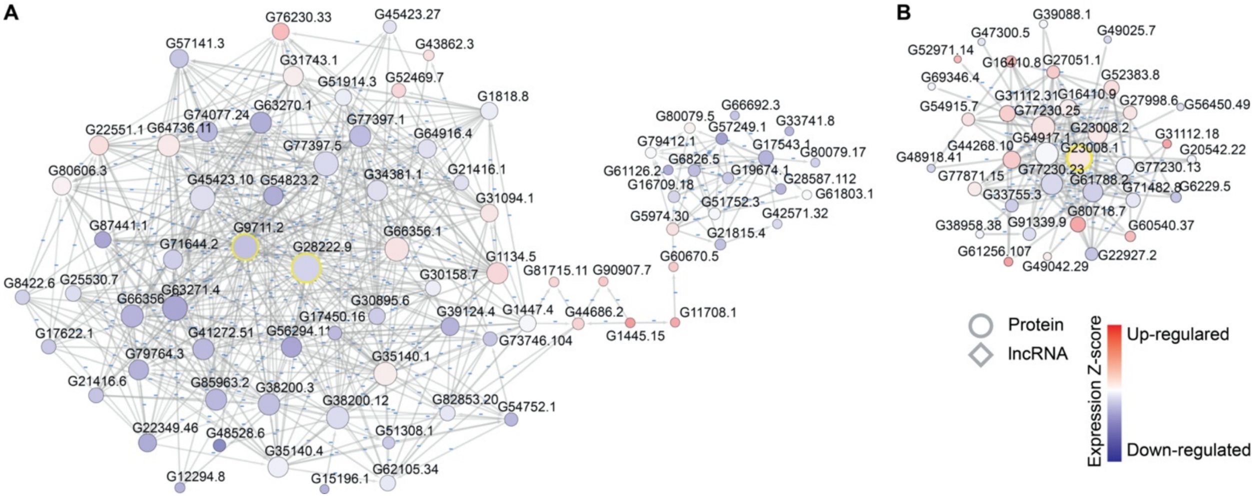

Supplementary Figure 10. Transcriptional regulator HRT-like compared in PI467895-stem up and down regulated by salt stress.

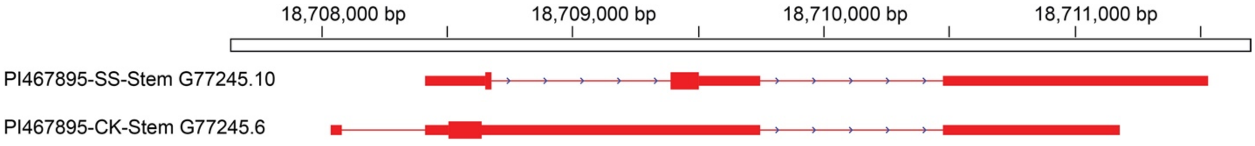

Supplementary Figure 11. Dehydrin isoforms distribution in rows across 21 full-length transcriptomes in columns. Green cells represent isoform presence in transcriptome.

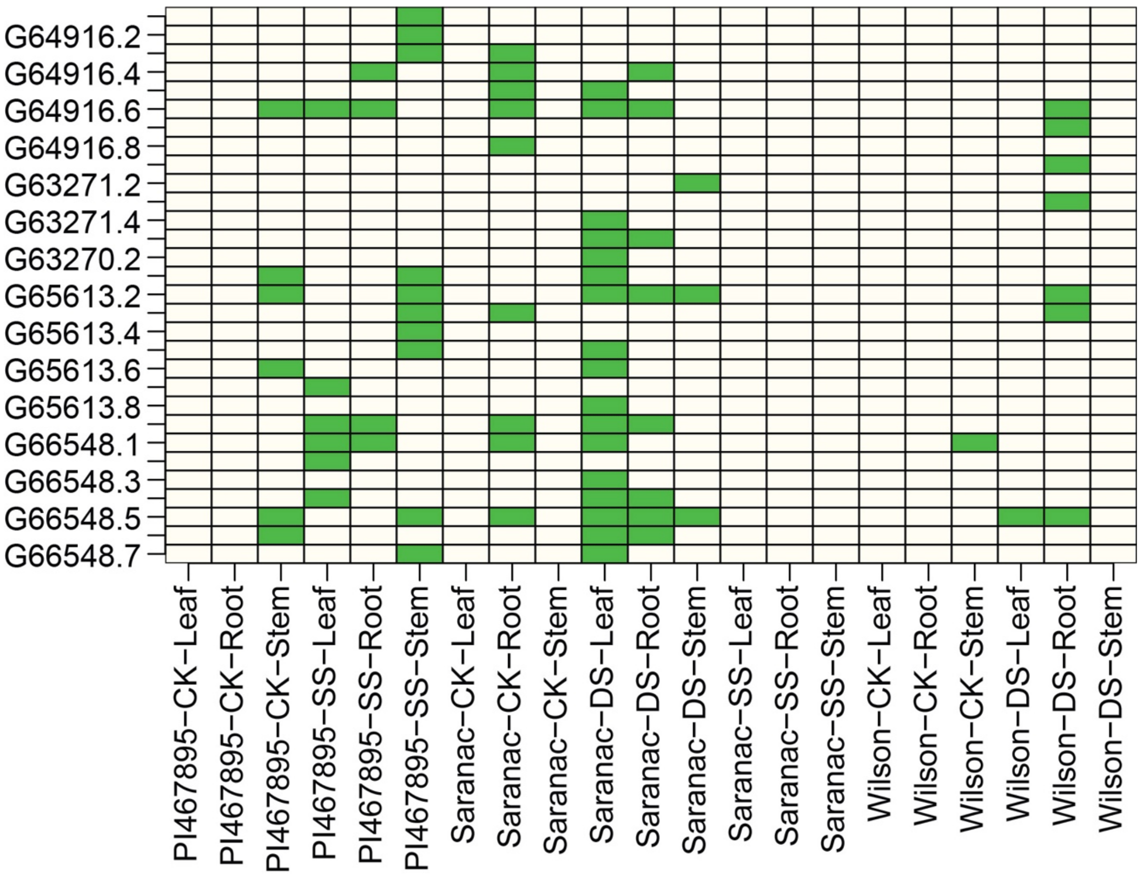

Supplementary Figure 12. SOS1 isoforms distribution in rows across 21 full-length transcriptomes in columns. Green cells represent isoform presence in transcriptome.

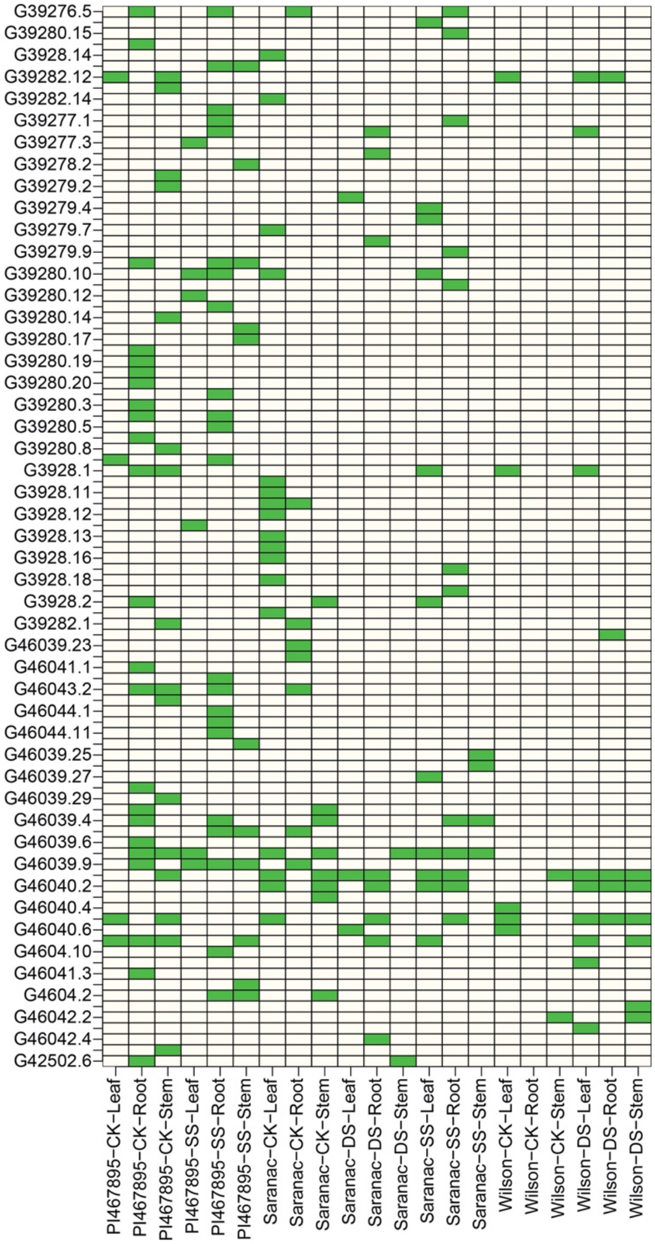

Supplement: Supplementary file 1 — Supplementary Information 1. [file 41598_2021_96712_MOESM1_ESM.pdf]
