# Supplementary material for: Pan-transcriptome identifying master genes and regulation network in response to drought and salt stresses in Alfalfa (Medicago sativa L.)
Source: Sci Rep. 2021 Aug 26;11:17203. doi: 10.1038/s41598-021-96712-x (PMC8390513; doi:10.1038/s41598-021-96712-x)
Supplement: Supplementary file 2 — Supplementary Information 2. [file 41598_2021_96712_MOESM2_ESM.pdf]

Pan-Transcriptome Identifying Master Genes and Regulation Network in Response to Drought and Salt Stresses in Alfalfa (*Medicago sativa* L.)

Cesar Augusto Medina1, Deborah A. Samac2 and Long-Xi Yu1*

1 United States Department of Agriculture-Agricultural Research Service, Plant Germplasm Introduction and Testing Research, Prosser, WA 99350, USA; cesar.medinaculma@wsu.edu

2 United States Department of Agriculture-Agricultural Research Service, Plant Science Research Unit, 1991 Upper Buford Circle, 495 Borlaug Hall St. Paul, MN 55108;
[illegible]

* Corresponding author: Long-Xi Yu (longxi.yu@usda.gov)

Supplementary Table 1. Analysis of seven SMRT cells generated by LIMA. Each SMRT cell was multiplexed with three samples.

**Parameter**	**LID50568**	**LID50569**	**LID50570**	**LID50571**	**LID50572**	**LID50573**	**LID50574**
ZMWs input (A)	2,996,218	3,307,976	2,584,542	2,602,140	2,620,134	3,144,744	2,150,176
ZMWs above all thresholds (B)	2,553,423	2,973,260	2,350,517	2,232,934	2,292,726	2,757,995	1,847,111
ZMWs below any threshold (C)	442,795	334,716	234,025	369,206	327,408	386,749	303,065
Below min length (C)	115	20	29	142	163	68	167
Below min score (C)	0	0	0	0	0	0	0
Below min end score (C)	105,014	84,760	59,973	78,295	74,433	118,577	131,195
Below min passes (C)	79	226	173	0	128	741	2
Below min score lead (C)	0	0	0	0	0	0	0
Below min ref span (C)	336,966	222,910	149,141	237,318	216,955	233,735	222,856
Without SMRT bell adapter (C)	79	226	173	0	128	741	0
Undesired hybrids (C)	142,026	59,745	91,849	141,793	108,325	100,293	90,630
Undesired 5p--5p pairs (C)	69,602	93,173	63,963	110,976	93,724	172,648	104,269
Undesired 3p--3p pairs (C)	96,965	84,340	61,587	62,400	63,787	77,035	68,855
Undesired no hit (C)	79	226	173	0	128	741	0
With different pair (B)	2,553,423	2,973,260	2,350,517	2,232,934	2,292,726	2,757,995	1,847,111
Coefficient of correlation (B)	65.30%	116.50%	87.16%	11.98%	82.77%	34.35%	84.40%
Allow diff pair (A)	2,996,139	3,307,750	2,584,369	2,602,140	2,620,006	3,144,003	2,150,174
Allow same pair (A)	2,996,139	3,307,750	2,584,369	2,602,140	2,620,006	3,144,003	2,150,174
Reads for (B) Above length	2,568,785	2,977,995	2,362,079	2,246,641	2,303,893	2,768,251	1,855,062
Reads for (B) Below length	0	0	0	0	0	0	0

ZMW, Zero-Mode Waveguide; A, Total ZMWs input; B, ZMWs above all thresholds; C, ZMWs below any threshold

Supplementary Table 2. Summary of number of zero mode wavelength (ZMW) reads passed quality control, full length non-concatemer (FLNC) reads passed quality control and the number and length of corrected and trimmed reads with LoRDEC.

Plant	Condition	Tissue	SMRTcell	ZMW	FLNC	LoRDEC	Length**
PI467895	SS	Leaf	LID50569_1004	1,018,143	1,014,677	1,014,457	2,227
PI467895	SS	Stem	LID50569_1003	1,184,956	1,180,236	1,180,161	2,204
PI467895	SS	Root	LID50573_1004	1,203,502	1,199,287	1,198,879	2,382
PI467895	CK	Leaf	LID50569_1002	731,987	704,165	704,077	2,014
PI467895	CK	Stem	LID50568_1001	1,001,710	996,841	996,784	2,338
PI467895	CK	Root	LID50572_1003	909,130	905,429	905,014	2,470
Saranac	DS	Leaf	LID50570_1005	656,883	653,540	652,999	2,188
Saranac	DS	Stem	LID50568_1006	519,534	489,272	489,151	2,403
Saranac	DS	Root	LID50570_1008	611,125	593,469	593,215	2,677
Saranac	SS	Leaf	LID50568_1020	1,025,535	1,020,745	1,020,648	2,382
Saranac	SS	Stem	LID50573_1006	478,947	456,594	456,574	2,310
Saranac	SS	Root	LID50573_1005	1,075,546	1,071,483	1,071,136	2,302
Saranac	CK	Leaf	LID50570_1012	1,067,380	1,061,790	1,061,656	2,724
Saranac	CK	Stem	LID50571_1019	744,240	739,015	738,910	2,145
Saranac	CK	Root	LID50571_1018	853,557	849,498	849,202	2,571
Wilson	DS	Leaf	LID50571_1023	635,137	631,766	1,230,654	2,686
Wilson	DS	Stem	LID50572_1001	747,528	744,938	857,514	2,544
Wilson	DS	Root	LID50574_1008	497,911	483,086	659,561	3,584
Wilson	CK	Leaf	LID50574_1012	781,363	777,618	777,548	3,109
Wilson	CK	Stem	LID50574_1018	556,696	553,680	553,636	3,568
Wilson	CK	Root	LID50572_1002	294,157	292,602	292,524	4,051

CK, Control; SS, Salt Stress; DS, Drought Stress; **, Mean length of transcript

Supplementary Table 3. Set distribution of isoform and gene counts grouped by tissue in PI46789, Saranac and Wilson.

	**Germplasm**	**L∩S∩R[1]**	**Stress**	**L∩S∩R[2]**	**(S∩R)\\(L)**	**(L∩R)\\(S)**	**(L∩S)\\(R)**	**(L)\\(S∪R)**	**(S)\\(L∪R)**	**(R)\\(L∪S)**
Isoform	PI467895	14,860	SS	35,958	20,516	11,028	22,853	74,643	95,969	91,857
			CK	21,260	19,878	6,052	23,444	60,858	100,189	94,690
	Saranac	9,438	CK	21,047	13,137	7,386	87,778	26,228	88,060	84,151
			DS	20,522	10,792	13,941	73,377	10,247	57,930	69,292
			SS	29,699	11,851	20,768	91,198	12,901	49,807	94,441
	Wilson	9,140	DS	21,340	5,471	7,991	27,605	97,797	58,131	43,425
			CK	12,336	4,680	4,087	14,843	58,657	47,409	20,825
Gene	PI467895	17,018	SS	28,703	4,885	2,637	5,738	4,631	5,416	7,558
			CK	20,362	7,433	1,577	8,846	3,050	8,416	12,380
	Saranac	11,909	CK	19,852	4,670	2,246	11,878	9,910	7,690	4,207
			DS	12,214	2,273	13,098	7,634	1,444	4,246	9,612
			SS	24,807	2,731	6,432	8,455	3,926	2,873	6,964
	Wilson	11,597	DS	20,950	1,482	2,404	10,998	7,880	5,045	3,178
			CK	13,170	1,904	1,794	8,165	7,057	5,801	3,309

L. leaf; S, stem; R, root; DS, drought stress; SS, salt stress; CK control; \, exclusion; ∩ intersection; ∪, union; 1, core genes and isoforms by germplasm; 2 core genes and isoforms by germplasm and condition.

Supplementary Table 4. Set distribution of isoform and gene counts grouped by treatment in PI467895, Saranac and Wilson.

		Isoform			Gene		
**Germplasm**	**Set**	**Leaf**	**Stem**	**Root**	**Leaf**	**Stem**	**Root**
PI467895	SS∩CK	35,316	58,055	49,012	24,710	34,272	30,997
PI467895	(CK)\(SS)	76,298	83,825	115,759	9,125	7,480	14,060
PI467895	(SS)\(CK)	109,166	101,304	126,284	16,999	9,511	13,745
Saranac	CK∩DS∩SS	30,863	24,459	21,293	25,523	21,132	18,974
Saranac	(CK∩SS)\(D	19,140	13,074	22,813	4,894	4,741	10,131
Saranac	(CK∩DS)\(S	10,858	7,793	12,664	2,151	2,181	3,646
Saranac	(DS∩SS)\(C	14,414	21,864	6,453	4,493	8,425	1,304
Saranac	(SS)\(CK∪D	93,392	94,119	53,699	7,219	8,127	3,928
Saranac	(DS)\(CK∪S	57,867	64,516	59,081	4,201	3,481	3,747
Saranac	(CK)\(DS∪S	77,951	84,022	91,702	3,647	10,592	9,371
Wilson	DS∩CK	38,040	17,159	31,807	26,347	15,664	24,387
Wilson	(CK)\(DS)	51,883	24,769	47,461	3,839	4,513	4,653
Wilson	(DS)\(CK)	116,693	61,068	80,740	15,885	12,350	14,088

DS, drought stress; SS, salt stress; CK control; \, right exclusion; ∩ intersection; ∪, union.

Supplementary Table 5. Total number of genes and isoforms annotated as

Family	Count_gene	Count_isoform	Class
ARID	42	590	Transcriptional regulator
AUX/IAA	79	855	Transcriptional regulator
Coactivator p15	17	86	Transcriptional regulator
GNAT	86	628	Transcriptional regulator
HMG	25	232	Transcriptional regulator
IWS1	15	586	Transcriptional regulator
Jumonji	46	941	Transcriptional regulator
LUG	12	501	Transcriptional regulator
MBF1	5	21	Transcriptional regulator
MED6	3	21	Transcriptional regulator
MED7	3	13	Transcriptional regulator
mTERF	182	1,258	Transcriptional regulator
Others	152	2,768	Transcriptional regulator
PHD	100	2,123	Transcriptional regulator
Pseudo ARR-B	5	133	Transcriptional regulator
RB	2	89	Transcriptional regulator
Rcd1-like	3	11	Transcriptional regulator
SET	93	1,368	Transcriptional regulator
SNF2	127	2,613	Transcriptional regulator
SOH1	4	122	Transcriptional regulator
SWI/SNF-BAF60b	45	490	Transcriptional regulator
SWI/SNF-SWI3	18	314	Transcriptional regulator
TAZ	18	490	Transcriptional regulator
TRAF	56	831	Transcriptional regulator
Total	1,138	17,084	
AGC	102	1,711	Protein kinase
AGC-Pl	7	236	Protein kinase
Aur	12	57	Protein kinase
AurL-Cr-1	4	250	Protein kinase
BUB	3	25	Protein kinase
CAMK	243	3,610	Protein kinase
CK1	46	1,739	Protein kinase
CMGC	245	5,614	Protein kinase
Group-Pl-2	2	8	Protein kinase
Group-Pl-3	5	52	Protein kinase
Group-Pl-4	5	118	Protein kinase
IRE1	6	58	Protein kinase
NAK	2	6	Protein kinase
NEK	13	288	Protein kinase
PEK	6	226	Protein kinase
RLK-Pelle	2,535	24,352	Protein kinase
SCY1	8	274	Protein kinase
STE	85	1,074	Protein kinase

TKL	29	738	Protein kinase
TKL-Cr-3	1	2	Protein kinase
TKL-Pl-1	6	113	Protein kinase
TKL-Pl-2	3	99	Protein kinase
TKL-Pl-3	3	25	Protein kinase
TKL-Pl-4	56	1,052	Protein kinase
TKL-Pl-5	10	226	Protein kinase
TKL-Pl-6	15	220	Protein kinase
TKL-Pl-7	7	52	Protein kinase
TKL-Pl-8	4	19	Protein kinase
TLK	5	65	Protein kinase
TTK	8	118	Protein kinase
ULK	9	65	Protein kinase
WEE	1	3	Protein kinase
WNK	33	511	Protein kinase
Total	3,519	43,006	
AP2	60	802	Transcription factor
ARF	71	1,986	Transcription factor
ARR-B	34	829	Transcription factor
B3	143	1,610	Transcription factor
BBR-BPC	13	154	Transcription factor
BES1	21	339	Transcription factor
bHLH	423	4,375	Transcription factor
bZIP	222	3,467	Transcription factor
C2H2	238	1,905	Transcription factor
C3H	139	2,396	Transcription factor
CAMTA	28	779	Transcription factor
CO-like	27	302	Transcription factor
CPP	21	279	Transcription factor
DBB	23	393	Transcription factor
Dof	122	636	Transcription factor
E2F/DP	26	428	Transcription factor
EIL	12	114	Transcription factor
ERF	257	1,333	Transcription factor
FAR1	181	1,649	Transcription factor
G2-like	143	2,327	Transcription factor
GATA	94	802	Transcription factor
GeBP	12	51	Transcription factor
GRAS	154	1,123	Transcription factor
GRF	27	238	Transcription factor
HB-other	46	831	Transcription factor
HB-PHD	4	54	Transcription factor
HD-ZIP	146	1,540	Transcription factor
HRT-like	6	22	Transcription factor
HSF	66	913	Transcription factor

LBD	58	226	Transcription factor
LFY	1	1	Transcription factor
LSD	15	150	Transcription factor
M-type_MADS	25	163	Transcription factor
MIKC_MADS	56	534	Transcription factor
MYB	274	1,850	Transcription factor
MYB_related	192	3,424	Transcription factor
NAC	249	1,983	Transcription factor
NF-X1	10	87	Transcription factor
NF-YA	29	649	Transcription factor
NF-YB	24	138	Transcription factor
NF-YC	37	334	Transcription factor
Nin-like	23	717	Transcription factor
RAV	7	51	Transcription factor
S1Fa-like	7	24	Transcription factor
SBP	76	1,080	Transcription factor
SRS	19	81	Transcription factor
STAT	4	50	Transcription factor
TALE	69	1,175	Transcription factor
TCP	60	460	Transcription factor
Trihelix	105	747	Transcription factor
VOZ	10	183	Transcription factor
Whirly	9	58	Transcription factor
WOX	15	63	Transcription factor
WRKY	237	1,870	Transcription factor
YABBY	20	69	Transcription factor
ZF-HD	27	111	Transcription factor
Total	4,417	47,955	

Supplementary Table 6. Total number of isoforms annotated as protein kinases, transcription factors and transcription regulators in 21 transcriptomes of alfalfa

	PI467895						Saranac									Wilson						
Family	CK-Leaf	CK-Roo	CK-Sten	SS-Leaf	SS-Root	SS-Stem	CK-Leaf	CK-Roo	CK-Sten	DS-Leaf	DS-Root	DS-Stem	SS-Leaf	SS-Root	SS-Stem	CK-Leaf	CK-Roo	CK-Sten	DS-Leaf	DS-Root	DS-Stem	Class
AGC	198	243	279	237	249	256	233	205	266	196	173	119	266	246	187	127	74	131	230	109	193	Protein kinase
AGC-Pl	42	30	37	53	26	29	40	42	33	24	29	37	30	22	16	20	7	15	23	16	13	Protein kinase
Aur	0	7	10	3	6	1	5	8	13	6	5	2	8	6	2	0	0	0	4	1	15	Protein kinase
AurL-Cr-1	13	29	44	23	33	24	50	29	38	44	40	49	40	24	37	23	6	12	30	19	9	Protein kinase
BUB	0	3	1	0	3	1	1	3	4	3	1	0	2	1	0	0	0	1	3	1	4	Protein kinase
CAMK	404	617	562	471	642	649	459	532	474	425	448	405	480	677	376	315	144	262	467	263	349	Protein kinase
CK1	196	201	224	179	293	283	203	210	192	163	187	163	180	242	176	163	66	128	204	129	154	Protein kinase
CMGC	563	669	887	699	815	897	655	671	749	553	594	609	698	748	557	501	247	417	792	451	579	Protein kinase
Group-Pl-2	1	2	2	1	1	0	0	0	4	2	1	0	1	1	0	0	0	0	1	0	1	Protein kinase
Group-Pl-3	4	1	2	9	5	9	7	3	2	11	2	3	6	5	3	1	0	7	3	3	0	Protein kinase
Group-Pl-4	44	4	29	22	5	22	24	3	21	18	0	10	19	2	7	21	1	8	18	3	9	Protein kinase
IRE1	2	11	7	13	10	11	6	11	7	5	10	8	6	3	2	5	5	4	6	9	3	Protein kinase
NEK	28	39	52	30	40	22	26	23	55	17	33	19	33	50	35	31	15	31	38	27	27	Protein kinase
PEK	14	26	36	48	14	41	31	10	15	5	15	6	24	15	14	10	4	9	22	17	19	Protein kinase
RLK-Pelle	2,084	3,476	3,824	2,833	3,753	3,360	2,879	2,448	3,238	2,067	2,302	1,751	3,135	3,937	2,106	2,325	928	1,678	3,500	1,243	2,631	Protein kinase
SCY1	15	41	24	21	34	26	28	31	24	30	39	23	50	53	24	21	12	15	24	31	26	Protein kinase
STE	101	123	154	117	164	150	118	117	114	99	105	117	118	133	80	95	32	81	133	73	101	Protein kinase
TKL	79	86	100	89	89	104	100	103	90	65	88	30	94	105	34	50	17	34	81	31	44	Protein kinase
TKL-Cr-3	0	0	2	0	0	0	1	0	0	0	0	0	0	0	0	0	0	0	0	0	0	Protein kinase
TKL-Pl-1	13	22	20	17	20	16	16	27	11	12	16	29	8	13	16	7	9	8	13	6	7	Protein kinase
TKL-Pl-2	12	10	11	19	16	17	16	12	31	18	14	23	19	25	23	15	8	13	17	12	18	Protein kinase
TKL-Pl-3	0	2	2	4	1	7	5	2	2	4	3	3	8	2	2	2	1	3	5	2	2	Protein kinase
TKL-Pl-4	119	142	174	139	155	170	127	127	150	109	131	126	126	210	107	81	46	86	148	86	108	Protein kinase
TKL-Pl-5	13	30	28	33	37	51	33	27	29	27	31	34	22	47	28	21	8	8	29	19	22	Protein kinase
TKL-Pl-6	27	35	37	28	35	31	38	34	31	25	41	40	37	42	27	16	5	31	25	13	25	Protein kinase
TKL-Pl-7	5	7	8	5	10	3	4	4	11	2	4	0	6	7	5	2	2	3	8	4	6	Protein kinase
TKL-Pl-8	1	0	3	0	0	2	1	0	8	1	0	0	0	0	6	0	0	1	0	0	0	Protein kinase
TLK	6	8	9	8	8	13	10	4	6	4	5	4	10	11	6	11	3	10	10	10	6	Protein kinase
TTK	15	20	23	15	22	11	12	19	19	4	12	4	18	19	12	5	2	4	23	8	28	Protein kinase
ULK	1	24	7	6	7	4	6	7	11	1	7	1	4	12	2	1	2	5	14	2	12	Protein kinase
WEE	1	0	1	0	0	0	0	0	0	0	0	0	0	0	0	0	0	0	0	0	1	Protein kinase
WNK	33	59	36	44	58	55	45	62	49	42	69	170	64	81	41	20	7	18	37	22	25	Protein kinase
NAK	1	0	0	0	0	2	0	0	0	0	0	0	0	0	0	1	1	2	3	1	3	Protein kinase
Total	4,035	5,967	6,635	5,166	6,551	6,267	5,179	4,774	5,697	3,982	4,405	3,785	5,512	6,739	3,931	3,890	1,652	3,025	5,911	2,611	4,440	
Max value	2,084	3,476	3,824	2,833	3,753	3,360	2,879	2,448	3,238	2,067	2,302	1,751	3,135	3,937	2,106	2,325	928	1,678	3,500	1,243	2,631	
AP2	44	72	102	122	88	133	91	86	117	69	73	62	97	97	82	26	25	37	102	52	74	Transcription factor
ARF	225	269	413	223	322	253	274	213	335	201	277	166	375	308	253	173	84	225	257	127	263	Transcription factor
ARR-B	97	85	230	89	141	89	81	106	172	86	75	68	113	130	121	46	41	63	98	76	129	Transcription factor
B3	113	221	202	172	240	221	173	139	190	136	167	109	243	196	127	144	62	113	215	94	168	Transcription factor
BBR-BPC	11	28	21	25	40	38	18	23	24	12	22	14	22	23	17	7	4	9	20	9	17	Transcription factor
BES1	34	50	47	33	61	60	38	41	51	31	36	29	48	59	32	23	8	33	38	25	41	Transcription factor
bHLH	488	609	660	526	611	611	553	443	579	363	383	294	500	647	393	277	143	286	604	222	390	Transcription factor
bZIP	282	512	496	362	571	509	320	671	416	385	493	331	341	542	274	217	155	188	412	352	275	Transcription factor
C2H2	235	296	366	331	312	415	258	247	300	210	213	138	348	287	219	195	79	179	330	125	263	Transcription factor
C3H	353	436	432	393	478	481	362	485	379	309	359	280	368	395	307	251	122	204	372	319	292	Transcription factor
CAMTA	114	161	130	104	151	158	140	124	140	120	143	112	122	136	122	85	49	79	123	74	122	Transcription factor
CO-like	68	18	76	92	24	126	62	26	54	74	26	41	94	29	38	34	5	24	73	11	52	Transcription factor
CPP	16	39	37	18	55	19	14	22	55	33	32	9	26	31	17	9	7	20	38	12	33	Transcription factor
DBB	45	33	55	108	33	89	42	41	46	36	21	27	88	34	26	54	7	41	80	23	50	Transcription factor
Dof	69	81	169	80	126	124	66	121	107	81	80	33	88	129	79	49	24	55	82	57	77	Transcription factor
E2F/DP	39	31	43	27	50	50	47	37	50	33	37	61	66	57	30	22	5	17	57	22	45	Transcription factor
EIL	18	33	36	30	42	46	27	37	31	32	33	42	22	32	23	24	14	23	26	25	21	Transcription factor
ERF	144	188	270	174	280	268	135	201	197	186	172	132	186	264	141	86	58	99	187	103	152	Transcription factor
FAR1	105	222	202	182	250	231	187	198	196	165	200	154	231	219	162	131	69	114	235	129	163	Transcription factor
G2-like	225	293	337	344	311	387	310	219	274	217	171	220	329	278	211	200	60	155	305	125	198	Transcription factor

GATA	116	92	145	134	107	134	118	83	122	87	73	47	155	114	98	71	24	50	136	40	94	Transcription factor
GeBP	6	12	9	9	14	12	8	13	8	8	10	6	11	12	11	10	5	8	11	8	15	Transcription factor
GRAS	130	293	230	194	271	235	155	267	186	149	194	148	169	244	157	141	107	144	194	143	200	Transcription factor
GRF	21	37	32	47	26	35	42	26	31	32	26	3	50	25	25	17	3	26	48	23	42	Transcription factor
HB-other	76	114	138	93	118	169	123	102	156	93	97	96	125	121	114	67	42	69	124	53	95	Transcription factor
HB-PHD	8	7	5	7	10	8	4	7	3	4	2	1	5	3	4	11	3	2	8	4	5	Transcription factor
HD-ZIP	209	206	357	232	226	283	205	175	284	200	195	113	255	226	193	149	66	157	270	140	252	Transcription factor
HRT-like	3	1	2	1	1	2	3	4	1	2	0	0	3	2	1	2	0	3	5	0	0	Transcription factor
HSF	81	95	172	113	131	154	102	148	85	114	135	102	155	144	87	88	67	129	105	162	85	Transcription factor
LBD	9	36	22	19	46	32	10	36	20	16	25	6	20	52	8	17	9	11	20	13	18	Transcription factor
LSD	21	9	17	18	15	26	14	26	18	7	5	6	28	8	7	4	2	4	14	5	8	Transcription factor
M-type_MADS	14	8	11	7	6	10	14	6	21	17	11	5	22	19	10	6	3	9	16	10	10	Transcription factor
MIKC_MADS	65	40	72	66	75	72	50	57	48	68	37	12	63	65	52	21	9	28	57	38	28	Transcription factor
MYB	131	270	296	233	300	277	206	305	261	234	263	226	284	288	181	126	67	151	285	168	235	Transcription factor
MYB_related	467	363	551	449	432	531	622	343	552	402	299	297	486	445	421	322	101	300	545	251	314	Transcription factor
NAC	105	194	261	171	301	287	156	339	209	268	340	400	209	338	172	148	86	128	200	215	163	Transcription factor
NF-X1	5	15	12	6	14	8	12	15	12	8	15	21	13	15	10	11	4	7	9	6	11	Transcription factor
NF-YA	54	73	89	61	119	87	64	118	75	84	114	61	78	113	69	35	20	40	75	56	35	Transcription factor
NF-YB	15	11	16	21	14	22	15	23	20	20	17	13	18	15	11	8	4	4	21	9	15	Transcription factor
NF-YC	41	36	59	49	46	56	36	55	48	47	40	55	46	46	30	22	17	28	40	28	25	Transcription factor
Nin-like	35	126	62	72	126	51	98	140	62	42	147	58	67	105	51	96	30	74	112	64	38	Transcription factor
RAV	10	5	9	11	17	17	6	10	10	15	4	5	10	26	4	6	1	2	13	1	8	Transcription factor
S1Fa-like	0	4	7	2	4	5	2	2	3	1	1	0	1	4	4	4	0	1	4	1	4	Transcription factor
SBP	105	115	178	137	150	180	112	149	188	127	143	78	135	163	124	105	44	111	148	75	142	Transcription factor
SRS	5	13	17	9	19	19	4	14	19	9	9	6	12	18	7	5	2	7	6	4	13	Transcription factor
STAT	3	8	6	1	6	6	8	2	6	4	4	1	9	14	7	5	0	3	5	1	2	Transcription factor
TALE	138	111	292	169	150	288	166	166	251	135	144	148	180	131	187	125	97	129	179	71	149	Transcription factor
TCP	75	68	91	108	67	104	107	54	94	64	44	42	137	82	66	57	40	33	107	53	66	Transcription factor
Trihelix	102	109	153	162	136	186	142	107	144	120	103	95	178	138	92	92	36	66	164	72	137	Transcription factor
VOZ	26	15	30	28	19	30	21	30	21	23	15	15	20	13	20	20	6	14	30	17	20	Transcription factor
Whirly	6	7	8	12	11	14	4	8	12	8	6	4	12	6	6	6	1	4	10	5	5	Transcription factor
WOX	4	5	14	11	8	15	11	2	13	8	4	0	19	12	10	3	3	3	9	2	13	Transcription factor
WRKY	124	308	319	157	340	290	186	304	207	188	212	184	178	368	151	182	80	102	226	105	170	Transcription factor
YABBY	13	0	14	12	1	0	5	1	4	6	0	0	19	0	2	2	0	0	21	0	2	Transcription factor
ZF-HD	10	3	23	22	6	22	9	6	16	16	5	4	27	7	15	15	2	9	29	2	29	Transcription factor
LFY	0	0	0	0	0	0	1	0	0	1	1	0	0	0	0	0	0	0	0	0	0	Transcription factor
Total	4,958	6,486	8,043	6,278	7,518	7,975	6,039	6,623	6,923	5,406	5,753	4,580	6,906	7,275	5,081	4,052	2,002	3,820	6,900	3,827	5,273	
Max value	488	609	660	526	611	611	622	671	579	402	493	400	500	647	421	322	155	300	604	352	390	
ARID	56	74	110	74	77	89	44	51	70	48	54	48	79	61	48	52	27	39	89	42	64	Transcriptional regulator
AUX/IAA	120	128	228	147	120	152	81	94	212	104	105	58	142	117	122	68	28	96	114	63	139	Transcriptional regulator
Coactivator p15	4	7	14	11	15	21	5	10	11	5	8	1	7	7	6	5	2	6	7	2	12	Transcriptional regulator
GNAT	64	58	93	76	76	82	88	69	90	76	40	41	86	63	46	49	11	28	99	47	61	Transcriptional regulator
HMG	39	18	35	39	23	32	39	18	36	41	18	13	45	29	23	15	7	17	42	18	35	Transcriptional regulator
IWS1	51	94	104	58	124	79	83	108	92	107	109	119	93	144	70	57	35	49	84	70	70	Transcriptional regulator
Jumonji	68	99	159	110	115	144	101	125	129	95	147	137	128	113	103	82	46	65	99	64	81	Transcriptional regulator
LUG	48	74	61	80	67	90	69	54	84	59	63	61	96	75	70	60	25	51	84	55	76	Transcriptional regulator
MBF1	0	3	3	7	2	4	3	3	2	4	1	3	1	1	1	3	1	1	4	5	3	Transcriptional regulator
MED6	2	4	4	3	3	3	2	4	1	2	4	2	3	3	3	3	4	3	4	2	2	Transcriptional regulator
MED7	0	0	5	3	1	2	0	1	2	0	3	0	2	1	1	0	0	0	0	1	1	Transcriptional regulator
mTERF	101	104	152	199	132	202	153	80	123	106	91	67	232	137	119	93	33	71	210	57	123	Transcriptional regulator
PHD	214	358	312	312	373	357	296	373	322	246	347	340	372	371	281	201	109	189	364	222	260	Transcriptional regulator
Pseudo ARR-B	1	10	9	25	14	31	14	18	15	21	12	16	36	12	11	5	5	9	34	10	31	Transcriptional regulator
RB	8	8	15	7	12	10	11	11	17	8	9	14	9	8	9	6	3	11	14	9	7	Transcriptional regulator
Rcd1-like	0	1	6	2	4	1	1	1	3	2	1	0	1	3	0	0	0	1	1	1	1	Transcriptional regulator
SET	129	238	221	202	244	247	141	222	216	157	189	107	246	215	175	140	70	142	235	135	188	Transcriptional regulator
SNF2	237	403	370	331	462	440	414	329	420	297	416	286	402	468	308	227	136	226	449	260	353	Transcriptional regulator
SOH1	5	9	11	9	12	18	18	12	11	13	10	14	22	14	7	7	8	7	16	8	7	Transcriptional regulator
SWI/SNF-BAF	44	59	75	62	72	67	72	77	100	63	82	68	80	76	57	46	24	34	89	54	59	Transcriptional regulator

SWI/SNF-SWI	25	53	45	52	53	54	49	45	49	35	52	41	53	48	39	34	14	25	47	30	33	Transcriptional regulator
TAZ	39	76	66	69	90	86	51	75	57	79	67	81	57	81	28	35	19	37	75	35	47	Transcriptional regulator
TRAF	89	118	152	101	134	159	112	117	142	100	105	118	111	131	64	88	39	49	118	57	95	Transcriptional regulator
Others	331	399	398	469	408	525	420	366	407	372	353	314	441	374	241	212	123	218	401	209	289	Transcriptional regulator
Total	1,344	1,996	2,250	1,979	2,225	2,370	1,847	1,897	2,204	1,668	1,933	1,635	2,303	2,178	1,591	1,276	646	1,156	2,278	1,247	1,748	
Max value	237	403	370	331	462	440	414	373	420	297	416	340	402	468	308	227	136	226	449	260	353	

DS, drought stress; SS, salt stress; CK control

Supplementary Table 7. Total number of isoforms annotated as protein kinases, transcription factors and transcription regulators in alfalfa 21 transcriptomes

	PI467895			Saranac			Saranac			Wilson				
**Family**	**SS-Leaf**	**SS-Stem**	**SS-Root**	**SS-Leaf**	**SS-Stem**	**SS-Root**	**DS-Leaf**	**DS-Stem**	**DS-Root**	**DS-Leaf**	**DS-Stem**	**DS-Root**	**Regulation**	**Class**
AGC	153	155	146	175	99	183	110	67	117	173	133	81	up	Protein kinase
AGC	114	178	140	142	178	142	147	214	149	70	71	46	do	Protein kinase
AGC-Pl	29	15	14	15	9	12	14	25	19	15	10	14	up	Protein kinase
AGC-Pl	18	23	18	25	26	32	30	21	32	12	12	5	do	Protein kinase
Aur	3	1	1	7	0	5	6	2	4	4	15	1	up	Protein kinase
Aur	0	10	2	4	11	7	5	13	7	0	0	0	do	Protein kinase
AurL-Cr-1	21	12	23	30	24	20	38	29	31	22	7	16	up	Protein kinase
AurL-Cr-1	11	32	19	40	25	25	44	18	20	15	10	3	do	Protein kinase
BUB	0	1	2	2	0	1	3	0	1	3	4	1	up	Protein kinase
BUB	0	1	2	1	4	3	1	4	3	0	1	0	do	Protein kinase
CAMK	312	431	349	301	192	475	251	234	281	328	230	181	up	Protein kinase
CAMK	245	344	324	280	290	330	285	303	365	176	143	62	do	Protein kinase
CK1	128	214	208	124	112	188	99	111	128	145	106	101	up	Protein kinase
CK1	145	155	116	147	128	156	139	140	151	104	80	38	do	Protein kinase
CMGC	516	612	519	439	322	546	335	389	426	543	392	336	up	Protein kinase
CMGC	380	602	373	396	514	469	437	529	503	252	230	132	do	Protein kinase
Group-Pl-2	1	0	1	1	0	1	2	0	1	1	1	0	up	Protein kinase
Group-Pl-2	1	2	2	0	4	0	0	4	0	0	0	0	do	Protein kinase
Group-Pl-3	8	9	5	3	3	5	8	3	2	3	0	3	up	Protein kinase
Group-Pl-3	3	2	1	4	2	3	4	2	3	1	7	0	do	Protein kinase
Group-Pl-4	9	13	3	9	3	1	11	6	0	11	4	3	up	Protein kinase
Group-Pl-4	31	20	2	14	17	2	17	17	3	14	3	1	do	Protein kinase
IRE1	12	8	5	3	1	2	3	7	7	3	2	7	up	Protein kinase
IRE1	1	4	6	3	6	10	4	6	8	2	3	3	do	Protein kinase
NAK	0	2	0	0	0	0	0	0	0	2	1	0	up	Protein kinase
NAK	1	0	0	0	0	0	0	0	0	0	0	0	do	Protein kinase
NEK	23	15	23	26	16	35	12	11	25	34	17	25	up	Protein kinase
NEK	21	45	22	19	36	8	21	47	15	27	21	13	do	Protein kinase
PEK	45	35	13	17	11	13	3	6	15	20	17	15	up	Protein kinase
PEK	11	30	25	24	12	8	29	15	10	8	7	2	do	Protein kinase
RLK-Pelle	2,208	2,372	2,233	2,135	1,108	3,140	1,295	1,218	1,690	2,622	1,873	938	up	Protein kinase
RLK-Pelle	1,459	2,836	1,956	1,879	2,240	1,651	2,107	2,705	1,836	1,447	920	623	do	Protein kinase
SCY1	16	19	24	42	17	41	22	14	33	17	18	23	up	Protein kinase
SCY1	10	17	31	20	17	19	20	15	25	14	7	4	do	Protein kinase
STE	86	112	106	75	47	100	64	89	72	95	69	57	up	Protein kinase
STE	70	116	65	75	81	84	83	86	84	57	49	16	do	Protein kinase
TKL	63	80	62	69	26	86	46	20	66	64	38	27	up	Protein kinase
TKL	53	76	59	75	82	84	81	80	81	33	28	13	do	Protein kinase
TKL-Cr-3	0	2	0	1	0	0	1	0	0	0	0	0	do	Protein kinase
TKL-Pl-1	12	10	10	3	10	7	7	22	7	8	3	1	up	Protein kinase
TKL-Pl-1	8	14	12	11	5	21	11	4	18	2	4	4	do	Protein kinase
TKL-Pl-2	16	14	12	11	12	18	9	12	10	12	13	7	up	Protein kinase
TKL-Pl-2	9	8	6	8	20	5	7	20	8	10	8	3	do	Protein kinase

TKL-Pl-3	4	6	0	6	1	1	2	1	2	4	1	1 up	Protein kinase
TKL-Pl-3	0	1	1	3	1	1	3	0	1	1	2	0 do	Protein kinase
TKL-Pl-4	90	106	91	70	58	168	61	78	95	116	73	68 up	Protein kinase
TKL-Pl-4	70	110	78	71	101	85	79	102	91	49	51	28 do	Protein kinase
TKL-Pl-5	27	37	22	11	16	35	15	21	24	16	17	15 up	Protein kinase
TKL-Pl-5	7	14	15	22	17	15	21	16	20	8	3	4 do	Protein kinase
TKL-Pl-6	19	19	21	21	11	30	13	22	25	16	11	10 up	Protein kinase
TKL-Pl-6	18	25	21	22	15	22	26	13	18	7	17	2 do	Protein kinase
TKL-Pl-7	5	2	7	4	3	5	2	0	4	8	4	4 up	Protein kinase
TKL-Pl-7	5	7	4	2	9	2	4	11	4	2	1	2 do	Protein kinase
TKL-Pl-8	0	2	0	0	4	0	1	0	0	0	0	0 up	Protein kinase
TKL-Pl-8	1	3	0	1	6	0	1	8	0	0	1	0 do	Protein kinase
TLK	4	11	3	6	4	9	2	3	4	7	3	10 up	Protein kinase
TLK	2	7	3	6	4	2	8	5	3	8	7	3 do	Protein kinase
TTK	10	5	14	13	8	17	1	1	7	23	25	7 up	Protein kinase
TTK	10	17	12	7	15	17	9	16	14	5	1	1 do	Protein kinase
ULK	6	4	2	3	2	10	1	0	6	14	10	2 up	Protein kinase
ULK	1	7	19	5	11	5	6	10	6	1	3	2 do	Protein kinase
WEE	0	0	0	0	0	0	0	0	0	0	1	0 up	Protein kinase
WEE	1	1	0	0	0	0	0	0	0	0	0	0 do	Protein kinase
WNK	34	42	40	52	27	61	28	153	55	24	15	19 up	Protein kinase
WNK	23	23	41	33	35	42	31	32	48	7	8	4 do	Protein kinase
AP2	107	94	58	67	56	74	46	45	54	87	58	45 up	Transcription factor
AP2	29	63	42	61	91	63	68	100	67	11	21	18 do	Transcription factor
ARF	154	159	213	253	131	237	116	86	218	198	179	96 up	Transcription factor
ARF	156	319	160	152	213	142	189	255	154	114	141	53 do	Transcription factor
ARR-B	70	50	92	76	60	99	56	28	49	74	101	61 up	Transcription factor
ARR-B	78	191	36	44	111	75	51	132	80	22	35	26 do	Transcription factor
B3	133	163	153	192	73	153	93	71	130	165	121	78 up	Transcription factor
B3	74	144	134	122	136	96	130	152	102	94	66	46 do	Transcription factor
BBR-BPC	21	30	26	18	7	14	11	8	13	17	12	8 up	Transcription factor
BBR-BPC	7	13	14	14	14	14	17	18	14	4	4	3 do	Transcription factor
BES1	26	45	41	36	17	42	20	17	25	25	28	21 up	Transcription factor
BES1	27	32	30	26	36	24	27	39	30	10	20	4 do	Transcription factor
bHLH	377	432	373	330	220	514	210	189	273	476	275	179 up	Transcription factor
bHLH	339	481	371	383	406	310	400	474	333	149	171	100 do	Transcription factor
bZIP	278	369	350	237	164	373	267	218	319	323	208	275 up	Transcription factor
bZIP	198	356	291	216	306	502	202	303	497	128	121	78 do	Transcription factor
C2H2	241	286	181	228	106	203	122	73	142	228	175	101 up	Transcription factor
C2H2	145	237	165	138	187	163	170	235	176	93	91	55 do	Transcription factor
C3H	263	302	267	206	152	238	165	157	220	245	189	250 up	Transcription factor
C3H	223	253	225	200	224	328	218	256	346	124	101	53 do	Transcription factor
CAMTA	66	104	93	66	67	82	74	67	93	92	81	56 up	Transcription factor
CAMTA	76	76	103	84	85	70	94	95	74	54	38	31 do	Transcription factor
CO-like	53	82	15	52	12	17	34	20	21	45	33	10 up	Transcription factor
CO-like	29	32	9	20	28	14	22	33	21	6	5	4 do	Transcription factor

CPP	15	16	32	18	7	27	25	5	26	38	27	10	up	Transcription factor
CPP	13	34	16	6	45	18	6	51	16	9	14	5	do	Transcription factor
DBB	89	65	27	67	13	22	21	9	12	56	26	21	up	Transcription factor
DBB	26	31	27	21	33	29	27	28	32	30	17	5	do	Transcription factor
Dof	56	69	79	59	34	90	56	15	43	56	51	46	up	Transcription factor
Dof	45	114	34	37	62	82	41	89	84	23	29	13	do	Transcription factor
E2F/DP	22	35	35	52	24	44	25	51	28	48	38	20	up	Transcription factor
E2F/DP	34	28	16	33	44	24	39	40	28	13	10	3	do	Transcription factor
EIL	17	25	15	7	4	13	14	17	15	8	6	13	up	Transcription factor
EIL	5	15	6	12	12	18	9	6	19	6	8	2	do	Transcription factor
ERF	112	178	183	119	68	173	123	80	102	141	95	74	up	Transcription factor
ERF	82	180	91	68	124	110	72	145	131	40	42	29	do	Transcription factor
FAR1	153	182	161	165	105	172	109	97	160	196	114	111	up	Transcription factor
FAR1	76	153	133	121	139	151	131	139	158	92	65	51	do	Transcription factor
G2-like	285	291	214	231	124	208	141	144	118	230	130	103	up	Transcription factor
G2-like	166	241	196	212	187	149	234	198	166	125	87	38	do	Transcription factor
GATA	86	89	69	97	48	88	45	22	56	102	73	30	up	Transcription factor
GATA	68	100	54	60	72	57	76	97	66	37	29	14	do	Transcription factor
GeBP	3	6	6	4	4	5	2	1	3	5	8	3	up	Transcription factor
GeBP	0	3	4	1	1	6	2	3	6	4	1	0	do	Transcription factor
GRAS	121	123	114	80	66	133	73	75	95	103	109	91	up	Transcription factor
GRAS	57	118	136	66	95	156	79	113	168	50	53	55	do	Transcription factor
GRF	38	23	12	34	16	20	19	2	19	42	30	22	up	Transcription factor
GRF	12	20	23	26	22	21	29	30	19	11	14	2	do	Transcription factor
HB-other	70	121	72	72	67	104	56	52	78	94	68	45	up	Transcription factor
HB-other	53	90	68	70	109	85	86	112	83	37	42	34	do	Transcription factor
HB-PHD	4	5	7	3	2	1	2	0	1	4	4	4	up	Transcription factor
HB-PHD	5	2	4	2	1	5	2	2	6	7	1	3	do	Transcription factor
HD-ZIP	158	161	121	164	89	162	128	67	126	202	171	98	up	Transcription factor
HD-ZIP	135	235	101	114	180	111	133	238	106	81	76	24	do	Transcription factor
HRT-like	1	1	1	3	1	2	2	0	0	5	0	0	up	Transcription factor
HRT-like	3	1	1	3	1	4	3	1	4	2	3	0	do	Transcription factor
HSF	88	102	81	101	59	108	76	73	97	58	43	124	up	Transcription factor
HSF	56	120	45	48	57	112	64	56	110	41	87	29	do	Transcription factor
LBD	17	26	33	16	5	40	13	3	17	16	16	11	up	Transcription factor
LBD	7	16	23	6	17	24	7	17	28	13	9	7	do	Transcription factor
LFY	0	0	0	0	0	0	0	0	1	0	0	0	up	Transcription factor
LFY	0	0	0	1	0	0	0	0	0	0	0	0	do	Transcription factor
LSD	12	22	11	23	4	6	5	3	2	13	7	5	up	Transcription factor
LSD	15	13	5	9	15	24	12	15	23	3	3	2	do	Transcription factor
M-type_MADS	7	9	5	20	7	19	17	5	10	13	7	10	up	Transcription factor
M-type_MADS	14	10	7	12	18	6	14	21	5	3	6	3	do	Transcription factor
MIKC_MADS	52	50	61	50	40	54	53	6	31	48	23	35	up	Transcription factor
MIKC_MADS	51	50	26	37	36	46	35	42	51	12	23	6	do	Transcription factor
MYB	172	184	171	184	91	198	154	161	172	211	167	131	up	Transcription factor
MYB	70	203	141	106	171	215	126	196	214	52	83	30	do	Transcription factor

MYB_related	315	365	260	311	238	325	251	191	224	405	218	212 up	Transcription factor
MYB_related	333	385	191	447	369	223	471	446	268	182	204	62 do	Transcription factor
NAC	132	217	203	144	101	231	203	330	237	140	110	169 up	Transcription factor
NAC	66	191	96	91	138	232	91	139	236	88	75	40 do	Transcription factor
NF-X1	5	3	6	11	3	10	4	14	10	4	5	4 up	Transcription factor
NF-X1	4	7	7	10	5	10	8	5	10	6	1	2 do	Transcription factor
NF-YA	44	57	84	52	47	82	60	43	80	60	24	49 up	Transcription factor
NF-YA	37	59	38	38	53	87	40	57	84	20	29	13 do	Transcription factor
NF-YB	12	15	9	13	4	7	13	4	9	17	14	7 up	Transcription factor
NF-YB	6	9	6	10	13	15	8	11	15	4	3	2 do	Transcription factor
NF-YC	36	33	31	30	18	28	30	42	25	33	17	24 up	Transcription factor
NF-YC	28	36	21	20	36	37	19	35	40	15	20	13 do	Transcription factor
Nin-like	62	41	78	41	38	83	19	44	128	65	23	49 up	Transcription factor
Nin-like	25	52	78	72	49	118	75	48	121	49	59	15 do	Transcription factor
RAV	6	12	13	5	1	20	10	3	1	8	7	0 up	Transcription factor
RAV	5	4	1	1	7	4	1	8	7	1	1	0 do	Transcription factor
S1Fa-like	2	1	2	0	3	3	1	0	0	3	3	1 up	Transcription factor
S1Fa-like	0	3	2	1	2	1	2	3	1	3	0	0 do	Transcription factor
SBP	99	132	95	86	65	114	91	40	100	105	94	59 up	Transcription factor
SBP	67	130	60	63	129	100	76	150	106	62	63	28 do	Transcription factor
SRS	8	12	12	9	0	12	7	2	6	2	9	2 up	Transcription factor
SRS	4	10	6	1	12	8	2	15	11	1	3	0 do	Transcription factor
STAT	1	4	4	6	5	13	2	0	3	4	1	1 up	Transcription factor
STAT	3	4	6	5	4	1	6	5	1	4	2	0 do	Transcription factor
TALE	113	185	99	105	82	84	78	76	96	113	92	36 up	Transcription factor
TALE	82	189	60	91	146	119	109	179	118	59	72	62 do	Transcription factor
TCP	66	56	31	74	28	62	29	24	32	71	44	29 up	Transcription factor
TCP	33	43	32	44	56	34	72	76	42	21	11	16 do	Transcription factor
Trihelix	120	118	82	107	41	88	61	52	67	97	97	48 up	Transcription factor
Trihelix	60	85	55	71	93	57	83	101	71	25	26	12 do	Transcription factor
VOZ	15	21	13	15	11	8	16	7	9	22	14	13 up	Transcription factor
VOZ	13	21	9	16	12	25	14	13	24	12	8	2 do	Transcription factor
Whirly	9	10	7	10	3	3	5	2	5	7	5	5 up	Transcription factor
Whirly	3	4	3	2	9	5	1	10	7	3	4	1 do	Transcription factor
WOX	10	11	5	14	4	10	4	0	3	8	12	1 up	Transcription factor
WOX	3	10	2	6	7	0	7	13	1	2	2	2 do	Transcription factor
WRKY	126	206	196	117	99	255	119	126	129	157	127	79 up	Transcription factor
WRKY	93	235	164	125	155	191	117	149	221	113	59	54 do	Transcription factor
YABBY	10	0	1	17	1	0	5	0	0	20	2	0 up	Transcription factor
YABBY	11	14	0	3	3	1	4	4	1	1	0	0 do	Transcription factor
ZF-HD	13	13	3	23	9	3	13	3	3	19	24	1 up	Transcription factor
ZF-HD	1	14	0	5	10	2	6	15	4	5	4	1 do	Transcription factor
ARID	56	66	52	57	28	44	31	31	40	71	47	36 up	Transcriptional regulator
ARID	38	87	49	22	50	34	27	53	37	34	22	21 do	Transcriptional regulator
AUX/IAA	100	89	73	104	49	76	68	30	69	88	98	48 up	Transcriptional regulator
AUX/IAA	73	165	81	43	139	53	45	184	58	42	55	13 do	Transcriptional regulator

Coactivator p15	10	13	13	6	4	6	4	1	7	5	9	2	up	Transcriptional regulator
Coactivator p15	3	6	5	4	9	9	4	11	9	3	3	2	do	Transcriptional regulator
GNAT	56	61	46	59	24	52	51	26	33	77	50	41	up	Transcriptional regulator
GNAT	44	72	28	61	68	58	63	75	62	27	17	5	do	Transcriptional regulator
HMG	26	18	12	25	9	25	25	8	15	34	29	16	up	Transcriptional regulator
HMG	26	21	7	19	22	14	23	31	15	7	11	5	do	Transcriptional regulator
IWS1	39	53	81	50	38	101	70	86	68	59	48	51	up	Transcriptional regulator
IWS1	32	78	51	40	60	65	46	59	67	32	27	16	do	Transcriptional regulator
Jumonji	91	96	78	98	69	74	67	113	109	74	63	55	up	Transcriptional regulator
Jumonji	49	111	62	71	95	86	73	105	87	57	47	37	do	Transcriptional regulator
LUG	60	61	37	61	35	47	35	34	43	58	54	38	up	Transcriptional regulator
LUG	28	32	44	34	49	26	45	57	34	34	29	8	do	Transcriptional regulator
MBF1	7	3	2	1	0	0	3	2	0	2	3	4	up	Transcriptional regulator
MBF1	0	2	3	3	1	2	2	1	2	1	1	0	do	Transcriptional regulator
MED6	2	1	2	1	2	2	0	1	3	2	1	1	up	Transcriptional regulator
MED6	1	2	3	0	0	3	0	0	3	1	2	3	do	Transcriptional regulator
MED7	3	1	1	2	1	0	0	0	2	0	1	1	up	Transcriptional regulator
MED7	0	4	0	0	2	0	0	2	0	0	0	0	do	Transcriptional regulator
mTERF	163	143	95	175	79	118	70	48	74	178	101	51	up	Transcriptional regulator
mTERF	65	93	67	96	83	61	117	104	63	61	49	27	do	Transcriptional regulator
Others	355	409	256	291	137	266	239	187	255	322	203	166	up	Transcriptional regulator
Others	217	282	247	270	303	258	287	280	268	133	132	80	do	Transcriptional regulator
PHD	232	234	219	254	156	246	150	211	220	282	184	164	up	Transcriptional regulator
PHD	134	189	204	178	197	248	200	193	246	119	113	51	do	Transcriptional regulator
Pseudo ARR-B	24	25	8	26	4	9	15	11	11	32	28	8	up	Transcriptional regulator
Pseudo ARR-B	0	3	4	4	8	15	8	10	17	3	6	3	do	Transcriptional regulator
RB	4	5	9	5	5	6	4	9	7	12	5	8	up	Transcriptional regulator
RB	5	10	5	7	13	9	7	12	9	4	9	2	do	Transcriptional regulator
Rcd1-like	2	0	3	0	0	2	1	0	1	1	0	1	up	Transcriptional regulator
Rcd1-like	0	5	0	0	3	0	0	3	1	0	0	0	do	Transcriptional regulator
SET	152	166	141	176	101	150	92	53	131	177	126	92	up	Transcriptional regulator
SET	79	140	135	71	142	157	76	162	164	82	80	27	do	Transcriptional regulator
SNF2	250	305	297	273	181	344	180	166	299	349	265	199	up	Transcriptional regulator
SNF2	156	235	238	285	293	205	297	300	212	127	138	75	do	Transcriptional regulator
SOH1	7	15	10	19	6	12	9	12	8	14	6	5	up	Transcriptional regulator
SOH1	3	8	7	15	10	10	14	9	10	5	6	5	do	Transcriptional regulator
SWI/SNF-BAF60	48	47	42	40	33	48	37	43	57	69	42	41	up	Transcriptional regulator
SWI/SNF-BAF60	30	55	29	32	76	49	46	75	52	26	17	11	do	Transcriptional regulator
SWI/SNF-SWI3	36	39	28	36	24	33	19	21	34	27	15	22	up	Transcriptional regulator
SWI/SNF-SWI3	9	30	28	32	34	30	33	29	27	14	7	6	do	Transcriptional regulator
TAZ	53	67	60	34	17	58	54	57	46	61	33	28	up	Transcriptional regulator
TAZ	23	47	46	28	46	52	26	33	54	21	23	12	do	Transcriptional regulator
TRAF	66	104	74	61	35	97	59	71	74	81	69	38	up	Transcriptional regulator
TRAF	54	97	58	62	113	83	71	95	86	51	23	20	do	Transcriptional regulator

Supplementary Table 8. Number of nonsense-mediated mRNA decay (NMD) events and their distribution by exons.

Germplasm	Condition	Tissue	NMD1	NMD2	NMD3	NMD4	NMD5	NMD6	NMD7	NMD8	NMD9	NND +10	OK
PI467895	SS	Leaf	7,069	5,234	4,199	3,399	2,845	2,347	1,781	1,460	1,161	3,946	18,376
PI467895	SS	Leaf	7,069	5,234	4,199	3,399	2,845	2,347	1,781	1,460	1,161	3,946	18,376
PI467895	SS	Stem	13,038	9,499	7,422	6,034	4,849	4,117	3,248	2,563	1,960	7,591	33,124
PI467895	SS	Root	13,771	9,990	7,857	6,292	5,244	4,507	3,448	2,844	2,230	9,089	35,555
PI467895	CK	Leaf	8,615	6,477	5,002	4,160	3,355	2,891	2,188	1,808	1,434	4,949	22,518
PI467895	CK	Stem	14,079	10,430	7,976	6,536	5,379	4,593	3,526	2,814	2,288	8,740	37,776
PI467895	CK	Root	11,722	8,590	6,617	5,361	4,452	3,727	3,063	2,257	1,868	7,569	31,875
Saranac	DS	Leaf	9,285	6,508	5,122	4,231	3,379	2,844	2,253	1,808	1,448	5,331	23,676
Saranac	DS	Stem	7,922	5,721	4,447	3,939	2,921	2,540	2,120	1,696	1,396	5,465	20,869
Saranac	DS	Root	10,401	7,600	6,000	4,901	3,931	3,286	2,637	2,167	1,794	7,117	26,860
Saranac	SS	Leaf	12,311	9,337	7,190	6,001	4,924	4,195	3,310	2,700	2,216	8,268	32,895
Saranac	SS	Stem	9,550	7,085	5,407	4,437	3,703	3,135	2,416	1,934	1,616	5,965	24,484
Saranac	SS	Root	12,714	9,161	7,139	5,755	4,698	4,071	3,182	2,469	2,021	7,879	33,988
Saranac	CK	Leaf	9,779	7,029	5,521	4,678	3,801	3,201	2,606	2,134	1,788	6,593	24,306
Saranac	CK	Stem	12,758	9,370	7,262	5,889	4,664	4,006	3,009	2,273	2,021	6,966	34,561
Saranac	CK	Root	11,186	8,047	6,331	5,282	4,164	3,486	2,827	2,255	1,884	7,860	29,880
Wilson	DS	Leaf	11,818	8,659	6,864	5,615	4,550	4,018	3,016	2,417	2,002	7,663	32,251
Wilson	DS	Stem	9,129	6,737	5,194	4,201	3,357	2,843	2,267	1,724	1,452	5,551	25,249
Wilson	DS	Root	6,728	4,759	3,767	3,107	2,572	1,997	1,677	1,323	1,120	4,449	17,207
Wilson	CK	Leaf	6,742	4,926	3,792	3,145	2,516	2,254	1,730	1,424	1,173	4,450	17,138
Wilson	CK	Stem	7,001	5,043	3,872	3,234	2,727	2,199	1,756	1,429	1,199	4,469	17,894
Wilson	CK	Root	3,872	2,722	2,183	1,758	1,475	1,242	1,090	840	710	2,746	10,503

OK, no-NMD; DS, drought stress; SS, salt stress; CK control

Supplementary Table 9. Count of genes and isoforms predicted as lncRNA and circRNAs by treatment

**Treatment**	**count_gene_lncRNAs**	**count_isoform_lncRNAs**	**count_circRNAs**
PI467895-CK-Stem	607	976	186
Saranac-DS-Stem	371	646	189
Saranac-SS-Leaf	647	1,007	200
PI467895-CK-Leaf	436	720	88
PI467895-SS-Stem	676	1,114	125
PI467895-SS-Leaf	390	663	79
Saranac-DS-Leaf	471	715	113
Saranac-DS-Root	167	204	260
Saranac-CK-Leaf	535	897	94
Saranac-CK-Root	511	730	132
Saranac-CK-Stem	598	984	171
Wilson-DS-Leaf	659	1,076	75
Wilson-DS-Stem	356	500	69
PI467895-CK-Root	551	928	175
PI467895-SS-Root	546	852	164
Saranac-SS-Root	489	774	172
Saranac-SS-Stem	471	728	144
Wilson-DS-Root	313	425	34
Wilson-CK-Leaf	328	530	49
Wilson-CK-Stem	346	496	61
Wilson-CK-Root	143	214	56
Total	9,611	15,179	2,636

Supplementary Table 10. Classification of lncRNA predicted according to SQANTI3 structural classification.

Germplasm	Condition	Tissue	Antisense	FSM	Fusion	Genic	ISM	Intergenic	NIC	NNIC
PI467895	SS	Leaf	12	15	2	7	59	330	38	99
PI467895	SS	Stem	43	45	2	15	100	636	47	128
PI467895	SS	Root	33	30	1	10	47	536	49	88
PI467895	CK	Leaf	18	28	2	4	57	354	38	91
PI467895	CK	Stem	34	57	2	8	62	509	58	117
PI467895	CK	Root	45	56	4	6	59	494	37	128
Saranac	DS	Leaf	14	58	2	10	68	317	46	83
Saranac	DS	Stem	26	38	1	3	67	278	51	96
Saranac	DS	Root	5	6	0	2	16	131	5	29
Saranac	SS	Leaf	30	64	6	19	86	521	58	118
Saranac	SS	Stem	16	37	3	7	53	374	40	92
Saranac	SS	Root	19	42	5	5	69	460	44	99
Saranac	CK	Leaf	31	38	6	14	77	467	61	136
Saranac	CK	Stem	11	79	3	16	93	411	43	124
Saranac	CK	Root	22	27	2	8	66	441	41	94
Wilson	DS	Leaf	19	63	3	12	70	592	72	113
Wilson	DS	Stem	6	51	4	6	32	288	19	49
Wilson	DS	Root	3	22	3	5	28	262	25	50
Wilson	CK	Leaf	7	23	2	3	44	318	18	58
Wilson	CK	Stem	12	19	5	4	39	304	27	54
Wilson	CK	Root	4	14	0	2	20	103	6	15

FSM, Full Splice Match; ISM, Incomplete Splice Match; NIC, Novel In Catalog; NNIC, Novel Not In Catalog; DS, drought stress; SS, salt stress; CK control

Supplementary Table 11. Gene onotology enrichment terms related with host genes in circRNAS and adjacent genes in lncRNAs

GO.ID	Term	Annotated	Significant	Expected	raw.p.value	p.adj	GO.Cat	ncRNA
GO:1902493	acetyltransferase complex	9	0	0.15	0.03205	0.0354	CC	circRNA
GO:0003779	actin binding	15	0	0.29	0.0124	0.0134	MF	circRNA
GO:0048046	apoplast	29	0	0.48	0.0021	0.0058	CC	circRNA
GO:0003824	catalytic activity	1832	38	35.27	5.50E-07	0	MF	circRNA
GO:1902494	catalytic complex	30	0	0.49	0.03366	0.0354	CC	circRNA
GO:0071944	cell periphery	29	0	0.48	0.01343	0.0269	CC	circRNA
GO:0007623	circadian rhythm	6	0	0.17	0.00273	0.0039	BP	circRNA
GO:0015036	disulfide oxidoreductase activity	19	0	0.37	0.0046	0.0077	MF	circRNA
GO:0004857	enzyme inhibitor activity	44	0	0.85	0.0104	0.0134	MF	circRNA
GO:0007186	G protein-coupled receptor signaling pathway	7	0	0.19	0.01499	0.0181	BP	circRNA
GO:0040007	growth	25	0	0.69	0.01625	0.0181	BP	circRNA
GO:0008083	growth factor activity	3	0	0.06	0.0134	0.0134	MF	circRNA
GO:0000123	histone acetyltransferase complex	9	0	0.15	0.03205	0.0354	CC	circRNA
GO:0016788	hydrolase activity, acting on ester bonds	169	1	3.25	0.0073	0.0112	MF	circRNA
GO:0010274	hydrotropism	5	0	0.14	0.00606	0.0081	BP	circRNA
GO:0005622	intracellular	585	12	9.59	0.00085	0.0034	CC	circRNA
GO:0043231	intracellular membrane-bounded organelle	498	10	8.17	0.0003	0.0021	CC	circRNA
GO:0043232	intracellular non-membrane-bounded organelle	34	1	0.56	0.03345	0.0354	CC	circRNA
GO:0043229	intracellular organelle	521	11	8.54	0.00042	0.0021	CC	circRNA
GO:0070013	intracellular organelle lumen	36	0	0.59	0.02993	0.0354	CC	circRNA
GO:0043227	membrane-bounded organelle	506	11	8.3	0.0003	0.0021	CC	circRNA
GO:0031974	membrane-enclosed lumen	36	0	0.59	0.02993	0.0354	CC	circRNA
GO:0003729	mRNA binding	9	0	0.17	0.0025	0.0058	MF	circRNA
GO:0043228	non-membrane-bounded organelle	34	1	0.56	0.03345	0.0354	CC	circRNA
GO:0005634	nucleus	380	9	6.23	0.00501	0.0111	CC	circRNA
GO:0043226	organelle	529	12	8.68	0.00042	0.0021	CC	circRNA
GO:0043233	organelle lumen	36	0	0.59	0.02993	0.0354	CC	circRNA
GO:1901564	organonitrogen compound metabolic process	111	3	3.06	0.0212	0.0223	BP	circRNA
GO:0010088	phloem development	13	0	0.36	0.00042	0.0014	BP	circRNA
GO:0010087	phloem or xylem histogenesis	16	0	0.44	0.00059	0.0017	BP	circRNA
GO:0042578	phosphoric ester hydrolase activity	67	0	1.29	0.0023	0.0058	MF	circRNA
GO:0005886	plasma membrane	26	0	0.43	0.0023	0.0058	CC	circRNA
GO:0031248	protein acetyltransferase complex	9	0	0.15	0.03205	0.0354	CC	circRNA
GO:0015035	protein disulfide oxidoreductase activity	19	0	0.37	0.0046	0.0077	MF	circRNA
GO:0032446	protein modification by small protein conjugation	62	2	1.71	0.00124	0.0025	BP	circRNA
GO:0070647	protein modification by small protein conjugation or remc	62	2	1.71	0.00124	0.0025	BP	circRNA
GO:0016567	protein ubiquitination	60	2	1.65	0.00111	0.0025	BP	circRNA

GO:0048018	receptor ligand activity	3	0	0.06	0.0134	0.0134	MF	circRNA
GO:0030545	receptor regulator activity	3	0	0.06	0.0134	0.0134	MF	circRNA
GO:0042752	regulation of circadian rhythm	6	0	0.17	0.00273	0.0039	BP	circRNA
GO:0040008	regulation of growth	25	0	0.69	0.01625	0.0181	BP	circRNA
GO:0009733	response to auxin	60	0	1.65	3.00E-07	0	BP	circRNA
GO:0042221	response to chemical	83	1	2.28	3.00E-06	0	BP	circRNA
GO:0009719	response to endogenous stimulus	69	0	1.9	5.30E-06	0	BP	circRNA
GO:0009725	response to hormone	69	0	1.9	5.30E-06	0	BP	circRNA
GO:0010033	response to organic substance	72	0	1.98	9.80E-07	0	BP	circRNA
GO:0050896	response to stimulus	201	2	5.53	0.02588	0.0259	BP	circRNA
GO:0048511	rhythmic process	6	0	0.17	0.00273	0.0039	BP	circRNA
GO:0000124	SAGA complex	2	0	0.03	0.03571	0.0357	CC	circRNA
GO:0070461	SAGA-type complex	5	0	0.08	0.00182	0.0058	CC	circRNA
GO:0030546	signaling receptor activator activity	3	0	0.06	0.0134	0.0134	MF	circRNA
GO:0005102	signaling receptor binding	3	0	0.06	0.0134	0.0134	MF	circRNA
GO:0009888	tissue development	18	0	0.5	0.00169	0.0031	BP	circRNA
GO:0016740	transferase activity	650	14	12.52	6.60E-07	0	MF	circRNA
GO:0016746	transferase activity, transferring acyl groups	163	4	3.14	7.30E-05	2.00E-04	MF	circRNA
GO:0016747	transferase activity, transferring acyl groups other than an	145	3	2.79	1.00E-05	0	MF	circRNA
GO:0016757	transferase activity, transferring glycosyl groups	221	6	4.26	9.50E-08	0	MF	circRNA
GO:0019787	ubiquitin-like protein transferase activity	65	0	1.25	0.0029	0.0058	MF	circRNA
GO:0004842	ubiquitin-protein transferase activity	65	0	1.25	0.0029	0.0058	MF	circRNA
GO:0008194	UDP-glycosyltransferase activity	189	2	3.64	4.50E-09	0	MF	circRNA
GO:0016407	acetyltransferase activity	32	2	3.16	0.0107	0.0107	MF	lncRNA
GO:0048046	apoplast	29	3	2.98	0.00163	0.0054	CC	lncRNA
GO:0003824	catalytic activity	1832	166	180.92	1.10E-07	0	MF	lncRNA
GO:0006575	cellular modified amino acid metabolic process	23	1	2.26	0.0263	0.0263	BP	lncRNA
GO:0007623	circadian rhythm	6	1	0.59	0.0235	0.0263	BP	lncRNA
GO:0015036	disulfide oxidoreductase activity	19	0	1.88	0.00077	0.0022	MF	lncRNA
GO:0004866	endopeptidase inhibitor activity	28	0	2.77	0.00667	0.0089	MF	lncRNA
GO:0061135	endopeptidase regulator activity	28	0	2.77	0.00667	0.0089	MF	lncRNA
GO:0004857	enzyme inhibitor activity	44	1	4.35	0.00191	0.0042	MF	lncRNA
GO:0005576	extracellular region	65	4	6.69	0.01132	0.0283	CC	lncRNA
GO:0007186	G protein-coupled receptor signaling pathway	7	0	0.69	0.0141	0.0217	BP	lncRNA
GO:0006749	glutathione metabolic process	23	1	2.26	0.0263	0.0263	BP	lncRNA
GO:0016788	hydrolase activity, acting on ester bonds	169	12	16.69	0.00907	0.0107	MF	lncRNA
GO:0010274	hydrotropism	5	0	0.49	0.0056	0.0093	BP	lncRNA
GO:0005622	intracellular	585	66	60.21	0.00115	0.0046	CC	lncRNA
GO:0043231	intracellular membrane-bounded organelle	498	54	51.26	0.00033	0.0016	CC	lncRNA
GO:0043232	intracellular non-membrane-bounded organelle	34	3	3.5	0.02741	0.0422	CC	lncRNA

GO:0043229	intracellular organelle	521	56	53.63	0.00027	0.0016	CC	lncRNA
GO:0098796	membrane protein complex	9	0	0.93	0.0222	0.0422	CC	lncRNA
GO:0043227	membrane-bounded organelle	506	55	52.08	0.00027	0.0016	CC	lncRNA
GO:0098798	mitochondrial protein complex	4	0	0.41	0.02423	0.0422	CC	lncRNA
GO:0098772	molecular function regulator	77	4	7.6	0.00739	0.0092	MF	lncRNA
GO:0008080	N-acetyltransferase activity	32	2	3.16	0.0107	0.0107	MF	lncRNA
GO:0016410	N-acyltransferase activity	32	2	3.16	0.0107	0.0107	MF	lncRNA
GO:0005854	nascent polypeptide-associated complex	6	0	0.62	0.02454	0.0422	CC	lncRNA
GO:0043228	non-membrane-bounded organelle	34	3	3.5	0.02741	0.0422	CC	lncRNA
GO:0005634	nucleus	380	42	39.11	0.00312	0.0089	CC	lncRNA
GO:0043226	organelle	529	57	54.45	0.00022	0.0016	CC	lncRNA
GO:0016667	oxidoreductase activity, acting on a sulfur group of donor	24	0	2.37	0.00268	0.0054	MF	lncRNA
GO:0030414	peptidase inhibitor activity	28	0	2.77	0.00667	0.0089	MF	lncRNA
GO:0061134	peptidase regulator activity	28	0	2.77	0.00667	0.0089	MF	lncRNA
GO:0006518	peptide metabolic process	23	1	2.26	0.0263	0.0263	BP	lncRNA
GO:0010088	phloem development	13	1	1.28	0.0017	0.0049	BP	lncRNA
GO:0010087	phloem or xylem histogenesis	16	1	1.57	0.0017	0.0049	BP	lncRNA
GO:0042578	phosphoric ester hydrolase activity	67	6	6.62	0.00442	0.008	MF	lncRNA
GO:0015035	protein disulfide oxidoreductase activity	19	0	1.88	0.00077	0.0022	MF	lncRNA
GO:0032446	protein modification by small protein conjugation	62	6	6.09	0.0052	0.0093	BP	lncRNA
GO:0070647	protein modification by small protein conjugation or remo	62	6	6.09	0.0052	0.0093	BP	lncRNA
GO:0016567	protein ubiquitination	60	6	5.9	0.0051	0.0093	BP	lncRNA
GO:0042752	regulation of circadian rhythm	6	1	0.59	0.0235	0.0263	BP	lncRNA
GO:0009733	response to auxin	60	3	5.9	9.70E-07	0	BP	lncRNA
GO:0042221	response to chemical	83	4	8.16	7.90E-06	0	BP	lncRNA
GO:0009719	response to endogenous stimulus	69	3	6.78	9.50E-06	0	BP	lncRNA
GO:0009725	response to hormone	69	3	6.78	9.50E-06	0	BP	lncRNA
GO:0010033	response to organic substance	72	3	7.08	1.60E-06	0	BP	lncRNA
GO:0050896	response to stimulus	201	21	19.76	0.0199	0.0263	BP	lncRNA
GO:0048511	rhythmic process	6	1	0.59	0.0235	0.0263	BP	lncRNA
GO:0009888	tissue development	18	1	1.77	0.0035	0.0088	BP	lncRNA
GO:0016740	transferase activity	650	68	64.19	9.20E-06	0	MF	lncRNA
GO:0016746	transferase activity, transferring acyl groups	163	20	16.1	0.00144	0.0036	MF	lncRNA
GO:0016747	transferase activity, transferring acyl groups other than an	145	18	14.32	0.0003	0.0012	MF	lncRNA
GO:0016757	transferase activity, transferring glycosyl groups	221	18	21.83	1.90E-08	0	MF	lncRNA
GO:0008194	UDP-glycosyltransferase activity	189	16	18.67	2.20E-08	0	MF	lncRNA

Supplementary Table 12. Count of isoforms differentially expressed by germplasm under salt and drought stress. Isoforms were classified as downregulated (down) and upregulated (up) according to z_score showed in Supplementary Figures 3 to 5.

Germplasm	Salt_stress	Drought_stress	Count
PI467895	Down	NA	46
PI467895	Up	NA	61
Wilson	NA	Down	62
Wilson	NA	Up	73
Saranac	Down	Down	13
Saranac	Down	Up	41
Saranac	Up	Down	28
Saranac	Up	Up	8

Supplementary Table 13. Differential expressed isoforms in PI467895, Wilson and Saranac under salt or drought stress. Isoforms were classified as downregulated(down) and upregulated (up) according to z_score showed in Supplementary Figure 3 to 5. Isoforms were annotated using different databases: *Medicago truncatula* database (Mt), transcription factor from PlantRegMap (TF), protein kinase from iTAK (PK), lncRNA, circRNA, Uniprot ID and protein name.

Germplasm	isoform	Salt_stress	Drought_stress	Mt	TF	family_iTAK	class_iTAK	PK_iTAK	lncRNA	circType	Uniprot	Prot_names
PI467895	G28604.4	Down	NA	Chr3g0110451	NA	NA	NA	NA	NA	NA	UPI0007AFA08F	uncharacterized protein LOC107625446;uncharacterized protein LOC112727602
PI467895	G83616.1	Down	NA	NA	NA	NA	NA	NA	NA	NA	NA	NA
PI467895	G26824.3	Down	NA	NA	NA	NA	NA	NA	NA	NA	NA	NA
PI467895	G84600.1	Down	NA	Chr1g0176241	NA	NA	NA	NA	NA	NA	K7MC23	Uncharacterized protein
PI467895	G83160.4	Down	NA	Chr5g0407721	NA	NA	NA	RLK-Pelle_L-LEC	NA	NA	UPI000B7B0920	L-type lectin-domain containing receptor kinase S.1
PI467895	G24387.7	Down	NA	Chr3g0126031	NA	NA	NA	NA	NA	NA	W9SF22	Carboxypeptidase (EC 3.4.16.-)
PI467895	G633.4	Down	NA	Chr5g0417781	NA	NA	NA	NA	NA	NA	UPI0007AF47D0	uncharacterized endoplasmic reticulum membrane protein C16E8.02
PI467895	G91254.1	Down	NA	Chr8g0336791	NA	NA	NA	NA	NA	NA	UPI00092E75A6	1-aminocyclopropane-1-carboxylate oxidase homolog 1-like
PI467895	G69537.18	Down	NA	Chr4g0008691	NA	NA	NA	NA	NA	NA	A0A4D6MS04	Uncharacterized protein
PI467895	G64977.4	Down	NA	Chr6g0485801	NA	NA	NA	NA	NA	NA	A0A371ETE1	tRNA(Ile)-2-lysyl-cytidine synthase (EC 6.3.4.19) (tRNA(Ile)-lysidine synthetase) (Fragment)
PI467895	G36295.121	Down	NA	Chr3g0105491	NA	NA	NA	NA	NA	NA	A0A1S3E6A5	uncharacterized protein LOC101508676 isoform X1
PI467895	G13425.78	Down	NA	Chr1g0198981	NA	NA	NA	NA	NA	NA	A0A218WCI6	RING-type E3 ubiquitin transferase (EC 2.3.2.27)
PI467895	G78234.87	Down	NA	Chr8g0356891	NA	NA	NA	NA	NA	NA	V7B446	Uncharacterized protein
PI467895	G46292.1	Down	NA	Chr2g0332901	NA	NA	NA	NA	NA	NA	A0A0S3QYL8	Uncharacterized protein
PI467895	G88117.4	Down	NA	Chr7g0235291	ERF	AP2/ERF-ERF	TF	NA	NA	NA	UPI000DECB523	ethylene-responsive transcription factor-like protein At4g13040 isoform X2
PI467895	G28526.5	Down	NA	Chr8g0367201	NA	NA	NA	NA	NA	NA	A0A0L9UGG2	1-deoxy-D-xylulose-5-phosphate synthase (EC 2.2.1.7)
PI467895	G11062.20	Down	NA	Chr2g0303971	NA	NA	NA	NA	NA	NA	N0DLJ8	Phytochrome
PI467895	G39330.3	Down	NA	Chr8g0389371	NA	NA	NA	NA	NA	NA	M5X2S6	Uncharacterized protein
PI467895	G77404.61	Down	NA	Chr5g0415871	NA	NA	NA	NA	NA	NA	UPI000786D0F0	inhibitor of Bruton tyrosine kinase
PI467895	G44607.5	Down	NA	Chr4g0009441	NA	NA	NA	NA	NA	NA	A0A445E1R0	Uncharacterized protein
PI467895	G81412.3	Down	NA	Chr8g0352251	NF-YA	NA	NA	NA	NA	NA	UPI000A2B6941	nuclear transcription factor Y subunit A-1 isoform X2;nuclear transcription factor Y subunit A-1-like isoform X2
PI467895	G66279.52	Down	NA	Chr8g0338741	NA	NA	NA	AGC_MAST	NA	NA	UPI0010A2B78E	probable serine/threonine protein kinase IREH1 isoform X2
PI467895	G2630.63	Down	NA	Chr1g0155031	NA	NA	NA	NA	NA	NA	A0A396JQU4	Uncharacterized protein
PI467895	G45674.6	Down	NA	Chr3g0095271	NA	NA	NA	NA	NA	NA	A0A0B2SGN1	Uncharacterized protein
PI467895	G35124.1	Down	NA	Chr4g0070961	NA	NA	NA	NA	NA	NA	A0A392N172	Guanine nucleotide-binding protein subunit beta-2-like (Fragment)
PI467895	G59102.1	Down	NA	Chr7g0255781	NA	NA	NA	NA	NA	NA	V7BMA8	Uncharacterized protein
PI467895	G43481.20	Down	NA	Chr1g0180291	NA	NA	NA	NA	NA	NA	UPI0010A2DC1B	rac-like GTP-binding protein RAC1 isoform X1
PI467895	G22771.3	Down	NA	Chr4g0075301	NA	NA	NA	NA	NA	NA	UPI00046E293F	CSC1-like protein ERD4
PI467895	G49920.13	Down	NA	Chr1g0206791	NA	NA	NA	NA	NA	NA	UPI000C9D4DA0	pyruvate kinase cytosolic isozyme-like
PI467895	G34256.3	Down	NA	Chr1g0170951	NA	NA	NA	NA	NA	NA	A0A5D2QWH2	Methyltransf_2 domain-containing protein
PI467895	G9771.1	Down	NA	Chr5g0410081	NA	NA	NA	NA	NA	NA	UPI00092F9C50	extra-large guanine nucleotide-binding protein 1-like
PI467895	G13446.5	Down	NA	Chr4g0039211	NA	NA	NA	NA	NA	NA	I1LPC1	Uncharacterized protein
PI467895	G90305.18	Down	NA	Chr8g0359641	NA	NA	NA	RLK-Pelle_DLSV	NA	NA	UPI000DECC7A7	cold-responsive protein kinase 1
PI467895	G89378.38	Down	NA	Chr4g0059861	NA	NA	NA	NA	NA	NA	UPI0007192CDB	HIPL1 protein
PI467895	G80639.8	Down	NA	Chr8g0370031	NA	NA	NA	NA	NA	NA	D7TPA5	Uncharacterized protein
PI467895	G30557.17	Down	NA	Chr3g0118821	NA	NA	NA	NA	NA	NA	A0A4D6KV57	Mitochondrial PGP phosphatase
PI467895	G65492.50	Down	NA	Chr3g0114341	NA	NA	NA	NA	NA	NA	A0A0B2NZS7	Amino acid transporter AVT6B isoform A (Amino acid transporter AVT6B isoform B) (Sodium-coupled neutral amino acid transporter 1)
PI467895	G29830.4	Down	NA	Chr8g0361471	NA	NA	NA	NA	NA	NA	A0A445JNI0	Cytochrome P450 71D11
PI467895	G55257.11	Down	NA	Chr8g0372721	NA	NA	NA	NA	NA	NA	A0A1Q3DEN4	UPF0261 domain-containing protein/TIM-br_sig_trns domain-containing protein
PI467895	G14319.3	Down	NA	Chr6g0480081	NA	NA	NA	NA	NA	NA	M5W736	SWIM-type domain-containing protein
PI467895	G69558.45	Down	NA	Chr3g0142381	NA	NA	NA	NA	NA	NA	A0A444ZNF5	Uncharacterized protein
PI467895	G11515.4	Down	NA	Chr4g0062991	NA	NA	NA	NA	NA	NA	A0A2N9ELM9	TPT domain-containing protein
PI467895	G45634.1	Down	NA	Chr5g0396911	ERF	AP2/ERF-ERF	TF	NA	NA	NA	UPI0007AF0235	ethylene-responsive transcription factor ERF060
PI467895	G74120.13	Down	NA	Chr7g0254797	NA	NA	NA	NA	NA	NA	A0A2U1LGA0	Non-reducing end alpha-L-arabinofuranosidase (EC 3.2.1.55)
PI467895	G5683.6	Down	NA	Chr1g0157381	NA	NA	NA	NA	NA	NA	A0A4D6MD78	Ubiquilin
PI467895	G84704.2	Down	NA	Chr5g0413681	NA	NA	NA	NA	NA	NA	UPI000F7CB2EF	U-box domain-containing protein 43-like isoform X3
PI467895	G62476.26	Up	NA	Chr8g0372721	NA	NA	NA	NA	NA	NA	V7CGM7	Uncharacterized protein
PI467895	G6446.30	Up	NA	Chr3g0137841	NA	NA	NA	NA	NA	NA	A0A4D6LLQ2	RNA-dependent RNA polymerase (EC 2.7.7.48)
PI467895	G17789.18	Up	NA	Chr4g0072741	NA	NA	NA	TKL-Pl-4	NA	NA	A0A445DR24	Protein kinase domain-containing protein
PI467895	G7039.8	Up	NA	Chr7g0228321	NA	NA	NA	NA	NA	NA	UPI000511162D	probable CCR4-associated factor 1 homolog 11 isoform X3;probable CCR4-associated factor 1 homolog 11 isoform X1;probable CCR4-associated factor 1 homolog 11 isoform X2
PI467895	G78142.16	Up	NA	Chr5g0408941	NA	NA	NA	RLK-Pelle_RLCK-\	NA	NA	V7AP55	Protein kinase domain-containing protein
PI467895	G89692.66	Up	NA	Chr8g0337661	NA	NA	NA	RLK-Pelle_CrRLK1	NA	NA	A0A4P1QR97	FRIGIDA-like protein
PI467895	G12587.7	Up	NA	Chr3g0138821	NA	NA	NA	NA	NA	NA	A0A0L9UG79	zinc_ribbon_10 domain-containing protein
PI467895	G73578.48	Up	NA	Chr7g0272581	NA	NA	NA	NA	NA	NA	K7KGN2	Uncharacterized protein
PI467895	G74437.3	Up	NA	Chr7g0275431	NA	NA	NA	NA	NA	NA	A0A2H3YGM6	Tubulin beta chain
PI467895	G81745.1	Up	NA	Chr2g0306111	NA	NA	NA	NA	NA	NA	A0A072TEN3	Uncharacterized protein (Fragment)
PI467895	G69600.7	Up	NA	Chr7g0233061	MYB	MYB	TF	NA	NA	NA	A0A368UJZ0	Uncharacterized protein
PI467895	G38336.8	Up	NA	Chr8g0391761	NA	NA	NA	NA	NA	NA	UPI00103660EA	40S ribosomal protein S2-4-like
PI467895	G22878.2	Up	NA	Chr2g0283151	NA	NA	NA	NA	NA	NA	A0A2N9HPA3	1-(5-phosphoribosyl)-5-[(5-phosphoribosylamino)methylideneamino] imidazole-4-carboxamide isomerase chloroplastic (EC 5.3.1.16) (5-proFAR isomerase) (Phosphoribosylformimino-5-aminoimidazole carboxamide ribotide isomerase)
PI467895	G9722.2	Up	NA	Chr4g0061811	NA	NA	NA	NA	NA	NA	V7C7K3	RING-type domain-containing protein
PI467895	G30609.17	Up	NA	Chr3g0120111	NA	NA	NA	NA	NA	NA	K7LYA2	N2227 domain-containing protein
PI467895	G5461.10	Up	NA	Chr6g0484701	NA	NA	NA	NA	NA	NA	A0A3P6BMI1	Uncharacterized protein
PI467895	G64546.12	Up	NA	Chr8g0354501	NA	NA	NA	NA	NA	NA	UPI0007F031F1	pre-mRNA-splicing factor ATP-dependent RNA helicase DEAH1-like
PI467895	G72781.4	Up	NA	Chr7g0258101	NA	NA	NA	NA	NA	NA	A0A4D6M5V6	C2 NT-type domain-containing protein
PI467895	G36936.24	Up	NA	Chr5g0442041	NA	NA	NA	NA	NA	NA	UPI001245AEE5	Uncharacterized protein;GPN-loop GTPase 3-like
PI467895	G27486.7	Up	NA	Chr8g0379181	NA	NA	NA	NA	NA	NA	G7JNU5	Nucleobase-ascorbate transporter-like protein
PI467895	G20310.38	Up	NA	Chr2g0282531	NA	NA	NA	NA	NA	NA	A0A445BBJ6	Uncharacterized protein
PI467895	G86136.167	Up	NA	Chr4g0020751	NA	NA	NA	NA	NA	NA	UPI00103DAE9A	protein JINGUBANG-like isoform X1
PI467895	G41263.8	Up	NA	Chr4g0005721	NA	NA	NA	NA	NA	NA	UPI001035CD8E	OTU domain-containing protein At3g57810 isoform X1
PI467895	G2879.10	Up	NA	Chr8g0390721	NA	NA	NA	NA	NA	NA	A0A2P5DMN9	Enoyl-CoA hydratase/isomerase HIBYL-CoA-H type
PI467895	G71953.2	Up	NA	NA	NA	NA	NA	NA	NA	NA	NA	NA
PI467895	G398.13	Up	NA	Chr4g0005181	NA	NA	NA	NA	NA	NA	UPI0007AEF6D3	coatomer subunit beta'-2 isoform X1
PI467895	G50153.26	Up	NA	Chr4g0038901	NA	NA	NA	NA	NA	NA	A0A371EEH3	Beta-adaptin-like protein C (Fragment)
PI467895	G24825.6	Up	NA	Chr3g0130341	NA	NA	NA	NA	NA	NA	A0A061GR29	Pentatricopeptide repeat superfamily protein isoform 1
PI467895	G90327.7	Up	NA	Chr3g0140441	HB-other	HB-other	TF	NA	NA	NA	A0A0B0MF94	Uncharacterized protein
PI467895	G35386.10	Up	NA	Chr3g0100831	NA	NA	NA	NA	NA	NA	A0A371GUE3	Disease resistance protein RPM1 (Fragment)
PI467895	G73420.2	Up	NA	Chr8g0352401	NA	NA	NA	NA	NA	NA	A0A445JSZ3	Jasmonic acid-amido synthetase JAR1 isoform A (Jasmonic acid-amido synthetase JAR1 isoform B) (Jasmonic acid-amido synthetase JAR1 isoform C) (Jasmonic acid-amido synthetase JAR1 isoform D)
PI467895	G87470.21	Up	NA	Chr1g0159501	NA	NA	NA	NA	NA	NA	A0A5D2I633	J domain-containing protein
PI467895	G84367.30	Up	NA	Chr3g0077931	NA	RB	TR	NA	NA	NA	V7ALH0	Uncharacterized protein
PI467895	G10878.38	Up	NA	Chr1g0190441	NA	NA	NA	NA	NA	NA	UPI001247AAF0	bifunctional TH2 protein mitochondrial-like isoform X2
PI467895	G86253.5	Up	NA	Chr3g0116171	NA	NA	NA	NA	NA	NA	A0A0B2QP92	Ribonuclease J
PI467895	G36599.4	Up	NA	Chr5g0436781	NA	NA	NA	NA	NA	NA	I1M7W2	Uncharacterized protein
PI467895	G54934.17	Up	NA	Chr5g0407021	NA	NA	NA	NA	NA	NA	V4SN51	AAA domain-containing protein
PI467895	G87106.9	Up	NA	Chr8g0363801	NA	NA	NA	NA	NA	NA	G7LGM4	F-box/RNI/FBD-like domain protein
PI467895	G30859.9	Up	NA	Chr5g0447111	NA	TRAF	TR	NA	NA	NA	A0A072V0U7	BTB/POZ domain plant protein
PI467895	G43865.25	Up	NA	Chr4g0029941	NA	NA	NA	NA	NA	NA	A0A0A9CPD9	RNL
PI467895	G25047.97	Up	NA	Chr7g0233411	NA	NA	NA	NA	NA	NA	UPI000DECAEDA	uncharacterized protein LOC112764188 isoform X2
PI467895	G64806.8	Up	NA	Chr7g0223051	NA	NA	NA	NA	NA	NA	A0A199UA29	Uncharacterized protein
PI467895	G80098.12	Up	NA	Chr2g0316821	NA	NA	NA	NA	NA	NA	A0A0S3RFA6	Glyceraldehyde-3-phosphate dehydrogenase (EC 1.2.1.-)
PI467895	G31508.7	Up	NA	Chr3g0136011	NA	NA	NA	NA	NA	NA	A0A151R4V5	Putative methyltransferase NSUN5
PI467895	G55822.3	Up	NA	Chr5g0425291	NA	NA	NA	NA	NA	NA	A0A4U5QYN9	Mannan endo-1 4-beta-mannosidase (EC 3.2.1.78)
PI467895	G21419.6	Up	NA	Chr2g0316851	NA	NA	NA	NA	NA	NA	A0A151R837	TAT-binding isogeny 7
PI467895	G29311.6	Up	NA	Chr3g0084721	NA	NA	NA	NA	NA	NA	UPI000A2C0E55	TMV resistance protein N-like isoform X2
PI467895	G59961.9	Up	NA	Chr8g0346011	NA	NA	NA	NA	NA	NA	A0A444WT97	Uncharacterized protein
PI467895	G41342.4	Up	NA	Chr1g0209771	NA	NA	NA	NA	NA	NA	A0A4P1QU35	Uncharacterized protein
PI467895	G2190.37	Up	NA	Chr7g0227361	NA	NA	NA	NA	NA	NA	UPI0012493A41	RNA-directed DNA methylation 4 isoform X5
PI467895	G70331.11	Up	NA	Chr5g0426671	NA	NA	NA	NA	NA	NA	UPI0002C31003	probable mitochondrial saccharopine dehydrogenase-like oxidoreductase At5g39410

PI467895	G40431.6	Up	NA	Chr3g0114941	NA	NA	NA	NA	NA	NA	A0A445DZC2	RNase_PH domain-containing protein
PI467895	G9141.1	Up	NA	Chr1g0153721	NA	NA	NA	NA	NA	NA	UPI000B7B1731	Uncharacterized protein;succinate dehydrogenase subunit 5 mitochondrial;(apricot) hypothetical protein
PI467895	G80468.6	Up	NA	Chr1g0163291	NA	NA	NA	NA	NA	NA	UPI0010A474BE	uncharacterized protein LOC114722208
PI467895	G47648.13	Up	NA	Chr1g0190271	NA	NA	NA	NA	NA	NA	I1L5P6	Uncharacterized protein
PI467895	G36928.13	Up	NA	Chr7g0253781	NA	NA	NA	NA	NA	NA	A0A2N9I411	5-methyltetrahydropteroyltriglutamate--homocysteine S-methyltransferase (EC 2.1.1.14)
PI467895	G73336.15	Up	NA	Chr5g0394341	NA	NA	NA	NA	NA	NA	A0A2K3NH38	Inositol transporter 1-like protein
PI467895	G11520.1	Up	NA	Chr1g0147151	NA	NA	NA	NA	NA	NA	I3SVX8	Uncharacterized protein
PI467895	G68499.14	Up	NA	Chr5g0440371	NA	NA	NA	NA	NA	NA	I1LID3	3-hydroxy-3-methylglutaryl coenzyme A reductase (HMG-CoA reductase) (EC 1.1.1.34)
PI467895	G10228.27	Up	NA	Chr7g0267701	NA	NA	NA	NA	NA	NA	UPI00092ED387	uncharacterized protein LOC109334869 isoform X2
PI467895	G37500.5	Up	NA	Chr8g0391291	NA	NA	NA	NA	NA	NA	A0A4S4DYM7	Pectinesterase (EC 3.1.1.11)
Wilson	G24054.29	NA	Down	Chr3g0123781	NA	NA	NA	NA	NA	NA	UPI000B78E3CB	Uncharacterized protein;lanC-like protein GCR2 isoform X1;lanC-like protein GCR2 isoform X2;hypothetical protein
Wilson	G81845.8	NA	Down	Chr3g0113431	NA	NA	NA	NA	NA	NA	UPI000C03B385	protein ELF4-LIKE 4-like isoform X2
Wilson	G52869.1	NA	Down	Chr4g0054421	HD-ZIP	HB-HD-ZIP	TF	NA	NA	NA	I3T115	Uncharacterized protein
Wilson	G18231.2	NA	Down	Chr7g0221311	NA	NA	NA	NA	NA	NA	UPI000B7B2330	protein FLX-like 3
Wilson	G36068.1	NA	Down	Chr7g0223381	NA	NA	NA	NA	NA	NA	A0A445JN11	Transcription initiation factor TFIID subunit 6 isoform B
Wilson	G22500.4	NA	Down	Chr1g0175081	G2-like	NA	NA	NA	NA	NA	A0A4P1QPZ1	HTH myb-type domain-containing protein
Wilson	G66825.21	NA	Down	Chr7g0253401	NA	NA	NA	NA	NA	NA	UPI000F7C592B	5'-adenylylsulfate reductase-like 5 isoform X2
Wilson	G41468.2	NA	Down	Chr4g0001871	NA	NA	NA	NA	NA	NA	A0A072UGJ1	Transmembrane protein putative
Wilson	G39991.3	NA	Down	Chr4g0037621	NA	NA	NA	NA	NA	NA	A0A2N9FKC9	Uncharacterized protein
Wilson	G7201.3	NA	Down	Chr1g0181551	NA	NA	NA	NA	NA	NA	R0HCW6	acidPPc domain-containing protein
Wilson	G61267.9	NA	Down	Chr5g0435541	NA	NA	NA	NA	NA	NA	UPI0007889062	aspartic proteinase PCS1
Wilson	G49800.4	NA	Down	Chr4g0044271	NA	NA	NA	NA	NA	NA	UPI00046E2039	leucine-rich repeat extensin-like protein 1
Wilson	G17773.7	NA	Down	Chr2g0282561	NA	NA	NA	NA	NA	NA	A0A445BBJ6	Uncharacterized protein
Wilson	G87432.8	NA	Down	Chr8g0360231	NA	NA	NA	NA	NA	NA	A0A540NGU4	Exonuclease domain-containing protein
Wilson	G80228.6	NA	Down	Chr6g0484011	NA	NA	NA	NA	NA	NA	A0A1S3VM71	bax inhibitor 1
Wilson	G49478.3	NA	Down	Chr1g0188071	NA	NA	NA	NA	NA	NA	A0A2P5EVS0	Phosphate-induced protein
Wilson	G48262.4	NA	Down	Chr4g0011831	NA	NA	NA	NA	NA	NA	A0A444XBT8	Golgi SNAP receptor complex member 1
Wilson	G46169.5	NA	Down	Chr4g0050461	NA	NA	NA	NA	NA	NA	A0A2P5AA34	Heavy metal-associated domain containing protein
Wilson	G45348.28	NA	Down	NA	NA	NA	NA	NA	NA	NA	NA	NA
Wilson	G60132.2	NA	Down	Chr5g0401911	NA	NA	NA	NA	NA	NA	A0A2K3L9D8	Filament-plant-like protein
Wilson	G81637.7	NA	Down	Chr7g0225661	bZIP	bZIP	TF	NA	NA	NA	A0A0S3S4W0	BZIP domain-containing protein
Wilson	G21227.8	NA	Down	Chr7g0265761	C2H2	C2H2	TF	NA	NA	NA	A0A392P026	Uncharacterized protein (Fragment)
Wilson	G83111.2	NA	Down	Chr2g0325921	NA	NA	NA	NA	NA	NA	A0A1J7H0S3	RING-type domain-containing protein
Wilson	G86699.6	NA	Down	Chr3g0103431	NA	NA	NA	NA	NA	NA	UPI00057A0E0E	heterogeneous nuclear ribonucleoprotein H2 isoform X1
Wilson	G24640.8	NA	Down	Chr4g0075181	NA	NA	NA	NA	NA	NA	A0A151SSN8	Aspartic proteinase (EC 3.4.23.40)
Wilson	G29098.3	NA	Down	Chr3g0080991	NA	NA	NA	NA	NA	NA	A0A2K3MJ21	Cold shock protein 1 (Fragment)
Wilson	G48106.11	NA	Down	Chr7g0244031	NA	NA	NA	NA	NA	NA	A0A1S3VD86	Protein SCAR (Protein WAVE)
Wilson	G20726.2	NA	Down	Chr2g0333131	NA	NA	NA	NA	NA	NA	UPI00098D92FA	histidine-containing phosphotransfer protein 4
Wilson	G29511.11	NA	Down	Chr3g0093251	NA	NA	NA	NA	NA	NA	A0A1S3TRZ8	5-formyltetrahydrofolate cyclo-ligase (EC 6.3.3.2)
Wilson	G65972.20	NA	Down	Chr8g0352841	NA	NA	NA	NA	NA	NA	A0A2N9GL55	CitMHS domain-containing protein
Wilson	G79315.10	NA	Down	Chr7g0270221	NA	NA	NA	NA	NA	NA	UPI000CE187CB	4-coumarate--coa ligase 1;4-coumarate--CoA ligase 1-like;4-coumarate--CoA ligase 1
Wilson	G38242.14	NA	Down	Chr3g0145951	NA	NA	NA	NA	NA	NA	UPI0011D29EA3	coiled-coil domain-containing protein 124
Wilson	G25466.2	NA	Down	Chr2g0283431	NA	NA	NA	NA	NA	NA	A0A2N9IXP1	Uncharacterized protein
Wilson	G21716.5	NA	Down	Chr8g0380421	NA	NA	NA	NA	NA	NA	UPI000809D4B5	uncharacterized protein LOC108334170 isoform X3
Wilson	G73091.10	NA	Down	Chr3g0119651	NA	NA	NA	NA	NA	NA	A0A5J5AHD7	Transcription initiation factor TFIID subunit 8
Wilson	G41911.3	NA	Down	Chr8g0387091	NA	NA	NA	NA	NA	NA	UPI000F7C868A	puromycin-sensitive aminopeptidase isoform X3
Wilson	G25979.18	NA	Down	Chr6g0457871	NA	NA	NA	NA	NA	NA	A0A0L9V8F0	Uncharacterized protein
Wilson	G31789.52	NA	Down	Chr3g0099611	NA	NA	NA	NA	NA	NA	A0A4P1QYI5	1-4-alpha-glucan branching enzyme (EC 2.4.1.18)
Wilson	G21814.10	NA	Down	Chr8g0351531	NA	NA	NA	NA	NA	NA	A0A1S3UQM9	uncharacterized protein LOC106767929
Wilson	G3086.55	NA	Down	Chr5g0402151	NA	NA	NA	NA	NA	NA	A0A371FM37	Amino acid permease 6
Wilson	G10985.2	NA	Down	Chr1g0192261	NA	NA	NA	NA	lncRNA	NA	UPI0010A496C1	uncharacterized protein LOC114737178
Wilson	G50958.4	NA	Down	Chr6g0486531	NA	NA	NA	NA	NA	NA	A0A392M9S0	Elongation factor Ts mitochondrial (EF-Ts) (EF-TsMt)
Wilson	G3448.12	NA	Down	Chr2g0332141	NA	NA	NA	NA	NA	NA	I1NIA4	Auxin-induced protein
Wilson	G48816.14	NA	Down	Chr3g0095791	NA	NA	NA	NA	NA	NA	A0A4P1R3T4	Cellulose synthase (EC 2.4.1.12)
Wilson	G62849.21	NA	Down	Chr4g0039911	NA	NA	NA	NA	NA	NA	UPI000CED42D3	K(+) efflux antiporter 5 isoform X2
Wilson	G23596.4	NA	Down	Chr4g0022901	MYB	MYB-related	TF	NA	NA	NA	A0A438FAG8	Transcription factor MYB59
Wilson	G91138.13	NA	Down	Chr3g0093281	NA	NA	NA	NA	NA	NA	A0A1S3CBW8	vacuolar protein sorting-associated protein 26A-like
Wilson	G58263.13	NA	Down	Chr4g0020191	NA	NA	NA	NA	NA	NA	A0A1S3VF81	calcium-binding protein 39
Wilson	G85468.1	NA	Down	Chr2g0284491	NA	NA	NA	NA	NA	NA	B7FME0	Transmembrane protein 230
Wilson	G20833.2	NA	Down	Chr2g0309091	NA	NA	NA	NA	NA	NA	UPI0003DEA9AF	uncharacterized protein LOC100775984 isoform X2;uncharacterized protein LOC114377336 isoform X2
Wilson	G82949.8	NA	Down	Chr4g0062701	NA	NA	NA	NA	NA	NA	A0A1F4AQL6	Uncharacterized protein
Wilson	G3625.3	NA	Down	Chr1g0170821	NA	NA	NA	NA	NA	NA	A0A4D6LWN1	Uncharacterized protein
Wilson	G30379.1	NA	Down	Chr2g0285851	NA	NA	NA	NA	NA	NA	A0A1S3UYM4	F-box/kelch-repeat protein At1g22040
Wilson	G81783.6	NA	Down	Chr5g0396911	ERF	AP2/ERF-ERF	TF	NA	NA	NA	A0A371EKP6	Ethylene-responsive transcription factor 1
Wilson	G72370.1	NA	Down	NA	NA	NA	NA	NA	NA	NA	NA	NA
Wilson	G65849.5	NA	Down	Chr6g0484961	NA	NA	NA	NA	NA	NA	A0A396HPQ3	Uncharacterized protein
Wilson	G52256.126	NA	Down	Chr5g0403961	NA	NA	NA	NA	lncRNA	NA	A0A396HS08	Uncharacterized protein
Wilson	G53946.6	NA	Down	Chr5g0442811	NA	NA	NA	NA	NA	NA	UPI0010A2E020	switch-associated protein 70 isoform X2
Wilson	G11575.13	NA	Down	Chr4g0067281	NA	NA	NA	NA	NA	NA	UPI0011E06B86	xyloglucan galactosyltransferase XLT2-like
Wilson	G19449.59	NA	Down	Chr7g0262411	NA	NA	NA	NA	NA	NA	A0A445GV65	DENN domain-containing protein 5B isoform D
Wilson	G74534.3	NA	Down	Chr6g0461011	NA	NA	NA	NA	NA	NA	A0A1J7GRX5	Uncharacterized protein
Wilson	G826.6	NA	Down	Chr8g0345331	NA	NA	NA	NA	NA	NA	C6T9C6	UBC core domain-containing protein
Wilson	G63788.3	NA	Up	Chr7g0257481	NA	NA	NA	NA	NA	NA	UPI0010169ADB	LOW QUALITY PROTEIN: eukaryotic translation initiation factor 3 subunit M-like
Wilson	G29511.12	NA	Up	Chr3g0093251	NA	NA	NA	NA	NA	NA	A0A1S3TRZ8	5-formyltetrahydrofolate cyclo-ligase (EC 6.3.3.2)
Wilson	G17184.9	NA	Up	Chr1g0174791	NA	NA	NA	NA	NA	NA	UPI00046E0D23	Importin subunit alpha;importin subunit alpha-2;(apricot) hypothetical protein
Wilson	G76866.1	NA	Up	Chr3g0118431	NA	NA	NA	NA	NA	NA	M5VJ61	S-(hydroxymethyl)glutathione dehydrogenase (EC 1.1.1.284)
Wilson	G60526.19	NA	Up	Chr1g0191711	NA	NA	NA	NA	NA	NA	A0A5N6R234	NPH3 domain-containing protein
Wilson	G36640.1	NA	Up	Chr3g0114041	NA	NA	NA	NA	NA	NA	A0A445FN45	Multiple inositol polyphosphate phosphatase 1 isoform D
Wilson	G23548.2	NA	Up	Chr1g0185161	NA	NA	NA	NA	NA	NA	UPI000C9D8159	uncharacterized protein LOC111794502
Wilson	G76113.24	NA	Up	Chr3g0135621	NA	NA	NA	NA	NA	NA	A0A445J585	KH domain-containing protein isoform A (KH domain-containing protein isoform B) (KH domain-containing protein isoform C) (KH domain-containing protein isoform D) (KH domain-containing protein isoform E) (KH domain-containing protein isoform F) (KH domain-containing protein isoform G)
Wilson	G22192.9	NA	Up	Chr3g0102421	NA	NA	NA	NA	NA	NA	A0A445DIK4	Uncharacterized protein
Wilson	G38269.16	NA	Up	Chr1g0147621	NA	NA	NA	NA	NA	NA	A0A392RT05	Translocon at the outer membrane of chloroplasts 64-like (Fragment)
Wilson	G50111.15	NA	Up	Chr4g0068581	NA	NA	NA	NA	NA	NA	A0A445H9C6	Methionyl-tRNA synthetase (EC 6.1.1.10)
Wilson	G73604.1	NA	Up	Chr4g0011751	NA	NA	NA	NA	NA	NA	UPI001247F0FE	Uncharacterized protein;putative pectinesterase/pectinesterase inhibitor 22
Wilson	G19995.12	NA	Up	Chr8g0376331	NA	NA	NA	NA	NA	NA	UPI00092F6B5D	starch synthase 3 chloroplastic/amyloplastic-like isoform X4
Wilson	G28918.19	NA	Up	Chr1g0211071	NA	NA	NA	NA	NA	NA	V7ARR0	Ribosomal_L18_c domain-containing protein
Wilson	G31754.2	NA	Up	Chr1g0157071	NA	NA	NA	NA	NA	NA	I3T3B2	Uncharacterized protein
Wilson	G8354.10	NA	Up	Chr3g0119231	NA	NA	NA	NA	NA	NA	A0A2P5CV38	GPN-loop GTPase
Wilson	G64834.42	NA	Up	Chr6g0483061	MYB	MYB	TF	NA	NA	NA	A0A0S3TC31	Uncharacterized protein
Wilson	G85808.1	NA	Up	Chr5g0396831	NA	NA	NA	NA	NA	NA	A0A2N9IUI6	Uncharacterized protein
Wilson	G31871.13	NA	Up	Chr5g0405011	NA	NA	NA	NA	NA	NA	A0A5B6U8E4	Protein FMP32 mitochondrial-like
Wilson	G55087.1	NA	Up	Chr7g0222991	NA	NA	NA	NA	NA	NA	A0A453MZP2	Histone domain-containing protein
Wilson	G44040.14	NA	Up	Chr4g0026241	NA	NA	NA	NA	lncRNA	NA	UPI0010A56E99	uncharacterized protein LOC114716085;uncharacterized protein LOC114744612
Wilson	G20797.3	NA	Up	Chr3g0092521	NA	NA	NA	NA	NA	NA	A0A5J5BAT9	Uncharacterized protein
Wilson	G33475.6	NA	Up	Chr5g0426311	NA	NA	NA	NA	NA	NA	A0A0R0JQU1	Uncharacterized protein
Wilson	G51495.10	NA	Up	Chr7g0236461	NA	NA	NA	NA	NA	NA	A0A5N6RHZ4	Uncharacterized protein
Wilson	G2769.4	NA	Up	Chr6g0463761	NA	NA	NA	NA	NA	NA	A0A5N6R9C8	Squalene monooxygenase (EC 1.14.14.17)
Wilson	G41976.6	NA	Up	Chr3g0141721	NA	NA	NA	NA	NA	NA	UPI00051129CE	gamma carbonic anhydrase-like 2 mitochondrial isoform X2;gamma carbonic anhydrase-like 2 mitochondrial isoform X1
Wilson	G11438.3	NA	Up	Chr4g0051121	NA	NA	NA	NA	NA	NA	C6THR0	Uncharacterized protein
Wilson	G65077.2	NA	Up	Chr6g0488811	NA	NA	NA	NA	NA	NA	UPI001015EAE7	protein APEM9-like
Wilson	G38160.30	NA	Up	Chr3g0144231	NA	NA	NA	NA	NA	NA	A0A061FDE1	Chloroplast channel forming outer membrane protein isoform 1

Wilson	G2903.7	NA	Up	Chr4g0055821	NA	NA	NA	NA	NA	NA	A0A4D6NTN2	PPPDE domain-containing protein
Wilson	G13118.1	NA	Up	Chr1g0181761	NA	NA	NA	NA	NA	NA	A0A075BIU4	60S ribosomal protein L4/L1
Wilson	G28426.58	NA	Up	Chr3g0136761	NA	NA	NA	NA	NA	NA	A0A2P5DIP9	Calcineurin-like phosphoesterase
Wilson	G15110.1	NA	Up	Chr3g0108661	NA	NA	NA	NA	NA	NA	UPI0005811CD4	Reticulon-like protein;reticulon-like protein B9
Wilson	G86699.5	NA	Up	Chr3g0103431	NA	NA	NA	NA	NA	NA	UPI00057A0E0E	heterogeneous nuclear ribonucleoprotein H2 isoform X1
Wilson	G3084.5	NA	Up	Chr1g0146551	NA	NA	NA	NA	NA	NA	A0A2I4EQN6	uncharacterized protein LOC108991772 isoform X1
Wilson	G12643.3	NA	Up	Chr3g0139391	NA	NA	NA	NA	NA	NA	A0A0B0NXH2	Uncharacterized protein
Wilson	G40474.20	NA	Up	Chr4g0026581	NA	NA	NA	NA	NA	NA	A0A251SR24	Putative bifunctional polymyxin resistance protein ArnA
Wilson	G38135.13	NA	Up	Chr7g0225431	NA	NA	NA	NA	NA	NA	A0A2I4FXL1	pre-rRNA-processing protein TSR1 homolog
Wilson	G43991.4	NA	Up	Chr2g0320901	NA	NA	NA	NA	lncRNA	NA	C6TJ33	C2H2-type domain-containing protein
Wilson	G15473.12	NA	Up	Chr4g0003811	NA	NA	NA	NA	NA	NA	V7APY7	Uncharacterized protein
Wilson	G52796.35	NA	Up	Chr5g0414351	NA	NA	NA	NA	NA	NA	A0A5N5H9S7	Protein NLRC3
Wilson	G67695.2	NA	Up	Chr7g0276081	NA	NA	NA	NA	NA	NA	A0A067FGQ3	Cytochrome b5 heme-binding domain-containing protein
Wilson	G34506.35	NA	Up	Chr3g0125071	NA	NA	NA	NA	NA	NA	A0A5N6RQH6	Peptidase_S26 domain-containing protein
Wilson	G80214.11	NA	Up	Chr1g0162951	NA	NA	NA	NA	NA	NA	A0A2N9H3T8	BED-type domain-containing protein
Wilson	G29604.1	NA	Up	Chr2g0286131	NA	NA	NA	NA	NA	NA	A0A4D6MT13	Large subunit ribosomal protein L18Ae
Wilson	G12363.2	NA	Up	Chr1g0152091	NA	NA	NA	NA	NA	NA	A0A5B7BBC4	Uncharacterized protein (Fragment)
Wilson	G89920.6	NA	Up	Chr5g0429811	NA	NA	NA	NA	NA	NA	A0A4D6KX77	Ditrans
Wilson	G89612.12	NA	Up	Chr4g0055491	NA	NA	NA	NA	NA	NA	A0A5J5TIY7	Cupin_2 domain-containing protein
Wilson	G53879.6	NA	Up	Chr4g0035591	SRS	SRS	TF	NA	NA	NA	A0A3Q7Y8K3	protein LATERAL ROOT PRIMORDIUM 1 isoform X2
Wilson	G76538.10	NA	Up	Chr7g0270481	NA	NA	NA	NA	NA	NA	A0A438IXJ7	RING-type E3 ubiquitin transferase (EC 2.3.2.27)
Wilson	G76862.1	NA	Up	Chr5g0408081	NA	NA	NA	NA	NA	NA	A0A0R0F461	Dimer_Tnp_hAT domain-containing protein
Wilson	G25108.13	NA	Up	Chr2g0311151	NA	NA	NA	NA	NA	NA	A0A4D6NAX7	Elongation factor G mitochondrial (EF-Gmt) (Elongation factor G 1 mitochondrial) (mEF-G 1) (Elongation factor G1)
Wilson	G24288.3	NA	Up	Chr2g0312311	NA	NA	NA	NA	NA	NA	A0A445C342	Uncharacterized protein
Wilson	G28523.2	NA	Up	Chr4g0063201	NA	NA	NA	NA	NA	NA	A0A371G8P5	GTP-binding nuclear protein (Fragment)
Wilson	G80719.49	NA	Up	Chr8g0368401	NA	NA	NA	NA	NA	NA	A0A1J7GW79	PPDK_N domain-containing protein
Wilson	G77658.3	NA	Up	Chr5g0397071	NA	NA	NA	NA	NA	NA	A0A2P5ARY8	GDT1 family protein
Wilson	G63570.7	NA	Up	Chr7g0274711	NA	NA	NA	NA	NA	NA	UPI000CE228FC	RING-H2 finger protein ATL46
Wilson	G75828.3	NA	Up	Chr1g0212551	NA	NA	NA	NA	NA	NA	V7BAX2	Transket_pyr domain-containing protein
Wilson	G20033.4	NA	Up	Chr2g0287881	NA	NA	NA	NA	NA	NA	A0A0L9TT88	ATP-dependent (S)-NAD(P)H-hydrate dehydratase (EC 4.2.1.93) (ATP-dependent NAD(P)HX dehydratase)
Wilson	G73619.9	NA	Up	Chr1g0174801	NA	NA	NA	NA	NA	NA	G7I9F7	Importin subunit alpha
Wilson	G7985.1	NA	Up	Chr5g0426461	NA	NA	NA	NA	NA	NA	UPI0010FC5FCB	rho GTPase-activating protein 2-like
Wilson	G87689.4	NA	Up	Chr5g0406711	NA	NA	NA	NA	NA	NA	M5VWV5	Serine O-acetyltransferase (EC 2.3.1.30)
Wilson	G91276.1	NA	Up	Chr8g0336121	NA	NA	NA	NA	NA	NA	UPI000904BDF5	uncharacterized protein LOC109219494
Wilson	G21218.8	NA	Up	Chr3g0122871	NA	NA	NA	NA	NA	NA	A0A660KNR9	Peptidylprolyl isomerase (EC 5.2.1.8)
Wilson	G4406.6	NA	Up	Chr1g0189421	NA	NA	NA	NA	NA	NA	UPI0011D18574	ubiquitin carboxyl-terminal hydrolase 2
Wilson	G20087.14	NA	Up	NA	NA	NA	NA	NA	lncRNA	NA	NA	NA
Wilson	G3703.4	NA	Up	Chr8g0356201	NA	NA	NA	NA	NA	NA	A0A1J7HJ11	GlcNAc-1-P transferase (EC 2.7.8.15) (N-acetylglucosamine-1-phosphate transferase) (UDP-N-acetylglucosamine--dolichyl-phosphate N-acetylglucosaminephosphotransferase)
Wilson	G7892.5	NA	Up	Chr4g0068171	NA	NA	NA	NA	NA	NA	A0A438IMM9	Uncharacterized protein
Wilson	G43646.3	NA	Up	Chr4g0034131	NA	NA	NA	NA	NA	NA	UPI0007887D19	Proteasome assembly chaperone 2;proteasome assembly chaperone 2 isoform X2
Wilson	G68915.9	NA	Up	Chr6g0481321	NA	NA	NA	NA	NA	NA	UPI0011E0080E	Uncharacterized protein;eukaryotic peptide chain release factor subunit 1-3
Wilson	G5398.7	NA	Up	Chr0c31g049416	NA	NA	NA	NA	NA	NA	A0A660KTN6	RRM domain-containing protein
Wilson	G79892.6	NA	Up	Chr1g0197401	NA	NA	NA	NA	NA	NA	UPI000809C40F	tip elongation aberrant protein 1-like isoform X4
Wilson	G41566.1	NA	Up	Chr1g0153871	NA	NA	NA	NA	NA	NA	UPI0010A3C524	eukaryotic translation initiation factor 3 subunit G-like
Saranac	G12460.3	Down	Down	Chr5g0427431	NA	NA	NA	NA	NA	NA	A0A067G2A8	RING-type domain-containing protein
Saranac	G12770.2	Down	Down	Chr3g0112681	NA	NA	NA	NA	NA	NA	A0A061FPS0	Ras-related protein Rab-18
Saranac	G14358.12	Down	Down	Chr1g0188941	WRKY	NA	NA	NA	NA	NA	A0A444XHZ1	WRKY transcription factor
Saranac	G17969.4	Down	Down	Chr1g0198231	NA	NA	NA	NA	NA	NA	A0A2P5FUS1	Coatomer beta subunit
Saranac	G28227.10	Down	Down	Chr3g0130841	NA	NA	NA	NA	NA	NA	UPI0010A41444	CBS domain-containing protein CBSCBSPB5-like isoform X2
Saranac	G32906.5	Down	Down	Chr2g0294681	NA	NA	NA	NA	NA	NA	A0A371I8H2	Phosducin-like protein 3
Saranac	G34047.3	Down	Down	Chr3g0126711	NA	NA	NA	NA	NA	NA	A0A396IY45	Uncharacterized protein
Saranac	G34114.9	Down	Down	Chr2g0312971	NA	NA	NA	NA	NA	NA	A0A445L4E6	Polyadenylate-binding protein-interacting protein 11 isoform A (Polyadenylate-binding protein-interacting protein 11 isoform B) (Polyadenylate-binding protein-interacting protein 11 isoform C) (Polyadenylate-binding protein-interacting protein 11 isoform D)
Saranac	G37119.2	Down	Down	Chr3g0122471	NA	NA	NA	NA	NA	NA	UPI000CE23635	Alpha-ketoglutarate-dependent dioxygenase alkb;alpha-ketoglutarate-dependent dioxygenase alkB;alpha-ketoglutarate-dependent dioxygenase alkb
Saranac	G37401.2	Down	Down	Chr1g0158601	NA	NA	NA	NA	NA	NA	UPI000C041EE4	IAA-amino acid hydrolase ILR1-like 3 isoform X2
Saranac	G49154.2	Down	Down	Chr7g0219391	NA	NA	NA	NA	NA	NA	A0A445AB21	AAI domain-containing protein
Saranac	G79521.4	Down	Down	Chr4g0068661	NA	NA	NA	NA	NA	NA	K7MJS5	Uncharacterized protein
Saranac	G82659.32	Down	Down	Chr5g0430701	NA	NA	NA	NA	NA	NA	UPI000786BB57	protein CHUP1 chloroplastic isoform X3
Saranac	G10323.17	Up	Down	Chr7g0265781	NA	NA	NA	NA	NA	NA	A0A4P1R8Q0	CID domain-containing protein
Saranac	G13471.1	Up	Down	Chr3g0125641	NA	NA	NA	NA	NA	NA	C6T8G9	Uncharacterized protein
Saranac	G15692.8	Up	Down	Chr7g0264951	NA	NA	NA	NA	NA	NA	A0A444Y8D3	Uncharacterized protein
Saranac	G16025.3	Up	Down	Chr1g0172661	NA	NA	NA	NA	NA	NA	A0A067EHS2	Uncharacterized protein
Saranac	G19037.5	Up	Down	Chr3g0116821	NA	NA	NA	NA	NA	NA	A0A445D960	Protein-synthesizing GTPase (EC 3.6.5.3)
Saranac	G26871.2	Up	Down	Chr5g0436951	NA	NA	NA	NA	NA	NA	A0A2P5XSV3	PH domain-containing protein
Saranac	G28697.13	Up	Down	Chr6g0486611	NA	NA	NA	CK1_CK1-Pl	NA	NA	A0A059AJ20	Protein kinase domain-containing protein
Saranac	G31951.5	Up	Down	Chr3g0077641	NA	NA	NA	NA	NA	NA	UPI000C20463B	LOW QUALITY PROTEIN: putative methyltransferase At1g22800 mitochondrial
Saranac	G41399.10	Up	Down	Chr3g0127811	NA	NA	NA	NA	NA	NA	A0A5N6QDG3	O-fucosyltransferase family protein
Saranac	G43439.9	Up	Down	Chr2g0312101	NA	NA	NA	NA	NA	NA	A0A151TMZ2	U-box domain-containing protein 62
Saranac	G56642.1	Up	Down	Chr8g0381831	NA	NA	NA	NA	NA	circRNA	UPI00077E3E2E	uncharacterized protein LOC107416119 isoform X2
Saranac	G59055.3	Up	Down	Chr5g0440361	NA	NA	NA	NA	NA	NA	A0A1U7ZFS6	KRR-R motif-containing protein 1
Saranac	G60077.2	Up	Down	Chr4g0035121	NA	NA	NA	NA	NA	NA	UPI0010167213	isoflavone reductase-like protein
Saranac	G60243.14	Up	Down	Chr8g0369931	NA	NA	NA	NA	NA	NA	A0A2K3LT54	Diacylglycerol o-acyltransferase 2-like protein
Saranac	G60858.4	Up	Down	Chr4g0054261	NA	NA	NA	NA	NA	NA	A0A1S3UHE3	ACT domain-containing protein ACR4 isoform X1
Saranac	G61869.5	Up	Down	Chr5g0443821	NA	NA	NA	NA	NA	NA	V7B8X3	tRNA-uridine aminocarboxypropyltransferase (EC 2.5.1.25)
Saranac	G61882.25	Up	Down	Chr8g0372071	NA	NA	NA	NA	NA	NA	UPI000F7C5E4A	peptide methionine sulfoxide reductase A1-like isoform X1
Saranac	G68386.38	Up	Down	Chr7g0265961	NA	NA	NA	NA	NA	NA	A0A4D6MAL4	ATP-binding cassette
Saranac	G71534.6	Up	Down	Chr4g0009901	G2-like	GARP-G2-like	TF	NA	NA	NA	A0A5N6RUZ8	HTH myb-type domain-containing protein
Saranac	G7185.1	Up	Down	Chr1g0198651	C2H2	NA	NA	NA	NA	NA	K7MZ06	C2H2-type domain-containing protein
Saranac	G74698.38	Up	Down	Chr4g0066581	NA	NA	NA	NA	NA	NA	A0A392V9Z1	Plastidic glucose transporter 4-like (Fragment)
Saranac	G75025.1	Up	Down	Chr4g0068381	NA	NA	NA	NA	NA	NA	A0A1S3TFC7	Dirigent protein
Saranac	G76360.4	Up	Down	Chr2g0314601	NA	NA	NA	NA	NA	NA	A0A314YLY3	GDP-mannose 3 5-epimerase 2
Saranac	G80285.9	Up	Down	Chr4g0054841	NA	NA	NA	NA	NA	NA	G7JHL6	Transmembrane protein putative
Saranac	G84737.1	Up	Down	Chr8g0347771	NA	NA	NA	NA	NA	NA	UPI0011E067BC	uncharacterized protein LOC115719695
Saranac	G86283.6	Up	Down	Chr5g0412271	MIKC_MA	MADS-MIKC	TF	NA	NA	NA	UPI000B76CD23	agamous-like MADS-box protein AGL19
Saranac	G88353.2	Up	Down	Chr3g0119291	HD-ZIP	NA	NA	NA	NA	NA	A0A660KX83	Uncharacterized protein
Saranac	G88413.17	Up	Down	Chr8g0354951	NA	NA	NA	NA	NA	NA	UPI00071932D6	snRNA-activating protein complex subunit isoform X4
Saranac	G11338.31	Down	Up	Chr5g0446911	NA	NA	NA	NA	NA	NA	UPI0010A33C4C	ABC transporter G family member 21-like isoform X2
Saranac	G14802.15	Down	Up	Chr1g0203311	NA	NA	NA	NA	NA	NA	UPI00092E2202	myosin-11-like
Saranac	G15621.17	Down	Up	Chr2g0329541	NA	NA	NA	NA	NA	NA	UPI0010A33850	LOW QUALITY PROTEIN: electron transfer flavoprotein-ubiquinone oxidoreductase mitochondrial-like
Saranac	G16361.16	Down	Up	Chr3g0115681	NA	NA	NA	NA	NA	NA	A0A061F8N8	Tetratricopetide-repeat thioredoxin-like 1 isoform 1
Saranac	G17129.17	Down	Up	Chr6g0484501	NA	NA	NA	NA	NA	NA	I1L0Z5	Kinesin-like protein
Saranac	G18198.2	Down	Up	Chr2g0333131	NA	NA	NA	NA	NA	NA	UPI00098D92FA	histidine-containing phosphotransfer protein 4
Saranac	G21122.1	Down	Up	Chr2g0324121	NA	NA	NA	NA	NA	NA	UPI000786D54F	uncharacterized protein LOC107462177 isoform X2
Saranac	G2291.8	Down	Up	Chr6g0467841	NA	NA	NA	NA	NA	NA	A0A2K3KZX2	Uncharacterized protein
Saranac	G24183.12	Down	Up	Chr2g0314981	NA	NA	NA	NA	NA	NA	G7IJE2	Non-specific serine/threonine protein kinase (EC 2.7.11.1)
Saranac	G2439.3	Down	Up	Chr3g0140391	NA	NA	NA	NA	NA	NA	K7KGB9	Hydrolase_4 domain-containing protein
Saranac	G24779.92	Down	Up	Chr8g0372581	NA	NA	NA	NA	NA	NA	A0A445A266	Beta-galactosidase (EC 3.2.1.23)
Saranac	G26264.36	Down	Up	Chr3g0087171	NA	NA	NA	NA	NA	NA	UPI00092F6AD5	AP2-associated protein kinase 1-like
Saranac	G30410.2	Down	Up	Chr2g0311311	NA	NA	NA	NA	NA	NA	A0A072V9F4	Transmembrane protein putative
Saranac	G32813.19	Down	Up	Chr3g0100731	NA	NA	NA	NA	NA	NA	G7J1G7	NB-ARC domain disease resistance protein
Saranac	G36727.7	Down	Up	Chr2g0285851	NA	NA	NA	NA	NA	NA	A0A1S3UYM4	F-box/kelch-repeat protein At1g22040
Saranac	G36742.112	Down	Up	Chr3g0080001	NA	NA	NA	NA	NA	NA	UPI001246FD05	uncharacterized WD repeat-containing protein C2A9.03-like isoform X1

Saranac	G37068.48	Down	Up	Chr3g0121581	NA	NA	NA	NA	NA	NA	A0A444YLY4	Cysteine desulfurase (EC 2.8.1.7)
Saranac	G41155.113	Down	Up	Chr4g0053671	NA	NA	NA	NA	NA	NA	J7H846	Delta-1-pyrroline-5-carboxylate synthase [Includes: Glutamate 5-kinase (GK) (EC 2.7.2.11) (Gamma-glutamyl kinase); Gamma-glutamyl phosphate reductase (GPR) (EC 1.2.1.41) (Glutamate-5-semialdehyde dehydrogenase) (Glutamyl-gamma-semialdehyde dehydrogenase)]
Saranac	G42730.5	Down	Up	Chr4g0048991	NA	NA	NA	NA	NA	NA	A0A2P5W636	Uncharacterized protein
Saranac	G43156.2	Down	Up	Chr5g0433661	NA	NA	NA	NA	NA	NA	A0A396GCX2	Ubiquitin-40S ribosomal protein S27a
Saranac	G44624.56	Down	Up	Chr3g0112411	NA	NA	NA	NA	NA	NA	J7H846	Delta-1-pyrroline-5-carboxylate synthase [Includes: Glutamate 5-kinase (GK) (EC 2.7.2.11) (Gamma-glutamyl kinase); Gamma-glutamyl phosphate reductase (GPR) (EC 1.2.1.41) (Glutamate-5-semialdehyde dehydrogenase) (Glutamyl-gamma-semialdehyde dehydrogenase)]
Saranac	G45232.10	Down	Up	Chr8g0391291	NA	NA	NA	NA	NA	NA	A0A5N6QH28	Pectinesterase (EC 3.1.1.11)
Saranac	G47938.151	Down	Up	Chr4g0053671	NA	NA	NA	NA	NA	NA	J7H846	Delta-1-pyrroline-5-carboxylate synthase [Includes: Glutamate 5-kinase (GK) (EC 2.7.2.11) (Gamma-glutamyl kinase); Gamma-glutamyl phosphate reductase (GPR) (EC 1.2.1.41) (Glutamate-5-semialdehyde dehydrogenase) (Glutamyl-gamma-semialdehyde dehydrogenase)]
Saranac	G49731.1	Down	Up	Chr4g0045531	NA	NA	NA	NA	NA	NA	UPI0010171FF7	uncharacterized protein LOC114176036 isoform X4
Saranac	G50188.6	Down	Up	Chr4g0038001	NA	NA	NA	NA	NA	NA	UPI000A2B5ADD	uncharacterized protein LOC107628723 isoform X4;uncharacterized protein LOC112737074 isoform X4
Saranac	G5163.4	Down	Up	Chr6g0458281	NA	NA	NA	NA	NA	NA	UPI00098D781D	BAG family molecular chaperone regulator 3 isoform X1
Saranac	G51993.24	Down	Up	Chr4g0040751	NA	NA	NA	NA	NA	NA	A0A2N9EP18	Acyl-coenzyme A oxidase
Saranac	G55755.3	Down	Up	Chr6g0453701	NA	NA	NA	NA	NA	NA	A0A396HE59	Uncharacterized protein
Saranac	G67686.4	Down	Up	Chr3g0140421	NA	NA	NA	NA	NA	NA	A0A2N9GB46	Uncharacterized protein
Saranac	G68894.9	Down	Up	Chr3g0109821	NAC	NA	NA	NA	NA	NA	UPI000788FA87	NAC transcription factor 29
Saranac	G73897.10	Down	Up	Chr2g0298401	NA	NA	NA	NA	NA	NA	UPI00077EC9CC	NA
Saranac	G74565.27	Down	Up	Chr7g0253361	NA	NA	NA	NA	NA	NA	I3T7E2	Uncharacterized protein
Saranac	G75882.36	Down	Up	Chr6g0479981	NA	NA	NA	NA	NA	NA	UPI0007871FF8	probable aldo-keto reductase 2
Saranac	G80057.12	Down	Up	Chr7g0264931	NA	NA	NA	NA	NA	NA	UPI00098D797B	zinc finger protein BRUTUS-like At1g18910 isoform X1
Saranac	G83299.9	Down	Up	Chr5g0399251	NA	NA	NA	NA	NA	NA	A0A0R0HJQ2	Protein transport protein sec16
Saranac	G84636.5	Down	Up	Chr8g0347711	NA	NA	NA	NA	NA	NA	A0A2R6QJN0	F-box/LRR-repeat protein
Saranac	G85143.1	Down	Up	Chr1g0187811	NA	NA	NA	NA	NA	NA	UPI00098DA204	1-aminocyclopropane-1-carboxylate oxidase homolog 1
Saranac	G87223.12	Down	Up	Chr5g0417001	NAC	NA	NA	NA	NA	NA	UPI000787145D	NAC domain-containing protein 72 isoform X2
Saranac	G87382.2	Down	Up	Chr3g0128461	NA	NA	NA	NA	NA	NA	A0A5P8TY68	Cytochrome P450 72A141
Saranac	G88105.11	Down	Up	Chr8g0337071	NA	NA	NA	NA	NA	NA	UPI000787ED3C	uncharacterized protein LOC107472603
Saranac	G89302.7	Down	Up	Chr8g0384361	NA	NA	NA	NA	NA	NA	UPI000B781D2C	histone deacetylase 5-like isoform X3
Saranac	G13423.5	Up	Up	Chr2g0279771	NA	NA	NA	NA	NA	NA	UPI000DEC61EF	myosin-binding protein 7
Saranac	G54617.13	Up	Up	Chr5g0401551	NA	NA	NA	NA	NA	NA	A0A067GK07	Uncharacterized protein
Saranac	G58231.12	Up	Up	Chr5g0420191	NA	NA	NA	NA	NA	NA	UPI000A2C2D4A	uncharacterized protein LOC107489508 isoform X3
Saranac	G65092.3	Up	Up	Chr6g0448891	NA	NA	NA	NA	NA	NA	A0A2N9J616	Uncharacterized protein
Saranac	G67364.5	Up	Up	Chr8g0353621	NA	NA	NA	AGC_MAST	NA	NA	UPI0010A2B78E	probable serine/threonine protein kinase IREH1 isoform X2
Saranac	G73113.73	Up	Up	Chr7g0219001	NA	NA	NA	NA	NA	NA	UPI00092E7BCC	protein RIK isoform X3
Saranac	G74317.24	Up	Up	Chr1g0159641	NA	NA	NA	NA	NA	NA	A0A392UBI8	MEI1 protein (Fragment)
Saranac	G88275.4	Up	Up	Chr4g0017231	NA	NA	NA	NA	lncRNA	NA	A0A444XF69	UMP-CMP kinase (EC 2.7.4.14) (Deoxycytidylate kinase) (CK) (dCMP kinase) (Uridine monophosphate/cytidine monophosphate kinase) (UMP/CMP kinase) (UMP/CMPK)

Supplementary Table 14. Transcription factors(TF) , protein kinases (PK), transcription regulators (TR) and lncRNAs in isoforms of modules significatively detected in Saranac, PI467895 and Wilson under salt (SS) or drought stress (DS).

Module	gene_id	isoform	treatment	family	class	Module	gene_id	isoform	treatment	family	class	Module	gene_id	isoform	treatment	family	class
skyblue3	G2821	G2821.33	PI467895_SS	C2H2	TF	violet	G4130	G4130.38	Saranac_SS	AGC	PK	mediumpurple3	G25354	G25354.1	Saranac_DS	SNF2	TR
skyblue3	G5837	G5837.2	PI467895_SS	B3	TF	violet	G10026	G10026.16	Saranac_SS	CAMK	PK	mediumpurple3	G25439	G25439.38	Saranac_DS	RLK-Pelle	PK
skyblue3	G16557	G16557.1	PI467895_SS	lncRNA	lncRNA	violet	G13153	G13153.3	Saranac_SS	PHD	TR	mediumpurple3	G25439	G25439.44	Saranac_DS	RLK-Pelle	PK
skyblue3	G27743	G27743.8	PI467895_SS	lncRNA	lncRNA	violet	G16540	G16540.12	Saranac_SS	TKL-Pl-6	PK	mediumpurple3	G25516	G25516.59	Saranac_DS	HB-other	TF
skyblue3	G30071	G30071.56	PI467895_SS	RLK-Pelle	PK	violet	G17256	G17256.6	Saranac_SS	CMGC	PK	mediumpurple3	G27051	G27051.1	Saranac_DS	MYB_relatec	TF
skyblue3	G32927	G32927.2	PI467895_SS	lncRNA	lncRNA	violet	G21125	G21125.2	Saranac_SS	lncRNA	lncRNA	mediumpurple3	G34944	G34944.5	Saranac_DS	RLK-Pelle	PK
skyblue3	G35525	G35525.1	PI467895_SS	lncRNA	lncRNA	violet	G22286	G22286.19	Saranac_SS	CAMTA	TF	mediumpurple3	G36366	G36366.3	Saranac_DS	lncRNA	lncRNA
skyblue3	G37571	G37571.4	PI467895_SS	CAMK	PK	violet	G23251	G23251.1	Saranac_SS	CAMK	PK	mediumpurple3	G37192	G37192.12	Saranac_DS	HD-ZIP	TF
skyblue3	G37771	G37771.2	PI467895_SS	CO-like	TF	violet	G28809	G28809.3	Saranac_SS	MYB	TF	mediumpurple3	G38620	G38620.30	Saranac_DS	lncRNA	lncRNA
skyblue3	G45411	G45411.31	PI467895_SS	bHLH	TF	violet	G30569	G30569.6	Saranac_SS	RLK-Pelle	PK	mediumpurple3	G39163	G39163.48	Saranac_DS	ARF	TF
skyblue3	G51123	G51123.2	PI467895_SS	lncRNA	lncRNA	violet	G36273	G36273.9	Saranac_SS	GNAT	TR	mediumpurple3	G40505	G40505.1	Saranac_DS	lncRNA	lncRNA
skyblue3	G56778	G56778.1	PI467895_SS	NF-YB	TF	violet	G38107	G38107.2	Saranac_SS	MYB	TF	mediumpurple3	G49979	G49979.1	Saranac_DS	lncRNA	lncRNA
skyblue3	G59115	G59115.16	PI467895_SS	CAMK	PK	violet	G39703	G39703.1	Saranac_SS	WRKY	TF	mediumpurple3	G50930	G50930.13	Saranac_DS	lncRNA	lncRNA
skyblue3	G59473	G59473.1	PI467895_SS	SWI/SNF-BA	TR	violet	G48713	G48713.17	Saranac_SS	lncRNA	lncRNA	mediumpurple3	G57396	G57396.23	Saranac_DS	G2-like	TF
skyblue3	G69075	G69075.2	PI467895_SS	Trihelix	TF	violet	G51171	G51171.13	Saranac_SS	RLK-Pelle	PK	mediumpurple3	G60442	G60442.8	Saranac_DS	bHLH	TF
skyblue3	G69252	G69252.9	PI467895_SS	MIKC_MADS	TF	violet	G51171	G51171.14	Saranac_SS	RLK-Pelle	PK	mediumpurple3	G60463	G60463.2	Saranac_DS	lncRNA	lncRNA
skyblue3	G76155	G76155.9	PI467895_SS	RLK-Pelle	PK	violet	G53103	G53103.1	Saranac_SS	NAC	TF	mediumpurple3	G61472	G61472.4	Saranac_DS	lncRNA	lncRNA
skyblue3	G86227	G86227.4	PI467895_SS	TRAF	TR	violet	G56495	G56495.1	Saranac_SS	TLK	PK	mediumpurple3	G63673	G63673.6	Saranac_DS	Dof	TF
skyblue3	G90953	G90953.1	PI467895_SS	ERF	TF	violet	G56905	G56905.40	Saranac_SS	CAMK	PK	mediumpurple3	G64738	G64738.20	Saranac_DS	MYB_relatec	TF
thistle1	G5833	G5833.26	PI467895_SS	B3	TF	violet	G56905	G56905.55	Saranac_SS	CAMK	PK	mediumpurple3	G66357	G66357.34	Saranac_DS	MYB_relatec	TF
thistle1	G8485	G8485.66	PI467895_SS	PHD	TR	violet	G65952	G65952.4	Saranac_SS	RLK-Pelle	PK	mediumpurple3	G66666	G66666.4	Saranac_DS	RLK-Pelle	PK
thistle1	G13060	G13060.49	PI467895_SS	CK1	PK	violet	G70628	G70628.11	Saranac_SS	GeBP	TF	mediumpurple3	G69346	G69346.4	Saranac_DS	CAMK	PK
thistle1	G22773	G22773.3	PI467895_SS	ARF	TF	violet	G72858	G72858.9	Saranac_SS	TCP	TF	mediumpurple3	G77871	G77871.15	Saranac_DS	CAMK	PK
thistle1	G24477	G24477.1	PI467895_SS	lncRNA	lncRNA	violet	G73346	G73346.13	Saranac_SS	NAC	TF	mediumpurple3	G80213	G80213.3	Saranac_DS	MYB_relatec	TF
thistle1	G28971	G28971.5	PI467895_SS	FAR1	TF	violet	G74379	G74379.38	Saranac_SS	bZIP	TF	mediumpurple3	G83485	G83485.28	Saranac_DS	lncRNA	lncRNA
thistle1	G31699	G31699.1	PI467895_SS	CMGC	PK	violet	G75470	G75470.1	Saranac_SS	lncRNA	lncRNA	mediumpurple3	G88517	G88517.1	Saranac_DS	CAMK	PK
thistle1	G31801	G31801.1	PI467895_SS	WOX	TF	violet	G80949	G80949.31	Saranac_SS	FAR1	TF	mediumpurple3	G88520	G88520.4	Saranac_DS	CAMK	PK
thistle1	G37163	G37163.36	PI467895_SS	RLK-Pelle	PK	violet	G86283	G86283.5	Saranac_SS	MIKC_MAI	TF	mediumpurple3	G89583	G89583.16	Saranac_DS	MYB_relatec	TF
thistle1	G42494	G42494.2	PI467895_SS	C3H	TF	mediumorchi	G1051	G1051.5	Saranac_SS	CMGC	PK	indianred4	G16937	G16937.5	Saranac_DS	lncRNA	lncRNA
thistle1	G42915	G42915.9	PI467895_SS	CAMK	PK	mediumorchi	G2148	G2148.1	Saranac_SS	HD-ZIP	TF	indianred4	G18621	G18621.1	Saranac_DS	Others	TR
thistle1	G48769	G48769.4	PI467895_SS	CAMK	PK	mediumorchi	G34166	G34166.21	Saranac_SS	IWS1	TR	indianred4	G18621	G18621.5	Saranac_DS	Others	TR
thistle1	G60110	G60110.2	PI467895_SS	GATA	TF	mediumorchi	G38480	G38480.59	Saranac_SS	RLK-Pelle	PK	indianred4	G23793	G23793.1	Saranac_DS	Others	TR
thistle1	G68066	G68066.6	PI467895_SS	SBP	TF	mediumorchi	G39724	G39724.22	Saranac_SS	G2-like	TF	indianred4	G27051	G27051.3	Saranac_DS	MYB_relatec	TF
thistle1	G73663	G73663.14	PI467895_SS	CMGC	PK	mediumorchi	G41691	G41691.1	Saranac_SS	lncRNA	lncRNA	indianred4	G30121	G30121.8	Saranac_DS	MYB_relatec	TF
thistle1	G78633	G78633.2	PI467895_SS	TCP	TF	mediumorchi	G41948	G41948.38	Saranac_SS	RLK-Pelle	PK	indianred4	G30456	G30456.3	Saranac_DS	lncRNA	lncRNA
thistle1	G86109	G86109.33	PI467895_SS	CAMK	PK	mediumorchi	G53650	G53650.3	Saranac_SS	lncRNA	lncRNA	indianred4	G55323	G55323.41	Saranac_DS	lncRNA	lncRNA
midnightblue	G3984	G3984.17	PI467895_SS	MYB_related	TF	mediumorchi	G63389	G63389.5	Saranac_SS	RLK-Pelle	PK	indianred4	G55701	G55701.21	Saranac_DS	Group-Pl-4	PK
midnightblue	G5839	G5839.2	PI467895_SS	B3	TF	mediumorchi	G83690	G83690.2	Saranac_SS	HMG	TR	indianred4	G59334	G59334.1	Saranac_DS	NF-YB	TF
midnightblue	G7314	G7314.21	PI467895_SS	CK1	PK	mediumorchi	G86938	G86938.1	Saranac_SS	lncRNA	lncRNA	indianred4	G72107	G72107.14	Saranac_DS	CAMK	PK
midnightblue	G8564	G8564.6	PI467895_SS	lncRNA	lncRNA	plum	G17144	G17144.1	Saranac_SS	mTERF	TR	indianred4	G88519	G88519.6	Saranac_DS	CAMK	PK
midnightblue	G16791	G16791.1	PI467895_SS	lncRNA	lncRNA	plum	G20486	G20486.3	Saranac_SS	lncRNA	lncRNA	blue	G534	G534.8	Saranac_DS&SS	CAMK	PK
midnightblue	G26476	G26476.1	PI467895_SS	lncRNA	lncRNA	plum	G28888	G28888.4	Saranac_SS	GATA	TF	blue	G1388	G1388.3	Saranac_DS&SS	lncRNA	lncRNA
midnightblue	G28719	G28719.4	PI467895_SS	RLK-Pelle	PK	plum	G30612	G30612.8	Saranac_SS	lncRNA	lncRNA	blue	G3984	G3984.38	Saranac_DS&SS	MYB_relatec	TF
midnightblue	G30809	G30809.3	PI467895_SS	C2H2	TF	plum	G37797	G37797.28	Saranac_SS	ARR-B	TF	blue	G7691	G7691.9	Saranac_DS&SS	AUX/IAA	TR
midnightblue	G35250	G35250.2	PI467895_SS	lncRNA	lncRNA	plum	G42377	G42377.7	Saranac_SS	lncRNA	lncRNA	blue	G7824	G7824.5	Saranac_DS&SS	MYB_relatec	TF
midnightblue	G36550	G36550.14	PI467895_SS	lncRNA	lncRNA	plum	G43228	G43228.3	Saranac_SS	lncRNA	lncRNA	blue	G7837	G7837.1	Saranac_DS&SS	AUX/IAA	TR
midnightblue	G37987	G37987.1	PI467895_SS	RLK-Pelle	PK	plum	G54605	G54605.9	Saranac_SS	CMGC	PK	blue	G7888	G7888.44	Saranac_DS&SS	CMGC	PK
midnightblue	G37987	G37987.2	PI467895_SS	RLK-Pelle	PK	plum	G57659	G57659.2	Saranac_SS	SWI/SNF-B/	TR	blue	G8439	G8439.33	Saranac_DS&SS	SOH1	TR
midnightblue	G38741	G38741.11	PI467895_SS	STE	PK	plum	G58786	G58786.5	Saranac_SS	RLK-Pelle	PK	blue	G10663	G10663.9	Saranac_DS&SS	TRAF	TR
midnightblue	G47808	G47808.15	PI467895_SS	GRAS	TF	plum	G68095	G68095.5	Saranac_SS	C3H	TF	blue	G10663	G10663.11	Saranac_DS&SS	TRAF	TR
midnightblue	G51663	G51663.8	PI467895_SS	SNF2	TR	plum	G69368	G69368.6	Saranac_SS	lncRNA	lncRNA	blue	G12336	G12336.61	Saranac_DS&SS	GNAT	TR
midnightblue	G59026	G59026.1	PI467895_SS	lncRNA	lncRNA	plum	G69865	G69865.16	Saranac_SS	GRAS	TF	blue	G12336	G12336.61	Saranac_DS&SS	lncRNA	lncRNA
midnightblue	G62524	G62524.1	PI467895_SS	lncRNA	lncRNA	plum	G91029	G91029.2	Saranac_SS	MYB_relatec	TF	blue	G12671	G12671.13	Saranac_DS&SS	PHD	TR
midnightblue	G67959	G67959.1	PI467895_SS	NF-YC	TF	navajowhite2	G4615	G4615.21	Saranac_SS	CMGC	PK	blue	G12926	G12926.5	Saranac_DS&SS	SBP	TF
midnightblue	G68304	G68304.2	PI467895_SS	S1Fa-like	TF	navajowhite2	G4615	G4615.21	Saranac_SS	C3H	TF	blue	G12926	G12926.29	Saranac_DS&SS	SBP	TF
midnightblue	G68789	G68789.56	PI467895_SS	G2-like	TF	navajowhite2	G9221	G9221.7	Saranac_SS	bHLH	TF	blue	G13250	G13250.1	Saranac_DS&SS	bZIP	TF
midnightblue	G76288	G76288.1	PI467895_SS	GNAT	TR	navajowhite2	G9933	G9933.1	Saranac_SS	AGC	PK	blue	G15713	G15713.10	Saranac_DS&SS	MYB	TF

midnightblue	G78051	G78051.6	PI467895_SS	lncRNA	lncRNA
midnightblue	G81468	G81468.3	PI467895_SS	lncRNA	lncRNA
midnightblue	G83107	G83107.7	PI467895_SS	MYB_related	TF
yellow	G1455	G1455.5	PI467895_SS	lncRNA	lncRNA
yellow	G4907	G4907.9	PI467895_SS	GATA	TF
yellow	G5042	G5042.35	PI467895_SS	bZIP	TF
yellow	G9623	G9623.1	PI467895_SS	RLK-Pelle	PK
yellow	G10378	G10378.1	PI467895_SS	lncRNA	lncRNA
yellow	G11051	G11051.12	PI467895_SS	lncRNA	lncRNA
yellow	G13153	G13153.1	PI467895_SS	PHD	TR
yellow	G13896	G13896.2	PI467895_SS	TRAF	TR
yellow	G14001	G14001.62	PI467895_SS	AGC	PK
yellow	G14019	G14019.3	PI467895_SS	AUX/IAA	TR
yellow	G17171	G17171.1	PI467895_SS	lncRNA	lncRNA
yellow	G17656	G17656.54	PI467895_SS	TKL-Pl-5	PK
yellow	G18751	G18751.1	PI467895_SS	MBF1	TR
yellow	G19081	G19081.15	PI467895_SS	TKL-Pl-6	PK
yellow	G21551	G21551.1	PI467895_SS	lncRNA	lncRNA
yellow	G23235	G23235.5	PI467895_SS	ERF	TF
yellow	G23369	G23369.4	PI467895_SS	lncRNA	lncRNA
yellow	G25355	G25355.7	PI467895_SS	RLK-Pelle	PK
yellow	G25450	G25450.2	PI467895_SS	Trihelix	TF
yellow	G27373	G27373.2	PI467895_SS	C2H2	TF
yellow	G27500	G27500.1	PI467895_SS	Others	TR
yellow	G28472	G28472.14	PI467895_SS	ARR-B	TF
yellow	G28970	G28970.6	PI467895_SS	lncRNA	lncRNA
yellow	G30796	G30796.6	PI467895_SS	HD-ZIP	TF
yellow	G34943	G34943.13	PI467895_SS	RLK-Pelle	PK
yellow	G35623	G35623.2	PI467895_SS	NAK	PK
yellow	G36967	G36967.29	PI467895_SS	WNK	PK
yellow	G38488	G38488.1	PI467895_SS	TKL-Pl-4	PK
yellow	G39199	G39199.16	PI467895_SS	GRAS	TF
yellow	G39308	G39308.15	PI467895_SS	PHD	TR
yellow	G40405	G40405.2	PI467895_SS	CAMK	PK
yellow	G41391	G41391.4	PI467895_SS	lncRNA	lncRNA
yellow	G44330	G44330.2	PI467895_SS	lncRNA	lncRNA
yellow	G46742	G46742.43	PI467895_SS	HB-other	TF
yellow	G51847	G51847.76	PI467895_SS	CAMK	PK
yellow	G53750	G53750.5	PI467895_SS	ERF	TF
yellow	G54631	G54631.4	PI467895_SS	MYB	TF
yellow	G55869	G55869.14	PI467895_SS	CAMK	PK
yellow	G56138	G56138.6	PI467895_SS	CAMK	PK
yellow	G56160	G56160.5	PI467895_SS	ERF	TF
yellow	G56520	G56520.10	PI467895_SS	FAR1	TF
yellow	G62008	G62008.1	PI467895_SS	GRAS	TF
yellow	G62009	G62009.2	PI467895_SS	WRKY	TF
yellow	G62051	G62051.8	PI467895_SS	RLK-Pelle	PK
yellow	G64149	G64149.24	PI467895_SS	lncRNA	lncRNA
yellow	G64707	G64707.1	PI467895_SS	BBR-BPC	TF
yellow	G66007	G66007.13	PI467895_SS	CAMK	PK
yellow	G67730	G67730.1	PI467895_SS	lncRNA	lncRNA
yellow	G67902	G67902.37	PI467895_SS	MYB	TF
yellow	G68501	G68501.11	PI467895_SS	lncRNA	lncRNA
yellow	G69075	G69075.3	PI467895_SS	Trihelix	TF
yellow	G71587	G71587.12	PI467895_SS	SET	TR
yellow	G72638	G72638.7	PI467895_SS	GRAS	TF
yellow	G73663	G73663.21	PI467895_SS	CMGC	PK
yellow	G73663	G73663.24	PI467895_SS	CMGC	PK
yellow	G76919	G76919.21	PI467895_SS	CK1	PK
yellow	G77153	G77153.2	PI467895_SS	CAMK	PK

navajowhite2	G10812	G10812.14	Saranac_SS	Jumonji	TR
navajowhite2	G20065	G20065.1	Saranac_SS	lncRNA	lncRNA
navajowhite2	G20808	G20808.1	Saranac_SS	MYB_relatec	TF
navajowhite2	G22500	G22500.13	Saranac_SS	G2-like	TF
navajowhite2	G22906	G22906.4	Saranac_SS	C3H	TF
navajowhite2	G23767	G23767.4	Saranac_SS	lncRNA	lncRNA
navajowhite2	G24809	G24809.1	Saranac_SS	HB-PHD	TF
navajowhite2	G25233	G25233.3	Saranac_SS	lncRNA	lncRNA
navajowhite2	G30714	G30714.1	Saranac_SS	RLK-Pelle	PK
navajowhite2	G32989	G32989.3	Saranac_SS	lncRNA	lncRNA
navajowhite2	G36243	G36243.1	Saranac_SS	lncRNA	lncRNA
navajowhite2	G39563	G39563.44	Saranac_SS	CMGC	PK
navajowhite2	G47469	G47469.12	Saranac_SS	lncRNA	lncRNA
navajowhite2	G50915	G50915.1	Saranac_SS	GNAT	TR
navajowhite2	G57832	G57832.30	Saranac_SS	AUX/IAA	TR
navajowhite2	G70051	G70051.18	Saranac_SS	SBP	TF
navajowhite2	G81783	G81783.11	Saranac_SS	ERF	TF
navajowhite2	G91241	G91241.1	Saranac_SS	lncRNA	lncRNA
darkolivegre	G7878	G7878.1	Wilson_DS	SBP	TF
darkolivegre	G11097	G11097.64	Wilson_DS	SBP	TF
darkolivegre	G12189	G12189.2	Wilson_DS	HMG	TR
darkolivegre	G14329	G14329.19	Wilson_DS	CMGC	PK
darkolivegre	G17337	G17337.2	Wilson_DS	TRAF	TR
darkolivegre	G19081	G19081.3	Wilson_DS	TKL-Pl-6	PK
darkolivegre	G23332	G23332.8	Wilson_DS	lncRNA	lncRNA
darkolivegre	G23659	G23659.9	Wilson_DS	Dof	TF
darkolivegre	G29416	G29416.3	Wilson_DS	GNAT	TR
darkolivegre	G32972	G32972.3	Wilson_DS	GNAT	TR
darkolivegre	G40969	G40969.1	Wilson_DS	lncRNA	lncRNA
darkolivegre	G41175	G41175.1	Wilson_DS	lncRNA	lncRNA
darkolivegre	G46520	G46520.15	Wilson_DS	G2-like	TF
darkolivegre	G48259	G48259.2	Wilson_DS	lncRNA	lncRNA
darkolivegre	G53644	G53644.6	Wilson_DS	MYB	TF
darkolivegre	G67813	G67813.31	Wilson_DS	CAMK	PK
darkolivegre	G68446	G68446.6	Wilson_DS	bHLH	TF
darkolivegre	G75082	G75082.3	Wilson_DS	CAMK	PK
darkolivegre	G77844	G77844.12	Wilson_DS	RLK-Pelle	PK
darkolivegre	G80765	G80765.22	Wilson_DS	AUX/IAA	TR
darkolivegre	G83410	G83410.7	Wilson_DS	CMGC	PK
darkolivegre	G83755	G83755.17	Wilson_DS	bZIP	TF
brown	G876	G876.1	Wilson_DS	GATA	TF
brown	G2754	G2754.5	Wilson_DS	Others	TR
brown	G3307	G3307.14	Wilson_DS	C2H2	TF
brown	G5834	G5834.1	Wilson_DS	B3	TF
brown	G9074	G9074.32	Wilson_DS	HMG	TR
brown	G9835	G9835.9	Wilson_DS	TCP	TF
brown	G10534	G10534.25	Wilson_DS	SET	TR
brown	G11127	G11127.2	Wilson_DS	bZIP	TF
brown	G11972	G11972.16	Wilson_DS	TCP	TF
brown	G14329	G14329.29	Wilson_DS	CMGC	PK
brown	G17105	G17105.1	Wilson_DS	mTERF	TR
brown	G17693	G17693.1	Wilson_DS	CMGC	PK
brown	G18719	G18719.5	Wilson_DS	C2H2	TF
brown	G19026	G19026.1	Wilson_DS	lncRNA	lncRNA
brown	G19484	G19484.1	Wilson_DS	bZIP	TF
brown	G20056	G20056.44	Wilson_DS	CMGC	PK
brown	G20115	G20115.2	Wilson_DS	lncRNA	lncRNA
brown	G21441	G21441.5	Wilson_DS	PHD	TR
brown	G21687	G21687.22	Wilson_DS	TKL-Pl-6	PK
brown	G23900	G23900.3	Wilson_DS	G2-like	TF

blue	G15813	G15813.4	Saranac_DS&SS	lncRNA	lncRNA
blue	G18319	G18319.15	Saranac_DS&SS	bHLH	TF
blue	G18438	G18438.14	Saranac_DS&SS	GATA	TF
blue	G21140	G21140.1	Saranac_DS&SS	lncRNA	lncRNA
blue	G22352	G22352.1	Saranac_DS&SS	CMGC	PK
blue	G22844	G22844.1	Saranac_DS&SS	Trihelix	TF
blue	G24630	G24630.27	Saranac_DS&SS	SBP	TF
blue	G25516	G25516.72	Saranac_DS&SS	HB-other	TF
blue	G25724	G25724.8	Saranac_DS&SS	CAMTA	TF
blue	G26210	G26210.4	Saranac_DS&SS	lncRNA	lncRNA
blue	G26823	G26823.1	Saranac_DS&SS	NF-YA	TF
blue	G28162	G28162.21	Saranac_DS&SS	CAMK	PK
blue	G28415	G28415.12	Saranac_DS&SS	lncRNA	lncRNA
blue	G28534	G28534.71	Saranac_DS&SS	LUG	TR
blue	G28720	G28720.4	Saranac_DS&SS	RLK-Pelle	PK
blue	G29367	G29367.15	Saranac_DS&SS	C3H	TF
blue	G29416	G29416.4	Saranac_DS&SS	GNAT	TR
blue	G29603	G29603.4	Saranac_DS&SS	lncRNA	lncRNA
blue	G29721	G29721.1	Saranac_DS&SS	lncRNA	lncRNA
blue	G30392	G30392.2	Saranac_DS&SS	ERF	TF
blue	G30392	G30392.4	Saranac_DS&SS	ERF	TF
blue	G30392	G30392.6	Saranac_DS&SS	ERF	TF
blue	G30392	G30392.8	Saranac_DS&SS	ERF	TF
blue	G30392	G30392.8	Saranac_DS&SS	lncRNA	lncRNA
blue	G31874	G31874.8	Saranac_DS&SS	FAR1	TF
blue	G34080	G34080.4	Saranac_DS&SS	Dof	TF
blue	G34138	G34138.21	Saranac_DS&SS	NAC	TF
blue	G37751	G37751.7	Saranac_DS&SS	lncRNA	lncRNA
blue	G37869	G37869.41	Saranac_DS&SS	LUG	TR
blue	G39363	G39363.18	Saranac_DS&SS	CMGC	PK
blue	G43487	G43487.14	Saranac_DS&SS	bZIP	TF
blue	G43487	G43487.18	Saranac_DS&SS	bZIP	TF
blue	G43505	G43505.2	Saranac_DS&SS	lncRNA	lncRNA
blue	G43517	G43517.9	Saranac_DS&SS	bZIP	TF
blue	G50283	G50283.7	Saranac_DS&SS	bZIP	TF
blue	G50358	G50358.2	Saranac_DS&SS	bZIP	TF
blue	G53092	G53092.17	Saranac_DS&SS	RLK-Pelle	PK
blue	G54079	G54079.11	Saranac_DS&SS	lncRNA	lncRNA
blue	G55123	G55123.1	Saranac_DS&SS	bZIP	TF
blue	G55925	G55925.1	Saranac_DS&SS	lncRNA	lncRNA
blue	G58409	G58409.3	Saranac_DS&SS	ERF	TF
blue	G58476	G58476.8	Saranac_DS&SS	GATA	TF
blue	G59967	G59967.8	Saranac_DS&SS	HSF	TF
blue	G60003	G60003.9	Saranac_DS&SS	RLK-Pelle	PK
blue	G60033	G60033.8	Saranac_DS&SS	BES1	TF
blue	G60033	G60033.11	Saranac_DS&SS	BES1	TF
blue	G61353	G61353.2	Saranac_DS&SS	CAMK	PK
blue	G62086	G62086.53	Saranac_DS&SS	TKL-Pl-4	PK
blue	G62086	G62086.59	Saranac_DS&SS	TKL-Pl-4	PK
blue	G63624	G63624.10	Saranac_DS&SS	RLK-Pelle	PK
blue	G64086	G64086.13	Saranac_DS&SS	MYB	TF
blue	G64810	G64810.3	Saranac_DS&SS	lncRNA	lncRNA
blue	G68581	G68581.2	Saranac_DS&SS	C3H	TF
blue	G69075	G69075.4	Saranac_DS&SS	Trihelix	TF
blue	G70174	G70174.3	Saranac_DS&SS	Trihelix	TF
blue	G70509	G70509.5	Saranac_DS&SS	lncRNA	lncRNA
blue	G71119	G71119.2	Saranac_DS&SS	S1Fa-like	TF
blue	G71732	G71732.12	Saranac_DS&SS	lncRNA	lncRNA
blue	G73474	G73474.2	Saranac_DS&SS	G2-like	TF
blue	G73602	G73602.36	Saranac_DS&SS	PHD	TR

yellow	G77251	G77251.4	PI467895_SS	bZIP	TF	brown	G25233	G25233.5	Wilson_DS	lncRNA	lncRNA	blue	G74445	G74445.5	Saranac_DS&SS	bZIP	TF
yellow	G78207	G78207.15	PI467895_SS	LUG	TR	brown	G26078	G26078.2	Wilson_DS	lncRNA	lncRNA	blue	G76540	G76540.23	Saranac_DS&SS	CMGC	PK
yellow	G78434	G78434.3	PI467895_SS	lncRNA	lncRNA	brown	G28809	G28809.1	Wilson_DS	MYB	TF	blue	G78965	G78965.6	Saranac_DS&SS	RLK-Pelle	PK
yellow	G80075	G80075.4	PI467895_SS	TAZ	TR	brown	G30071	G30071.57	Wilson_DS	RLK-Pelle	PK	blue	G79144	G79144.27	Saranac_DS&SS	TKL-Pl-4	PK
yellow	G80220	G80220.3	PI467895_SS	ERF	TF	brown	G30780	G30780.8	Wilson_DS	RLK-Pelle	PK	blue	G81663	G81663.6	Saranac_DS&SS	lncRNA	lncRNA
yellow	G80220	G80220.3	PI467895_SS	lncRNA	lncRNA	brown	G31226	G31226.17	Wilson_DS	CAMK	PK	blue	G81783	G81783.12	Saranac_DS&SS	ERF	TF
yellow	G80387	G80387.8	PI467895_SS	MYB	TF	brown	G32278	G32278.1	Wilson_DS	lncRNA	lncRNA	blue	G81955	G81955.1	Saranac_DS&SS	MED6	TR
yellow	G82499	G82499.10	PI467895_SS	STE	PK	brown	G33279	G33279.8	Wilson_DS	Others	TR	blue	G82006	G82006.5	Saranac_DS&SS	lncRNA	lncRNA
yellow	G83071	G83071.16	PI467895_SS	HD-ZIP	TF	brown	G36179	G36179.8	Wilson_DS	GATA	TF	blue	G82458	G82458.21	Saranac_DS&SS	lncRNA	lncRNA
yellow	G83321	G83321.3	PI467895_SS	ERF	TF	brown	G36592	G36592.1	Wilson_DS	GeBP	TF	blue	G83690	G83690.4	Saranac_DS&SS	HMG	TR
yellow	G84905	G84905.18	PI467895_SS	lncRNA	lncRNA	brown	G36648	G36648.8	Wilson_DS	lncRNA	lncRNA	blue	G87242	G87242.23	Saranac_DS&SS	CMGC	PK
yellow	G86125	G86125.16	PI467895_SS	MYB_related	TF	brown	G38021	G38021.3	Wilson_DS	BES1	TF	blue	G87362	G87362.2	Saranac_DS&SS	bZIP	TF
yellow	G87820	G87820.6	PI467895_SS	ERF	TF	brown	G40268	G40268.4	Wilson_DS	lncRNA	lncRNA	blue	G89509	G89509.67	Saranac_DS&SS	CMGC	PK
yellow	G89035	G89035.23	PI467895_SS	MYB_related	TF	brown	G41957	G41957.15	Wilson_DS	RLK-Pelle	PK	blue	G90664	G90664.9	Saranac_DS&SS	M-type_MA	TF
yellow	G89432	G89432.9	PI467895_SS	TKL-Pl-4	PK	brown	G54299	G54299.3	Wilson_DS	lncRNA	lncRNA	floralwhite	G6061	G6061.5	Saranac_DS	CAMK	PK
yellow4	G6499	G6499.5	PI467895_SS	C3H	TF	brown	G66327	G66327.7	Wilson_DS	BBR-BPC	TF	floralwhite	G6344	G6344.1	Saranac_DS	GRAS	TF
yellow4	G7691	G7691.11	PI467895_SS	AUX/IAA	TR	brown	G67637	G67637.2	Wilson_DS	SCY1	PK	floralwhite	G6502	G6502.29	Saranac_DS	SBP	TF
yellow4	G12015	G12015.3	PI467895_SS	WOX	TF	brown	G71524	G71524.14	Wilson_DS	CAMK	PK	floralwhite	G7878	G7878.16	Saranac_DS	SBP	TF
yellow4	G12234	G12234.9	PI467895_SS	B3	TF	brown	G72082	G72082.7	Wilson_DS	RLK-Pelle	PK	floralwhite	G10894	G10894.62	Saranac_DS	bHLH	TF
yellow4	G20298	G20298.40	PI467895_SS	HB-other	TF	brown	G73473	G73473.4	Wilson_DS	MYB	TF	floralwhite	G12325	G12325.2	Saranac_DS	C2H2	TF
yellow4	G30455	G30455.4	PI467895_SS	lncRNA	lncRNA	brown	G74973	G74973.5	Wilson_DS	RLK-Pelle	PK	floralwhite	G12769	G12769.8	Saranac_DS	GRAS	TF
yellow4	G31226	G31226.19	PI467895_SS	CAMK	PK	brown	G76057	G76057.3	Wilson_DS	lncRNA	lncRNA	floralwhite	G17543	G17543.1	Saranac_DS	FAR1	TF
yellow4	G46848	G46848.1	PI467895_SS	GRAS	TF	brown	G81763	G81763.2	Wilson_DS	RLK-Pelle	PK	floralwhite	G17545	G17545.1	Saranac_DS	lncRNA	lncRNA
yellow4	G52501	G52501.6	PI467895_SS	RLK-Pelle	PK	brown	G83038	G83038.2	Wilson_DS	TCP	TF	floralwhite	G17697	G17697.3	Saranac_DS	CAMK	PK
yellow4	G59968	G59968.5	PI467895_SS	lncRNA	lncRNA	brown	G85196	G85196.3	Wilson_DS	AGC	PK	floralwhite	G20099	G20099.62	Saranac_DS	SNF2	TR
yellow4	G66131	G66131.5	PI467895_SS	C3H	TF	brown	G85600	G85600.1	Wilson_DS	lncRNA	lncRNA	floralwhite	G23085	G23085.22	Saranac_DS	CAMTA	TF
yellow4	G71262	G71262.10	PI467895_SS	bHLH	TF	brown	G86112	G86112.1	Wilson_DS	TCP	TF	floralwhite	G25526	G25526.2	Saranac_DS	ERF	TF
yellow4	G78146	G78146.20	PI467895_SS	MYB	TF	brown	G87112	G87112.1	Wilson_DS	Whirly	TF	floralwhite	G25582	G25582.10	Saranac_DS	SNF2	TR
yellow4	G81010	G81010.1	PI467895_SS	lncRNA	lncRNA	brown	G88880	G88880.8	Wilson_DS	lncRNA	lncRNA	floralwhite	G27915	G27915.1	Saranac_DS	lncRNA	lncRNA
paleturquoise	G10748	G10748.2	PI467895_SS	AGC	PK	brown	G90184	G90184.7	Wilson_DS	bHLH	TF	floralwhite	G28471	G28471.18	Saranac_DS	PHD	TR
paleturquoise	G11127	G11127.1	PI467895_SS	bZIP	TF	red	G374	G374.11	Wilson_DS	lncRNA	lncRNA	floralwhite	G29602	G29602.1	Saranac_DS	lncRNA	lncRNA
paleturquoise	G13535	G13535.3	PI467895_SS	AGC	PK	red	G889	G889.36	Wilson_DS	ARR-B	TF	floralwhite	G34619	G34619.1	Saranac_DS	C3H	TF
paleturquoise	G14329	G14329.9	PI467895_SS	CMGC	PK	red	G1055	G1055.15	Wilson_DS	CMGC	PK	floralwhite	G34619	G34619.3	Saranac_DS	C3H	TF
paleturquoise	G15281	G15281.6	PI467895_SS	WOX	TF	red	G3882	G3882.2	Wilson_DS	lncRNA	lncRNA	floralwhite	G34619	G34619.4	Saranac_DS	C3H	TF
paleturquoise	G25840	G25840.36	PI467895_SS	CMGC	PK	red	G5057	G5057.1	Wilson_DS	Trihelix	TF	floralwhite	G34903	G34903.34	Saranac_DS	HD-ZIP	TF
paleturquoise	G29998	G29998.1	PI467895_SS	RLK-Pelle	PK	red	G7878	G7878.8	Wilson_DS	SBP	TF	floralwhite	G48319	G48319.6	Saranac_DS	PHD	TR
paleturquoise	G32405	G32405.6	PI467895_SS	C3H	TF	red	G8485	G8485.4	Wilson_DS	PHD	TR	floralwhite	G49335	G49335.7	Saranac_DS	C3H	TF
paleturquoise	G33480	G33480.42	PI467895_SS	CMGC	PK	red	G10534	G10534.22	Wilson_DS	SET	TR	floralwhite	G55367	G55367.1	Saranac_DS	Others	TR
paleturquoise	G33873	G33873.3	PI467895_SS	C2H2	TF	red	G11096	G11096.5	Wilson_DS	RLK-Pelle	PK	floralwhite	G56035	G56035.27	Saranac_DS	B3	TF
paleturquoise	G37579	G37579.6	PI467895_SS	CAMK	PK	red	G11435	G11435.7	Wilson_DS	NAC	TF	floralwhite	G58829	G58829.19	Saranac_DS	lncRNA	lncRNA
paleturquoise	G38060	G38060.19	PI467895_SS	CMGC	PK	red	G12239	G12239.5	Wilson_DS	B3	TF	floralwhite	G60293	G60293.2	Saranac_DS	lncRNA	lncRNA
paleturquoise	G41495	G41495.8	PI467895_SS	WRKY	TF	red	G14329	G14329.31	Wilson_DS	CMGC	PK	floralwhite	G65222	G65222.6	Saranac_DS	IWS1	TR
paleturquoise	G42445	G42445.2	PI467895_SS	GRAS	TF	red	G14386	G14386.1	Wilson_DS	lncRNA	lncRNA	floralwhite	G67219	G67219.3	Saranac_DS	RLK-Pelle	PK
paleturquoise	G42445	G42445.4	PI467895_SS	GRAS	TF	red	G16151	G16151.2	Wilson_DS	C2H2	TF	floralwhite	G67927	G67927.1	Saranac_DS	GeBP	TF
paleturquoise	G44359	G44359.5	PI467895_SS	SNF2	TR	red	G16151	G16151.4	Wilson_DS	C2H2	TF	floralwhite	G71287	G71287.1	Saranac_DS	lncRNA	lncRNA
paleturquoise	G47221	G47221.2	PI467895_SS	lncRNA	lncRNA	red	G18043	G18043.2	Wilson_DS	C2H2	TF	floralwhite	G73402	G73402.36	Saranac_DS	Jumonji	TR
paleturquoise	G50930	G50930.16	PI467895_SS	lncRNA	lncRNA	red	G18710	G18710.2	Wilson_DS	lncRNA	lncRNA	floralwhite	G79064	G79064.5	Saranac_DS	NAC	TF
paleturquoise	G52830	G52830.2	PI467895_SS	lncRNA	lncRNA	red	G22411	G22411.43	Wilson_DS	NF-YA	TF	floralwhite	G79412	G79412.1	Saranac_DS	STE	PK
paleturquoise	G57367	G57367.7	PI467895_SS	RLK-Pelle	PK	red	G25852	G25852.9	Wilson_DS	FAR1	TF	floralwhite	G86245	G86245.49	Saranac_DS	CMGC	PK
paleturquoise	G57994	G57994.2	PI467895_SS	lncRNA	lncRNA	red	G28534	G28534.69	Wilson_DS	LUG	TR	floralwhite	G86445	G86445.1	Saranac_DS	bZIP	TF
paleturquoise	G60792	G60792.1	PI467895_SS	lncRNA	lncRNA	red	G31879	G31879.6	Wilson_DS	lncRNA	lncRNA	floralwhite	G89692	G89692.27	Saranac_DS	RLK-Pelle	PK
paleturquoise	G61620	G61620.12	PI467895_SS	ERF	TF	red	G35115	G35115.14	Wilson_DS	ARID	TR	floralwhite	G90509	G90509.7	Saranac_DS	MYB	TF
paleturquoise	G63564	G63564.15	PI467895_SS	HD-ZIP	TF	red	G36435	G36435.3	Wilson_DS	lncRNA	lncRNA	green	G749	G749.52	Saranac_DS	Others	TR
paleturquoise	G73663	G73663.1	PI467895_SS	CMGC	PK	red	G54424	G54424.10	Wilson_DS	lncRNA	lncRNA	green	G1134	G1134.5	Saranac_DS	ERF	TF
paleturquoise	G74978	G74978.5	PI467895_SS	AGC-Pl	PK	red	G54933	G54933.2	Wilson_DS	lncRNA	lncRNA	green	G3628	G3628.2	Saranac_DS	GNAT	TR
paleturquoise	G77422	G77422.2	PI467895_SS	CK1	PK	red	G56428	G56428.4	Wilson_DS	lncRNA	lncRNA	green	G4450	G4450.1	Saranac_DS	CAMK	PK
paleturquoise	G77973	G77973.4	PI467895_SS	HMG	TR	red	G57637	G57637.3	Wilson_DS	lncRNA	lncRNA	green	G4814	G4814.2	Saranac_DS	lncRNA	lncRNA
paleturquoise	G83107	G83107.6	PI467895_SS	MYB_related	TF	red	G59115	G59115.9	Wilson_DS	CAMK	PK	green	G4814	G4814.5	Saranac_DS	lncRNA	lncRNA
paleturquoise	G89545	G89545.38	PI467895_SS	MIKC_MADS	TF	red	G63831	G63831.43	Wilson_DS	lncRNA	lncRNA	green	G7888	G7888.12	Saranac_DS	CMGC	PK
paleturquoise	G89701	G89701.6	PI467895_SS	RLK-Pelle	PK	red	G77312	G77312.8	Wilson_DS	SET	TR	green	G8197	G8197.4	Saranac_DS	NAC	TF

black	G3034	G3034.11	PI467895_SS	lncRNA	lncRNA
black	G6502	G6502.27	PI467895_SS	SBP	TF
black	G10123	G10123.3	PI467895_SS	CAMK	PK
black	G10545	G10545.2	PI467895_SS	CK1	PK
black	G10779	G10779.1	PI467895_SS	MYB_related	TF
black	G12895	G12895.6	PI467895_SS	B3	TF
black	G12895	G12895.7	PI467895_SS	B3	TF
black	G13623	G13623.3	PI467895_SS	lncRNA	lncRNA
black	G14019	G14019.1	PI467895_SS	AUX/IAA	TR
black	G16342	G16342.4	PI467895_SS	ERF	TF
black	G16848	G16848.8	PI467895_SS	MYB	TF
black	G18454	G18454.13	PI467895_SS	CAMK	PK
black	G18719	G18719.4	PI467895_SS	C2H2	TF
black	G18869	G18869.19	PI467895_SS	TCP	TF
black	G23009	G23009.1	PI467895_SS	GeBP	TF
black	G25638	G25638.5	PI467895_SS	GeBP	TF
black	G27409	G27409.1	PI467895_SS	CMGC	PK
black	G29627	G29627.21	PI467895_SS	CMGC	PK
black	G31800	G31800.3	PI467895_SS	HMG	TR
black	G32390	G32390.2	PI467895_SS	C3H	TF
black	G33816	G33816.4	PI467895_SS	Trihelix	TF
black	G34213	G34213.7	PI467895_SS	RLK-Pelle	PK
black	G34218	G34218.1	PI467895_SS	lncRNA	lncRNA
black	G36167	G36167.8	PI467895_SS	NF-YB	TF
black	G36299	G36299.1	PI467895_SS	lncRNA	lncRNA
black	G38537	G38537.4	PI467895_SS	lncRNA	lncRNA
black	G40402	G40402.4	PI467895_SS	NF-YC	TF
black	G44472	G44472.25	PI467895_SS	RLK-Pelle	PK
black	G45475	G45475.1	PI467895_SS	lncRNA	lncRNA
black	G50682	G50682.1	PI467895_SS	lncRNA	lncRNA
black	G51566	G51566.3	PI467895_SS	lncRNA	lncRNA
black	G53110	G53110.1	PI467895_SS	lncRNA	lncRNA
black	G53462	G53462.1	PI467895_SS	CAMK	PK
black	G54585	G54585.19	PI467895_SS	GNAT	TR
black	G55110	G55110.18	PI467895_SS	IWS1	TR
black	G59042	G59042.1	PI467895_SS	lncRNA	lncRNA
black	G60340	G60340.72	PI467895_SS	CMGC	PK
black	G60442	G60442.11	PI467895_SS	bHLH	TF
black	G60442	G60442.12	PI467895_SS	bHLH	TF
black	G61713	G61713.26	PI467895_SS	CAMK	PK
black	G62997	G62997.4	PI467895_SS	NF-X1	TF
black	G64131	G64131.10	PI467895_SS	Trihelix	TF
black	G65222	G65222.84	PI467895_SS	IWS1	TR
black	G66279	G66279.76	PI467895_SS	AGC	PK
black	G66915	G66915.8	PI467895_SS	Trihelix	TF
black	G68876	G68876.18	PI467895_SS	CK1	PK
black	G73209	G73209.2	PI467895_SS	lncRNA	lncRNA
black	G73663	G73663.12	PI467895_SS	CMGC	PK
black	G76628	G76628.2	PI467895_SS	AGC	PK
black	G77480	G77480.1	PI467895_SS	GNAT	TR
black	G77973	G77973.2	PI467895_SS	HMG	TR
black	G79064	G79064.10	PI467895_SS	NAC	TF
black	G79210	G79210.15	PI467895_SS	bHLH	TF
black	G80477	G80477.2	PI467895_SS	MYB_related	TF
black	G87938	G87938.9	PI467895_SS	lncRNA	lncRNA
black	G89321	G89321.1	PI467895_SS	bZIP	TF
black	G89509	G89509.66	PI467895_SS	CMGC	PK
black	G89991	G89991.6	PI467895_SS	bZIP	TF
black	G90071	G90071.4	PI467895_SS	AUX/IAA	TR
lightsteelblue	G163	G163.3	Saranac_SS	MYB_related	TF
red	G78965	G78965.8	Wilson_DS	RLK-Pelle	PK
red	G79598	G79598.44	Wilson_DS	CMGC	PK
red	G79775	G79775.2	Wilson_DS	RLK-Pelle	PK
red	G83410	G83410.3	Wilson_DS	CMGC	PK
red	G83858	G83858.1	Wilson_DS	bHLH	TF
red	G83989	G83989.10	Wilson_DS	HD-ZIP	TF
red	G85380	G85380.1	Wilson_DS	bZIP	TF
red	G87537	G87537.12	Wilson_DS	M-type_MA	TF
red	G88273	G88273.17	Wilson_DS	lncRNA	lncRNA
red	G88401	G88401.3	Wilson_DS	lncRNA	lncRNA
red	G88719	G88719.14	Wilson_DS	lncRNA	lncRNA
maroon	G11805	G11805.1	Wilson_DS	RLK-Pelle	PK
maroon	G25025	G25025.1	Wilson_DS	CMGC	PK
maroon	G26989	G26989.1	Wilson_DS	lncRNA	lncRNA
maroon	G39199	G39199.12	Wilson_DS	GRAS	TF
maroon	G41844	G41844.1	Wilson_DS	RLK-Pelle	PK
maroon	G45732	G45732.18	Wilson_DS	bZIP	TF
maroon	G55418	G55418.13	Wilson_DS	HD-ZIP	TF
maroon	G67420	G67420.5	Wilson_DS	BBR-BPC	TF
maroon	G69075	G69075.1	Wilson_DS	Trihelix	TF
maroon	G75082	G75082.7	Wilson_DS	CAMK	PK
lightcyan	G573	G573.10	Wilson_DS	ERF	TF
lightcyan	G748	G748.3	Wilson_DS	Others	TR
lightcyan	G2480	G2480.28	Wilson_DS	MYB	TF
lightcyan	G2664	G2664.8	Wilson_DS	B3	TF
lightcyan	G4828	G4828.4	Wilson_DS	AGC	PK
lightcyan	G5905	G5905.5	Wilson_DS	ERF	TF
lightcyan	G10663	G10663.4	Wilson_DS	TRAF	TR
lightcyan	G14128	G14128.1	Wilson_DS	lncRNA	lncRNA
lightcyan	G22927	G22927.1	Wilson_DS	TKL-Pl-4	PK
lightcyan	G23084	G23084.5	Wilson_DS	lncRNA	lncRNA
lightcyan	G23596	G23596.12	Wilson_DS	MYB	TF
lightcyan	G23928	G23928.12	Wilson_DS	C2H2	TF
lightcyan	G24639	G24639.33	Wilson_DS	CAMK	PK
lightcyan	G31644	G31644.3	Wilson_DS	RLK-Pelle	PK
lightcyan	G32807	G32807.18	Wilson_DS	GRAS	TF
lightcyan	G34684	G34684.2	Wilson_DS	RLK-Pelle	PK
lightcyan	G36744	G36744.6	Wilson_DS	ERF	TF
lightcyan	G43317	G43317.2	Wilson_DS	lncRNA	lncRNA
lightcyan	G45971	G45971.1	Wilson_DS	lncRNA	lncRNA
lightcyan	G48479	G48479.8	Wilson_DS	lncRNA	lncRNA
lightcyan	G51891	G51891.11	Wilson_DS	HSF	TF
lightcyan	G52829	G52829.5	Wilson_DS	MYB_relatec	TF
lightcyan	G55437	G55437.35	Wilson_DS	RLK-Pelle	PK
lightcyan	G56389	G56389.3	Wilson_DS	lncRNA	lncRNA
lightcyan	G58889	G58889.5	Wilson_DS	lncRNA	lncRNA
lightcyan	G58931	G58931.2	Wilson_DS	lncRNA	lncRNA
lightcyan	G59625	G59625.22	Wilson_DS	lncRNA	lncRNA
lightcyan	G62411	G62411.31	Wilson_DS	TKL-Pl-6	PK
lightcyan	G62887	G62887.9	Wilson_DS	RLK-Pelle	PK
lightcyan	G63336	G63336.1	Wilson_DS	lncRNA	lncRNA
lightcyan	G63366	G63366.2	Wilson_DS	AGC	PK
lightcyan	G79598	G79598.47	Wilson_DS	CMGC	PK
lightcyan	G89660	G89660.4	Wilson_DS	RLK-Pelle	PK
lightcyan	G90438	G90438.51	Wilson_DS	PHD	TR
darkslateblue	G1661	G1661.1	Saranac_DS	C2H2	TF
darkslateblue	G2506	G2506.3	Saranac_DS	lncRNA	lncRNA
darkslateblue	G2875	G2875.3	Saranac_DS	CAMK	PK
darkslateblue	G4994	G4994.48	Saranac_DS	NAC	TF
darkslateblue	G9055	G9055.11	Saranac_DS	lncRNA	lncRNA
green	G8422	G8422.6	Saranac_DS	M-type_MA	TF
green	G9325	G9325.1	Saranac_DS	CAMK	PK
green	G9325	G9325.8	Saranac_DS	CAMK	PK
green	G10860	G10860.6	Saranac_DS	CAMK	PK
green	G11097	G11097.60	Saranac_DS	SBP	TF
green	G12294	G12294.8	Saranac_DS	ERF	TF
green	G12481	G12481.2	Saranac_DS	CAMK	PK
green	G12481	G12481.3	Saranac_DS	CAMK	PK
green	G14801	G14801.4	Saranac_DS	lncRNA	lncRNA
green	G15196	G15196.1	Saranac_DS	MYB	TF
green	G18843	G18843.5	Saranac_DS	ERF	TF
green	G20711	G20711.1	Saranac_DS	ERF	TF
green	G21416	G21416.1	Saranac_DS	ERF	TF
green	G21416	G21416.6	Saranac_DS	ERF	TF
green	G21594	G21594.3	Saranac_DS	RLK-Pelle	PK
green	G21688	G21688.4	Saranac_DS	NF-YC	TF
green	G22908	G22908.8	Saranac_DS	lncRNA	lncRNA
green	G26995	G26995.7	Saranac_DS	ERF	TF
green	G27973	G27973.3	Saranac_DS	HD-ZIP	TF
green	G30158	G30158.7	Saranac_DS	MYB	TF
green	G30531	G30531.11	Saranac_DS	EIL	TF
green	G31744	G31744.4	Saranac_DS	MYB	TF
green	G32784	G32784.7	Saranac_DS	SWI/SNF-B	TR
green	G32784	G32784.7	Saranac_DS	lncRNA	lncRNA
green	G34100	G34100.1	Saranac_DS	HD-ZIP	TF
green	G34684	G34684.3	Saranac_DS	RLK-Pelle	PK
green	G34943	G34943.10	Saranac_DS	RLK-Pelle	PK
green	G38448	G38448.3	Saranac_DS	C3H	TF
green	G39693	G39693.1	Saranac_DS	lncRNA	lncRNA
green	G40088	G40088.2	Saranac_DS	lncRNA	lncRNA
green	G41523	G41523.1	Saranac_DS	STE	PK
green	G45179	G45179.7	Saranac_DS	AUX/IAA	TR
green	G48528	G48528.6	Saranac_DS	C3H	TF
green	G49950	G49950.2	Saranac_DS	lncRNA	lncRNA
green	G50040	G50040.1	Saranac_DS	ERF	TF
green	G50358	G50358.3	Saranac_DS	bZIP	TF
green	G50358	G50358.8	Saranac_DS	bZIP	TF
green	G54752	G54752.1	Saranac_DS	HD-ZIP	TF
green	G58052	G58052.18	Saranac_DS	RLK-Pelle	PK
green	G59319	G59319.4	Saranac_DS	GRAS	TF
green	G62086	G62086.55	Saranac_DS	TKL-Pl-4	PK
green	G65415	G65415.2	Saranac_DS	RLK-Pelle	PK
green	G68179	G68179.17	Saranac_DS	TALE	TF
green	G68864	G68864.16	Saranac_DS	RLK-Pelle	PK
green	G68864	G68864.17	Saranac_DS	RLK-Pelle	PK
green	G70939	G70939.56	Saranac_DS	TALE	TF
green	G70939	G70939.59	Saranac_DS	TALE	TF
green	G70939	G70939.62	Saranac_DS	TALE	TF
green	G70939	G70939.63	Saranac_DS	TALE	TF
green	G71414	G71414.2	Saranac_DS	CAMK	PK
green	G77397	G77397.5	Saranac_DS	lncRNA	lncRNA
green	G77397	G77397.7	Saranac_DS	lncRNA	lncRNA
green	G79825	G79825.5	Saranac_DS	NF-YB	TF
green	G79833	G79833.5	Saranac_DS	NF-YB	TF
green	G83532	G83532.1	Saranac_DS	C2H2	TF
green	G84709	G84709.20	Saranac_DS	HD-ZIP	TF
green	G85463	G85463.6	Saranac_DS	WRKY	TF
green	G86245	G86245.60	Saranac_DS	CMGC	PK
green	G88582	G88582.2	Saranac_DS	lncRNA	lncRNA
green	G89583	G89583.8	Saranac_DS	MYB_relatec	TF

lightsteelblue	G4762	G4762.7	Saranac_SS	RLK-Pelle	PK	darkslateblue	G9325	G9325.6	Saranac_DS	CAMK	PK	green	G90363	G90363.26	Saranac_DS	CMGC	PK
lightsteelblue	G19540	G19540.1	Saranac_SS	lncRNA	lncRNA	darkslateblue	G10342	G10342.1	Saranac_DS	CMGC	PK	green	G90907	G90907.10	Saranac_DS	HD-ZIP	TF
lightsteelblue	G29627	G29627.10	Saranac_SS	CMGC	PK	darkslateblue	G10779	G10779.3	Saranac_DS	MYB_relatec	TF	salmon4	G647	G647.1	Saranac_DS	C2H2	TF
lightsteelblue	G39199	G39199.14	Saranac_SS	GRAS	TF	darkslateblue	G11097	G11097.1	Saranac_DS	SBP	TF	salmon4	G4431	G4431.45	Saranac_DS	RLK-Pelle	PK
lightsteelblue	G39199	G39199.15	Saranac_SS	GRAS	TF	darkslateblue	G14719	G14719.24	Saranac_DS	bZIP	TF	salmon4	G5774	G5774.1	Saranac_DS	bZIP	TF
lightsteelblue	G43302	G43302.1	Saranac_SS	C3H	TF	darkslateblue	G16771	G16771.83	Saranac_DS	CAMK	PK	salmon4	G7888	G7888.5	Saranac_DS	CMGC	PK
lightsteelblue	G45458	G45458.5	Saranac_SS	CMGC	PK	darkslateblue	G17773	G17773.21	Saranac_DS	lncRNA	lncRNA	salmon4	G7888	G7888.43	Saranac_DS	CMGC	PK
lightsteelblue	G90880	G90880.1	Saranac_SS	ERF	TF	darkslateblue	G20099	G20099.6	Saranac_DS	SNF2	TR	salmon4	G11708	G11708.1	Saranac_DS	C2H2	TF
white	G2636	G2636.3	Saranac_SS	lncRNA	lncRNA	darkslateblue	G20099	G20099.10	Saranac_DS	SNF2	TR	salmon4	G11871	G11871.5	Saranac_DS	MYB	TF
white	G2907	G2907.25	Saranac_SS	TALE	TF	darkslateblue	G22077	G22077.33	Saranac_DS	lncRNA	lncRNA	salmon4	G16342	G16342.6	Saranac_DS	ERF	TF
white	G5056	G5056.12	Saranac_SS	Trihelix	TF	darkslateblue	G23452	G23452.2	Saranac_DS	CK1	PK	salmon4	G20815	G20815.18	Saranac_DS	NF-YA	TF
white	G12189	G12189.19	Saranac_SS	HMG	TR	darkslateblue	G28628	G28628.2	Saranac_DS	lncRNA	lncRNA	salmon4	G21416	G21416.7	Saranac_DS	ERF	TF
white	G13672	G13672.11	Saranac_SS	Pseudo ARR-I	TR	darkslateblue	G37254	G37254.42	Saranac_DS	RLK-Pelle	PK	salmon4	G29759	G29759.1	Saranac_DS	SWI/SNF-B/	TR
white	G14372	G14372.3	Saranac_SS	bZIP	TF	darkslateblue	G42790	G42790.1	Saranac_DS	MYB_relatec	TF	salmon4	G30068	G30068.2	Saranac_DS	ERF	TF
white	G29734	G29734.4	Saranac_SS	SWI/SNF-BA	TR	darkslateblue	G44987	G44987.9	Saranac_DS	WRKY	TF	salmon4	G30998	G30998.2	Saranac_DS	HD-ZIP	TF
white	G31744	G31744.3	Saranac_SS	MYB	TF	darkslateblue	G56529	G56529.18	Saranac_DS	EIL	TF	salmon4	G33212	G33212.2	Saranac_DS	lncRNA	lncRNA
white	G35425	G35425.26	Saranac_SS	C2H2	TF	darkslateblue	G58149	G58149.1	Saranac_DS	lncRNA	lncRNA	salmon4	G33416	G33416.12	Saranac_DS	ERF	TF
white	G50089	G50089.6	Saranac_SS	E2F/DP	TF	darkslateblue	G59294	G59294.7	Saranac_DS	PHD	TR	salmon4	G34549	G34549.3	Saranac_DS	ERF	TF
white	G52841	G52841.4	Saranac_SS	lncRNA	lncRNA	darkslateblue	G61695	G61695.7	Saranac_DS	EIL	TF	salmon4	G34549	G34549.4	Saranac_DS	ERF	TF
white	G57658	G57658.3	Saranac_SS	SWI/SNF-BA	TR	darkslateblue	G63579	G63579.39	Saranac_DS	IWS1	TR	salmon4	G39626	G39626.7	Saranac_DS	bZIP	TF
white	G58215	G58215.2	Saranac_SS	lncRNA	lncRNA	darkslateblue	G65222	G65222.68	Saranac_DS	IWS1	TR	salmon4	G43862	G43862.3	Saranac_DS	LBD	TF
white	G58215	G58215.4	Saranac_SS	lncRNA	lncRNA	darkslateblue	G65222	G65222.83	Saranac_DS	IWS1	TR	salmon4	G48794	G48794.3	Saranac_DS	NAC	TF
white	G60270	G60270.1	Saranac_SS	SWI/SNF-BA	TR	darkslateblue	G68635	G68635.18	Saranac_DS	CAMK	PK	salmon4	G49209	G49209.9	Saranac_DS	MIKC_MAI	TF
white	G63326	G63326.6	Saranac_SS	RLK-Pelle	PK	darkslateblue	G68635	G68635.38	Saranac_DS	CAMK	PK	salmon4	G52054	G52054.1	Saranac_DS	bHLH	TF
white	G80765	G80765.7	Saranac_SS	AUX/IAA	TR	darkslateblue	G73821	G73821.3	Saranac_DS	RLK-Pelle	PK	salmon4	G52063	G52063.1	Saranac_DS	HD-ZIP	TF
white	G84598	G84598.38	Saranac_SS	CMGC	PK	darkslateblue	G74478	G74478.11	Saranac_DS	SET	TR	salmon4	G53751	G53751.29	Saranac_DS	C3H	TF
orangered3	G14172	G14172.4	Saranac_SS	AUX/IAA	TR	darkslateblue	G76548	G76548.6	Saranac_DS	MYB_relatec	TF	salmon4	G60114	G60114.56	Saranac_DS	C3H	TF
orangered3	G18438	G18438.4	Saranac_SS	GATA	TF	darkslateblue	G80213	G80213.1	Saranac_DS	MYB_relatec	TF	salmon4	G63248	G63248.1	Saranac_DS	RLK-Pelle	PK
orangered3	G19891	G19891.57	Saranac_SS	AGC	PK	darkslateblue	G80213	G80213.2	Saranac_DS	MYB_relatec	TF	salmon4	G65222	G65222.52	Saranac_DS	IWS1	TR
orangered3	G27544	G27544.51	Saranac_SS	lncRNA	lncRNA	darkslateblue	G90981	G90981.1	Saranac_DS	Trihelix	TF	salmon4	G71136	G71136.3	Saranac_DS	NF-YB	TF
orangered3	G37402	G37402.17	Saranac_SS	NAC	TF	mediumpurpl	G4615	G4615.41	Saranac_DS	CMGC	PK	salmon4	G79943	G79943.5	Saranac_DS	bHLH	TF
orangered3	G52256	G52256.124	Saranac_SS	lncRNA	lncRNA	mediumpurpl	G4615	G4615.41	Saranac_DS	C3H	TF	salmon4	G80321	G80321.7	Saranac_DS	HD-ZIP	TF
orangered3	G57607	G57607.5	Saranac_SS	CMGC	PK	mediumpurpl	G17693	G17693.17	Saranac_DS	CMGC	PK	salmon4	G81658	G81658.6	Saranac_DS	WRKY	TF
orangered3	G68404	G68404.38	Saranac_SS	G2-like	TF	mediumpurpl	G22077	G22077.10	Saranac_DS	lncRNA	lncRNA	salmon4	G81715	G81715.11	Saranac_DS	HD-ZIP	TF
orangered3	G70997	G70997.2	Saranac_SS	AGC	PK	mediumpurpl	G22148	G22148.14	Saranac_DS	SET	TR	salmon4	G83400	G83400.1	Saranac_DS	HD-ZIP	TF
orangered3	G82273	G82273.22	Saranac_SS	CAMK	PK	mediumpurpl	G22927	G22927.2	Saranac_DS	TKL-Pl-4	PK	salmon4	G90907	G90907.7	Saranac_DS	HD-ZIP	TF

Supplementary Table 15. Pathways annotation in isoforms of modules significatively detected in Saranac, PI467895 and Wilson under salt (SS) or drought stress (DS).

Module	gene_id	isoform	treatment	Uniprot_ID	Protein_names	Pathway
darkslateblue	G3052	G3052.3	Saranac_DS	A0A2K3KZB5	RING-type E3 ubiquitin transferase (EC 2.3.2.27)	Protein modification; protein ubiquitination. {ECO:0000256|ARBA:ARBA00004906}.
darkslateblue	G21939	G21939.2	Saranac_DS	A0A0S3R8S2	S-adenosylmethionine synthase (EC 2.5.1.6)	Amino-acid biosynthesis; S-adenosyl-L-methionine biosynthesis; S-adenosyl-L-methionine from L-methionine: step 1/1. {ECO:0000256|ARBA:ARBA00005224, ECO:0000256|RuleBase:RU000541}.
darkslateblue	G33538	G33538.5	Saranac_DS	V7CER6	RING-type E3 ubiquitin transferase (EC 2.3.2.27)	Protein modification; protein ubiquitination. {ECO:0000256|ARBA:ARBA00004906}.
darkslateblue	G82844	G82844.35	Saranac_DS	A0A2I4F566	Sucrose-phosphate synthase (EC 2.4.1.14)	Glycan biosynthesis; sucrose biosynthesis; sucrose from D-fructose 6-phosphate and UDP-alpha-D-glucose: step 1/2. {ECO:0000256|ARBA:ARBA00005027}.
darkslateblue	G90339	G90339.1	Saranac_DS	A0A5N6QR28	Fructose-bisphosphate aldolase (EC 4.1.2.13)	Carbohydrate degradation; glycolysis; D-glyceraldehyde 3-phosphate and glycerone phosphate from D-glucose: step 4/4. {ECO:0000256|ARBA:ARBA00004714}.
mediumpurple3	G18202	G18202.8	Saranac_DS	A0A0R0KMH5	Uncharacterized protein	Protein modification; protein ubiquitination. {ECO:0000256|ARBA:ARBA00004906}.
mediumpurple3	G20849	G20849.6	Saranac_DS	A0A445BVJ4	Uncharacterized protein	Protein modification; protein ubiquitination. {ECO:0000256|ARBA:ARBA00004906}.
mediumpurple3	G48918	G48918.1	Saranac_DS	A0A3P9QNB7	Inositol-3-phosphate synthase (EC 5.5.1.4)	Polyol metabolism; myo-inositol biosynthesis; myo-inositol from D-glucose 6-phosphate: step 1/2. {ECO:0000256|ARBA:ARBA00005117}.
mediumpurple3	G48918	G48918.41	Saranac_DS	A0A4D6MD20	Inositol-3-phosphate synthase (EC 5.5.1.4)	Polyol metabolism; myo-inositol biosynthesis; myo-inositol from D-glucose 6-phosphate: step 1/2. {ECO:0000256|ARBA:ARBA00005117}.
mediumpurple3	G56450	G56450.49	Saranac_DS	A0A445C4V4	E3 ubiquitin protein ligase (EC 2.3.2.27)	Protein modification; protein ubiquitination. {ECO:0000256|ARBA:ARBA00004906, ECO:0000256|RuleBase:RU365038}.
mediumpurple3	G59465	G59465.3	Saranac_DS	A0A5N6QLK2	Diacylglycerol O-acyltransferase (EC 2.3.1.20)	Glycerolipid metabolism; triacylglycerol biosynthesis. {ECO:0000256|ARBA:ARBA00004771}.; Lipid metabolism. {ECO:0000256|ARBA:ARBA00005189}.
indianred4	G42177	G42177.39	Saranac_DS	A0A4P1RP56	Inositol-3-phosphate synthase (EC 5.5.1.4)	Polyol metabolism; myo-inositol biosynthesis; myo-inositol from D-glucose 6-phosphate: step 1/2. {ECO:0000256|ARBA:ARBA00005117}.
indianred4	G42177	G42177.60	Saranac_DS	A0A3P9QNB7	Inositol-3-phosphate synthase (EC 5.5.1.4)	Polyol metabolism; myo-inositol biosynthesis; myo-inositol from D-glucose 6-phosphate: step 1/2. {ECO:0000256|ARBA:ARBA00005117}.
indianred4	G42177	G42177.85	Saranac_DS	A0A1J3EMT6	Inositol-3-phosphate synthase (EC 5.5.1.4) (Fragment)	Polyol metabolism; myo-inositol biosynthesis; myo-inositol from D-glucose 6-phosphate: step 1/2. {ECO:0000256|ARBA:ARBA00005117}.
indianred4	G42177	G42177.86	Saranac_DS	A0A3P9QNB7	Inositol-3-phosphate synthase (EC 5.5.1.4)	Polyol metabolism; myo-inositol biosynthesis; myo-inositol from D-glucose 6-phosphate: step 1/2. {ECO:0000256|ARBA:ARBA00005117}.
indianred4	G80184	G80184.29	Saranac_DS	P29001	Acid beta-fructofuranosidase (EC 3.2.1.26) (Acid invertase) (AI) (Acid sucrose hydrolase) (Vacuolar invertase) [Cleaved into: Acid beta-fructofuranosidase 30 kDa subunit; Acid beta-fructofuranosidase 38 kDa subunit; Acid beta-fructofuranosidase 70 kDa monomer]	Glycan biosynthesis; sucrose metabolism. {ECO:0000305}.
blue	G6812	G6812.3	Saranac_DS	A0A061DF70	SKP1-like protein	Protein modification; protein ubiquitination. {ECO:0000256|ARBA:ARBA00004906, ECO:0000256|PIRNR:PIRNR028729}.
blue	G8440	G8440.11	Saranac_DS	A0A2P2JDP0	Sulfate adenylyltransferase (EC 2.7.7.4)	Sulfur metabolism. {ECO:0000256|ARBA:ARBA00004678}.
blue	G8440	G8440.20	Saranac_DS	A0A371I2Y8	Sulfate adenylyltransferase (EC 2.7.7.4) (Fragment)	Sulfur metabolism. {ECO:0000256|ARBA:ARBA00004678}.
blue	G10086	G10086.1	Saranac_DS	I3SWX0	SKP1-like protein	Protein modification; protein ubiquitination. {ECO:0000256|ARBA:ARBA00004906, ECO:0000256|PIRNR:PIRNR028729}.
blue	G11581	G11581.2	Saranac_DS	A0A087HBS0	Sulfate adenylyltransferase (EC 2.7.7.4)	Sulfur metabolism. {ECO:0000256|ARBA:ARBA00004678}.
blue	G14551	G14551.3	Saranac_DS	E5GCD7	Succinate dehydrogenase [ubiquinone] iron-sulfur subunit, mitochondrial (EC 1.3.5.1)	Carbohydrate metabolism; tricarboxylic acid cycle; fumarate from succinate (eukaryal route): step 1/1. {ECO:0000256|ARBA:ARBA00004788, ECO:0000256|RuleBase:RU361237}.
blue	G17475	G17475.3	Saranac_DS	A0A5N5FI72	Lactoylglutathione lyase (EC 4.4.1.5) (Glyoxalase I)	Secondary metabolite metabolism; methylglyoxal degradation; (R)-lactate from methylglyoxal: step 1/2. {ECO:0000256|ARBA:ARBA00005008, ECO:0000256|RuleBase:RU361179}.
blue	G17475	G17475.5	Saranac_DS	A0A5N5FI72	Lactoylglutathione lyase (EC 4.4.1.5) (Glyoxalase I)	Secondary metabolite metabolism; methylglyoxal degradation; (R)-lactate from methylglyoxal: step 1/2. {ECO:0000256|ARBA:ARBA00005008, ECO:0000256|RuleBase:RU361179}.
blue	G19354	G19354.12	Saranac_DS	A0A0S3R8S2	S-adenosylmethionine synthase (EC 2.5.1.6)	Amino-acid biosynthesis; S-adenosyl-L-methionine biosynthesis; S-adenosyl-L-methionine from L-methionine: step 1/1. {ECO:0000256|ARBA:ARBA00005224, ECO:0000256|RuleBase:RU000541}.
blue	G19941	G19941.12	Saranac_DS	A0A444ZCX7	Fumarylacetoacetase (EC 3.7.1.2) (Fumarylacetoacetate hydrolase)	Amino-acid degradation; L-phenylalanine degradation; acetoacetate and fumarate from L-phenylalanine: step 6/6. {ECO:0000256|ARBA:ARBA00004782}.

blue	G20541	G20541.35	Saranac_DS	A0A2I4GDS0	Serine hydroxymethyltransferase (EC 2.1.2.1)	One-carbon metabolism; tetrahydrofolate interconversion. {ECO:0000256|ARBA:ARBA00004777, ECO:0000256|RuleBase:RU000585}.
blue	G21939	G21939.1	Saranac_DS	A0A0S3R8S2	S-adenosylmethionine synthase (EC 2.5.1.6)	Amino-acid biosynthesis; S-adenosyl-L-methionine biosynthesis; S-adenosyl-L-methionine from L-methionine: step 1/1. {ECO:0000256|ARBA:ARBA00005224, ECO:0000256|RuleBase:RU000541}.
blue	G29371	G29371.40	Saranac_DS	A0A4D6M6H2	Cellulose synthase (EC 2.4.1.12)	Glycan metabolism; plant cellulose biosynthesis. {ECO:0000256|ARBA:ARBA00004768, ECO:0000256|RuleBase:RU361116}.
blue	G35677	G35677.59	Saranac_DS	A0A445GZ81	Cellulose synthase (EC 2.4.1.12)	Glycan metabolism; plant cellulose biosynthesis. {ECO:0000256|ARBA:ARBA00004768, ECO:0000256|RuleBase:RU361116}.
blue	G42592	G42592.5	Saranac_DS	A0A1S3T8F5	RING-type E3 ubiquitin transferase (EC 2.3.2.27)	Protein modification; protein ubiquitination. {ECO:0000256|ARBA:ARBA00004906}.
blue	G46912	G46912.13	Saranac_DS	A0A5D3BUW0	Amidophosphoribosyltransferase (EC 2.4.2.14)	Purine metabolism; IMP biosynthesis via de novo pathway; N(1)-(5-phospho-D-ribosyl)glycinamide from 5-phospho-alpha-D-ribose 1-diphosphate: step 1/2. {ECO:0000256|ARBA:ARBA00005209}.
blue	G60813	G60813.10	Saranac_DS	A0A061EAW6	UDP-glucose 6-dehydrogenase (EC 1.1.1.22)	Nucleotide-sugar biosynthesis; UDP-alpha-D-glucuronate biosynthesis; UDP-alpha-D-glucuronate from UDP-alpha-D-glucose: step 1/1. {ECO:0000256|ARBA:ARBA00004701}.
blue	G71754	G71754.5	Saranac_DS	A0A5D2IIX8	F-box domain-containing protein	Protein modification; protein ubiquitination. {ECO:0000256|ARBA:ARBA00004906}.
blue	G77399	G77399.9	Saranac_DS	A0A1J7IBJ7	5-methyltetrahydropteroyltriglutamate--homocysteine S-methyltransferase (EC 2.1.1.14)	Amino-acid biosynthesis; L-methionine biosynthesis via de novo pathway; L-methionine from L-homocysteine (MetE route): step 1/1. {ECO:0000256|ARBA:ARBA00004681}.
blue	G77399	G77399.45	Saranac_DS	A0A4D6M7Z0	5-methyltetrahydropteroyltriglutamate--homocysteine S-methyltransferase (EC 2.1.1.14)	Amino-acid biosynthesis; L-methionine biosynthesis via de novo pathway; L-methionine from L-homocysteine (MetE route): step 1/1. {ECO:0000256|ARBA:ARBA00004681}.
blue	G82243	G82243.32	Saranac_DS	A0A4P1QW91	Pyrophosphate--fructose 6-phosphate 1-phosphotransferase subunit beta (PFP) (EC 2.7.1.90) (6-phosphofructokinase, pyrophosphate dependent) (PPi-PFK) (Pyrophosphate-dependent 6-phosphofructose-1-kinase)	Carbohydrate degradation; glycolysis; D-glyceraldehyde 3-phosphate and glycerone phosphate from D-glucose: step 3/4. {ECO:0000256|ARBA:ARBA00004679, ECO:0000256|HAMAP-Rule:MF_03185}.
blue	G82990	G82990.8	Saranac_DS	A0A2P5D6M2	UDP-glucose 6-dehydrogenase (EC 1.1.1.22)	Nucleotide-sugar biosynthesis; UDP-alpha-D-glucuronate biosynthesis; UDP-alpha-D-glucuronate from UDP-alpha-D-glucose: step 1/1. {ECO:0000256|ARBA:ARBA00004701}.
floralwhite	G42435	G42435.9	Saranac_DS	I1K3G2	Ornithine aminotransferase (EC 2.6.1.13)	Amino-acid biosynthesis; L-proline biosynthesis; L-glutamate 5-semialdehyde from L-ornithine: step 1/1. {ECO:0000256|ARBA:ARBA00004998, ECO:0000256|RuleBase:RU365036}.
floralwhite	G47094	G47094.18	Saranac_DS	A0A151SUG9	RBR-type E3 ubiquitin transferase (EC 2.3.2.31)	Protein modification; protein ubiquitination. {ECO:0000256|ARBA:ARBA00004906}.
green	G518	G518.5	Saranac_DS	A0A1S3VFI3	Serine O-acetyltransferase (EC 2.3.1.30)	Amino-acid biosynthesis; L-cysteine biosynthesis; L-cysteine from L-serine: step 1/2. {ECO:0000256|ARBA:ARBA00004876}.
green	G3988	G3988.7	Saranac_DS	A0A077CU52	Phenylalanine ammonia-lyase (EC 4.3.1.24)	Phenylpropanoid metabolism; trans-cinnamate biosynthesis; trans-cinnamate from L-phenylalanine: step 1/1. {ECO:0000256|ARBA:ARBA00005138, ECO:0000256|RuleBase:RU003955}.
green	G47008	G47008.1	Saranac_DS	A0A1R3HVC4	Uncharacterized protein	Protein modification; protein ubiquitination. {ECO:0000256|ARBA:ARBA00004906}.
green	G68854	G68854.23	Saranac_DS	A0A151U067	5-methyltetrahydropteroyltriglutamate--homocysteine S-methyltransferase (EC 2.1.1.14)	Amino-acid biosynthesis; L-methionine biosynthesis via de novo pathway; L-methionine from L-homocysteine (MetE route): step 1/1. {ECO:0000256|ARBA:ARBA00004681}.
green	G76732	G76732.1	Saranac_DS	G7IBI3	Phenylalanine ammonia-lyase (EC 4.3.1.24)	Phenylpropanoid metabolism; trans-cinnamate biosynthesis; trans-cinnamate from L-phenylalanine: step 1/1. {ECO:0000256|ARBA:ARBA00005138, ECO:0000256|RuleBase:RU003955}.
salmon4	G8313	G8313.87	Saranac_DS	A0A2D1WBB1	RING-type E3 ubiquitin transferase (EC 2.3.2.27)	Protein modification; protein ubiquitination. {ECO:0000256|ARBA:ARBA00004906}.
salmon4	G50021	G50021.28	Saranac_DS	A0A5A7RH59	Ketol-acid reductoisomerase (EC 1.1.1.86) (Acetohydroxy-acid reductoisomerase) (Alpha-keto-beta-hydroxylacyl reductoisomerase)	Amino-acid biosynthesis; L-isoleucine biosynthesis; L-isoleucine from 2-oxobutanoate: step 2/4. {ECO:0000256|ARBA:ARBA00004885, ECO:0000256|PIRNR:PIRNR000118}.; Amino-acid biosynthesis; L-valine biosynthesis; L-valine from pyruvate: step 2/4. {ECO:0000256|ARBA:ARBA00004864, ECO:0000256|PIRNR:PIRNR000118}.

salmon4	G88991	G88991.4	Saranac_DS	V7C7S9	3-hydroxyacyl-CoA dehydrogenase (EC 1.1.1.35) (EC 4.2.1.17) (EC 5.1.2.3) (EC 5.3.3.8) (Enoyl-CoA hydratase/3-2-trans-enoyl-CoA isomerase/3-hydroxybutyryl-CoA epimerase)	Lipid metabolism; fatty acid beta-oxidation. {ECO:0000256|ARBA:ARBA00005005}.
lightsteelblue	G26540	G26540.1	Saranac_SS	A0A0A0M065	Tryptophan synthase (EC 4.2.1.20)	Amino-acid biosynthesis; L-tryptophan biosynthesis; L-tryptophan from chorismate: step 5/5. {ECO:0000256|ARBA:ARBA00004733, ECO:0000256|RuleBase:RU003663}.
lightsteelblue	G41892	G41892.11	Saranac_SS	A0A445DTU8	BTB domain-containing protein	Protein modification; protein ubiquitination. {ECO:0000256|ARBA:ARBA00004906}.
lightsteelblue	G54045	G54045.1	Saranac_SS	A0A0S3RJ91	Dihydrolipoyllysine-residue succinyltransferase (EC 2.3.1.61)	Amino-acid degradation; L-lysine degradation via saccharopine pathway; glutaryl-CoA from L-lysine: step 6/6. {ECO:0000256|ARBA:ARBA00005145}.
lightsteelblue	G66391	G66391.3	Saranac_SS	A0A2I0WP31	Succinate--CoA ligase [ADP-forming] subunit alpha, mitochondrial (EC 6.2.1.5) (Succinyl-CoA synthetase subunit alpha) (SCS-alpha)	Carbohydrate metabolism; tricarboxylic acid cycle; succinate from succinyl-CoA (ligase route): step 1/1. {ECO:0000256|HAMAP-Rule:MF_03222}.
lightsteelblue	G79227	G79227.15	Saranac_SS	A0A2H5PWX6	ATP phosphoribosyltransferase (EC 2.4.2.17) (Fragment)	Amino-acid biosynthesis; L-histidine biosynthesis; L-histidine from 5-phospho-alpha-D-ribose 1-diphosphate: step 1/9. {ECO:0000256|ARBA:ARBA00004667}.
lightsteelblue	G80745	G80745.1	Saranac_SS	A0A5E4F0W4	Very-long-chain (3R)-3-hydroxyacyl-CoA dehydratase (EC 4.2.1.134)	Lipid metabolism; fatty acid biosynthesis. {ECO:0000256|ARBA:ARBA00005194, ECO:0000256|RuleBase:RU363109}.
white	G7124	G7124.7	Saranac_SS	A0A061ESX4	S-adenosylmethionine synthase (EC 2.5.1.6)	Amino-acid biosynthesis; S-adenosyl-L-methionine biosynthesis; S-adenosyl-L-methionine from L-methionine: step 1/1. {ECO:0000256|ARBA:ARBA00005224, ECO:0000256|RuleBase:RU000541}.
white	G16369	G16369.1	Saranac_SS	A0A2P5A8Z3	Mannosyltransferase (EC 2.4.1.-)	Protein modification; protein glycosylation. {ECO:0000256|ARBA:ARBA00004922}.
white	G25806	G25806.1	Saranac_SS	A0A0S3RJ91	Dihydrolipoyllysine-residue succinyltransferase (EC 2.3.1.61)	Amino-acid degradation; L-lysine degradation via saccharopine pathway; glutaryl-CoA from L-lysine: step 6/6. {ECO:0000256|ARBA:ARBA00005145}.
white	G34039	G34039.15	Saranac_SS	A0A1J7GGC5	Phosphomevalonate kinase (EC 2.7.4.2)	Isoprenoid biosynthesis; isopentenyl diphosphate biosynthesis via mevalonate pathway; isopentenyl diphosphate from (R)-mevalonate: step 2/3. {ECO:0000256|ARBA:ARBA00005017}.
white	G34826	G34826.10	Saranac_SS	A0A1S4DVC5	tRNA (guanine-N(7)-)-methyltransferase (EC 2.1.1.33) (tRNA (guanine(46)-N(7))-methyltransferase) (tRNA(m7G46)-methyltransferase)	tRNA modification; N(7)-methylguanine-tRNA biosynthesis. {ECO:0000256|HAMAP-Rule:MF_03055}.
white	G35475	G35475.2	Saranac_SS	A0A371FHR0	OMPdecase (EC 2.4.2.10) (EC 4.1.1.23) (Orotate phosphoribosyltransferase) (Orotidine 5'-phosphate decarboxylase) (Uridine 5'-monophosphate synthase) (Fragment)	Pyrimidine metabolism; UMP biosynthesis via de novo pathway; UMP from orotate: step 2/2. {ECO:0000256|ARBA:ARBA00004861}.
white	G39293	G39293.3	Saranac_SS	A0A445JC56	HECT-type E3 ubiquitin transferase (EC 2.3.2.26)	Protein modification; protein ubiquitination. {ECO:0000256|ARBA:ARBA00004906}.
white	G41225	G41225.94	Saranac_SS	A0A2C9V9M8	AMP deaminase (EC 3.5.4.6)	Purine metabolism; IMP biosynthesis via salvage pathway; IMP from AMP: step 1/1. {ECO:0000256|ARBA:ARBA00004955}.
white	G43858	G43858.1	Saranac_SS	A0A061FV80	Adenosine kinase (EC 2.7.1.20)	Purine metabolism; AMP biosynthesis via salvage pathway; AMP from adenosine: step 1/1. {ECO:0000256|ARBA:ARBA00004801}.
white	G47234	G47234.1	Saranac_SS	F6HNF4	Adenosine kinase (EC 2.7.1.20)	Purine metabolism; AMP biosynthesis via salvage pathway; AMP from adenosine: step 1/1. {ECO:0000256|ARBA:ARBA00004801}.
white	G50393	G50393.3	Saranac_SS	I1LQ58	Dolichyl-diphosphooligosaccharide--protein glycosyltransferase 48 kDa subunit (Oligosaccharyl transferase 48 kDa subunit)	Protein modification; protein glycosylation. {ECO:0000256|ARBA:ARBA00004922, ECO:0000256|RuleBase:RU361142}.
white	G55636	G55636.1	Saranac_SS	A0A1S2YFE8	Inositol oxygenase (EC 1.13.99.1) (Myo-inositol oxygenase)	Polyol metabolism; myo-inositol degradation into D-glucuronate; D-glucuronate from myo-inositol: step 1/1. {ECO:0000256|ARBA:ARBA00005167, ECO:0000256|RuleBase:RU367039}.
white	G66390	G66390.6	Saranac_SS	M5WFL7	Dolichyl-diphosphooligosaccharide--protein glycotransferase (EC 2.4.99.18)	Protein modification; protein glycosylation. {ECO:0000256|ARBA:ARBA00004922}.
white	G67478	G67478.4	Saranac_SS	M5WFL7	Dolichyl-diphosphooligosaccharide--protein glycotransferase (EC 2.4.99.18)	Protein modification; protein glycosylation. {ECO:0000256|ARBA:ARBA00004922}.

white	G74289	G74289.2	Saranac_SS	U5TMI2	Microsomal omega-6-desaturase	Lipid metabolism. {ECO:0000256|ARBA:ARBA00005189}.
white	G74289	G74289.12	Saranac_SS	U5TMI2	Microsomal omega-6-desaturase	Lipid metabolism. {ECO:0000256|ARBA:ARBA00005189}.
orangered3	G5566	G5566.1	Saranac_SS	A0A5E4FJR3	Molybdenum cofactor biosynthesis protein C (EC 4.6.1.17)	Cofactor biosynthesis; molybdopterin biosynthesis. {ECO:0000256|ARBA:ARBA00005046}.
orangered3	G10086	G10086.3	Saranac_SS	I3SWX0	SKP1-like protein	Protein modification; protein ubiquitination. {ECO:0000256|ARBA:ARBA00004906, ECO:0000256|PIRNR:PIRNR028729}.
orangered3	G28333	G28333.3	Saranac_SS	A0A1S3X040	Adenine phosphoribosyltransferase (EC 2.4.2.7)	Purine metabolism; AMP biosynthesis via salvage pathway; AMP from adenine: step 1/1. {ECO:0000256|ARBA:ARBA00004659}.
orangered3	G31563	G31563.17	Saranac_SS	A0A1J7G7Y0	Adenine phosphoribosyltransferase (EC 2.4.2.7)	Purine metabolism; AMP biosynthesis via salvage pathway; AMP from adenine: step 1/1. {ECO:0000256|ARBA:ARBA00004659}.
orangered3	G38160	G38160.19	Saranac_SS	A0A445K393	Dolichyl-diphosphooligosaccharide--protein glycosyltransferase subunit 1	Protein modification; protein glycosylation. {ECO:0000256|ARBA:ARBA00004922, ECO:0000256|RuleBase:RU361143}.
orangered3	G55928	G55928.8	Saranac_SS	A0A0R0K6G4	HECT-type E3 ubiquitin transferase (EC 2.3.2.26)	Protein modification; protein ubiquitination. {ECO:0000256|ARBA:ARBA00004906}.
orangered3	G69715	G69715.1	Saranac_SS	A0A0L9VNP6	Dolichol-phosphate mannosyltransferase subunit 3	Protein modification; protein glycosylation. {ECO:0000256|RuleBase:RU365085}.
orangered3	G78465	G78465.3	Saranac_SS	A0A5J5BZ12	Succinate dehydrogenase [ubiquinone] iron-sulfur subunit, mitochondrial (EC 1.3.5.1)	Carbohydrate metabolism; tricarboxylic acid cycle; fumarate from succinate (eukaryal route): step 1/1. {ECO:0000256|ARBA:ARBA00004788, ECO:0000256|RuleBase:RU361237}.
orangered3	G83516	G83516.1	Saranac_SS	A0A445HXW5	BTB/POZ domain-containing protein isoform A (BTB/POZ domain-containing protein isoform B) (BTB/POZ domain-containing protein isoform C)	Protein modification; protein ubiquitination. {ECO:0000256|ARBA:ARBA00004906}.
blue	G6812	G6812.3	Saranac_SS	A0A061DF70	SKP1-like protein	Protein modification; protein ubiquitination. {ECO:0000256|ARBA:ARBA00004906, ECO:0000256|PIRNR:PIRNR028729}.
blue	G8440	G8440.11	Saranac_SS	A0A2P2JDP0	Sulfate adenylyltransferase (EC 2.7.7.4)	Sulfur metabolism. {ECO:0000256|ARBA:ARBA00004678}.
blue	G8440	G8440.20	Saranac_SS	A0A371I2Y8	Sulfate adenylyltransferase (EC 2.7.7.4) (Fragment)	Sulfur metabolism. {ECO:0000256|ARBA:ARBA00004678}.
blue	G10086	G10086.1	Saranac_SS	I3SWX0	SKP1-like protein	Protein modification; protein ubiquitination. {ECO:0000256|ARBA:ARBA00004906, ECO:0000256|PIRNR:PIRNR028729}.
blue	G11581	G11581.2	Saranac_SS	A0A087HBS0	Sulfate adenylyltransferase (EC 2.7.7.4)	Sulfur metabolism. {ECO:0000256|ARBA:ARBA00004678}.
blue	G14551	G14551.3	Saranac_SS	E5GCD7	Succinate dehydrogenase [ubiquinone] iron-sulfur subunit, mitochondrial (EC 1.3.5.1)	Carbohydrate metabolism; tricarboxylic acid cycle; fumarate from succinate (eukaryal route): step 1/1. {ECO:0000256|ARBA:ARBA00004788, ECO:0000256|RuleBase:RU361237}.
blue	G17475	G17475.3	Saranac_SS	A0A5N5FI72	Lactoylglutathione lyase (EC 4.4.1.5) (Glyoxalase I)	Secondary metabolite metabolism; methylglyoxal degradation; (R)-lactate from methylglyoxal: step 1/2. {ECO:0000256|ARBA:ARBA00005008, ECO:0000256|RuleBase:RU361179}.
blue	G17475	G17475.5	Saranac_SS	A0A5N5FI72	Lactoylglutathione lyase (EC 4.4.1.5) (Glyoxalase I)	Secondary metabolite metabolism; methylglyoxal degradation; (R)-lactate from methylglyoxal: step 1/2. {ECO:0000256|ARBA:ARBA00005008, ECO:0000256|RuleBase:RU361179}.
blue	G19354	G19354.12	Saranac_SS	A0A0S3R8S2	S-adenosylmethionine synthase (EC 2.5.1.6)	Amino-acid biosynthesis; S-adenosyl-L-methionine biosynthesis; S-adenosyl-L-methionine from L-methionine: step 1/1. {ECO:0000256|ARBA:ARBA00005224, ECO:0000256|RuleBase:RU000541}.
blue	G19941	G19941.12	Saranac_SS	A0A444ZCX7	Fumarylacetoacetase (EC 3.7.1.2) (Fumarylacetoacetate hydrolase)	Amino-acid degradation; L-phenylalanine degradation; acetoacetate and fumarate from L-phenylalanine: step 6/6. {ECO:0000256|ARBA:ARBA00004782}.
blue	G20541	G20541.35	Saranac_SS	A0A2I4GDS0	Serine hydroxymethyltransferase (EC 2.1.2.1)	One-carbon metabolism; tetrahydrofolate interconversion. {ECO:0000256|ARBA:ARBA00004777, ECO:0000256|RuleBase:RU000585}.
blue	G21939	G21939.1	Saranac_SS	A0A0S3R8S2	S-adenosylmethionine synthase (EC 2.5.1.6)	Amino-acid biosynthesis; S-adenosyl-L-methionine biosynthesis; S-adenosyl-L-methionine from L-methionine: step 1/1. {ECO:0000256|ARBA:ARBA00005224, ECO:0000256|RuleBase:RU000541}.
blue	G29371	G29371.40	Saranac_SS	A0A4D6M6H2	Cellulose synthase (EC 2.4.1.12)	Glycan metabolism; plant cellulose biosynthesis. {ECO:0000256|ARBA:ARBA00004768, ECO:0000256|RuleBase:RU361116}.
blue	G35677	G35677.59	Saranac_SS	A0A445GZ81	Cellulose synthase (EC 2.4.1.12)	Glycan metabolism; plant cellulose biosynthesis. {ECO:0000256|ARBA:ARBA00004768, ECO:0000256|RuleBase:RU361116}.
blue	G42592	G42592.5	Saranac_SS	A0A1S3T8F5	RING-type E3 ubiquitin transferase (EC 2.3.2.27)	Protein modification; protein ubiquitination. {ECO:0000256|ARBA:ARBA00004906}.

blue	G46912	G46912.13	Saranac_SS	A0A5D3BUW0	Amidophosphoribosyltransferase (EC 2.4.2.14)	Purine metabolism; IMP biosynthesis via de novo pathway; N(1)-(5-phospho-D-ribosyl)glycinamide from 5-phospho-alpha-D-ribose 1-diphosphate: step 1/2. {ECO:0000256|ARBA:ARBA00005209}.
blue	G60813	G60813.10	Saranac_SS	A0A061EAW6	UDP-glucose 6-dehydrogenase (EC 1.1.1.22)	Nucleotide-sugar biosynthesis; UDP-alpha-D-glucuronate biosynthesis; UDP-alpha-D-glucuronate from UDP-alpha-D-glucose: step 1/1. {ECO:0000256|ARBA:ARBA00004701}.
blue	G71754	G71754.5	Saranac_SS	A0A5D2IIX8	F-box domain-containing protein	Protein modification; protein ubiquitination. {ECO:0000256|ARBA:ARBA00004906}.
blue	G77399	G77399.9	Saranac_SS	A0A1J7IBJ7	5-methyltetrahydropteroyltriglutamate--homocysteine S-methyltransferase (EC 2.1.1.14)	Amino-acid biosynthesis; L-methionine biosynthesis via de novo pathway; L-methionine from L-homocysteine (MetE route): step 1/1. {ECO:0000256|ARBA:ARBA00004681}.
blue	G77399	G77399.45	Saranac_SS	A0A4D6M7Z0	5-methyltetrahydropteroyltriglutamate--homocysteine S-methyltransferase (EC 2.1.1.14)	Amino-acid biosynthesis; L-methionine biosynthesis via de novo pathway; L-methionine from L-homocysteine (MetE route): step 1/1. {ECO:0000256|ARBA:ARBA00004681}.
blue	G82243	G82243.32	Saranac_SS	A0A4P1QW91	Pyrophosphate--fructose 6-phosphate 1-phosphotransferase subunit beta (PFP) (EC 2.7.1.90) (6-phosphofructokinase, pyrophosphate dependent) (PPi-PFK) (Pyrophosphate-dependent 6-phosphofructose-1-kinase)	Carbohydrate degradation; glycolysis; D-glyceraldehyde 3-phosphate and glycerone phosphate from D-glucose: step 3/4. {ECO:0000256|ARBA:ARBA00004679, ECO:0000256|HAMAP-Rule:MF_03185}.
blue	G82990	G82990.8	Saranac_SS	A0A2P5D6M2	UDP-glucose 6-dehydrogenase (EC 1.1.1.22)	Nucleotide-sugar biosynthesis; UDP-alpha-D-glucuronate biosynthesis; UDP-alpha-D-glucuronate from UDP-alpha-D-glucose: step 1/1. {ECO:0000256|ARBA:ARBA00004701}.
violet	G19821	G19821.1	Saranac_SS	Q155U2	SKP1-like protein	Protein modification; protein ubiquitination. {ECO:0000256|ARBA:ARBA00004906, ECO:0000256|PIRNR:PIRNR028729}.
violet	G21939	G21939.3	Saranac_SS	A0A0S3R8S2	S-adenosylmethionine synthase (EC 2.5.1.6)	Amino-acid biosynthesis; S-adenosyl-L-methionine biosynthesis; S-adenosyl-L-methionine from L-methionine: step 1/1. {ECO:0000256|ARBA:ARBA00005224, ECO:0000256|RuleBase:RU000541}.
violet	G51599	G51599.13	Saranac_SS	A0A2I4DI22	Glycerol kinase (EC 2.7.1.30)	Polyol metabolism; glycerol degradation via glycerol kinase pathway; sn-glycerol 3-phosphate from glycerol: step 1/1. {ECO:0000256|ARBA:ARBA00005190}.
violet	G51766	G51766.27	Saranac_SS	A0A445C2G2	Ditrans,polycis-polyprenyl diphosphate synthase ((2E,6E)-farnesyldiphosphate specific) (EC 2.5.1.87)	Protein modification; protein glycosylation. {ECO:0000256|ARBA:ARBA00004922}.
violet	G65501	G65501.5	Saranac_SS	A0A2P5ALY8	Hexosyltransferase (EC 2.4.1.-)	Glycan metabolism; pectin biosynthesis. {ECO:0000256|ARBA:ARBA00004877, ECO:0000256|RuleBase:RU362027}.
violet	G66391	G66391.5	Saranac_SS	A0A2I0WP31	Succinate--CoA ligase [ADP-forming] subunit alpha, mitochondrial (EC 6.2.1.5) (Succinyl-CoA synthetase subunit alpha) (SCS-alpha)	Carbohydrate metabolism; tricarboxylic acid cycle; succinate from succinyl-CoA (ligase route): step 1/1. {ECO:0000256|HAMAP-Rule:MF_03222}.
violet	G80465	G80465.16	Saranac_SS	A0A445EXC6	Uncharacterized protein	Protein modification; protein ubiquitination. {ECO:0000256|ARBA:ARBA00004906}.
mediumorchid	G7079	G7079.45	Saranac_SS	I1NAH7	Pyruvate kinase (EC 2.7.1.40)	Carbohydrate degradation; glycolysis; pyruvate from D-glyceraldehyde 3-phosphate: step 5/5. {ECO:0000256|ARBA:ARBA00004997, ECO:0000256|RuleBase:RU000504}.
mediumorchid	G10540	G10540.32	Saranac_SS	A0A2R6PQ01	Succinate--CoA ligase [ADP-forming] subunit beta, mitochondrial (EC 6.2.1.5) (Succinyl-CoA synthetase beta chain) (SCS-beta)	Carbohydrate metabolism; tricarboxylic acid cycle; succinate from succinyl-CoA (ligase route): step 1/1. {ECO:0000256|ARBA:ARBA00005064, ECO:0000256|HAMAP-Rule:MF_03219}.
mediumorchid	G41713	G41713.42	Saranac_SS	M5VVH3	Phosphotransferase (EC 2.7.1.-)	Carbohydrate degradation; glycolysis; D-glyceraldehyde 3-phosphate and glycerone phosphate from D-glucose: step 1/4. {ECO:0000256|ARBA:ARBA00004888}.; Carbohydrate metabolism; hexose metabolism. {ECO:0000256|ARBA:ARBA00005028}.
mediumorchid	G49920	G49920.6	Saranac_SS	B9SBM7	Pyruvate kinase (EC 2.7.1.40)	Carbohydrate degradation; glycolysis; pyruvate from D-glyceraldehyde 3-phosphate: step 5/5. {ECO:0000256|ARBA:ARBA00004997, ECO:0000256|RuleBase:RU000504}.
mediumorchid	G77741	G77741.2	Saranac_SS	A0A1R3K005	Phosphomannomutase (EC 5.4.2.8)	Nucleotide-sugar biosynthesis; GDP-alpha-D-mannose biosynthesis; alpha-D-mannose 1-phosphate from D-fructose 6-phosphate: step 2/2. {ECO:0000256|ARBA:ARBA00004699, ECO:0000256|RuleBase:RU361118}.
plum	G37147	G37147.2	Saranac_SS	A0A1U7ZKJ3	3-oxoacyl-[acyl-carrier-protein] reductase (EC 1.1.1.100)	Lipid metabolism; fatty acid biosynthesis. {ECO:0000256|RuleBase:RU366074}.

navajowhite2	G3969	G3969.14	Saranac_SS	A0A5E4E3X7	S-adenosylmethionine synthase (EC 2.5.1.6)	Amino-acid biosynthesis; S-adenosyl-L-methionine biosynthesis; S-adenosyl-L-methionine from L-methionine: step 1/1. {ECO:0000256|ARBA:ARBA00005224, ECO:0000256|RuleBase:RU000541}.
navajowhite2	G5429	G5429.1	Saranac_SS	A0A0B0MXI6	Fructose-bisphosphate aldolase (EC 4.1.2.13)	Carbohydrate degradation; glycolysis; D-glyceraldehyde 3-phosphate and glycerone phosphate from D-glucose: step 4/4. {ECO:0000256|ARBA:ARBA00004714}.
navajowhite2	G7309	G7309.22	Saranac_SS	A0A371G7Q9	Succinate--CoA ligase [ADP-forming] subunit beta, mitochondrial (EC 6.2.1.5) (Succinyl-CoA synthetase beta chain) (SCS-beta)	Carbohydrate metabolism; tricarboxylic acid cycle; succinate from succinyl-CoA (ligase route): step 1/1. {ECO:0000256|ARBA:ARBA00005064, ECO:0000256|HAMAP-Rule:MF_03219}.
navajowhite2	G10381	G10381.2	Saranac_SS	A0A061ESX4	S-adenosylmethionine synthase (EC 2.5.1.6)	Amino-acid biosynthesis; S-adenosyl-L-methionine biosynthesis; S-adenosyl-L-methionine from L-methionine: step 1/1. {ECO:0000256|ARBA:ARBA00005224, ECO:0000256|RuleBase:RU000541}.
navajowhite2	G11898	G11898.9	Saranac_SS	A0A4Y7KDI9	Urea amidohydrolase (EC 3.5.1.5) (Urease)	Nitrogen metabolism; urea degradation; CO(2) and NH(3) from urea (urease route): step 1/1. {ECO:0000256|ARBA:ARBA00004897}.
navajowhite2	G15042	G15042.22	Saranac_SS	A0A1J7H315	D-3-phosphoglycerate dehydrogenase (EC 1.1.1.95)	Amino-acid biosynthesis; L-serine biosynthesis; L-serine from 3-phospho-D-glycerate: step 1/3. {ECO:0000256|ARBA:ARBA00005216, ECO:0000256|RuleBase:RU363003}.
navajowhite2	G15226	G15226.2	Saranac_SS	A0A4Y7KDI9	Urea amidohydrolase (EC 3.5.1.5) (Urease)	Nitrogen metabolism; urea degradation; CO(2) and NH(3) from urea (urease route): step 1/1. {ECO:0000256|ARBA:ARBA00004897}.
navajowhite2	G21691	G21691.16	Saranac_SS	A0A3Q7HGJ9	Phosphoglycerate kinase (EC 2.7.2.3)	Carbohydrate degradation; glycolysis; pyruvate from D-glyceraldehyde 3-phosphate: step 2/5. {ECO:0000256|ARBA:ARBA00004838}.
navajowhite2	G34261	G34261.3	Saranac_SS	A0A2I4GLE6	BTB/POZ domain-containing protein At2g13690-like	Protein modification; protein ubiquitination. {ECO:0000256|ARBA:ARBA00004906}.
navajowhite2	G35106	G35106.22	Saranac_SS	V7ARM2	Dolichyl-diphosphooligosaccharide--protein glycosyltransferase subunit 1	Protein modification; protein glycosylation. {ECO:0000256|ARBA:ARBA00004922, ECO:0000256|RuleBase:RU361143}.
navajowhite2	G54773	G54773.112	Saranac_SS	A0A444WS55	Succinate dehydrogenase [ubiquinone] flavoprotein subunit, mitochondrial (EC 1.3.5.1)	Carbohydrate metabolism; tricarboxylic acid cycle; fumarate from succinate (eukaryal route): step 1/1. {ECO:0000256|RuleBase:RU362051}.
navajowhite2	G55683	G55683.8	Saranac_SS	B0LW67	Adenosylmethionine decarboxylase (EC 4.1.1.50) (Fragment)	Amine and polyamine biosynthesis; S-adenosylmethioninamine biosynthesis; S-adenosylmethioninamine from S-adenosyl-L-methionine: step 1/1. {ECO:0000256|ARBA:ARBA00004911}.
navajowhite2	G57772	G57772.1	Saranac_SS	A0A5J4ZW39	Dolichyl-diphosphooligosaccharide--protein glycosyltransferase subunit DAD1 (Oligosaccharyl transferase subunit DAD1)	Protein modification; protein glycosylation. {ECO:0000256|ARBA:ARBA00004922, ECO:0000256|RuleBase:RU361136}.
navajowhite2	G65275	G65275.9	Saranac_SS	A0A5D2DPK6	Glucose-6-phosphate isomerase (EC 5.3.1.9)	Carbohydrate degradation; glycolysis; D-glyceraldehyde 3-phosphate and glycerone phosphate from D-glucose: step 2/4. {ECO:0000256|ARBA:ARBA00004926, ECO:0000256|RuleBase:RU000612}.
navajowhite2	G67478	G67478.2	Saranac_SS	M5WFL7	Dolichyl-diphosphooligosaccharide--protein glycotransferase (EC 2.4.99.18)	Protein modification; protein glycosylation. {ECO:0000256|ARBA:ARBA00004922}.
navajowhite2	G71021	G71021.49	Saranac_SS	A0A2G5DIQ4	Phosphopyruvate hydratase (EC 4.2.1.11)	Carbohydrate degradation; glycolysis; pyruvate from D-glyceraldehyde 3-phosphate: step 4/5. {ECO:0000256|ARBA:ARBA00005031}.
navajowhite2	G73951	G73951.47	Saranac_SS	A0A445D4P9	Phosphopyruvate hydratase (EC 4.2.1.11)	Carbohydrate degradation; glycolysis; pyruvate from D-glyceraldehyde 3-phosphate: step 4/5. {ECO:0000256|ARBA:ARBA00005031}.
navajowhite2	G75080	G75080.3	Saranac_SS	A0A3Q7XUG7	omega-3 fatty acid desaturase, chloroplastic-like	Lipid metabolism. {ECO:0000256|ARBA:ARBA00005189}.
navajowhite2	G75387	G75387.1	Saranac_SS	A0A4D6NFI4	Uncharacterized protein	Glycan metabolism; pectin biosynthesis. {ECO:0000256|ARBA:ARBA00004877}.
darkolivegreen	G3289	G3289.4	Wilson_DS	A0A5N5GB72	E3 ubiquitin-protein ligase RNF217	Protein modification; protein ubiquitination. {ECO:0000256|ARBA:ARBA00004906}.
darkolivegreen	G7321	G7321.30	Wilson_DS	A0A2K3P663	Dolichyl-diphosphooligosaccharide--protein glycosyltransferase subunit 2 (Ribophorin-2) (Fragment)	Protein modification; protein glycosylation. {ECO:0000256|RuleBase:RU366029}.
darkolivegreen	G12675	G12675.4	Wilson_DS	A0A445BSD5	Pectinesterase (EC 3.1.1.11)	Glycan metabolism; pectin degradation; 2-dehydro-3-deoxy-D-gluconate from pectin: step 1/5. {ECO:0000256|ARBA:ARBA00005184, ECO:0000256|RuleBase:RU000589}.
darkolivegreen	G13617	G13617.39	Wilson_DS	A0A2P6SJC1	Adenosine kinase (EC 2.7.1.20)	Purine metabolism; AMP biosynthesis via salvage pathway; AMP from adenosine: step 1/1. {ECO:0000256|ARBA:ARBA00004801}.
darkolivegreen	G20849	G20849.7	Wilson_DS	A0A2K3LEQ2	Putative E3 ubiquitin-protein ligase ARI7-like protein (Fragment)	Protein modification; protein ubiquitination. {ECO:0000256|ARBA:ARBA00004906}.
darkolivegreen	G26511	G26511.7	Wilson_DS	A0A0R0L809	Hexosyltransferase (EC 2.4.1.-)	Glycan metabolism; pectin biosynthesis. {ECO:0000256|ARBA:ARBA00004877, ECO:0000256|RuleBase:RU362027}.

darkolivegreen	G31075	G31075.3	Wilson_DS	A0A0B2R0N3	Chalcone-flavonone isomerase family protein	Secondary metabolite biosynthesis; flavonoid biosynthesis. {ECO:0000256|ARBA:ARBA00004966}.
darkolivegreen	G37082	G37082.7	Wilson_DS	I3SEQ9	Serine hydroxymethyltransferase (EC 2.1.2.1)	One-carbon metabolism; tetrahydrofolate interconversion. {ECO:0000256|ARBA:ARBA00004777, ECO:0000256|RuleBase:RU000585}.
darkolivegreen	G49222	G49222.25	Wilson_DS	A0A0B2QRR9	RING-type E3 ubiquitin transferase (EC 2.3.2.27)	Protein modification; protein ubiquitination. {ECO:0000256|ARBA:ARBA00004906}.
darkolivegreen	G58551	G58551.2	Wilson_DS	A0A314XST5	Fructose-bisphosphate aldolase (EC 4.1.2.13)	Carbohydrate degradation; glycolysis; D-glyceraldehyde 3-phosphate and glycerone phosphate from D-glucose: step 4/4. {ECO:0000256|ARBA:ARBA00004714}.
darkolivegreen	G61987	G61987.3	Wilson_DS	A0A2R6QJ67	Phosphopyruvate hydratase (EC 4.2.1.11)	Carbohydrate degradation; glycolysis; pyruvate from D-glyceraldehyde 3-phosphate: step 4/5. {ECO:0000256|ARBA:ARBA00005031}.
darkolivegreen	G65093	G65093.8	Wilson_DS	A0A445D3V5	Pyruvate kinase (EC 2.7.1.40)	Carbohydrate degradation; glycolysis; pyruvate from D-glyceraldehyde 3-phosphate: step 5/5. {ECO:0000256|ARBA:ARBA00004997, ECO:0000256|RuleBase:RU000504}.
darkolivegreen	G74050	G74050.1	Wilson_DS	M5W5K0	Pyruvate kinase (EC 2.7.1.40)	Carbohydrate degradation; glycolysis; pyruvate from D-glyceraldehyde 3-phosphate: step 5/5. {ECO:0000256|ARBA:ARBA00004997, ECO:0000256|RuleBase:RU000504}.
darkolivegreen	G74551	G74551.4	Wilson_DS	A0A1J7IBJ7	5-methyltetrahydropteroyltriglutamate--homocysteine S-methyltransferase (EC 2.1.1.14)	Amino-acid biosynthesis; L-methionine biosynthesis via de novo pathway; L-methionine from L-homocysteine (MetE route): step 1/1. {ECO:0000256|ARBA:ARBA00004681}.
darkolivegreen	G78145	G78145.89	Wilson_DS	A0A3Q0ETC7	E3 ubiquitin-protein ligase (EC 2.3.2.27)	Protein modification; protein ubiquitination. {ECO:0000256|RuleBase:RU366018}.
darkolivegreen	G78300	G78300.1	Wilson_DS	A0A1S2Z4Q1	Hexosyltransferase (EC 2.4.1.-)	Glycan metabolism; pectin biosynthesis. {ECO:0000256|ARBA:ARBA00004877, ECO:0000256|RuleBase:RU362027}.
brown	G5380	G5380.1	Wilson_DS	A0A1J7H315	D-3-phosphoglycerate dehydrogenase (EC 1.1.1.95)	Amino-acid biosynthesis; L-serine biosynthesis; L-serine from 3-phospho-D-glycerate: step 1/3. {ECO:0000256|ARBA:ARBA00005216, ECO:0000256|RuleBase:RU363003}.
brown	G6497	G6497.7	Wilson_DS	A0A061FEW0	Pyrophosphate--fructose 6-phosphate 1-phosphotransferase subunit alpha (PFP) (6-phosphofructokinase, pyrophosphate dependent) (PPi-PFK) (Pyrophosphate-dependent 6-phosphofructose-1-kinase)	Carbohydrate degradation; glycolysis; D-glyceraldehyde 3-phosphate and glycerone phosphate from D-glucose: step 3/4. {ECO:0000256|ARBA:ARBA00004679, ECO:0000256|HAMAP-Rule:MF_03185}.
brown	G7828	G7828.14	Wilson_DS	A0A2I4G6B1	Pyruvate kinase (EC 2.7.1.40)	Carbohydrate degradation; glycolysis; pyruvate from D-glyceraldehyde 3-phosphate: step 5/5. {ECO:0000256|ARBA:ARBA00004997, ECO:0000256|RuleBase:RU000504}.
brown	G8783	G8783.7	Wilson_DS	A0A4Y7KDI9	Urea amidohydrolase (EC 3.5.1.5) (Urease)	Nitrogen metabolism; urea degradation; CO(2) and NH(3) from urea (urease route): step 1/1. {ECO:0000256|ARBA:ARBA00004897}.
brown	G13778	G13778.12	Wilson_DS	A0A4D6N2W3	Dolichyl-diphosphooligosaccharide--protein glycosyltransferase subunit 2 (Ribophorin-2)	Protein modification; protein glycosylation. {ECO:0000256|RuleBase:RU366029}.
brown	G14031	G14031.13	Wilson_DS	A0A5B7AAU1	Pyruvate kinase (EC 2.7.1.40)	Carbohydrate degradation; glycolysis; pyruvate from D-glyceraldehyde 3-phosphate: step 5/5. {ECO:0000256|ARBA:ARBA00004997, ECO:0000256|RuleBase:RU000504}.
brown	G17642	G17642.4	Wilson_DS	A0A1J7GHA3	Serine hydroxymethyltransferase (EC 2.1.2.1)	One-carbon metabolism; tetrahydrofolate interconversion. {ECO:0000256|ARBA:ARBA00004777, ECO:0000256|RuleBase:RU000585}.
brown	G17724	G17724.1	Wilson_DS	A0A3G1VTM9	Hexosyltransferase (EC 2.4.1.-)	Glycan metabolism; pectin biosynthesis. {ECO:0000256|ARBA:ARBA00004877, ECO:0000256|RuleBase:RU362027}.
brown	G23656	G23656.13	Wilson_DS	A0A444Y9U9	Carbamoyl-phosphate synthase (glutamine-hydrolyzing) (EC 6.3.5.5)	Pyrimidine metabolism; UMP biosynthesis via de novo pathway; (S)-dihydroorotate from bicarbonate: step 1/3. {ECO:0000256|ARBA:ARBA00004812}.
brown	G26886	G26886.9	Wilson_DS	A0A384RFK9	Microsomal delta 15 desaturase	Lipid metabolism. {ECO:0000256|ARBA:ARBA00005189}.
brown	G28699	G28699.7	Wilson_DS	A0A444XRV8	Uncharacterized protein	Glycolipid biosynthesis; glycosylphosphatidylinositol-anchor biosynthesis. {ECO:0000256|ARBA:ARBA00004687}.
brown	G40306	G40306.7	Wilson_DS	A0A067FZR7	RING-type E3 ubiquitin transferase (EC 2.3.2.27)	Protein modification; protein ubiquitination. {ECO:0000256|ARBA:ARBA00004906}.
brown	G54603	G54603.14	Wilson_DS	A0A445F4R7	BTB/POZ domain-containing protein NPY2 isoform C (BTB/POZ domain-containing protein NPY2 isoform D)	Protein modification; protein ubiquitination. {ECO:0000256|ARBA:ARBA00004906}.
brown	G59911	G59911.23	Wilson_DS	I1MVM4	Uncharacterized protein	Protein modification; protein ubiquitination. {ECO:0000256|ARBA:ARBA00004906}.
brown	G70858	G70858.1	Wilson_DS	A0A444ZK95	GDP-L-fucose synthase (EC 1.1.1.271)	Nucleotide-sugar biosynthesis; GDP-L-fucose biosynthesis via de novo pathway; GDP-L-fucose from GDP-alpha-D-mannose: step 2/2. {ECO:0000256|ARBA:ARBA00004883}.
brown	G74289	G74289.16	Wilson_DS	U5TMI2	Microsomal omega-6-desaturase	Lipid metabolism. {ECO:0000256|ARBA:ARBA00005189}.

brown	G75442	G75442.1	Wilson_DS	A0A1S2XNU4	Pectinesterase (EC 3.1.1.11)	Glycan metabolism; pectin degradation; 2-dehydro-3-deoxy-D-gluconate from pectin: step 1/5. {ECO:0000256|ARBA:ARBA00005184, ECO:0000256|RuleBase:RU000589}.
brown	G75445	G75445.3	Wilson_DS	V7CGC4	Pectinesterase (EC 3.1.1.11)	Glycan metabolism; pectin degradation; 2-dehydro-3-deoxy-D-gluconate from pectin: step 1/5. {ECO:0000256|ARBA:ARBA00005184, ECO:0000256|RuleBase:RU000589}.
brown	G77128	G77128.16	Wilson_DS	A0A444ZJY2	Adenylosuccinate synthetase, chloroplastic (AMPSase) (AdSS) (EC 6.3.4.4) (IMP--aspartate ligase)	Purine metabolism; AMP biosynthesis via de novo pathway; AMP from IMP: step 1/2. {ECO:0000256|HAMAP-Rule:MF_03125, ECO:0000256|RuleBase:RU000520}.
brown	G82257	G82257.6	Wilson_DS	A0A067JAG7	Glyoxalase I (EC 4.4.1.5)	Secondary metabolite metabolism; methylglyoxal degradation; (R)-lactate from methylglyoxal: step 1/2. {ECO:0000256|ARBA:ARBA00005008}.
brown	G84492	G84492.27	Wilson_DS	V7AQP2	Hexosyltransferase (EC 2.4.1.-)	Glycan metabolism; pectin biosynthesis. {ECO:0000256|ARBA:ARBA00004877, ECO:0000256|RuleBase:RU362027}.
brown	G88489	G88489.20	Wilson_DS	A0A445GRZ7	Lactoylglutathione lyase (EC 4.4.1.5) (Glyoxalase I)	Secondary metabolite metabolism; methylglyoxal degradation; (R)-lactate from methylglyoxal: step 1/2. {ECO:0000256|ARBA:ARBA00005008, ECO:0000256|RuleBase:RU361179}.
red	G3969	G3969.13	Wilson_DS	A0A061ESX4	S-adenosylmethionine synthase (EC 2.5.1.6)	Amino-acid biosynthesis; S-adenosyl-L-methionine biosynthesis; S-adenosyl-L-methionine from L-methionine: step 1/1. {ECO:0000256|ARBA:ARBA00005224, ECO:0000256|RuleBase:RU000541}.
red	G5636	G5636.13	Wilson_DS	A0A2I0A245	Formyltetrahydrofolate synthetase (EC 6.3.4.3)	One-carbon metabolism; tetrahydrofolate interconversion. {ECO:0000256|ARBA:ARBA00004777}.
red	G11153	G11153.1	Wilson_DS	A0A444ZLG2	4-hydroxy-tetrahydrodipicolinate synthase (EC 4.3.3.7)	Amino-acid biosynthesis; L-lysine biosynthesis via DAP pathway; (S)-tetrahydrodipicolinate from L-aspartate: step 3/4. {ECO:0000256|ARBA:ARBA00005120}.
red	G12676	G12676.10	Wilson_DS	A0A2K3KZB5	RING-type E3 ubiquitin transferase (EC 2.3.2.27)	Protein modification; protein ubiquitination. {ECO:0000256|ARBA:ARBA00004906}.
red	G33122	G33122.1	Wilson_DS	A0A1J6KCE8	Deoxyuridine 5'-triphosphate nucleotidohydrolase (dUTPase) (EC 3.6.1.23) (dUTP pyrophosphatase)	Pyrimidine metabolism; dUMP biosynthesis; dUMP from dCTP (dUTP route): step 2/2. {ECO:0000256|ARBA:ARBA00005142, ECO:0000256|RuleBase:RU367024}.
red	G35544	G35544.1	Wilson_DS	A0A4P1RHA5	Dolichyl-diphosphooligosaccharide--protein glycosyltransferase subunit DAD1 (Oligosaccharyl transferase subunit DAD1)	Protein modification; protein glycosylation. {ECO:0000256|ARBA:ARBA00004922, ECO:0000256|RuleBase:RU361136}.
red	G55683	G55683.5	Wilson_DS	B0LW67	Adenosylmethionine decarboxylase (EC 4.1.1.50) (Fragment)	Amine and polyamine biosynthesis; S-adenosylmethioninamine biosynthesis; S-adenosylmethioninamine from S-adenosyl-L-methionine: step 1/1. {ECO:0000256|ARBA:ARBA00004911}.
red	G61945	G61945.2	Wilson_DS	A0A2Z6LT42	AICAR transformylase (EC 2.1.2.3) (EC 3.5.4.10) (IMP synthase) (Inosinicase) (Phosphoribosylaminoimidazolecarboxamide formyltransferase)	Purine metabolism; IMP biosynthesis via de novo pathway; 5-formamido-1-(5-phospho-D-ribosyl)imidazole-4-carboxamide from 5-amino-1-(5-phospho-D-ribosyl)imidazole-4-carboxamide (10-formyl THF route): step 1/1. {ECO:0000256|ARBA:ARBA00004954}.; Purine metabolism; IMP biosynthesis via de novo pathway; IMP from 5-formamido-1-(5-phospho-D-ribosyl)imidazole-4-carboxamide: step 1/1. {ECO:0000256|ARBA:ARBA00004844}.
red	G73129	G73129.7	Wilson_DS	A0A2R6P9N1	UDP-glucose 6-dehydrogenase (EC 1.1.1.22)	Nucleotide-sugar biosynthesis; UDP-alpha-D-glucuronate biosynthesis; UDP-alpha-D-glucuronate from UDP-alpha-D-glucose: step 1/1. {ECO:0000256|ARBA:ARBA00004701}.
red	G77127	G77127.30	Wilson_DS	A0A4D6MUU0	1-acylglycerol-3-phosphate O-acyltransferase (EC 2.3.1.51)	Lipid metabolism. {ECO:0000256|ARBA:ARBA00005189}.; Phospholipid metabolism; CDP-diacylglycerol biosynthesis; CDP-diacylglycerol from sn-glycerol 3-phosphate: step 2/3. {ECO:0000256|ARBA:ARBA00004728}.
red	G77128	G77128.38	Wilson_DS	A0A0L9TAA3	Adenylosuccinate synthetase, chloroplastic (AMPSase) (AdSS) (EC 6.3.4.4) (IMP--aspartate ligase)	Purine metabolism; AMP biosynthesis via de novo pathway; AMP from IMP: step 1/2. {ECO:0000256|HAMAP-Rule:MF_03125, ECO:0000256|RuleBase:RU000520}.
red	G82590	G82590.6	Wilson_DS	A0A0L9TS94	Pectinesterase (EC 3.1.1.11)	Glycan metabolism; pectin degradation; 2-dehydro-3-deoxy-D-gluconate from pectin: step 1/5. {ECO:0000256|ARBA:ARBA00005184, ECO:0000256|RuleBase:RU000589}.
red	G85842	G85842.2	Wilson_DS	A0A4P1RU79	Sucrose-phosphate synthase (EC 2.4.1.14)	Glycan biosynthesis; sucrose biosynthesis; sucrose from D-fructose 6-phosphate and UDP-alpha-D-glucose: step 1/2. {ECO:0000256|ARBA:ARBA00005027}.
maroon	G5769	G5769.6	Wilson_DS	A0A5N6RB15	DDB1- and CUL4-associated factor 13 (WD repeat and SOF domain-containing protein 1)	Protein modification; protein ubiquitination. {ECO:0000256|ARBA:ARBA00004906}.
maroon	G43785	G43785.22	Wilson_DS	A0A5B6ZX12	RING-type E3 ubiquitin transferase (EC 2.3.2.27)	Protein modification; protein ubiquitination. {ECO:0000256|ARBA:ARBA00004906}.
maroon	G44981	G44981.2	Wilson_DS	A0A0B0MVI6	Phosphatidyl-N-methylethanolamine N-methyltransferase (EC 2.1.1.71) (Phospholipid methyltransferase) (PLMT)	Phospholipid metabolism; phosphatidylcholine biosynthesis. {ECO:0000256|ARBA:ARBA00004969, ECO:0000256|HAMAP-Rule:MF_03216}.

maroon	G68113	G68113.12	Wilson_DS	A0A444ZK95	GDP-L-fucose synthase (EC 1.1.1.271)	Nucleotide-sugar biosynthesis; GDP-L-fucose biosynthesis via de novo pathway; GDP-L-fucose from GDP-alpha-D-mannose: step 2/2. {ECO:0000256|ARBA:ARBA00004883}.
maroon	G72557	G72557.2	Wilson_DS	V7CGC4	Pectinesterase (EC 3.1.1.11)	Glycan metabolism; pectin degradation; 2-dehydro-3-deoxy-D-gluconate from pectin: step 1/5. {ECO:0000256|ARBA:ARBA00005184, ECO:0000256|RuleBase:RU000589}.
maroon	G73129	G73129.2	Wilson_DS	A0A0L9VNF1	UDP-glucose 6-dehydrogenase (EC 1.1.1.22)	Nucleotide-sugar biosynthesis; UDP-alpha-D-glucuronate biosynthesis; UDP-alpha-D-glucuronate from UDP-alpha-D-glucose: step 1/1. {ECO:0000256|ARBA:ARBA00004701}.
lightcyan	G17741	G17741.13	Wilson_DS	A0A2N9HPA3	1-(5-phosphoribosyl)-5-[(5-phosphoribosylamino)methylideneamino] imidazole-4-carboxamide isomerase, chloroplastic (EC 5.3.1.16) (5-proFAR isomerase) (Phosphoribosylformimino-5-aminoimidazole carboxamide ribotide isomerase)	Amino-acid biosynthesis; L-histidine biosynthesis; L-histidine from 5-phospho-alpha-D-ribose 1-diphosphate: step 4/9. {ECO:0000256|ARBA:ARBA00005133, ECO:0000256|RuleBase:RU364022}.
lightcyan	G50021	G50021.27	Wilson_DS	A0A498HI13	Acetohydroxy-acid reductoisomerase (Alpha-keto-beta-hydroxylacyl reductoisomerase)	Amino-acid biosynthesis; L-isoleucine biosynthesis; L-isoleucine from 2-oxobutanoate: step 2/4. {ECO:0000256|ARBA:ARBA00004885}.; Amino-acid biosynthesis; L-valine biosynthesis; L-valine from pyruvate: step 2/4. {ECO:0000256|ARBA:ARBA00004864}.
lightcyan	G58166	G58166.8	Wilson_DS	A0A061EAW6	UDP-glucose 6-dehydrogenase (EC 1.1.1.22)	Nucleotide-sugar biosynthesis; UDP-alpha-D-glucuronate biosynthesis; UDP-alpha-D-glucuronate from UDP-alpha-D-glucose: step 1/1. {ECO:0000256|ARBA:ARBA00004701}.
lightcyan	G87483	G87483.26	Wilson_DS	A0A076JE29	Cellulose synthase (EC 2.4.1.12)	Glycan metabolism; plant cellulose biosynthesis. {ECO:0000256|ARBA:ARBA00004768, ECO:0000256|RuleBase:RU361116}.
skyblue3	G4976	G4976.26	PI467895_SS	B9SLI0	Sucrose transport protein, putative	Glycan biosynthesis; sucrose metabolism. {ECO:0000256|ARBA:ARBA00004914}.
skyblue3	G40053	G40053.2	PI467895_SS	A0A2P5FQ65	Amidophosphoribosyltransferase (EC 2.4.2.14)	Purine metabolism; IMP biosynthesis via de novo pathway; N(1)-(5-phospho-D-ribosyl)glycinamide from 5-phospho-alpha-D-ribose 1-diphosphate: step 1/2. {ECO:0000256|ARBA:ARBA00005209}.
skyblue3	G50021	G50021.17	PI467895_SS	M5X0L5	Ketol-acid reductoisomerase (EC 1.1.1.86) (Acetohydroxy-acid reductoisomerase) (Alpha-keto-beta-hydroxylacyl reductoisomerase)	Amino-acid biosynthesis; L-isoleucine biosynthesis; L-isoleucine from 2-oxobutanoate: step 2/4. {ECO:0000256|ARBA:ARBA00004885, ECO:0000256|PIRNR:PIRNR000118}.; Amino-acid biosynthesis; L-valine biosynthesis; L-valine from pyruvate: step 2/4. {ECO:0000256|ARBA:ARBA00004864, ECO:0000256|PIRNR:PIRNR000118}.
skyblue3	G51961	G51961.4	PI467895_SS	A0A1S2Z8A2	Adenine phosphoribosyltransferase (EC 2.4.2.7)	Purine metabolism; AMP biosynthesis via salvage pathway; AMP from adenine: step 1/1. {ECO:0000256|ARBA:ARBA00004659}.
skyblue3	G60454	G60454.2	PI467895_SS	A0A151TH94	Glycogen synthase (Fragment)	Glycan biosynthesis; starch biosynthesis. {ECO:0000256|ARBA:ARBA00004727}.
skyblue3	G76366	G76366.1	PI467895_SS	A0A2P5DKN6	Ribose-5-phosphate isomerase (EC 5.3.1.6)	Carbohydrate degradation; pentose phosphate pathway; D-ribose 5-phosphate from D-ribulose 5-phosphate (non-oxidative stage): step 1/1. {ECO:0000256|ARBA:ARBA00004988}.
skyblue3	G76618	G76618.65	PI467895_SS	I1JPY7	1,4-alpha-glucan branching enzyme (EC 2.4.1.18)	Glycan biosynthesis; starch biosynthesis. {ECO:0000256|ARBA:ARBA00004727}.
skyblue3	G79990	G79990.8	PI467895_SS	A0A540MEK0	1-deoxy-D-xylulose-5-phosphate reductoisomerase (EC 1.1.1.267)	Isoprenoid biosynthesis; isopentenyl diphosphate biosynthesis via DXP pathway; isopentenyl diphosphate from 1-deoxy-D-xylulose 5-phosphate: step 1/6. {ECO:0000256|ARBA:ARBA00005094}.
skyblue3	G81870	G81870.27	PI467895_SS	A0A445JQD6	Lipoxygenase (EC 1.13.11.-)	Lipid metabolism; oxylipin biosynthesis. {ECO:0000256|RuleBase:RU003975}.
skyblue3	G84643	G84643.2	PI467895_SS	A0A1J7GVN1	Serine O-acetyltransferase (EC 2.3.1.30)	Amino-acid biosynthesis; L-cysteine biosynthesis; L-cysteine from L-serine: step 1/2. {ECO:0000256|ARBA:ARBA00004876}.
skyblue3	G87941	G87941.99	PI467895_SS	A0A445JQL7	Lipoxygenase (EC 1.13.11.-)	Lipid metabolism; oxylipin biosynthesis. {ECO:0000256|RuleBase:RU003975}.
thistle1	G16816	G16816.7	PI467895_SS	A0A0S3R8S2	S-adenosylmethionine synthase (EC 2.5.1.6)	Amino-acid biosynthesis; S-adenosyl-L-methionine biosynthesis; S-adenosyl-L-methionine from L-methionine: step 1/1. {ECO:0000256|ARBA:ARBA00005224, ECO:0000256|RuleBase:RU000541}.
thistle1	G27554	G27554.3	PI467895_SS	A0A4D6MDV6	Adenosylhomocysteinase (EC 3.3.1.1)	Amino-acid biosynthesis; L-homocysteine biosynthesis; L-homocysteine from S-adenosyl-L-homocysteine: step 1/1. {ECO:0000256|ARBA:ARBA00005195, ECO:0000256|RuleBase:RU000548}.
thistle1	G30031	G30031.1	PI467895_SS	A0A072UXQ2	Phosphatidylinositol N-acetylglucosaminyltransferase (EC 2.4.1.198)	Glycolipid biosynthesis; glycosylphosphatidylinositol-anchor biosynthesis. {ECO:0000256|ARBA:ARBA00004687}.
thistle1	G32700	G32700.2	PI467895_SS	A0A443P2U2	Tryptophan synthase (EC 4.2.1.20)	Amino-acid biosynthesis; L-tryptophan biosynthesis; L-tryptophan from chorismate: step 5/5. {ECO:0000256|ARBA:ARBA00004733, ECO:0000256|RuleBase:RU003663}.
thistle1	G71644	G71644.74	PI467895_SS	A0A4D6M7Z0	5-methyltetrahydropteroyltriglutamate--homocysteine S-methyltransferase (EC 2.1.1.14)	Amino-acid biosynthesis; L-methionine biosynthesis via de novo pathway; L-methionine from L-homocysteine (MetE route): step 1/1. {ECO:0000256|ARBA:ARBA00004681}.

thistle1	G74551	G74551.36	PI467895_SS	A0A1J7IBJ7	5-methyltetrahydropteroyltriglutamate--homocysteine S-methyltransferase (EC 2.1.1.14)	Amino-acid biosynthesis; L-methionine biosynthesis via de novo pathway; L-methionine from L-homocysteine (MetE route): step 1/1. {ECO:0000256|ARBA:ARBA00004681}.
thistle1	G77399	G77399.30	PI467895_SS	A0A1J7IBJ7	5-methyltetrahydropteroyltriglutamate--homocysteine S-methyltransferase (EC 2.1.1.14)	Amino-acid biosynthesis; L-methionine biosynthesis via de novo pathway; L-methionine from L-homocysteine (MetE route): step 1/1. {ECO:0000256|ARBA:ARBA00004681}.
midnightblue	G4390	G4390.47	PI467895_SS	A0A3P9QN32	Starch synthase, chloroplastic/amyloplastic (EC 2.4.1.-)	Glycan biosynthesis; starch biosynthesis. {ECO:0000256|ARBA:ARBA00004727, ECO:0000256|RuleBase:RU361232}.
midnightblue	G6812	G6812.1	PI467895_SS	A0A061DF70	SKP1-like protein	Protein modification; protein ubiquitination. {ECO:0000256|ARBA:ARBA00004906, ECO:0000256|PIRNR:PIRNR028729}.
midnightblue	G24479	G24479.5	PI467895_SS	A0A1R3KEY6	Phosphopyruvate hydratase (EC 4.2.1.11)	Carbohydrate degradation; glycolysis; pyruvate from D-glyceraldehyde 3-phosphate: step 4/5. {ECO:0000256|ARBA:ARBA00005031}.
midnightblue	G35078	G35078.85	PI467895_SS	A0A3Q7YFB6	1,4-alpha-glucan branching enzyme (EC 2.4.1.18)	Glycan biosynthesis; starch biosynthesis. {ECO:0000256|ARBA:ARBA00004727}.
midnightblue	G38140	G38140.9	PI467895_SS	A0A445BRN5	1,4-alpha-glucan branching enzyme (EC 2.4.1.18)	Glycan biosynthesis; starch biosynthesis. {ECO:0000256|ARBA:ARBA00004727}.
midnightblue	G43352	G43352.8	PI467895_SS	A0A059DAZ5	Biotin synthase (EC 2.8.1.6)	Cofactor biosynthesis; biotin biosynthesis; biotin from 7,8-diaminononanoate: step 2/2. {ECO:0000256|ARBA:ARBA00004942}.
midnightblue	G48245	G48245.4	PI467895_SS	A0A0B0MVI6	Phosphatidyl-N-methylethanolamine N-methyltransferase (EC 2.1.1.71) (Phospholipid methyltransferase) (PLMT)	Phospholipid metabolism; phosphatidylcholine biosynthesis. {ECO:0000256|ARBA:ARBA00004969, ECO:0000256|HAMAP-Rule:MF_03216}.
midnightblue	G53409	G53409.18	PI467895_SS	A0A0S3RLR9	E1 ubiquitin-activating enzyme (EC 6.2.1.45)	Protein modification; protein ubiquitination. {ECO:0000256|ARBA:ARBA00004906}.
midnightblue	G61189	G61189.1	PI467895_SS	A0A314XST5	Fructose-bisphosphate aldolase (EC 4.1.2.13)	Carbohydrate degradation; glycolysis; D-glyceraldehyde 3-phosphate and glycerone phosphate from D-glucose: step 4/4. {ECO:0000256|ARBA:ARBA00004714}.
midnightblue	G61189	G61189.4	PI467895_SS	A0A314XST5	Fructose-bisphosphate aldolase (EC 4.1.2.13)	Carbohydrate degradation; glycolysis; D-glyceraldehyde 3-phosphate and glycerone phosphate from D-glucose: step 4/4. {ECO:0000256|ARBA:ARBA00004714}.
midnightblue	G61189	G61189.7	PI467895_SS	A0A314XST5	Fructose-bisphosphate aldolase (EC 4.1.2.13)	Carbohydrate degradation; glycolysis; D-glyceraldehyde 3-phosphate and glycerone phosphate from D-glucose: step 4/4. {ECO:0000256|ARBA:ARBA00004714}.
midnightblue	G61189	G61189.9	PI467895_SS	A0A314XST5	Fructose-bisphosphate aldolase (EC 4.1.2.13)	Carbohydrate degradation; glycolysis; D-glyceraldehyde 3-phosphate and glycerone phosphate from D-glucose: step 4/4. {ECO:0000256|ARBA:ARBA00004714}.
midnightblue	G66886	G66886.3	PI467895_SS	A0A445GIQ4	Aspartate kinase (EC 2.7.2.4)	Amino-acid biosynthesis; L-lysine biosynthesis via DAP pathway; (S)-tetrahydrodipicolinate from L-aspartate: step 1/4. {ECO:0000256|ARBA:ARBA00004766, ECO:0000256|RuleBase:RU004249}.; Amino-acid biosynthesis; L-methionine biosynthesis via de novo pathway; L-homoserine from L-aspartate: step 1/3. {ECO:0000256|RuleBase:RU004249}.; Amino-acid biosynthesis; L-threonine biosynthesis; L-threonine from L-aspartate: step 1/5. {ECO:0000256|RuleBase:RU004249}.
midnightblue	G71021	G71021.51	PI467895_SS	M5VPR4	Phosphopyruvate hydratase (EC 4.2.1.11)	Carbohydrate degradation; glycolysis; pyruvate from D-glyceraldehyde 3-phosphate: step 4/5. {ECO:0000256|ARBA:ARBA00005031}.
midnightblue	G71844	G71844.13	PI467895_SS	A0A2P5C2H3	Isopentenyl-diphosphate Delta-isomerase (EC 5.3.3.2)	Isoprenoid biosynthesis; dimethylallyl diphosphate biosynthesis; dimethylallyl diphosphate from isopentenyl diphosphate: step 1/1. {ECO:0000256|ARBA:ARBA00004826}.; Porphyrin-containing compound metabolism; chlorophyll biosynthesis. {ECO:0000256|ARBA:ARBA00005173}.
midnightblue	G80640	G80640.8	PI467895_SS	A0A1U8B3G1	3-hydroxy-3-methylglutaryl coenzyme A reductase (HMG-CoA reductase) (EC 1.1.1.34)	Metabolic intermediate biosynthesis; (R)-mevalonate biosynthesis; (R)-mevalonate from acetyl-CoA: step 3/3. {ECO:0000256|ARBA:ARBA00005084, ECO:0000256|RuleBase:RU361219}.
midnightblue	G87941	G87941.84	PI467895_SS	G7K036	Lipoxygenase (EC 1.13.11.-)	Lipid metabolism; oxylipin biosynthesis. {ECO:0000256|RuleBase:RU003975}.
midnightblue	G88952	G88952.3	PI467895_SS	A0A097GX09	S-adenosylmethionine synthase (EC 2.5.1.6)	Amino-acid biosynthesis; S-adenosyl-L-methionine biosynthesis; S-adenosyl-L-methionine from L-methionine: step 1/1. {ECO:0000256|ARBA:ARBA00005224, ECO:0000256|RuleBase:RU000541}.
yellow	G18144	G18144.21	PI467895_SS	A0A4P1RED4	Uncharacterized protein	Lipid metabolism; fatty acid biosynthesis. {ECO:0000256|ARBA:ARBA00005194}.
yellow	G19941	G19941.23	PI467895_SS	A0A444ZCX7	Fumarylacetoacetase (EC 3.7.1.2) (Fumarylacetoacetate hydrolase)	Amino-acid degradation; L-phenylalanine degradation; acetoacetate and fumarate from L-phenylalanine: step 6/6. {ECO:0000256|ARBA:ARBA00004782}.
yellow	G27970	G27970.5	PI467895_SS	A0A4D6NRG5	Probable 6-phosphogluconolactonase	Carbohydrate degradation; pentose phosphate pathway. {ECO:0000256|ARBA:ARBA00004959}.

yellow	G32026	G32026.3	PI467895_SS	A0A5J4ZVF7	Molybdopterin synthase sulfur carrier subunit (Molybdenum cofactor synthesis protein 2 small subunit) (Molybdenum cofactor synthesis protein 2A) (MOCS2A) (Sulfur carrier protein MOCS2A)	Cofactor biosynthesis; molybdopterin biosynthesis. {ECO:0000256|HAMAP-Rule:MF_03051}.
yellow	G42592	G42592.4	PI467895_SS	A0A2P6RGT3	RING-type E3 ubiquitin transferase (EC 2.3.2.27)	Protein modification; protein ubiquitination. {ECO:0000256|ARBA:ARBA00004906}.
yellow	G61340	G61340.2	PI467895_SS	Q42797	Trans-cinnamate 4-monooxygenase (EC 1.14.14.91) (Cinnamic acid 4-hydroxylase) (C4H) (CA4H) (Cytochrome P450 73) (Cytochrome P450C4H)	Phenylpropanoid metabolism; trans-4-coumarate biosynthesis; trans-4-coumarate from trans-cinnamate: step 1/1. {ECO:0000305}.
yellow	G68875	G68875.25	PI467895_SS	A0A1J7HFW6	Inosine-5'-monophosphate dehydrogenase (IMP dehydrogenase) (IMPD) (IMPDH) (EC 1.1.1.205)	Purine metabolism; XMP biosynthesis via de novo pathway; XMP from IMP: step 1/1. {ECO:0000256|HAMAP-Rule:MF_03156, ECO:0000256|RuleBase:RU003928}.
yellow	G69291	G69291.30	PI467895_SS	I3SGX3	Phosphoglycerate mutase (2,3-diphosphoglycerate-independent) (EC 5.4.2.12)	Carbohydrate degradation; glycolysis; pyruvate from D-glyceraldehyde 3-phosphate: step 3/5. {ECO:0000256|ARBA:ARBA00004798}.
yellow	G69291	G69291.46	PI467895_SS	I3SGX3	Phosphoglycerate mutase (2,3-diphosphoglycerate-independent) (EC 5.4.2.12)	Carbohydrate degradation; glycolysis; pyruvate from D-glyceraldehyde 3-phosphate: step 3/5. {ECO:0000256|ARBA:ARBA00004798}.
yellow	G69291	G69291.72	PI467895_SS	I3SGX3	Phosphoglycerate mutase (2,3-diphosphoglycerate-independent) (EC 5.4.2.12)	Carbohydrate degradation; glycolysis; pyruvate from D-glyceraldehyde 3-phosphate: step 3/5. {ECO:0000256|ARBA:ARBA00004798}.
yellow	G77511	G77511.11	PI467895_SS	A0A2I4FTA9	protein AUXIN SIGNALING F-BOX 2-like	Protein modification; protein ubiquitination. {ECO:0000256|ARBA:ARBA00004906}.
yellow	G77511	G77511.14	PI467895_SS	A0A1W6S2G5	F-box/kelch-repeat protein	Protein modification; protein ubiquitination. {ECO:0000256|ARBA:ARBA00004906}.
yellow	G88186	G88186.7	PI467895_SS	A0A4P1RVN0	Uncharacterized protein	Protein modification; protein ubiquitination. {ECO:0000256|ARBA:ARBA00004906}.
yellow	G90732	G90732.3	PI467895_SS	A0A166FHK6	L-gulonolactone oxidase (EC 1.1.3.8)	Cofactor biosynthesis; L-ascorbate biosynthesis. {ECO:0000256|ARBA:ARBA00005147}.
yellow4	G14078	G14078.78	PI467895_SS	Q9ZSQ5	Starch synthase, chloroplastic/amyloplastic (EC 2.4.1.-)	Glycan biosynthesis; starch biosynthesis. {ECO:0000256|ARBA:ARBA00004727, ECO:0000256|RuleBase:RU361232}.
yellow4	G14078	G14078.81	PI467895_SS	A0A3Q0F854	Starch synthase, chloroplastic/amyloplastic (EC 2.4.1.-)	Glycan biosynthesis; starch biosynthesis. {ECO:0000256|ARBA:ARBA00004727, ECO:0000256|RuleBase:RU361232}.
yellow4	G14078	G14078.94	PI467895_SS	A0A3P9QN32	Starch synthase, chloroplastic/amyloplastic (EC 2.4.1.-)	Glycan biosynthesis; starch biosynthesis. {ECO:0000256|ARBA:ARBA00004727, ECO:0000256|RuleBase:RU361232}.
yellow4	G14078	G14078.95	PI467895_SS	A0A3P9QN32	Starch synthase, chloroplastic/amyloplastic (EC 2.4.1.-)	Glycan biosynthesis; starch biosynthesis. {ECO:0000256|ARBA:ARBA00004727, ECO:0000256|RuleBase:RU361232}.
yellow4	G14078	G14078.96	PI467895_SS	A0A3P9QN32	Starch synthase, chloroplastic/amyloplastic (EC 2.4.1.-)	Glycan biosynthesis; starch biosynthesis. {ECO:0000256|ARBA:ARBA00004727, ECO:0000256|RuleBase:RU361232}.
yellow4	G14078	G14078.97	PI467895_SS	A0A3P9QN32	Starch synthase, chloroplastic/amyloplastic (EC 2.4.1.-)	Glycan biosynthesis; starch biosynthesis. {ECO:0000256|ARBA:ARBA00004727, ECO:0000256|RuleBase:RU361232}.
yellow4	G46501	G46501.10	PI467895_SS	B6ECZ0	Acyl-[acyl-carrier-protein] desaturase (EC 1.14.19.-)	Lipid metabolism; fatty acid metabolism. {ECO:0000256|ARBA:ARBA00004872}.
yellow4	G69291	G69291.34	PI467895_SS	A0A4D6NGT5	Phosphoglycerate mutase (2,3-diphosphoglycerate-independent) (EC 5.4.2.12)	Carbohydrate degradation; glycolysis; pyruvate from D-glyceraldehyde 3-phosphate: step 3/5. {ECO:0000256|ARBA:ARBA00004798}.
paleturquoise	G8783	G8783.4	PI467895_SS	A0A4Y7KDI9	Urea amidohydrolase (EC 3.5.1.5) (Urease)	Nitrogen metabolism; urea degradation; CO(2) and NH(3) from urea (urease route): step 1/1. {ECO:0000256|ARBA:ARBA00004897}.
paleturquoise	G15684	G15684.27	PI467895_SS	A0A445DQT7	Dolichol-phosphate mannosyltransferase subunit 1 (EC 2.4.1.83)	Protein modification; protein glycosylation. {ECO:0000256|RuleBase:RU365083}.
paleturquoise	G16816	G16816.1	PI467895_SS	A0A0S3R8S2	S-adenosylmethionine synthase (EC 2.5.1.6)	Amino-acid biosynthesis; S-adenosyl-L-methionine biosynthesis; S-adenosyl-L-methionine from L-methionine: step 1/1. {ECO:0000256|ARBA:ARBA00005224, ECO:0000256|RuleBase:RU000541}.
paleturquoise	G28022	G28022.3	PI467895_SS	A0A0B2R0N3	Chalcone-flavonone isomerase family protein	Secondary metabolite biosynthesis; flavonoid biosynthesis. {ECO:0000256|ARBA:ARBA00004966}.
paleturquoise	G46820	G46820.1	PI467895_SS	A0A059DAZ5	Biotin synthase (EC 2.8.1.6)	Cofactor biosynthesis; biotin biosynthesis; biotin from 7,8-diaminononanoate: step 2/2. {ECO:0000256|ARBA:ARBA00004942}.
paleturquoise	G47029	G47029.25	PI467895_SS	A0A2G9GAK7	Dolichyl-diphosphooligosaccharide--protein glycosyltransferase 48 kDa subunit (Oligosaccharyl transferase 48 kDa subunit)	Protein modification; protein glycosylation. {ECO:0000256|ARBA:ARBA00004922, ECO:0000256|RuleBase:RU361142}.

paleturquoise	G50555	G50555.20	PI467895_SS	A0A067FZR7	RING-type E3 ubiquitin transferase (EC 2.3.2.27)	Protein modification; protein ubiquitination. {ECO:0000256|ARBA:ARBA00004906}.
paleturquoise	G54773	G54773.110	PI467895_SS	A0A444WS55	Succinate dehydrogenase [ubiquinone] flavoprotein subunit, mitochondrial (EC 1.3.5.1)	Carbohydrate metabolism; tricarboxylic acid cycle; fumarate from succinate (eukaryal route): step 1/1. {ECO:0000256|RuleBase:RU362051}.
paleturquoise	G58439	G58439.8	PI467895_SS	A0A444Y924	Phospho-2-dehydro-3-deoxyheptonate aldolase (EC 2.5.1.54)	Metabolic intermediate biosynthesis; chorismate biosynthesis; chorismate from D-erythrose 4-phosphate and phosphoenolpyruvate: step 1/7. {ECO:0000256|ARBA:ARBA00004688, ECO:0000256|RuleBase:RU363071}.
paleturquoise	G69291	G69291.55	PI467895_SS	I3SGX3	Phosphoglycerate mutase (2,3-diphosphoglycerate-independent) (EC 5.4.2.12)	Carbohydrate degradation; glycolysis; pyruvate from D-glyceraldehyde 3-phosphate: step 3/5. {ECO:0000256|ARBA:ARBA00004798}.
paleturquoise	G71021	G71021.34	PI467895_SS	A0A2C9VE87	Phosphopyruvate hydratase (EC 4.2.1.11)	Carbohydrate degradation; glycolysis; pyruvate from D-glyceraldehyde 3-phosphate: step 4/5. {ECO:0000256|ARBA:ARBA00005031}.
paleturquoise	G71021	G71021.48	PI467895_SS	A0A5B7C0H9	Phosphopyruvate hydratase (EC 4.2.1.11)	Carbohydrate degradation; glycolysis; pyruvate from D-glyceraldehyde 3-phosphate: step 4/5. {ECO:0000256|ARBA:ARBA00005031}.
paleturquoise	G73951	G73951.32	PI467895_SS	A0A438I1T0	Phosphopyruvate hydratase (EC 4.2.1.11)	Carbohydrate degradation; glycolysis; pyruvate from D-glyceraldehyde 3-phosphate: step 4/5. {ECO:0000256|ARBA:ARBA00005031}.
paleturquoise	G83175	G83175.25	PI467895_SS	A0A0R0FCJ9	Quinolinate synthase (EC 2.5.1.72)	Cofactor biosynthesis; NAD(+) biosynthesis; quinolinate from iminoaspartate: step 1/1. {ECO:0000256|ARBA:ARBA00005065}.
black	G5723	G5723.7	PI467895_SS	A0A5N6RBS0	Phosphotransferase (EC 2.7.1.-)	Carbohydrate degradation; glycolysis; D-glyceraldehyde 3-phosphate and glycerone phosphate from D-glucose: step 1/4. {ECO:0000256|ARBA:ARBA00004888}.; Carbohydrate metabolism; hexose metabolism. {ECO:0000256|ARBA:ARBA00005028}.
black	G10338	G10338.8	PI467895_SS	A0A5D2RYS9	Pyruvate kinase (EC 2.7.1.40)	Carbohydrate degradation; glycolysis; pyruvate from D-glyceraldehyde 3-phosphate: step 5/5. {ECO:0000256|ARBA:ARBA00004997, ECO:0000256|RuleBase:RU000504}.
black	G19964	G19964.6	PI467895_SS	A0A445EL27	Pyrophosphate--fructose 6-phosphate 1-phosphotransferase subunit beta (PFP) (EC 2.7.1.90) (6-phosphofructokinase, pyrophosphate dependent) (PPi-PFK) (Pyrophosphate-dependent 6-phosphofructose-1-kinase)	Carbohydrate degradation; glycolysis; D-glyceraldehyde 3-phosphate and glycerone phosphate from D-glucose: step 3/4. {ECO:0000256|ARBA:ARBA00004679, ECO:0000256|HAMAP-Rule:MF_03185}.
black	G24479	G24479.1	PI467895_SS	A0A1R3KEY6	Phosphopyruvate hydratase (EC 4.2.1.11)	Carbohydrate degradation; glycolysis; pyruvate from D-glyceraldehyde 3-phosphate: step 4/5. {ECO:0000256|ARBA:ARBA00005031}.
black	G25577	G25577.3	PI467895_SS	A0A1S3VJU5	Allantoinase (EC 3.5.2.5)	Nitrogen metabolism; (S)-allantoin degradation; allantoate from (S)-allantoin: step 1/1. {ECO:0000256|ARBA:ARBA00004968}.
black	G25915	G25915.1	PI467895_SS	I3SQQ9	Orotidine 5'-phosphate decarboxylase (EC 4.1.1.23) (Uridine 5'-monophosphate synthase)	Pyrimidine metabolism; UMP biosynthesis via de novo pathway; UMP from orotate: step 2/2. {ECO:0000256|ARBA:ARBA00004861}.
black	G29029	G29029.2	PI467895_SS	A0A2R6R298	Molybdopterin synthase sulfur carrier subunit (Molybdenum cofactor synthesis protein 2 small subunit) (Molybdenum cofactor synthesis protein 2A) (MOCS2A) (Sulfur carrier protein MOCS2A)	Cofactor biosynthesis; molybdopterin biosynthesis. {ECO:0000256|HAMAP-Rule:MF_03051}.
black	G30758	G30758.21	PI467895_SS	A0A453MAW5	Urease (EC 3.5.1.5) (Urea amidohydrolase)	Nitrogen metabolism; urea degradation; CO(2) and NH(3) from urea (urease route): step 1/1. {ECO:0000256|ARBA:ARBA00004897, ECO:0000256|PIRNR:PIRNR001222}.
black	G37082	G37082.9	PI467895_SS	I3SEQ9	Serine hydroxymethyltransferase (EC 2.1.2.1)	One-carbon metabolism; tetrahydrofolate interconversion. {ECO:0000256|ARBA:ARBA00004777, ECO:0000256|RuleBase:RU000585}.
black	G47029	G47029.1	PI467895_SS	I1LQ58	Dolichyl-diphosphooligosaccharide--protein glycosyltransferase 48 kDa subunit (Oligosaccharyl transferase 48 kDa subunit)	Protein modification; protein glycosylation. {ECO:0000256|ARBA:ARBA00004922, ECO:0000256|RuleBase:RU361142}.
black	G50682	G50682.4	PI467895_SS	C6T1M8	Lactoylglutathione lyase (EC 4.4.1.5) (Glyoxalase I)	Secondary metabolite metabolism; methylglyoxal degradation; (R)-lactate from methylglyoxal: step 1/2. {ECO:0000256|ARBA:ARBA00005008, ECO:0000256|RuleBase:RU361179}.
black	G54931	G54931.1	PI467895_SS	A0A4P1RFV7	Acyl-[acyl-carrier-protein] desaturase (EC 1.14.19.-)	Lipid metabolism; fatty acid metabolism. {ECO:0000256|ARBA:ARBA00004872}.
black	G71844	G71844.6	PI467895_SS	A0A067EG43	Isopentenyl-diphosphate Delta-isomerase (EC 5.3.3.2)	Isoprenoid biosynthesis; dimethylallyl diphosphate biosynthesis; dimethylallyl diphosphate from isopentenyl diphosphate: step 1/1. {ECO:0000256|ARBA:ARBA00004826}.; Porphyrin-containing compound metabolism; chlorophyll biosynthesis. {ECO:0000256|ARBA:ARBA00005173}.

Supplementary Table 16. Summary of ncRNAs mechanisms of action in drought and salt stress.

Stress	ncRNAs	Name	Mechanism	Plant	Ref
Drought	lncRNA	XLOC_012868, XLOC_052298 and XLOC_094954	ABA signaling pathways and signal transport and defense/stress response	*Brassica napus*	1
	lncRNA	*DRIR*	ABA- mediated stress response: RD29A and RD29B	Arabidopsis	2
	circRNA	circGORK	Guard cell outward-rectifying K+-channel). ABA signaling and water stress responses	Maize and Arabidopsis	3
	lncRNA	lincRNA340	miR169-targeted Nuclear Factor Y (NF-Y)	*M. esculenta*	4
Salt	lncRNA	TCONS_00046739 and TCONS_00020253	cytochrome P450 and Na+/H+ ex- changer (NHX)	*M. truncatula*	5
	lncRNA	lnc_883	Modulation in expression of mechanosensitive ion channel (Gh_D03G0339)	*Gossypium hirsutum*	6
	circRNA	Chr3:25629954|25,631,297, Chr2:16186442|16,191,680, Chr3:7346435|7,347,924 and Chr5:2818033|2,823,737	Vacuolar cation/H+ exchanger 2-like (CHX2-like), K+ efflux antiporter (KEA2-like and KEA6-like) and sodium/hydrogen exchanger 7-like protein, (NHX7-like)	*Cucumis sativus*	7
	lncRNA	*DRIR*	ABA-regulated stress tolerance: P5CS1, RD29A, RD29B, FUT4, ANNAT7, NAC3, and AtrbohB. Enhancing water transport: TIP4 and NIP1	Arabidopsis	2
	lncRNA	lncRNA973	Downregulation of ROS-scavenging genes SOD, CAT and POD	*Gossypium hirsutum*	8

References

1 Tan, X., Li, S., Hu, L. & Zhang, C. Genome-wide analysis of long non-coding RNAs (lncRNAs) in two contrasting rapeseed (*Brassica napus* L.) genotypes subjected to drought stress and re-watering. *BMC Plant Biol.* **20**, 81 (2020).

2 Qin, T., Zhao, H., Cui, P., Albesher, N. & Xiong, L. A Nucleus-Localized Long Non-Coding RNA Enhances Drought and Salt Stress Tolerance. *Plant Physiol.* **175**, 1321–1336 (2017).

3 Zhang, P. *et al.* A large-scale circular RNA profiling reveals universal molecular mechanisms responsive to drought stress in maize and Arabidopsis. *Plant J.* **98**, 697–713 (2019).

4 Li, S. *et al.* Genome-wide identification and functional prediction of cold and/or drought-responsive lncRNAs in cassava. *Sci. Rep.* **7**, 45981 (2017).

5 Wang, T.-Z., Liu, M., Zhao, M.-G., Chen, R. & Zhang, W.-H. Identification and characterization of long non-coding RNAs involved in osmotic and salt stress in *Medicago truncatula* using genome-wide high-throughput sequencing. *BMC Plant Biol.* **15**, 131 (2015).

6 Deng, F., Zhang, X., Wang, W., Yuan, R. & Shen, F. Identification of *Gossypium hirsutum* long non-coding RNAs (lncRNAs) under salt stress. *BMC Plant Biol.* **18**, 23 (2018).

7 Zhu, Y. X. *et al.* Identification of cucumber circular RNAs responsive to salt stress. *BMC Plant Biol.* **19**, 1–18 (2019).

8 Zhang, X. *et al.* The long non-coding RNA lncRNA973 is involved in cotton response to salt stress. *BMC Plant Biol.* **19**, 459 (2019).
